# A systematic review on the associations between the built environment and adult’s physical activity in global tropical and subtropical climate regions

**DOI:** 10.1186/s12966-024-01582-x

**Published:** 2024-05-21

**Authors:** Carina Nigg, Shaima A. Alothman, Abdullah F. Alghannam, Jasper Schipperijn, Reem AlAhmed, Reem F. Alsukait, Severin Rakic, Volkan Cetinkaya, Hazzaa M. Al-Hazzaa, Saleh A. Alqahtani

**Affiliations:** 1 Institute of Social and Preventive Medicine, University of Bern, Mittelstrasse 43, Bern, 3012 Switzerland; 2 Lifestyle and Health Research Center, Health Sciences Research Center, Princess Nourah bint Abdulrahman University, PO Box 47330, Riyadh, 11552 Saudi Arabia; 3 Department of Sports Science and Clinical Biomechanics, University of Southern Denmark, Campusvej 39, Odense, 5230 Denmark; 4 Biostatistics, Epidemiology and Scientific Computing Department (BESC), King Faisal Specialist Hospital & Research Center, Riyadh, Saudi Arabia; 5 Community Health Sciences, King Saud University, PO Box 145111, Riyadh, 11362 Saudi Arabia; 6 The World Bank, 1818 H Street N.W, Washington, DC 20433 USA; 7 Liver Transplant Center, King Faisal Specialist Hospital & Research Center, Riyadh, 11564 Saudi Arabia; 8 Division of Gastroenterology and Hepatology, Johns Hopkins University, Baltimore, MD 21287 USA; 9 School of Sport Sciences, University of Jordan, King Abdullah II St, Amman, Jordan

**Keywords:** Active travel, Climate change, Exercise, Health-enhancing physical activity, Heat, Tropical

## Abstract

**Background:**

Physical inactivity is a major public health concern, exacerbated in countries with a (sub)tropical climate. The built environment can facilitate physical activity; however, current evidence is mainly from North American and European countries with activity-friendly climate conditions. This study explored associations between built environment features and physical activity in global tropical or subtropical dry or desert climate regions.

**Methods:**

A systematic review of four major databases (Web of Science, Scopus, PubMed, and SportDISCUS) was performed. To be included, studies had to investigate associations between perceived or objective built environment characteristics and adult’s physical activity and had to be conducted in a location with (sub)tropical climate. Each investigated association was reported as one case and results were synthesized based upon perceived and objectively assessed environment characteristics as well as Western and non-Western countries. Study quality was evaluated using a tool designed for assessing studies on built environment and physical activity.

**Results:**

Eighty-four articles from 50 studies in 13 countries with a total of 2546 built environment-physical activity associations were included. Design (connectivity, walking/cycling infrastructure), desirability (aesthetics, safety), and destination accessibility were the built environment characteristics most frequently associated with physical activity across the domains active transport, recreational physical activity, total walking and cycling, and moderate-to-vigorous physical activity, particularly if multiple attributes were present at the same time. Very few studies assessed built environment attributes specifically relevant to physical activity in (sub)tropical climates. Most studies were conducted in Western countries, with results being largely comparable with non-Western countries. Findings were largely generalizable across gender and age groups. Results from natural experiments indicated that relocating to an activity-friendly neighborhood impacted sub-groups differently.

**Conclusions:**

Built environment attributes, including destination accessibility, connectivity, walking and cycling infrastructure, safety, and aesthetics, are positively associated with physical activity in locations with (sub)tropical climate. However, few studies focus on built environment attributes specifically relevant in a hot climate, such as shade or indoor recreation options. Further, there is limited evidence from non-Western countries, where most of the urban population lives in (sub)tropical climates. Policy makers should focus on implementing activity-friendly environment attributes to create sustainable and climate-resilient cities.

**Supplementary Information:**

The online version contains supplementary material available at 10.1186/s12966-024-01582-x.

## Introduction

Physical activity is one of the most powerful health behaviors to prevent chronic diseases and promote well-being [1]. However, almost 30% of adults worldwide are not active enough, i.e., they do not engage in 150 min of moderate-to-vigorous intensity physical activity (MVPA) per week [2]. The non-communicable diseases and mental health issues associated with inactivity are expected to incur treatment costs of US$ 27 billion annually by 2030 if physical inactivity prevalence remains unchanged [2].

In places with tropical or subtropical climate, physical activity engagement can be even more challenging due to high temperatures. According to two reviews, hot weather was a barrier to physical activity in Middle Eastern and North African countries [3, 4]. A two-year longitudinal study in the Arabian Gulf showed an inverse relationship between temperatures and step count: Daily step count was largest (∼8700) at 15–25°C, which dropped to 7600 during high temperatures (35–40°C) [5]. A study in Australia showed similar results, with light physical activity and MVPA peaking at 31°C and 27°C, respectively, then dropping when going above these temperatures [6]. A previous review showed that extreme weather events, especially hot temperatures, negatively affect physical activity [7]. From a health perspective, physical activity in high temperatures can result in serious medical conditions, e.g., heat exhaustion and heat stroke [8]. For vulnerable groups, such as pregnant women and older adults, it is recommended to avoid physical activity during heat [1, 9]. The relationship between temperature and physical activity is globally relevant since 43% of the world’s population currently lives in tropical areas, which is expected to grow to over 50% by 2050 [10]. In addition, extreme weather events, including hot and dry periods and extreme heat waves, will occur more frequently worldwide due to climate change [11, 12]. This makes it important to understand how physical activity can be promoted during high temperature periods.

The World Health Organization’s (WHO) *Global Action Plan on Physical Activity* emphasizes creating active environments for physical activity promotion [13]. Five systematic reviews found that improvements in active transport infrastructure, walkability characteristics, provision of high-quality parks, and objective destination accessibility positively impacted physical activity and active transport [14–18]. However, studies included in these reviews focused predominantly on Western countries in Europe and the USA [14–16, 18], mainly with a cool or warm climate [19], limiting generalizability to places with a hot climate and extreme heat. Due to the high temperatures and solar radiation, the environmental needs are likely different from those in a cool or warm climate. For example, in a study in Singapore and Kuala Lumpur, both with a tropical climate, citizens highlighted the need for shade and preservation of public open space to allow year-round activity, while citizens in Delhi, experiencing both hot and cold weather phases, did not deem this as important [20]. People in Kuala Lumpur worried about skin darkening and cancer from sun exposure while walking [21], which could be prevented with shade along walking paths. Providing access to indoor facilities (e.g., shopping malls and recreational facilities) may be even more important in places with high temperatures to facilitate physical activity at local destinations where temperatures can be regulated [3].

While tropical and subtropical climate conditions are mostly present in non-Western countries [19], we lack reviews that compare results from Western or non-Western locations. Apart from the high temperatures, this could be relevant since non-Western cultures, e.g., in the Middle East, have their own unique built environment design traditions that are often overlooked when focusing on Western cultures and related environments [22], while urban form characteristics also show differences, e.g., in population density and land use mix [23]. This Western bias has been previously noticed in reviews on the built environment and physical activity [18] and should be addressed in further evidence synthesis. The primary purpose of this review was to synthesize results from previous studies on associations between built environment features and physical activity in global tropical or subtropical dry or desert climate regions. The secondary purpose was to investigate associations stratified by Western and non-Western locations.

## Methods

This systematic review followed the reporting of the Preferred Reporting Items for Systematic Reviews and Meta Analyses (PRISMA) guidelines [24] and was registered in PROSPERO (CRD42022362485). We report the core information regarding the methods in the following and provide more details on the methodology in Additional File 1.

### Search strategy and information sources

**Fig. 1:**
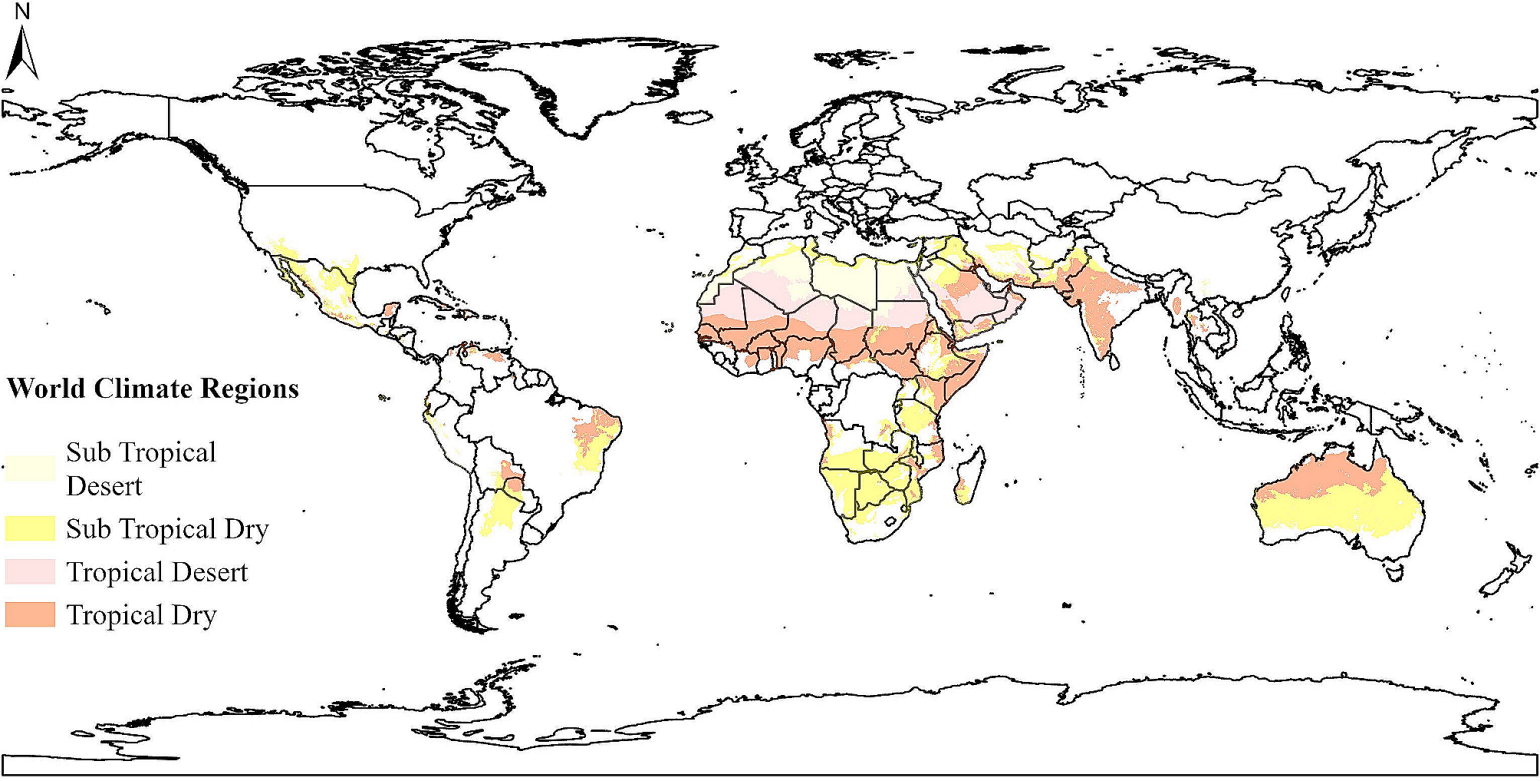
Locations considered for this review with a tropical or subtropical dry or desert climate [19]

A systematic search was performed in the four major databases Web of Science, Scopus, PubMed, and SportDISCUS (via EBSCOhost) from inception until 16 November 2022 based upon terms for physical activity, the built environment, and study locations in relevant World Climate Regions (see Additional File 2). The World Climate Regions were determined based upon temperatures and aridity, following the International Panel on Climate Change’s (IPCC) definitions [19]. For this review, we were interested in study locations with a tropical or subtropical climate (24–34°C and 18–24°C mean annual temperature, respectively) with an arid or hyper-arid moisture index (< 0.65; see Fig. 1).

### Eligibility criteria

Article eligibility criteria were: (1) Adults (≥ 18 years) without physical disabilities in a dry or desert tropical or subtropical climate (19; see also Additional File 2) as study population; (2) quantitative; (3) peer-reviewed; (4) published in English, German, Dutch, Danish, or Arabic based upon the authors’ language skills; and (5) study assessed the relationship between any perceived or objective built environment characteristics and any form of self-reported or device-assessed individual-level physical activity behavior. The built environment was conceptualized as the physical form of communities, including land use patterns, built and natural features, and the transportation system [25].

### Selection, data collection process, and data extraction

Records obtained from the database search were imported into EndNote X9 (version 3.3) and de-duplicated [26] before being uploaded into ASReview, an open-source machine-learning tool for arranging the records based on their relevance [27]. Two reviewers independently screened titles and abstracts in ASReview. Following modeling studies [27] and previous health sciences practices [28], we screened 35% of the records in the presented order, and until 60 studies in a row were labeled as irrelevant. Any record labeled relevant by at least one reviewer was included for full-text screening. To ensure that no relevant records were missed, we trained the model again with a more sophisticated algorithm.

Full texts were screened independently by two investigators. Disagreements were discussed and if no agreement could be reached, a third investigator was consulted. The included full texts were then used to identify additional eligible articles via backward- and forward-citation screening using the Shiny app *Citationchaser* [29]. Data extraction was conducted independently by two investigators for 10% of the included articles and continued by one investigator after an 80% agreement rate was reached [30]. The following information was extracted for each article: 1) study name, author, and publication year; 2) participant and location information; 3) study design and sampling strategy; 4) environmental exposure and measures; 5) physical activity type and measures; 6) statistical analysis;’ and 7) results (see Additional File 3). To avoid reporting bias, built environment variables not considered in multivariable analysis due to low significance (as defined by the respective authors) in univariable analysis were included in the data extraction table as null associations.

### Article quality assessment

Article quality was independently assessed by two investigators. Disagreements were discussed and if no agreement could be reached, a third investigator was consulted. Article quality was assessed using ten criteria that were previously developed and used in similar reviews [17, 18]. The criteria included: (a) study design; (b) study areas or participant recruitment stratified by key environmental attributes; (c) participant response rate ≥ 60% or evidence of representativeness; (d) outcome measurements reliable and valid; (e) adjustment for sociodemographic covariates (at least age, sex, and education or similar); (f) adjustment for self-selection; suitable analytical approach (g-i), including (g) adjustment for clustering; (h) approach for distributional assumptions; (i) correct implementation and presentation of results; (j) no inappropriate categorization of continuous environmental exposures. We added the criterion k) validity of the environmental exposure measure. A score of 0, 1, and 2 was assigned for cross-sectional, longitudinal, and quasi-experimental/natural experiment studies, respectively. Criteria regarding the analytical approach (g-i) were scored 1/3 if answered “yes” and 0 if answered “no”, respectively. For all other items, a score of 1 was assigned if answered “yes” and 0 if answered “no” (see Additional File 4). To establish quality categories, we applied the previous categorization of Barnett and colleagues [11], but transformed the cut-off points into percentages since for some studies, the criterion “adjustment for clustering” did not apply, resulting in a total score of nine instead of ten points. Thus, articles were categorized into low (< 38.9% of total points), medium (39.0-65.6%), and high (> 65.6%) quality.

### Data synthesis

All data were entered into SPSS (version 29) for further processing, and each studied association was recorded as one case. For example, if a study investigated the association between aesthetics and walking and between aesthetics and MVPA, this would be recorded as two cases. Next, environmental attributes were assigned to one of the “11D” categories, which describe urban transport planning and design features that are hypothesized to enhance physical activity [31, 32] (see Table 1). For categories containing diverse environmental features, environmental sub-categories were specified based on the 11D’s description [31, 32]. Sub-categories were considered as “favorable features” if they were phrased or hypothesized to relate positively to physical activity (e.g., feeling safe walking along the street), and as “unfavorable features” if they were phrased or hypothesized to relate negatively to physical activity (e.g., feeling unsafe walking along the street). Since the 11D’s are understood as a framework of integrated interventions to create healthy and sustainable cities, this means that some features could be assigned to multiple categories [32, 33]. Variables were assigned to the category they fit best based upon their operationalization. For example, in the long version of the frequently used Neighborhood Environment Walkability Scale (NEWS), land use mix is included as a sub-scale twice: (a) one sub-scale representing time to walk to different destinations in the neighborhood, such as restaurants, parks, public transport, or work/school, and (b) one subscale representing accessibility of destinations through a combination of items asking about access to shopping at local stores, public transit, and topography [34]. Due to their different operationalizations, land use mix sub-scale (a) was assigned to “diverse housing and land use”, and sub-scale (b) to “destination accessibility”. Public transport is another example: If an item asked merely about distance to public transport (e.g., distance to the next bus stop), this was assigned to “distance to public transport”. If the item or scale was more about the quality of public transport (e.g., the number of bus routes through the neighborhood), public transport was assigned to “destination accessibility”. The same applied to walking and cycling infrastructure: If the item asked about availability of walking infrastructure, this was assigned to “design”; if the item was more about the quality of the infrastructure (e.g., benches along sidewalks), this was assigned to “destination accessibility”. The category “multicomponent” was added for environmental scores consisting of multiple 11D categories impossible to separate into single 11D categories, such as the walkability index [35].

Physical activity outcomes were assigned to the categories transport physical activity, recreational physical activity, total walking and cycling, and MVPA, with the two former ones representing important physical activity domains [36]. We determined study region [37] and World Bank income information [38] for each study location. For the synthesis, we split the cases into main effects (analysis for the whole sample) and cases with moderation or stratified analysis (e.g., stratified analysis by gender). For the main effects, positive, null, and negative associations were specified for each environment-outcome association based on the reported statistical results and synthesized for each 11D and physical activity categories. For each 11D category, we were interested in the total, i.e., how much each of the 11D categories supports physical activity. For this, associations for unfavorable features had to be reversed. The number of positive associations of the favorable features were added up with the number of negative associations of the unfavorable features and vice versa, as well as the null associations. To demonstrate this on an example: If traffic safety (favorable feature) had 2 positive, 7 null, and 2 negative association, and traffic hazards and concerns (unfavorable feature) had 1 positive, 3 null, and 4 negative associations, the total would be 6 positive, 10 null, and 3 negative associations.

Results were usually extracted from fully adjusted models. However, some studies first investigated bivariable associations between built environment characteristics and physical activity, and then only included variables below a specified p-value in the final analysis. Synthesis was further stratified by perceived and objective built environment measures due to representing different concepts [39, 40] and potentially different associations with physical activity [41, 42].

We further synthesized the results based upon study region, comparing Western and non-Western countries [37] (see Additional File 5). For cases with stratified analysis (e.g., separate gender analysis), the effect for each environment-outcome association was recorded for each stratum separately. For moderation analysis, it was specified if a moderation effect was present. If so, it was noted how the moderating variable (e.g., socio-economic status) impacted the built environment-physical association. Results were again synthesized based on 11D’s and physical activity categories (see Additional File 6). To explore the robustness of our results, we additionally synthesized the results only including studies rated as high quality (see Additional File 7).

**Table 1:** The 11D’s used to categorize environmental features based upon Giles-Corti et al. [31, 32]

Category	Features	Examples based upon the extracted data
Destination accessibility	Accessibility and quality of shops and services for daily living, regional employment, and public transport conveniently accessible	*Land use mix accessibility; number or recreational destinations accessible by walking, park safety; friendly topography (= area with few or no hills)*
Destination proximity	Distance to local destinations	*Proximity to parks, work destination within 1 km service area*
Distribution of employment	Adequate employment mix available across an area	*Not available in this data*
Demand management	Pricing policies and parking supply decreasing the attractiveness of car driving and increasing attractiveness of public and active transport	*Lack of parking, retail-floor-area ratio*
Design	Urban design incorporating street networks that minimize distances between destinations for daily living and homes, lot layouts that facilitate surveillance and increase residential density, public open space, reduced traffic exposure, safe walking, cycling, and public transport networks to create walkable catchments around activity centers	*Street connectivity; few cul-de-sacs; footpath length*
Density	Adequate residential densities to ensure high-frequency public transport and maintain local businesses	*Residential density; block density*
Distance to public transport	Short walking distance to high-frequency public transport from home	*Distance to nearest bus stop; bus stop within 400 m*
Diverse housing and land use	Different housing types mixed with recreational, commercial, and public opportunities in residential areas	*Land use mix; number of dwelling types*
Desirability	Convenient, comfortable, affordable, and safe neighborhood design with attractive, safe, and accessible public transport	*Traffic-slowing devices in the neighborhood; crime safety; neighborhood aesthetics*
Disaster mitigation	Measures to mitigate and adapt to a hot climate or climate change	*Tree canopy cover; greenness*
Distribution of interventions and resources	Design features that promote equal access to health-enhancing environments and avoid gentrification	*Affordable housing (only available as part of the multicomponent category)*
Multicomponent	Environmental features comprising more than one “D”-category	*Walkability index, new-urbanist designed developments*

*Note* The category “multicomponent” was added for environmental indices consisting of more than one of the 11D categories

## Results

### Study characteristics

**Fig. 2:**
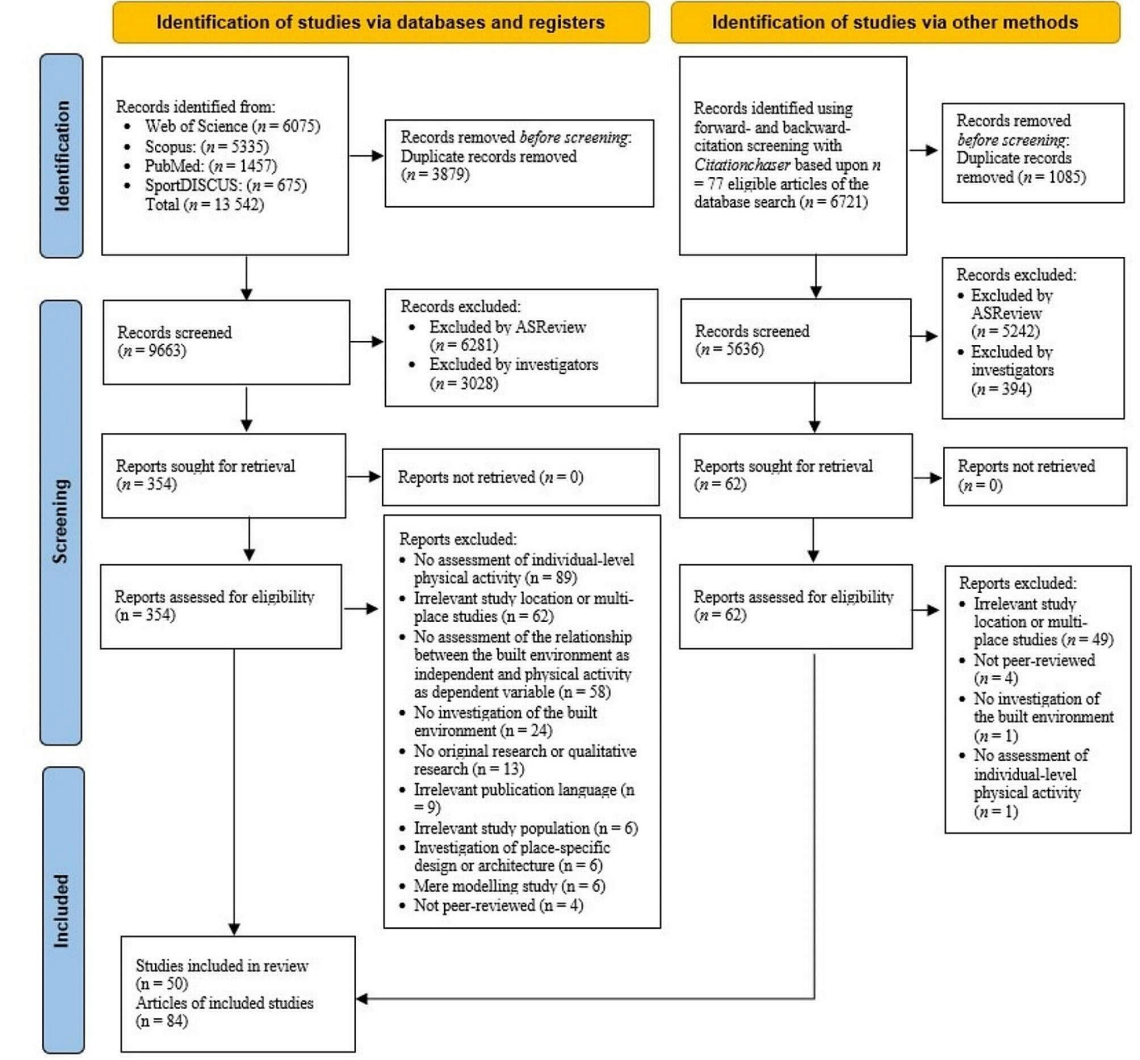
Flowchart of study selection process

The study selection process is presented in Fig. 2. Screenings was ceased after 3,382 titles and abstracts in the presented order (35% of the search results). Eighty-four articles from 50 studies [43–126] making up a total of 2546 environment-physical activity associations were included in this review. Most articles reported results from a single study (*n* = 39), followed by articles from the Residential Environment Project (RESIDE) in Australia (*n* = 18), the International Physical Activity and Environment Network (IPEN) Adult Study in Mexico (*n* = 5), and the Gated Communities Physical Activity Study (*n* = 4) in Pakistan. Articles were published between 1996 and 2022. Results were predominantly reported from urban settings (*n* = 66) and Western countries (*n* = 50). Most articles covered findings from Australia (*n* = 28; 33.3%) and the USA (*n* = 22; 26.2%), followed by Saudi Arabia, Mexico, and Nigeria (each *n* = 6; 7.1%). The sample size ranged from *n* = 39 to *n* = 15,148 participants (*median* = 670.5, *mean* = 1152, *SD* = 1804). Most articles applied a cross-sectional design (*n* = 73; including one retrospective study), followed by natural experiments (*n* = 7), and observational longitudinal studies (*n* = 4). Physical activity was predominantly assessed via self-report (*n* = 78). Device-based assessment via accelerometers (*n* = 3) as well as self-report and accelerometer (*n* = 3) was rare. Most articles assessed the perceived environment (*n* = 39), followed by articles with both objective and perceived environment assessments (*n* = 23) and articles solely assessing the objective environment (*n* = 22). Within the multicomponent category, the most frequent constructs were new-urbanist designed developments (*n* = 170; 57% of cases within this category), followed by walkability and walking-friendly environment indices (*n* = 80; 26.8%).

## Article quality

Of the 84 articles, 15.5% were considered high quality, 67.9% moderate quality, and 16.7% low quality. The mean percentage of total points reached was 50.5% (median: 50.8%), the lowest score was 11.1% (1/9 points), and the highest score was 84.9% (8.5/10 points).

### Synthesis of built environment associations with active transport

Main effect associations between the built environment and active transport were investigated in 38 studies (see Table 2), including 30 cross-sectional [43, 46, 58, 59, 62, 65, 73, 78, 81–83, 85, 87, 90, 92–94, 97–100, 102, 105, 107, 112, 119, 122, 124–126], three longitudinal [66, 67, 89], and five quasi-experimental studies [51, 56, 60, 70, 101], resulting in a total of 492 associations (*n* = 402 in Western countries). The most frequent positive associations occurred for the multicomponent category (*n* = 31 positive, *n* = 32 null associations), followed by destination accessibility (*n* = 24 positive, *n* = 57 null associations), specifically for destination mix, parks, recreational facilities, and shops and services for daily living. Design features (*n* = 19 positive, *n* = 68 null associations) also showed frequent positive associations, especially in relation to connectivity as well as walking and cycling infrastructure supporting active transport. Disaster mitigation, e.g., trees and shade (*n* = 3 positive, *n* = 11 null associations) and demand management (*n* = 2 null associations) were the least investigated.

Stratifying the associations by study region showed that if results were available for Western and non-Western locations, the direction of the relationship was mostly similar across study regions. Benefits of desirability features seemed to be more pronounced in non-Western (*n* = 8 positive and *n* = 16 null associations) compared to Western countries (*n* = 7 positive and *n* = 57 null associations; see Additional File 5).

### Synthesis of built environment associations with recreational physical activity

Main effect associations between the built environment and recreational physical activity were investigated in 39 studies (see Table 3), including 32 cross-sectional [46, 59, 63, 65, 73, 77, 81–83, 85, 87, 90, 92, 93, 97–99, 105, 107, 110–112, 114, 116, 119, 120, 123–126], two longitudinal [66, 67], and five natural experimental studies [51, 56, 60, 61, 70], resulting in a total of 568 investigated associations (*n* = 457 in Western countries). The most frequent favorable associations occurred for desirability (*n* = 32 positive, *n* = 95 null associations), especially in relation with aesthetically pleasing environments and general safety. The second most frequent associations occurred for the multi-component category (*n* = 25 positive, *n* = 39 null associations), followed by design features, specifically connectivity and walking/cycling infrastructure (*n* = 20 positive, *n* = 77 null associations). Disaster mitigation (*n* = 1 positive, *n* = 16 null associations), distance to public transport (*n* = 16 null associations), and demand management (*n* = 3 null associations) were the least investigated. If associations were investigated across both Western and non-Western locations, associations were similar across study regions, while the benefit of desirability features seemed to be more pronounced in non-Western countries (*n* = 9 positive and *n* = 15 null associations) compared to Western countries (*n* = 23 positive and *n* = 80 null associations; see Additional File 5).

**Table 2:** Synthesis of built environment associations with active transport

11D-category	Sub-category	Perceived			Objective			Total		
		*+*	*0*	*-*	*+*	*0*	*-*	*+*	*0*	*-*
Demand management	Parking restrictions		1	1						
	Parking support		1							
	***Demand management total***	*0*	*2*	*1*	*0*	*0*	*0*	***0***	***2***	***1***
Density		*1*	*5*	*0*	*4*	*18*	*1*	***5***	***23***	***1***
Design	Connectivity	2	15	1	7	8				
	Walking/cycling infrastructure	2	21		4	8				
	Lot layout				2	2				
	*Total favorable features*	*4*	*36*	*1*	*13*	*18*	*0*	*17*	*54*	*1*
	Unfavorable connectivity features			2	1	11				
	Unfavorable walking/cycling infrastructure		3							
	*Total unfavorable features*	*0*	*3*	*2*	*1*	*11*	*0*	*1*	*14*	*2*
	***Design total***							***19***	***68***	***2***
Desirability	General safety	2	4	1						
	Crime safety	2	10							
	Traffic safety	6	18			3				
	Aesthetics	1	16							
	*Total favorable features*	*11*	*48*	*1*	*0*	*3*	*0*	*11*	*51*	*1*
	Compromised general safety		2							
	Criminality and crime concerns		14	1		3				
	Traffic hazards and concerns		9	3		3				
	Unfavorable aesthetics		2							
	*Total unfavorable features*	*0*	*27*	*4*		*6*		*0*	*33*	*4*
	***Desirability total***							***15***	***84***	***1***
Destination accessibility	Destination mix	3	5		1	4				
	Parks, natural features, and public open space	2	6		4	14				
	Public transport	3	1		3	7				
	Recreational facilities		2		1					
	Shops and services for daily living	4	7		2	2				
	Walking/cycling infrastructure	1								
	Friendly topography		1	1						
	*Total favorable features*	*13*	*23*	*1*	*11*	*27*	*0*	*24*	*49*	*1*
	Inaccessible public transport	2								
	Inaccessible walking/cycling infrastructure		1							
	Unfriendly topography		6			1				
	*Total unfavorable features*	*2*	*7*	*0*	*0*	*1*	*0*	*2*	*8*	*0*
	***Destination accessibility total***							***24***	***57***	***3***
Destination proximity	Proximate destination mix	2			1					
	Proximate parks, natural features, and public open space		4		3	14				
	Proximate recreational facilities		5			1				
	Proximate shops and services for daily living		2			11				
	Proximate sport and exercise facilities					1				
	Proximate school/work					4	1			
	*Total favorable features*	*2*	*11*	*0*	*4*	*31*	*1*	*6*	*42*	*1*
	Proximate unfavorable destinations					1				
	Distance to recreational facilities		2			5				
	Distance to parks, natural features, and public open space					1				
	Distance to shops and services for daily living					3	2			
	Distance to school/work			1		2				
	General travel distance or time			3						
	*Total unfavorable features*	*0*	*2*	*4*	*0*	*12*	*2*	*0*	*14*	*6*
	***Destination proximity total***							***12***	***56***	***1***
Disaster mitigation	General greenery	1	1			2				
	Trees and shade	1			1	4				
	Parks and park area					4				
	***Disaster mitigation total***	*2*	*1*	*0*	*1*	*10*	*0*	***3***	***11***	***0***
Distance to public transport			2			13		***0***	***15***	***0***
Diverse housing and land use		*2*	*3*	*0*	*2*	*19*	*0*	***4***	***22***	***0***
Multi-component category	General environment supportive of physical activity	3	4			2				
	Walkability and walking-friendly environment	4	2		14	7				
	New urbanist designed development				10	16	1			
	*Total favorable features*	*7*	*6*	*0*	*24*	*25*	*1*	*31*	*31*	*1*
	General environment compromising physical activity		1					*0*	*1*	*0*
	**Multicomponent category total**							***31***	***32***	***1***

*Note* “+” = positive relationship / supports physical activity, “0” = null relationship, “-“ = negative relationship / compromises physical activity

**Table 3:** Synthesis of built environment associations with recreational physical activity

11D-category	Sub-category	Perceived			Objective			Total		
		*+*	*0*	*-*	*+*	*0*	*-*	*+*	*0*	*-*
Demand management	Parking support		2							
	Parking restrictions		1							
	***Demand management total***	*0*	*3*	*0*	*0*	*0*	*0*	***0***	***3***	***0***
Density		*1*	*5*	*0*	*2*	*19*	*1*	***3***	***24***	***1***
Design	Connectivity	3	16	2	6	10				
	Walking/cycling infrastructure	5	27		2	8				
	Lot layout				2	2				
	*Total favorable features*	*8*	*43*	*2*	*10*	*20*	*0*	*18*	*63*	*2*
	Unfavorable connectivity features					11	1			
	Unfavorable walking/cycling infrastructure		3	1						
	*Total unfavorable features*	*0*	*3*	*1*	*0*	*11*	*1*	*0*	*14*	*2*
	***Design total***							***20***	***77***	***2***
Desirability	General safety	8	12			2				
	Crime safety	5	9	2						
	Traffic safety	5	22			3				
	Aesthetics	8	16							
	*Total favorable features*	*26*	*59*	*2*	*0*	*5*	*0*	*26*	*64*	*2*
	Compromised general safety		3							
	Criminality and crime concerns		15	1		3				
	Traffic hazards and concerns		8	4	1	1				
	Unfavorable aesthetics		1	1						
	*Total unfavorable features*	*0*	*27*	*6*	*1*	*4*	*0*	*1*	*31*	*6*
	***Desirability total***							***32***	***95***	***3***
Destination accessibility	Destination mix	7	6	1	1	3				
	Parks, natural features, and public open space	1	14		5	14	1			
	Public transport		1			8				
	Recreational facilities		2		1					
	Shops and services for daily living	2	10		2	2				
	Walking/cycling infrastructure		2							
	Friendly topography		2							
	*Total favorable features*	*10*	*37*	*1*	*9*	*28*	*1*	*19*	*65*	*2*
	Inaccessible parks, natural features, and public open space						1			
	Unfriendly topography		8			1				
	*Total unfavorable features*		*8*			*1*	*1*	*0*	*9*	*1*
	***Destination accessibility total***							***20***	***74***	***2***
Destination proximity	Proximate destination mix	1	2		1	1				
	Proximate parks, natural features, and public open space	4	8		2	22	1			
	Proximate school/work				1	4				
	Proximate recreational facilities	1	5		1	1				
	Proximate shops and services for daily living		2	1	2	9				
	*Total favorable features*	*6*	*17*	*1*	*7*	*37*	*1*	*13*	*54*	*2*
	Travel time or distance			1						
	Proximate unfavorable destination mix					1				
	Distance to recreational facilities		1		1	4				
	Distance to destination mix					1				
	Distance to parks, natural features, and public open space					2				
	Distance to shops and services for daily living					4	1			
	Distance to school/work					2				
	Distance to walking/cycling infrastructure						1			
	*Total unfavorable features*		*1*	*1*	*1*	*14*	*2*	*1*	*15*	*3*
	***Destination proximity total***							***16***	***69***	***3***
Disaster mitigation	General greenery		3			2				
	Trees and shade	1	1			5				
	Parks and park area					4				
	Unspecified		1							
	***Disaster mitigation total***	*1*	*5*			*11*		***1***	***16***	***0***
Distance to public transport			4			12		***0***	***16***	***0***
Diverse housing and land use			*5*			*20*		***0***	***25***	***0***
Multi-component category	General environment supportive of physical activity	9	2			4				
	Walkability and walking-friendly environment	2	1		4	15	1			
	New urbanist designed development				10	16				
	*Total favorable features*	11	3		*14*	*35*	*1*	*25*	*38*	*1*
	General environment compromising physical activity	*1*	*1*					*1*	*1*	*0*
	**Multicomponent category total**							***25***	***39***	***2***

*Note* “+” = positive relationship / increased physical activity, “0” = null relationship, “-“ = negative relationship / compromised physical activity

### Synthesis of built environment associations with total walking and cycling

Main effect associations between the built environment and total walking and cycling were investigated in 30 studies (see Table 4), including 26 cross-sectional [48, 55, 59, 68, 69, 76, 80, 82, 83, 86, 88, 90, 93, 95–98, 103, 104, 107, 110, 112, 124–126], two longitudinal [66, 67], and two natural experiment studies [54, 60], resulting in a total of 372 associations (*n* = 319 Western countries). The most frequent favorable associations occurred for the multi-component category (*n* = 17 positive, *n* = 19 null associations), followed by desirability features (*n* = 16 positive, *n* = 39 null associations), especially for aesthetics and crime safety being relevant. Also, design features (*n* = 8 positive, *n* = 48 null associations) supported total walking and cycling. Disaster mitigation, distance to public transport, and diverse housing and land use were the least investigated and showed predominantly null associations. No study investigated demand management. When associations were investigated in both Western and non-Western locations, associations were mostly similar across study regions. Desirability (especially crime safety and aesthetics) seemed to be more important in non-Western locations (*n* = 10 positive, *n* = 9 null associations) compared to Western locations (*n* = 6 positive, *n* = 30 null associations) (see Additional File 5).

**Table 4:** Synthesis of built environment associations with total walking and cycling

11D-category	Sub-category	Perceived			Objective			Total		
		*+*	*0*	*-*	*+*	*0*	*-*	*+*	*0*	*-*
Density		*3*	*0*	*0*	*1*	*15*	*0*	***4***	***15***	***0***
Design	Connectivity	3	2			11				
	Walking/cycling infrastructure	1	7		1	7				
	Lot layout				3	3				
	*Total favorable features*	*4*	*9*	*0*	*4*	*21*	*0*	*8*	*30*	*0*
	Unfavorable connectivity features				1	17				
	Unfavorable walking/cycling infrastructure		1							
	*Total unfavorable features*	*0*	*1*	*0*	*1*	*17*	*0*	*1*	*18*	*0*
	***Design total***							***8***	***48***	***1***
Desirability	General safety	3	4							
	Crime safety	5	4							
	Traffic safety	1	12	2						
	Aesthetics	5	5			1				
	*Total favorable features*	*14*	*25*	*2*	*0*	*1*	*0*	*14*	*26*	*2*
	Compromised general safety		1							
	Criminality and crime concerns		5	1		1				
	Traffic hazards and concerns		3	1		2				
	Unfavorable aesthetics		1							
	*Total unfavorable features*	*0*	*10*	*2*	*0*	*3*	*0*	*0*	*13*	*2*
	***Desirability total***							***16***	***39***	***2***
Destination accessibility	Destination mix	2								
	Parks, natural features, and public open space				3	27				
	Public transport					7				
	Recreational facilities	1	2			1				
	Shops and services for daily living	1	5		1	2				
	Friendly topography		2							
	*Total favorable features*	*4*	*9*		*4*	*37*		*8*	*46*	*0*
	Inaccessible shops and services for daily living		1							
	Unfriendly topography		1			1				
	*Total unfavorable features*		*2*			*1*		*0*	*3*	*0*
	***Destination accessibility total***							***8***	***49***	***0***
Destination proximity	Proximate destination mix	1	1			9				
	Proximate parks, natural features, and public open space	1		1	2	22				
	Proximate school/work					6	1			
	Proximate recreational facilities		7			1				
	Proximate shops and services for daily living		5		2	27	3			
	*Total favorable features*	*2*	*13*	*1*	*4*	*65*	*4*	*6*	*78*	*5*
	Distance to recreational facilities					6				
	Distance to parks, natural features, and public open space					2				
	Distance to shops and services for daily living			1		2				
	Distance to school/work					3				
	*Total unfavorable features*			*1*		*13*		*0*	*13*	*1*
	***Destination proximity total***							***7***	***91***	***5***
Disaster mitigation	Trees and shade	*1*			*1*	*5*				
	Parks and park area					*8*				
	***Disaster mitigation total***	*1*			*1*	*13*		***2***	***13***	***0***
Distance to public transport						14		***0***	***14***	***0***
Diverse housing and land use						*15*		***0***	***15***	***0***
Multi-component category	General environment supportive of physical activity	6	2			1				
	Walkability and walking-friendly environment	2			3	9				
	New urbanist designed development				6	6				
	*Total favorable features*	*8*	*2*		*9*	*16*		*17*	*18*	*0*
	General environment compromising physical activity		1					*0*	*1*	*0*
	**Multicomponent category**							***17***	***19***	***0***

***Note*** “+” = positive relationship / increased physical activity, “0” = null relationship, “-“ = negative relationship / compromised physical activity

### Synthesis of built environment associations with MVPA

Main effect associations between the built environment and MVPA were investigated in 29 studies (see Table 5), including 28 cross-sectional studies [50, 52, 53, 63, 64, 69, 71–75, 87, 95, 97, 98, 103–106, 109, 112–115, 121, 124] and one natural experiment study [56], resulting in a total of 250 investigated associations (*n* = 96 in high-income Western countries). The most frequent favorable associations occurred for desirability features (*n* = 18 positive, *n* = 58 null associations), specifically regarding aesthetics and crime safety. This was followed by destination accessibility (*n* = 13 positive, *n* = 38 null associations), especially regarding recreational facilities and the multi-component category (*n* = 7 positive, *n* = 8 null associations). Distance to public transport, disaster mitigation, and demand management were the least investigated and showed consistently null associations.

Investigating Western and non-Western countries study locations showed associations were mostly similar across study regions; however, for multicomponent indices, positive associations were only observed in Western locations (*n* = 7), while negative associations were predominantly observed in non-Western locations (*n* = 3). All negative associations between density and MVPA occurred in non-Western study locations (*n* = 5). Desirability features seemed to be more frequently beneficial in non-Western (*n* = 13 positive, *n* = 30 null associations) compared to Western locations (*n* = 5 positive, *n* = 28 null associations; see Additional File 5).

**Table 5:** Synthesis of built environment associations with MVPA

11D-category	Sub-category	Perceived			Objective			Total		
		*+*	*0*	*-*	*+*	*0*	*-*	*+*	*0*	*-*
Demand management	Parking restrictions		1					***0***	***1***	***0***
Density			*5*	*1*		*3*	*4*	***0***	***8***	***5***
Design	Connectivity	2	8	1	1	6	1			
	Walking/cycling infrastructure	1	14							
	*Total favorable features*	*3*	*22*	*1*	*1*	*6*	*1*	*4*	*28*	*2*
	Unfavorable walking/cycling infrastructure		5					*0*	*5*	*0*
	***Design total***							***4***	***35***	***2***
Desirability	General safety	1	11			2				
	Crime safety	3	4	1						
	Traffic safety	2	11							
	Aesthetics	5	8							
	*Total favorable features*	*11*	*34*	*1*	*0*	*2*	*0*	*11*	*36*	*1*
	Compromised general safety		5	1						
	Criminality and crime concerns		8	4						
	Traffic hazards and concerns		7	2		1				
	Unfavorable aesthetics		1							
	*Total*	*0*	*21*	*7*	*0*	*1*	*0*	*0*	*22*	*7*
	***Desirability total***							***18***	***58***	***1***
Destination accessibility	Destination mix	2								
	Parks, natural features, and public open space	3	10			4	1			
	Public transport		1			1	1			
	Recreational facilities	3	1		3	1				
	Shops and services for daily living	1	8			2	2			
	Walking/cycling infrastructure		1							
	Friendly topography		2							
	*Total favorable features*	*9*	*23*		*3*	*8*	*4*	*12*	*31*	*4*
	Inaccessible recreational facilities		1							
	Inaccessible shops and services for daily living			1						
	Inaccessible parks, natural features, and public open space					1				
	Unfriendly topography		4			1				
	*Total unfavorable features*	*0*	*5*	*1*	*0*	*2*	*0*	*0*	*7*	*1*
	***Destination accessibility total***							***13***	***38***	***4***
Destination proximity	Proximate destination mix				1	2				
	Proximate parks, natural features, and public open space	2	3	1						
	Proximate recreational facilities		5							
	Proximate shops and services for daily living		2							
	*Total favorable features*	*2*	*10*	*1*	*1*	*2*	*0*	*3*	*12*	*1*
	Distance to destination mix					1				
	Distance to parks, natural features, and public open space				1	2				
	Distance to recreational facilities					3				
	Distance to shops and services for daily living					4				
	Distance to walking/cycling infrastructure					1				
	*Total unfavorable features*	*0*	*0*	*0*	*1*	*11*	*0*	*1*	*11*	*0*
	***Destination proximity total***							***3***	***23***	***2***
Disaster mitigation	General greenery		1							
	Trees and shade		1							
	***Disaster mitigation total***		*2*					***0***	***2***	***0***
Distance to public transport			3			2		***0***	***5***	***0***
Diverse housing and land use			4		1	5	1	***1***	***9***	***1***
Multi-component category	General environment supportive of physical activity	1	1			2				
	Walkability and walking-friendly environment	1	1			3	3			
	New urbanist designed development				5	1				
	*Total favorable features*	*2*	*2*	0	*5*	*6*	*3*	*7*	*8*	*3*
	General environment compromising physical activity	1						*1*	*0*	*0*
	**Multi-component category total**							***7***	***8***	***4***

*Note* “+” = positive relationship / increased physical activity, “0” = null relationship, “-“ = negative relationship / compromised physical activity

### Synthesis from moderation and stratified analyses

A total of 27 studies [43–47, 49, 56, 57, 66, 74, 75, 83–85, 91, 92, 96, 103, 105, 106, 108, 112, 113, 117–119, 124] reported stratified or interaction analysis, resulting in *n* = 864 cases (see Additional file 6). Age and gender were the most common moderation/stratification variables. Across most physical activity and 11D-dimensions, synthesized results indicated similar associations across strata or moderator variables. However, some variation was observed. For design features and destination accessibility, some interactions were reported with other environmental variables (e.g., connectivity features, safety) across the four physical activity dimensions. For the multi-component category, results indicated that particularly people with low physical activity benefited from moving to a new urbanist-designed development. Some results also showed that recreational physical activity increased inside but decreased outside the neighborhood after relocating. Results indicated that crime safety was beneficial for men’s, but not for women’s MVPA. In contrast, aesthetics and accessibility of shops and services were beneficial for women, but not for men. Inaccessible recreational facilities were disadvantageous for retired, but not working adults. For active transport and recreational physical activity, some results indicated that particularly people with low socio-economic status benefit from diverse housing and land use and desirable aesthetics.

## Discussion

This review summarized the literature on associations between the built environment and physical activity in locations with a tropical or subtropical arid world climate. Across the different physical activity outcomes, destination accessibility, aesthetics and safety (desirability), as well as connectivity and walking/cycling infrastructure (design), were the built environment attributes most frequently associated with physical activity, particularly if multiple attributes were present at the same time. This pattern remained if we only included studies rated as high quality (see Additional File 7). Very few studies assessed built environment attributes specifically relevant for physical activity in tropical or subtropical climates or that could be relevant during extreme heat waves. Results from Western countries dominated, but were largely comparable with results from non-Western countries. Stratified and moderation analyses showed that results were largely generalizable across gender and age groups. Results from natural experiments indicated that relocating to an activity-friendly neighborhood impacted sub-groups differently. A summary of the key findings and research needs is provided in Table 6.

**Table 6:** Built environment and physical activity key findings and research needs in hot climate

Key findings and policy recommendations	Research needs
• Amongst the built environment features researched, providing access to destinations, walking and cycling infrastructure, connected streets and locations (design), and providing a safe and aesthetically pleasing environment (desirability) are the most promising single characteristics and categories to increase physical activity also in tropical or subtropical arid locations. • Environments that contain attributes across multiple 11D categories– such as walkable neighborhoods or new-urbanist developments– seem to be most promising. • Evidence on the importance of disaster mitigation features, such as shade and tree canopy, is scarce but these features can likely facilitate physical activity in (sub) tropical arid locations. • Many associations are similar across Western- and non-Western locations. However, in contrast to Western countries, density and walkability were mostly negatively associated with physical activity, while aesthetics and safety seem to be more important in non-Western locations. • Introducing or adapting policies tailored to the 11D’s is a powerful tool to support the implementation of physical activity-enhancing built environment characteristics. Policies are most promising to support the creation and maintenance of physical activity-enhancing environments if they are integrated across relevant sectors and governance levels, provide specific and measurable targets for monitoring, and are informed by evidence.	• The importance of tree shade and greenery to facilitate physical activity in a tropical or subtropical arid climate needs to be studied, in particular in longitudinal or quasi-experimental studies. • The large majority of cities in tropical or subtropical arid regions are non-Western. Evaluating the association between physical activity and environmental characteristics, cultural values, and urban forms in this context is needed. • Future multi-national and multi-cultural studies would be valuable to investigate differences and underlying reasons regarding environment–physical activity associations

Comparing our reviews’ results to previous systematic reviews without location restrictions, many results regarding the supportive character of the environment are similar, including destination accessibility, walking/cycling infrastructure and street connectivity, and aesthetics [15, 17, 18]. Previous reviews mostly concluded that density was supportive of physical activity [15, 17, 18], which in our review was only applicable to Western countries. Regarding an aesthetically pleasing environment, there has been variability in previous reviews, with some reviews concluding that aesthetics are supportive of physical activity [15, 18], and others not [17], while this also seemed to be important in our review. This was similar for crime safety, with some reviews not supporting its importance for physical activity [15, 17], while others [18] and our review considered personal safety as supportive for physical activity, especially in non-Western countries.

Destination accessibility had frequent positive associations with physical activity both in Western and non-Western countries. Specifically, accessible parks and open space were important across physical activity categories, while there were also more specific destinations for each physical activity category, including accessible public transport and shops and services for daily living for active transport, or recreational facilities for MVPA. Hence, to support physical activity as part of an active lifestyle in different domains, destinations for daily living and transport should be easily accessible. To facilitate this, a practical approach could be the “15-Minute City” concept building on density, diverse housing and land use, and digitalization prioritizing short active commutes in city planning, so that infrastructure for recreational, cultural, and social needs are accessible within a short distance [127]. Many cities worldwide have started using the concept, both when adapting existing neighborhoods and when planning new ones. For example, Saudi Arabia is planning “The Line”, a 170 km long urban vertical city with the goal to accommodate nine million people without highways and vehicles, but focusing on walkable neighborhoods and public parks, access to destinations for daily living within five minutes, and access to high-speed public transport [128, 129]. While several associations included in this data synthesis also showed the importance of destination proximity, there were fewer positive associations than for destination accessibility. This indicates that proximity alone is insufficient, but the destinations must fit citizens’ needs. For example, in this review, proximity to parks and public open space was frequently investigated, but often showed null associations with physical activity. One reason for this may be that although a park is close to one’s home, feelings of insecurities, park criminality, or lack of maintenance and infrastructure may hinder accessibility [130]. Hence, public destinations should be designed to address different needs. For example, the “Sports Boulevard” project in Riyadh, Saudia Arabia aims to provide state-of-the-art infrastructure and amenities that support pursing a healthier lifestyle through eight distinguished districts [131]. This is closely related to desirability, another category with frequent favorable associations with physical activity, represented through safety and aesthetics. Although associations were similar across study regions for total walking and cycling as well as MVPA, favorable associations for desirability seemed to be more pronounced for the non-Western countries. A reason for this maybe that except for some countries in North Africa and the Middle East (Saudi Arabia, United Arab Emirates, Kuwait, and Israel), all other countries representing the non-Western world in this review (Mexico, Ghana, Pakistan, Iran, Nigeria, India, and Ethiopia) are low-to-middle income countries, where perceived safety and crime might be more important [132]. Crime safety seemed to be beneficial for men’s, but not women’s MVPA. A reason for this could be that the studies showing those associations were conducted in Nigeria [103, 105], where sociocultural and traditional norms may prevent women from physical activity engagement independent of built environment characteristics [133].

Design features were often favorably associated with physical activity, especially concerning connectivity as well as walking and cycling infrastructure, supporting findings from another review for active transport and recreational physical activity [14]. Although associations seem to be more frequently favorable in Western countries, especially for active transport and recreational physical activity, comparisons with non-Western countries are difficult due to the limited number of associations there.

Density was mostly assessed as residential density and objectively, showing some positive associations with physical activity; however, those positive associations were almost exclusively observed in Western countries, while the negative associations were exclusively observed in non-Western countries. This could be because the population size and density in non-Western study locations differed from the Western ones. For example, higher residential density was related to less physical activity in Karachi, Pakistan, a compact city with approximately 15 million citizens, whereas in mainly sub-urban Perth, Australia, home to around 2 million people, higher residential density related positively to physical activity [134]. Hence, the size and density in Karachi may exceed optimal threshold values to foster physical activity [135]. Disaster mitigation, distance to public transport, and demand management were predominantly unrelated to any physical activity. Diverse housing and land use showed some positive associations with active transport but was otherwise largely unrelated.

While these categories may not have been related to physical activity as single-environment variables, this does not necessarily mean that they do not have the potential to facilitate physical activity, but rather that they may need to be implemented in combination with other environmental measures [32, 33]. Indeed, environmental indices or investigations comprising multiple features across 11D categories had the highest share of positive associations, ranging from 37% for MVPA up to 48% for active transportation. This is in line with previous results from the international and multi-cultural IPEN study, which demonstrated that multivariable indices of the built environment showed the strongest associations with physical activity, arguing that single variables may underestimate associations [136]. Coming back to the results of this review, while density as well as diverse housing and land use were predominantly unrelated to physical activity, walkability indices, classically consisting of density, design, and diverse housing and land use [35], were often related to enhanced physical activity, especially for active transport. “Livable neighborhoods” is another multi-component concept, which was studied in the RESIDE project in Perth. This concept refers to new urbanist-designed developments, characterized through a mixture of residential areas with different independent retail and commercial services, interconnected networks, dispersed traffic, as well as affordable housing and job containment [137]. The developments were created based upon community design, movement network, lot layout, and public parkland policies [83]. While only few single parameters of each single policy and environment domain showed associations with physical activity [83], higher compliance with each of the overall policy domains was consistently associated with more walking, albeit compliance with different policies was relevant for different physical activity types [82]. This highlights the need to combine and employ environmental strategies across the 11D’s to enhance physical activity. However, in our review, such concepts were predominantly implemented and evaluated in Western countries, with the impact of similar concepts yet to be explored in non-Western countries.

Several associations were reported in both Western and non-Western study locations. Still, due to the large dominance of associations found in Western countries, the generalizability of results across study regions and cultural contexts was unclear. However, previous international studies support the generalizability of built environment– physical activity associations across international contexts. For example, the IPEN study, including up to 17 cities in twelve middle- and high-income countries across culturally different countries (e.g., Brazil, China, Denmark, and Australia), showed that associations between built environment attributes and physical activity were largely generalizable across cities [138, 139]. Nonetheless, evaluating non-Western environmental projects, such as the “Sports Boulevard” or “The Line” in Saudi Arabia, provides valuable opportunities to enhance our understanding of the impact of environmental changes in non-Western contexts with different cultural values and norms. In addition, due to the relatively low number of associations reported for non-Western compared to Western countries, we could not further distinguish between non-Western study regions. Future studies should concentrate on countries across the non-Western world in different regions (e.g., Middle East, Latin America, Southeast Asia) to account for the different environmental designs within non-Western cultures.

Practically speaking, when considering implementing urban planning and environmental changes to facilitate physical activity in tropical or subtropical locations, solutions targeting multiple 11D-components are likely to have the most potential, while desirability, destination accessibility, and design are promising singular categories. To facilitate the implementation of the 11D-components, policies are powerful tools [31, 32]. Several relevant policies tailored to support the implementation of the 11D’s have been suggested based upon the best available evidence, e.g., specifying requirements for employment and infrastructure being accessible by public transport (destination accessibility), for public open space, street connectivity, walking and cycling infrastructure, and defining targets for walking and cycling participation (design), as well as urban design codes incorporating design principles for crime prevention and traffic reduction (desirability) [32, 140]. These policies should be informed by evidence, integrated across government levels and relevant sectors (e.g., transport, housing, parks), and formulated with specific and measurable targets to monitor policy implementation [140]. For a comprehensive overview of evidence-based policies to enhance physical activity, *the Lancet Urban Design, Transport, and Health Series* provides a useful framework with specific policy recommendations [31, 32, 140].

### Limitations

This review does not come without limitations. On the study level, perceived and built environment measures as well as physical activity outcomes were assessed using various tools and neighborhood definitions, making comparisons difficult and preventing a meta-analysis. For future studies, assessing spatial indicators with open-source data is a valuable and reliable opportunity to obtain comparable results across study sites and facilitate data harmonization [141]. Physical activity was predominantly assessed via self-report, which is prone to bias. Objective built environment measures were exclusively assessed using pre-defined buffer sizes (e.g., 1600 m street-network distance around the residential home) to predominantly evaluate the neighborhood space, without knowing if the defined spatial area represents the relevant geographic context [142, 143]. To overcome this problem, ambulatory assessment methods combining accelerometers for physical activity assessment with global positioning systems (GPS) provide a valuable opportunity [144, 145], facilitating the assessment of one’s actual activity space together with device-based assessment of domain- and location-specific physical activity. However, especially in locations with hot climate, this should still be combined with the option to self-report physical activities since device-based assessment is limited in capturing water-based activities, which may be particularly relevant in such locations. Finally, as already discussed, the focus on Western (high-income) countries limits generalizability to non-Western locations.

On the review level, we solely focused on peer-reviewed publications without searching for grey literature, which may have introduced publication bias. Our search strategy has neither been created nor been peer-reviewed by a librarian. Still, experienced research team members created and refined the search strategy based upon previously published systematic review and all authors’ input. Our search strategy was based upon a combination of countries and cities in relevant world climate regions. It may not have shown the results of studies conducted in smaller cities or rural areas. However, cities are and will be even more crucial locations for physical activity and climate change, considering that cities are expected to house 60% of the world’s population by 2030 [134], tropical cities are growing at a faster rate than non-tropical cities [10], and extreme heat events due to climate change have also intensified in non-tropical cities [11]. Furthermore, as we used a machine learning approach, we did not screen all search results, but stopped after the stopping criterion was reached. However, we applied a very conservative stopping criterion, that exceeded the recommended percentage of studies screened to obtain 95% of eligible articles [27]. Due to available resources, one reviewer extracted the data, a second reviewer extracted only data for 10% of the articles to ensure data quality.

## Conclusions

Built environment attributes such as destination accessibility, connectivity, walking and cycling infrastructure, safety, and aesthetics are positively associated with physical activity, also in tropical or subtropical climates. Few studies focused on built environment attributes that are specifically relevant in a hot climate, such as shade or the availability of indoor recreation options. There is limited evidence from non-Western countries, where most of the urban population lives in subtropical or tropical climates. Built environment characteristics should be further explored in subtropical or tropical non-Western locations to enhance our understanding regarding the built environment and physical activity, considering different urban forms as well as cultural values and norms embedded into environmental designs. Based on these findings, we recommend that urban planning and policies improve existing less activity-friendly environments, facilitate the creation of activity-friendly and health-enhancing new developments, and contribute to sustainable and climate-resilient cities [146–148].

### Electronic supplementary material

Below is the link to the electronic supplementary material.

Additional File 1: Detailed methods.

Additional File 2: Search strategy.

Additional File 3: Data extraction table.

Additional File 4: Quality assessment.

Additional File 5: Associations by study region.

Additional File 6: Data synthesis for studies with stratified and moderation analysis.

Additional File 7: Associations for main effects only including studies rated as high quality.

Additional File 8:

## Data Availability

Not applicable.

## Abbreviations

WHO: World Health Organization
MVPA: moderate-to-vigorous physical activity
IPEN: International Physical Activity and Environment Network
RESIDE: Residential Environment Project
