# Supplementary material for: A systematic review on the associations between the built environment and adult’s physical activity in global tropical and subtropical climate regions
Source: Int J Behav Nutr Phys Act. 2024 May 21;21:59. doi: 10.1186/s12966-024-01582-x (PMC11107026; doi:10.1186/s12966-024-01582-x)
Supplement: Supplementary file 1 — Additional File 1: Detailed methods. [file 12966_2024_1582_MOESM1_ESM.docx]

**Additional File 4: Quality assessment**

| **#** | **Authors N** | **Study design [weight: cross-sectional = 0; longitudinal = 1; quasi-experimental and natural experiments= 2]** | **Stratification of recruitment sites by relevant environmental attributes [weight 1]** | **Adequate response rate (>60%) or shown to be representative of the population [weight 1]** | **Exposure measures shown to be valid or frequently used [weight 1]** | **Outcome measures shown to be valid [weight 1]** | **Adjustment for socio-demographic covariates (at least age, gender, and education considered) [weight 1]**  **[if one has not been considered, deduct 1/3)** | **Adjustment for self-selection [weight 1]** | **Appropriate analytical approach – accounting for clustering (if needed) [weight 1/3]** | **Appropriate analytical approach – accounting for distributional assumptions [weight 1/3]** | **Appropriate analytical approach –analyses conducted and presented correctly (e.g., formal testing of moderators; presentation of point estimates and *p*-values, 95% CIs) [weight 1/3]** | **Did not (inappropriately) categorise continuous environmental exposure [weight 1]** | **Total quality score** | **% of total points** |  |
| --- | --- | --- | --- | --- | --- | --- | --- | --- | --- | --- | --- | --- | --- | --- | --- |
| 1 | Acheampong & Siiba, 2018 | 0 | 0 | 0 | 0 | 0 | 1 | 0 | 0 | 0.33 | 0.33 | 1 | 2.66 | 26.60 |  |
| 2 | Adeniyi & Chedi 2010 | 0 | 0 | 0 | 0 | 1 | 1 | 0 | NA | 0.33 | 0 | 0 | 2.33 | 25.89 |  |
| 3 | Aliyas, 2018 | 0 | 1 | 1 | 0.5 | 1 | 0.33 | 0 | 0 | 0.33 | 0.33 | 1 | 5.49 | 54.90 |  |
| 4 | Aliyas 2020 | 0 | 1 | 1 | 0.5 | 1 | 1 | 0 | 0 | 0.33 | 0.33 | 1 | 6.16 | 61.60 |  |
| 5 | AlKheder et al., 2017 | 0 | 0 | 0 | 0 | 0 | 0 | 0 | NA | 0 | 0 | 1 | 1 | 11.11 |  |
| 6 | Alqathani et al., 2021 | 0 | 0 | 0 | 0 | 1 | 1 | 0 | NA | 0.33 | 0.33 | 1 | 3.66 | 40.67 |  |
| 7 | Arasan, 1996 | 0 | 1 | 0 | 0 | 0 | 1 | 0 | NA | 0.33 | 0.33 | 1 | 3.66 | 40.67 |  |
| 8 | Awadallea et al., 2014 | 0 | 0 | 1 | 0 | 1 | 0 | 0 | NA | 0 | 0.33 | 0 | 2.33 | 25.89 |  |
| 9 | Badland et al., 2013 | 1 | 1 | 0 | 1 | 1 | 1 | 0 | 0 | 0.33 | 0.33 | 1 | 6.66 | 66.60 |  |
| 10 | Bartshe et al., 2018 | 0 | 0 | 0 | 1 | 1 | 0.66 | 0 | NA | 0.33 | 0.33 | 1 | 4.32 | 48.00 |  |
| 11 | Bartshe et al., 2021 | 0 | 0 | 0 | 1 | 1 | 0.66 | 0 | NA | 0.33 | 0.33 | 1 | 4.32 | 48.00 |  |
| 12 | Beenackers et al., 2012 | 2 | 1 | 0 | 1 | 1 | 1 | 0 | 0 | 0.33 | 0.33 | 1 | 7.66 | 76.60 |  |
| 13 | Bungum et al., 2012 | 0 | 0 | 0 | 0 | 1 | 1 | 0 | NA | 0.33 | 0.33 | 1 | 3.66 | 40.67 |  |
| 14 | Calise et al., 2012 | 2 | 0 | 1 | 0 | 1 | 0 | 0 | NA | 0.33 | 0.33 | 1 | 5.66 | 62.89 |  |
| 15 | Calise et al. 2013 | 2 | 0 | 1 | 0 | 1 | 0 | 0 | NA | 0.33 | 0.33 | 1 | 5.66 | 62.89 |  |
| 16 | Cao et al., 2005 | 0 | 1 | 0 | 1 | 0 | 0.66 | 1 | 0 | 0.33 | 0.33 | 1 | 5.32 | 53.20 |  |
| 17 | Christian et al., 2011 | 0 | 0 | 0 | 1 | 1 | 1 | 0 | NA | 0.33 | 0.33 | 1 | 4.66 | 51.78 |  |
| 18 | Christian et al., 2013 | 2 | 1 | 0 | 1 | 1 | 1 | 0 | 0.33 | 0 | 0.33 | 1 | 7.66 | 76.60 |  |
| 19 | Christian et al., 2017 | 1 | 1 | 0 | 1 | 1 | 1 | 0 | NA | 0.33 | 0.33 | 1 | 6.66 | 74.00 |  |
| 20 | Coughenor et al., 2019 | 0 | 1 | 0 | 1 | 1 | 1 | 0 | 0 | 0.33 | 0.33 | 1 | 5.66 | 56.60 |  |
| 21 | Delassera et al., 2018 | 0 | 0 | 0 | 1 | 1 | 0.66 | 0 | NA | 0.33 | 0.33 | 1 | 4.32 | 48.00 |  |
| 22 | Duncan et al., 2004 | 0 | 0 | 0 | 0.5 | 1 | 1 | 0 | NA | 0.33 | 0.33 | 1 | 4.16 | 46.22 |  |
| 23 | Foster et al., 2014a | 0 | 1 | 0 | 0.5 | 1 | 1 | 0 | 0.33 | 0.33 | 0.33 | 1 | 5.49 | 54.90 |  |
| 24 | Foster et al., 2014b | 1 | 1 | 0 | 0.5 | 1 | 1 | 0 | 0.33 | 0 | 0.33 | 1 | 6.16 | 61.60 |  |
| 25 | Foster et al., 2016 | 1 | 1 | 0 | 1 | 1 | 1 | 1 | 0.33 | 0.33 | 0.33 | 1 | 7.99 | 79.90 |  |
| 26 | García-Pérez et al., 2021 | 0 | 0 | 1 | 1 | 1 | 1 | 0 | 0.33 | 0.33 | 0.33 | 1 | 5.99 | 59.90 |  |
| 27 | Giles-Corti et al., 2002 | 0 | 0 | 0 | 1 | 0 | 1 | 0 | NA | 0.33 | 0.33 | 1 | 3.66 | 40.67 |  |
| 28 | Giles-Corti et al., 2005 | 0 | 0 | 0 | 1 | 0 | 1 | 0 | NA | 0.33 | 0.33 | 1 | 3.66 | 40.67 |  |
| 29 | Giles-Corti et al., 2013 | 2 | 1 | 0 | 1 | 1 | 1 | 0.5 | 0.33 | 0.33 | 0.33 | 1 | 8.49 | 84.90 |  |
| 30 | Gul et al., 2018a | 0 | 0 | 1 | 1 | 1 | 0 | 0 | 0 | 0.33 | 0.33 | 1 | 4.66 | 46.60 |  |
| 31 | Gul et al., 2018b | 0 | 0 | 1 | 1 | 1 | 0 | 0 | 0 | 0.33 | 0.33 | 1 | 4.66 | 46.60 |  |
| 32 | Gul et al., 2020 | 0 | 0 | 1 | 1 | 1 | 0 | 0 | 0 | 0.33 | 0.33 | 1 | 4.66 | 46.60 |  |
| 33 | Gul et al., 2021 | 0 | 0 | 1 | 1 | 1 | 0.66 | 0 | 0 | 0.33 | 0.33 | 1 | 5.32 | 53.20 |  |
| 34 | Hailemariam et al. 2020 | 0 | 0 | 1 | 0 | 1 | 0 | 0 | 0 | 0.33 | 0.33 | 0 | 2.66 | 26.60 |  |
| 35 | Handy, 1996 | 0 | 1 | 0 | 0 | 0 | 0 | 0 | 0 | 0 | 0.33 | 1 | 2.33 | 23.30 |  |
| 36 | Handy, 2001 | 0 | 1 | 0 | 0 | 0 | 0.66 | 0 | 0 | 0 | 0.33 | 1 | 2.99 | 29.90 |  |
| 37 | Heredia et al., 2022 | 0 | 0 | 0 | 1 | 1 | 1 | 0 | 0.33 | 0.33 | 0.33 | 1 | 4.99 | 49.90 |  |
| 38 | Holt et al., 2016 | 0 | 0 | 1 | 1 | 0 | 1 | 0 | 0 | 0.33 | 0 | 1 | 4.33 | 43.30 |  |
| 39 | Hooper et al., 2014 | 0 | 1 | 0 | 1 | 1 | 1 | 1 | 0.33 | 0.33 | 0.33 | 1 | 6.99 | 69.90 |  |
| 40 | Hooper et al., 2015a | 0 | 1 | 0 | 1 | 1 | 1 | 1 | 0.33 | 0.33 | 0.33 | 1 | 6.99 | 69.90 |  |
| 41 | Hooper et al., 2015b | 0 | 1 | 0 | 1 | 1 | 1 | 1 | 0.33 | 0.33 | 0.33 | 1 | 6.99 | 69.90 |  |
| 42 | Jáuregui et al., 2016 | 0 | 0 | 0 | 1 | 1 | 1 | 0 | 0.33 | 0.33 | 0.33 | 1 | 4.99 | 49.90 |  |
| 43 | Jáuregui et al., 2017 | 0 | 1 | 0 | 1 | 1 | 1 | 0 | 0.33 | 0.33 | 0.33 | 1 | 5.99 | 59.90 |  |
| 44 | Joseph et al., 2020 | 0 | 0 | 0 | 1 | 1 | 0.66 | 0 | NA | 0 | 0.33 | 1 | 3.99 | 44.33 |  |
| 45 | Joseph et al., 2021 | 0 | 0 | 0 | 1 | 1 | 1 | 0 | NA | 0.5 | 0.33 | 1 | 4.83 | 53.67 |  |
| 46 | Khalaf et al., 2013 | 0 | 0 | 1 | 0 | 1 | 0.5 | 0 | NA | 0.33 | 0.33 | 1 | 4.16 | 46.22 |  |
| 47 | Knuiman et al., 2014 | 1 | 1 | 0 | 1 | 1 | 1 | 0 | 0.33 | 0 | 0.33 | 1 | 6.66 | 66.60 |  |
| 48 | Learnihan et al., 2011 | 0 | 0 | 0 | 1 | 1 | 1 | 0 | NA | 0.33 | 0.33 | 1 | 4.66 | 51.78 |  |
| 49 | Manoj et al., 2015 | 0 | 0 | 1 | 0 | 0 | 0.66 | 0 | NA | 0.33 | 0 | 1 | 2.99 | 33.22 |  |
| 50 | Manoj et al., 2016 | 0 | 0 | 1 | 0.5 | 0 | 1 | 0 | NA | 0.33 | 0 | 1 | 3.83 | 42.56 |  |
| 51 | McCormack et al., 2012 | 0 | 0 | 0 | 1 | 1 | 1 | 1 | NA | 0.33 | 0.33 | 1 | 5.66 | 62.89 |  |
| 52 | Mehriar et al., 2021 | 0 | 1 | 0 | 1 | 0 | 0 | 0 | 0 | 0.33 | 0 | 0 | 2.33 | 23.30 |  |
| 53 | Mohamed et al., 2020 | 0 | 0 | 1 | 0 | 1 | 0.5 | 0 | NA | 0.33 | 0.33 | 1 | 4.16 | 46.22 |  |
| 54 | Nathan et al., 2012 | 0 | 0 | 1 | 1 | 0 | 1 | 0 | NA | 0.33 | 0.33 | 1 | 4.66 | 51.78 |  |
| 55 | Nathan et al., 2014a | 0 | 1 | 0 | 0 | 1 | 1 | 0 | 0.33 | 0.33 | 0.33 | 1 | 4.99 | 49.90 |  |
| 56 | Nathan et al., 2014b | 0 | 1 | 0 | 1 | 1 | 1 | 1 | 0.33 | 0.33 | 0.33 | 1 | 6.99 | 69.90 |  |
| 57 | Nathan et al., 2014c | 0 | 1 | 0 | 1 | 1 | 1 | 1 | 0.33 | 0.33 | 0.33 | 1 | 6.99 | 69.90 |  |
| 58 | Obaid et al. 2020 | 0 | 0 | 0 | 0 | 0 | 1 | 0 | NA | 0.33 | 0.33 | 1 | 2.66 | 29.56 |  |
| 59 | Olaru & Curtis, 2015 | 2 | 1 | 0 | 1 | 0 | 0 | 0 | 0.33 | 0 | 0 | 1 | 5.33 | 53.30 |  |
| 60 | Oloje et al., 2017 | 0 | 0 | 1 | 0 | 0 | 0 | 0 | 0 | 0 | 0 | 1 | 2 | 20.00 |  |
| 61 | Oyeyemi et al., 2012 | 0 | 1 | 0 | 1 | 1 | 1 | 0 | 0 | 0.33 | 0.33 | 1 | 5.66 | 56.60 |  |
| 62 | Oyeyemi et al., 2013 | 0 | 1 | 1 | 1 | 1 | 1 | 0 | 0 | 0 | 0.33 | 1 | 6.33 | 63.30 |  |
| 63 | Oyeyemi et al., 2015 | 0 | 0 | 1 | 1 | 1 | 1 | 0 | 0 | 0.33 | 0.33 | 1 | 5.66 | 56.60 |  |
| 64 | Oyeyemi et al., 2018 | 0 | 1 | 1 | 1 | 1 | 1 | 0 | 0.33 | 0.33 | 0.33 | 1 | 6.99 | 69.90 |  |
| 65 | Pimenta et al., 2020 | 0 | 0 | 0 | 0.5 | 1 | 0 | 0 | 0.33 | 0.33 | 0 | 1 | 3.16 | 31.60 |  |
| 66 | Rahul et al., 2017 | 0 | 0 | 1 | 0 | 0 | 0.66 | 0 | 0 | 0.33 | 0 | 1 | 2.99 | 29.90 |  |
| 67 | Rai et al., 2018 | 0 | 0 | 0 | 1 | 1 | 0 | 0 | NA | 0 | 0.33 | 1 | 3.33 | 37.00 |  |
| 68 | Ross et al., 2018 | 0 | 0 | 0 | 1 | 1 | 1 | 0 | NA | 0.33 | 0.33 | 1 | 4.66 | 51.78 |  |
| 69 | Ross et al., 2021 | 0 | 0 | 1 | 1 | 1 | 0 | 0 | NA | 0.33 | 0.33 | 1 | 4.66 | 51.78 |  |
| 70 | Salinas et al., 2018 | 0 | 0 | 0 | 1 | 1 | 1 | 0 | NA | 0.33 | 0.33 | 1 | 4.66 | 51.78 |  |
| 71 | Salvo et al., 2014 | 0 | 1 | 0 | 1 | 1 | 1 | 0 | 0.33 | 0.33 | 0.33 | 1 | 5.99 | 59.90 |  |
| 72 | Salvo et al., 2017 | 0 | 1 | 0 | 0 | 1 | 1 | 0 | 0.33 | 0.33 | 0.33 | 1 | 4.99 | 49.90 |  |
| 73 | Shuval et al., 2009 | 0 | 0 | 1 | 0 | 1 | 0.66 | 0 | NA | 0.33 | 0.33 | 1 | 4.32 | 48.00 |  |
| 74 | Sugiyama et al., 2010 | 0 | 1 | 0 | 1 | 1 | 1 | 0 | 0.33 | 0.33 | 0.33 | 1 | 5.99 | 59.90 |  |
| 75 | Sugiyama et al., 2014 | 0 | 1 | 0 | 1 | 1 | 1 | 0 | 0.33 | 0.33 | 0.33 | 1 | 5.99 | 59.90 |  |
| 76 | Sugiyama et al., 2015 | 0 | 1 | 0 | 1 | 0 | 1 | 0 | 0.33 | 0.33 | 0.33 | 1 | 4.99 | 49.90 |  |
| 77 | Titze et al., 2010 | 0 | 0 | 0 | 1 | 1 | 1 | 0 | NA | 0.33 | 0.33 | 1 | 4.66 | 51.78 |  |
| 78 | Vest et al., 2005 | 0 | 0 | 0 | 1 | 1 | 1 | 0 | NA | 0.33 | 0.33 | 1 | 4.66 | 51.78 |  |
| 79 | Zahra et al., 2022 | 0 | 0 | 0 | 1 | 1 | 0.66 | 0 | NA | 0.33 | 0.33 | 1 | 4.32 | 48.00 |  |
| 80 | Zhong et al., 2020 | 0 | 0 | 0 | 1 | 1 | 1 | 1 | NA | 0.33 | 0.33 | 1 | 5.66 | 62.89 |  |
| 81 | Zhu et al., 2014 | 0 | 0 | 0 | 1 | 1 | 0 | 0 | NA | 0.33 | 0.33 | 1 | 3.66 | 40.67 |  |
| 82 | Zhu et al., 2019 | 0 | 0 | 0 | 0.5 | 1 | 1 | 0 | NA | 0 | 0.33 | 1 | 3.83 | 42.56 |  |
| 83 | Zuniga-Teran et al., 2017a | 0 | 1 | 0 | 1 | 1 | 0 | 0 | NA | 0 | 0.33 | 1 | 4.33 | 48.11 |  |
| 84 | Zuniga-Teran et al., 2017b | 0 | 1 | 0 | 1 | 1 | 0 | 0 | 0 | 0 | 0.33 | 1 | 4.33 | 43.30 |  |
