# Supplementary material for: A systematic review on the associations between the built environment and adult’s physical activity in global tropical and subtropical climate regions
Source: Int J Behav Nutr Phys Act. 2024 May 21;21:59. doi: 10.1186/s12966-024-01582-x (PMC11107026; doi:10.1186/s12966-024-01582-x)
Supplement: Supplementary file 3 — Additional File 3: Data extraction table. [file 12966_2024_1582_MOESM3_ESM.docx]

**Additional File 1: Detailed methods**

**Search strategy and information sources**

A systematic search was performed in the four major databases Web of Science, Scopus, PubMed, and SportDISCUS (via EBSCOhost) from inception until 16 November 2022 based upon terms for physical activity, the built environment, and relevant study locations in relevant world climate regions (see *Additional File 2*). The World Climate Regions were determined based upon temperatures and aridity, following the International Panel on Climate Change’s (IPCC) definitions (1). For this review, we were interested in study locations with a tropical or sub-tropical climate (24-34 °C and 18-24°C mean annual temperature, respectively) with an arid or hyper-arid moisture index <0.65. We intersected countries (2) and cities (3) with the World Climate Regions (1) in ArcGIS Pro and included countries that were largely intersecting with a relevant climate zone and cities in countries where only small parts intersected with a relevant climate zone (e.g., USA) in the search term upon visual inspection. Articles meeting the eligibility criteria were used for backwards- and forwards citation search to identify additional eligible articles.

**Eligibility Criteria**

Article eligibility criteria were: 1) (Older) adults without physical disabilities in a dry or desert tropical or sub-tropical climate (1; see also *Additional File 2*) as study population; 2) quantitative; 3) peer-reviewed; 4) English, German, Dutch, Danish, or Arabian article language based upon the authors’ language skills; 5) study assessed the relationship between any perceived or objective built environment characteristics and any form of self-reported or device-assessed individual-level physical activity behavior. All study designs were eligible. Studies with adults with physical disabilities (e.g., being in a wheelchair) were excluded since their built environment needs may be different (4). The built environment was conceptualized as the physical form of communities, including land use patterns, built and natural features, and the transportation system (25). Studies only assessing aggregate environment characteristics without any further specification (e.g., urban-rural classification) or examining detailed location designs (e.g., workplace, indoor leisure facilities) were excluded.

**Selection, data collection process, and data extraction**

Records obtained from the database search were imported into EndNote X9 (version 3.3) and de-duplicated (5), before being uploaded into ASReview, an open-source machine learning tool which arranges the records based on their relevance (6). We provided twelve relevant and 13 irrelevant articles as information to ASReview, which were used to train the model using the default classifier (Naïve Bayes) and feature extraction (TF-IDF), which have fast and excellent performance (6). Next, two reviewers screened independently title and abstract in ASReview. Notably, ASReview uses an active learning system, so that the model is constantly re-trained throughout the review process based upon labeling decisions. Following modeling studies (6) and previous practices in health sciences (7), we screened 35% of all titles and abstract in the presented order, until 60 studies in a row were labeled as irrelevant. Any record labeled relevant by at least one reviewer was included for full-text screening.

To ensure that no relevant records were missed, we followed ASReview lab recommendations (8) and uploaded the dataset with all references again, using the labeled records from the first round label as prior knowledge to train the model again, now using the sophisticated classifier “Fully connected neural network (2 hidden layers)” in combination with the Doc2Vec feature extraction (6). Screening was stopped when 60 articles in a row were labeled irrelevant.

Full-texts were screened independently by two investigators. Any disagreements were discussed and if no agreement could be reached, a third investigator was consulted. The included full-texts were then used to identify additional eligible articles via backwards- and forwards-citation screening using the Shiny app *Citationchaser* (9). We merged the obtained results with the results obtained from the database search, de-duplicated the results, added the already included articles as prior knowledge, and uploaded it again into ASReview for title and abstract screening. Screening was stopped when 60 articles in a row were labeled as irrelevant.

Data extraction was conducted independently by two investigators for 10% of the included articles and continued by one investigator after an 80% agreement rate was reached (10). The following information was extracted for each article: 1) Study name, author, publication year; 2) participant and location information, 3) study design and sampling strategy, 4) environmental exposure and measures, 5) physical activity type and measures, 6) statistical analysis, and 7) study results (see *Additional File 3*). To avoid reporting bias, built environment variables not considered for the final analysis due to low significance were included in the data extraction table as null association.

**Quality assessment**

Study quality was assessed using ten criteria that were previously developed and used in similar reviews (11, 12). The criteria included: a) study design; b) study areas or participant recruitment stratified by key environmental attributes; c) participant response rate ≥60% or evidence of a representative sample; d) outcome measurements reliable and valid; e) adjustment for socio-demographic covariates; f) adjustment for self-selection; suitable analytical approach, including g) adjustment for clustering; h) approach for distributional assumptions; i) correct implementation and presentation of results; j) no inappropriate categorization of continuous environmental exposures. We the criterion k) validity of the environmental exposure measure. A score of 0, 1, and 2 was assigned for cross-sectional, longitudinal, and quasi-experimental / natural experiment study design, respectively. Criteria regarding the suitable analytical approach (g-i) were scored 1/3 if answered “yes” and 0 if answered “no”. For all other items, a score of 1 was assigned if answered “yes” and 0 if answered “no” (see *Additional File 4*). To establish quality categories, we applied the previous categorization of Barnett and colleagues (11), but transformed the cut-off points (≤ 3.5 points; 3.6- into percentages for some studies, the criterion “adjustment for clustering” did not apply, resulting in a total score of only nine instead of ten points.. Based upon these percentages, articles were categorized into low (<38.9% of total points), medium (39.0-65.6%), and high (>65.6%) quality.

**Data synthesis**

All data were entered into SPSS (version 29) for further processing, and each studied association was recorded as one case. For example, if a study investigated the association between aesthetics and walking and between aesthetics and MVPA, this would be recorded as two cases. Next, environmental attributes were assigned to one of the “11D” categories, which describe urban transport planning and design features that are hypothesized to enhance physical activity (30, 31) (see *Table 1* in the main manuscript). For categories containing diverse environmental features, we further specified environmental sub-categories based on the 11D’s description (30, 31). The 11D’s are understood as a framework of integrated interventions to create healthy and sustainable cities, which means that some features could be assigned to multiple categories (31, 33). In this work, we looked at the operationalization of the variables and assigned them to the category that best fit. For example, in the long version of the Neighborhood Environment Walkability Scale (NEWS), a scale frequently used in articles included in our review, land use mix is included as a sub-scale twice – (a) one sub-scale representing time to walk to different destinations in the neighborhood, such as restaurants, parks, public transport, or work/school, and (b) one subscale representing accessibility of destinations through a combination of items asking about access to shopping at local stores, public transit, and topography (34). Due to their different operationalizations, land use mix sub-scale (a) was assigned to “diverse housing and land use”, and sub-scale (b) to “destination accessibility”. Public transport is another example: If an item asked merely about distance to public transport (e.g., distance to the next bus stop), this was assigned to “Distance to public transport”. If the item or scale was more about the quality of public transport (e.g., the number of bus routes through the neighborhood), public transport was assigned to “destination accessibility”. The same applied to “walking and cycling infrastructure”: If the item asked about availability of walking infrastructure, this was assigned to “design”; if the item was more about the quality of the infrastructure (e.g., benches along sidewalks), this was assigned to “destination accessibility”. We further added the category “multicomponent” for environmental scores consisting of multiple 11D categories impossible to separate into single 11-D categories, such as the walkability index (32).

Physical activity outcomes were assigned to the categories of recreational physical activity, transport physical activity, total walking and cycling, and MVPA, with the two former ones representing important physical activity domains (35). We also considered sub-categories for recreational and transport physical activity, however, across all domains, walking was the outcome that received most attention (see *Table A2*), hence, we did not split the categories anymore. We determined study region (36) and World Bank income information (37) for each study location.

For the synthesis, we split the cases into main effects (analysis for the whole sample) and cases with moderation or stratified analysis (e.g., stratified analysis by gender). For the main effects, positive, null, and negative associations were specified for each environment-outcome association based on the reported statistical results and synthesized for each 11D and physical activity categories. Results were usually extracted from fully adjusted models. However, some studies first investigated bivariable associations between built environment characteristics and physical activity, and then only included variables below a specified p-value in the final analysis. Synthesis was further stratified by perceived and objective built environment measures due to representing different concepts (38, 39) and potentially different associations with physical activity (40, 41). We further synthesized the results based upon study region (17). Due to majority of cases in (high-income) Western countries (76% of all environment-physical activity associations for the main effect synthesis), we distinguished only between Western and non-Western countries (see *Additional File 5*). While we had planned to synthesize the results also based on World Bank income information, it turned out that high-income / non-high income and Western / non-Western countries were almost completely congruent, so that this would just have replicated the synthesis based upon study region.

For cases with stratified analysis (e.g., separate gender analysis), the effect for each environment-outcome association was recorded for each stratum separately. For moderation analysis, it was specified if a moderation effect was present. If so, it was noted how the moderating variable (e.g., socio-economic status) impacted the built environment-physical association. Results were again synthesized based on 11D’s and physical activity categories (see also *Additional File 6*).

*Table A2. Physical activity categories and sub-categories*

| **Physical activity category** | **Frequency** | **% within each domain** |
| --- | --- | --- |
| *Transport physical activity* |  |  |
| Transport walking | 513 | 68.9% |
| Transport cycling | 108 | 14.5% |
| Active transport | 124 | 16.6% |
| *Recreational physical activity* |  |  |
| Recreational walking | 616 | 74.2% |
| Recreational cycling | 105 | 12.7% |
| Recreational physical activity | 109 | 13.1% |
| *Total walking and cycling* |  |  |
| Total cycling | 16 | 3.0% |
| Total walking | 435 | 81.8% |
| Exercise | 23 | 4.3% |
| Unspecified physical activity | 58 | 10.9% |
| *MVPA* |  |  |
| Meeting PA guidelines | 144 | 32.8% |
| MVPA | 295 | 67.2% |

For the synthesis, we split the cases into main effects (analysis for the whole sample) and cases with moderation or stratified analysis (e.g., stratified analysis by gender). For the main effects, positive, null, and negative associations were specified for each environment-outcome association based on the reported statistical results and synthesized for each 11D and physical activity categories. Results were usually extracted from fully adjusted models. However, some studies first investigated bivariable associations between built environment characteristics and physical activity, and then only included variables below a specified p-value in the final analysis. Synthesis was further stratified by perceived and objective built environment measures due to representing different concepts (38, 39) and potentially different associations with physical activity (40, 41).

For cases with a stratified analysis (e.g., separate gender analysis), the effect for each environment-outcome association was recorded for each stratum separately. If a moderation analysis was applied, it was specified if a moderation effect was present (1) or not (0) and if so, what the moderation looked like. Results were synthesized based upon 11D- and physical activity categories (*Additional File 6*).

**References**

1. Sayre R, Karagulle D, Frye C, Boucher T, Wolff NH, Breyer S, et al. An assessment of the representation of ecosystems in global protected areas using new maps of World Climate Regions and World Ecosystems. Global Ecology and Conservation. 2020;21:e00860.

2. Maps EDa. World Countries (Generalized) 2022 [Available from: <https://bit.ly/3oA3a2D>.

3. Maps EDa. World Cities 2021 [Available from: <https://bit.ly/41PE263>.

4. Eisenberg Y, Vanderbom KA, Vasudevan V. Does the built environment moderate the relationship between having a disability and lower levels of physical activity? A systematic review. Prev Med. 2017;95:S75-S84.

5. Bramer WM, Giustini D, De Jonge GB, Holland L, Bekhuis T. De-duplication of database search results for systematic reviews in EndNote. Journal of the Medical Library Association : JMLA. 2016;104(3):240-3.

6. Van De Schoot R, De Bruin J, Schram R, Zahedi P, De Boer J, Weijdema F, et al. An open source machine learning framework for efficient and transparent systematic reviews. Nature Machine Intelligence. 2021;3(2):125-33.

7. Croon PM, Selder JL, Allaart CP, Bleijendaal H, Chamuleau SAJ, Hofstra L, et al. Current state of artificial intelligence-based algorithms for hospital admission prediction in patients with heart failure: a scoping review European Heart Journal - Digital Health. 2022;3(3):415-25.

8. developers AL. Screening and Projects. Create a Project. Model 2023 [Available from: <https://bit.ly/43YyMyO>.

9. Haddaway NR, Grainger MJ, Gray CT. Citationchaser: A tool for transparent and efficient forward and backward citation chasing in systematic searching. Research Synthesis Methods. 2022;13(4):533-45.

10. Shea BJ, Reeves BC, Wells G, Thuku M, Hamel C, Moran J, et al. AMSTAR 2: a critical appraisal tool for systematic reviews that include randomised or non-randomised studies of healthcare interventions, or both. BMJ. 2017:j4008.

11. Barnett DW, Barnett A, Nathan A, Van Cauwenberg J, Cerin E. Built environmental correlates of older adults’ total physical activity and walking: a systematic review and meta-analysis. ‎Int J Behav Nutr Phys Act. 2017;14(1).

12. Cerin E, Nathan A, Van Cauwenberg J, Barnett DW, Barnett A. The neighbourhood physical environment and active travel in older adults: a systematic review and meta-analysis. ‎Int J Behav Nutr Phys Act. 2017;14(1).

13. Giles-Corti B, Moudon AV, Lowe M, Cerin E, Boeing G, Frumkin H, et al. What next? Expanding our view of city planning and global health, and implementing and monitoring evidence-informed policy. The Lancet Global Health. 2022;10(6):e919-e26.

14. Giles-Corti B, Vernez-Moudon A, Reis R, Turrell G, Dannenberg AL, Badland H, et al. City planning and population health: a global challenge. The Lancet. 2016;388(10062):2912-24.

15. Frank LD, Sallis JF, Saelens BE, Leary L, Cain K, Conway TL, et al. The development of a walkability index: application to the Neighborhood Quality of Life Study. Br J Sports Med. 2010;44(13):924.

16. Bull FC, Al-Ansari SS, Biddle S, Borodulin K, Buman MP, Cardon G, et al. World Health Organization 2020 guidelines on physical activity and sedentary behaviour. Br J Sports Med. 2020;54(24):1451.

17. Guthold R, Stevens GA, Riley LM, Bull FC. Worldwide trends in insufficient physical activity from 2001 to 2016: a pooled analysis of 358 population-based surveys with 1·9 million participants. The Lancet Global Health. 2018;6(10):e1077-e86.

18. Bank TW. The World by Income and Region 2022 [Available from: <https://bit.ly/2oeieDT>.

19. Jáuregui A, Salvo D, Lamadrid-Figueroa H, Hernández B, Rivera-Dommarco JA, Pratt M. Perceived and Objective Measures of Neighborhood Environment for Physical Activity Among Mexican Adults, 2011. Prev Chronic Dis. 2016;13.

20. Desgeorges MM, Nazare J-A, Enaux C, Oppert J-M, Menai M, Charreire H, et al. Perceptions of the environment moderate the effects of objectively-measured built environment attributes on active transport. An ACTI-Cités study. Journal of Transport & Health. 2021;20:100972.

21. Nyunt MSZ, Shuvo FK, Eng JY, Yap KB, Scherer S, Hee LM, et al. Objective and subjective measures of neighborhood environment (NE): relationships with transportation physical activity among older persons. ‎Int J Behav Nutr Phys Act. 2015;12(1).

22. Hoehner CM, Brennan Ramirez LK, Elliott MB, Handy SL, Brownson RC. Perceived and objective environmental measures and physical activity among urban adults. Am J Prev Med. 2005;28(2, Supplement 2):105-16.
