# Supplementary material for: A systematic review on the associations between the built environment and adult’s physical activity in global tropical and subtropical climate regions
Source: Int J Behav Nutr Phys Act. 2024 May 21;21:59. doi: 10.1186/s12966-024-01582-x (PMC11107026; doi:10.1186/s12966-024-01582-x)
Supplement: Supplementary file 4 — Additional File 4: Quality assessment. [file 12966_2024_1582_MOESM4_ESM.docx]

**Additional File 2: Search strategy and eligible countries/cities**

**Search strategy in the different data bases**

**Web of Science**

#1

TS=(“physical activity” OR exercis* OR sport* OR walking OR cycling OR biking OR bike OR “active transport*” OR “active commut*” OR “active travel*” OR “active living” OR “active lifestyle” OR “active play” OR “outdoor play”)

#2

TS=(“built environment” OR “healthy environment” OR “physical environment” OR “community characteristic*” OR “community feature*” OR neighborhood OR neighbourhood OR “living environment” OR “urban environment” OR “urban design” OR “urban feature*” OR “environment* feature*” OR “urban feature*” OR “walkability” OR “green space” OR “greenspace” OR “public space” OR “open space” OR park* OR greenery OR facility* OR safety OR traffic OR facilit* OR infrastructure OR aesthetic*)

#3

TS=(Austin OR “San Antonio” OR Arizona OR “Las Vegas” OR Mexico OR “El Progreso” OR Zacapa OR Chiquimula OR Salama OR “La Paz” OR Comayagua OR Tegucigalpa OR Somoto OR Ocotal OR Esteli OR “Santa Marta” OR Barranquilla OR Cartagena OR Valledupar OR Maracaibo OR Riohacha OR Coro OR Trujillo OR Barquisimeto OR (Valencia AND Venezuela) OR Maracay OR Caracas OR Cucuta OR (Barcelona AND Venezuela) OR “La Asuncion” OR “Ciudad Bolivar” OR Portoviejo OR Guayaquil OR Machala OR Tumbes OR Piura OR Chiclayo OR Chimbote OR Huanuco OR Callao OR Lima OR Ica OR “Mayo Pablo Lagarenza” OR “Fortin Coronel Eugenia Garay” OR Mariscal OR “Fuerte Otimpo” OR “Pozo Colorado” OR “San Miguel de Tucuman” OR “Santiago del Estero” OR Catamarca OR “La Rioja” OR Gonaives OR “Monte Cristi” OR “Fort Liberte” OR Fort-Liberte OR Mao OR Santiago OR “San Juan” OR Neiba OR Jimani OR Azua OR Bani OR Pedernales OR “La Romana” OR Ponce OR Marrakech OR Marrakech OR Laayoune OR Algeria OR Tunisia OR Libya OR Egypt OR Mauritania OR Senegal OR Gambia OR Siguiri OR Mali OR Niger OR Chad OR Sudan OR Eritrea OR Ethiopia OR Djibouti OR Somalia OR Kenya OR Tanzania OR Moroto OR Mbarara OR Birao OR Ndele OR “Kaga Bandoro” OR Tingrela OR Bouna OR Dabakala OR Katiola OR Beoumi OR Bouake OR Sakassou OR Tanda OR Mbahiakro OR Daoukro OR Dimbokro OR Toumodi OR Yamoussoukro OR Benin OR Nigeria OR Maroua OR Garoua OR Kigali OR Kibungo OR Bubanza OR Bujumbura OR Angola OR Namibia OR Botswana OR Zambia OR Zimbabwe OR Mozambique OR Malawi OR Eswatini OR Pietersburg OR Mmabatho OR Toliara OR Cyprus OR Mersin OR Seyhan OR Israel OR “Palestinian Territory” OR Palestine OR “Ar Raqqah” OR “Al-Hasakah” OR “Dayr az Zawr” OR Iraq OR Kuwait OR “Saudi Arabia” OR Bahrain OR Khorrambad OR Ahvaz OR Bushehr OR “Bandar E Abbas” OR Yazd OR “Arab Emirates” OR “United Emirates” OR Oman Or Yemen OR Jordan OR Farah OR Zaranj OR “Lashkar Gah” OR Kandahar OR “Mehtar Lam” OR Jalabad OR Pakistan OR India OR Mandalay OR Sagaing OR Magway OR “Mae Hong Son” OR Lamphun OR Lampang OR Phrae OR Tak OR “Kamphaeng Phet” OR Phetchabun OR “Nakhon Ratchasima” OR Perth OR Fremantle OR “Alice Springs” OR Birdum OR Rockhampton)

**PubMed**

#1

“physical activity”[Title/Abstract] OR exercis*[Title/Abstract] OR sport*[Title/Abstract] OR walking[Title/Abstract] OR cycling[Title/Abstract] OR biking[Title/Abstract] OR bike[Title/Abstract] OR “active transport*”[Title/Abstract] OR “active commut*”[Title/Abstract] OR “active travel*”[Title/Abstract] OR “active living”[Title/Abstract] OR “active lifestyle” [Title/Abstract] OR “active play”[Title/Abstract] OR “outdoor play”[Title/Abstract]

#2

“built environment”[Title/Abstract] OR “healthy environment”[Title/Abstract] OR “physical environment”[Title/Abstract] OR “community characteristic*”[Title/Abstract] OR “community feature*”[Title/Abstract] OR neighborhood[Title/Abstract] OR neighbourhood[Title/Abstract] OR “living environment”[Title/Abstract] OR “urban environment”[Title/Abstract] OR “urban design”[Title/Abstract] OR “urban feature*”[Title/Abstract] OR “environment* feature*”[Title/Abstract] OR “urban feature*”[Title/Abstract] OR “walkability”[Title/Abstract] OR “green space”[Title/Abstract] OR “greenspace”[Title/Abstract] OR “public space”[Title/Abstract] OR “open space”[Title/Abstract] OR park*[Title/Abstract] OR greenery[Title/Abstract] OR greenness[Title/Abstract] OR facility*[Title/Abstract] OR safety[Title/Abstract] OR traffic[Title/Abstract] OR facilit*[Title/Abstract] OR infrastructure[Title/Abstract] OR aesthetic*[Title/Abstract]

#3

Austin[Title/Abstract] OR “San Antonio”[Title/Abstract] OR Arizona[Title/Abstract] OR “Las Vegas”[Title/Abstract] OR Mexico[Title/Abstract] OR “El Progreso”[Title/Abstract] OR Zacapa[Title/Abstract] OR Chiquimula[Title/Abstract] OR Salama[Title/Abstract] OR “La Paz”[Title/Abstract] OR Comayagua[Title/Abstract] OR Tegucigalpa[Title/Abstract] OR Somoto[Title/Abstract] OR Ocotal[Title/Abstract] OR Esteli[Title/Abstract] OR “Santa Marta”[Title/Abstract] OR Barranquilla[Title/Abstract] OR Cartagena[Title/Abstract] OR Valledupar[Title/Abstract] OR Maracaibo[Title/Abstract] OR Riohacha[Title/Abstract] OR Coro[Title/Abstract] OR Trujillo[Title/Abstract] OR Barquisimeto[Title/Abstract] OR (Valencia[Title/Abstract] AND Venezuela[Title/Abstract]) OR Maracay[Title/Abstract] OR Caracas[Title/Abstract] OR Cucuta[Title/Abstract] OR (Barcelona[Title/Abstract] AND Venezuela[Title/Abstract]) OR “La Asuncion”[Title/Abstract] OR “Ciudad Bolivar”[Title/Abstract] OR Portoviejo[Title/Abstract] OR Guayaquil[Title/Abstract] OR Machala[Title/Abstract] OR Tumbes[Title/Abstract] OR Piura[Title/Abstract] OR Chiclayo[Title/Abstract] OR Chimbote[Title/Abstract] OR Huanuco[Title/Abstract] OR Callao[Title/Abstract] OR Lima[Title/Abstract] OR Ica[Title/Abstract] OR Moquegua[Title/Abstract] OR Mariscal[Title/Abstract] OR “Santiago del Estero”[Title/Abstract] OR Catamarca[Title/Abstract] OR “La Rioja”[Title/Abstract] OR Gonaives[Title/Abstract] OR “Monte Cristi”[Title/Abstract] OR “Fort Liberte”[Title/Abstract] OR Fort-Liberte[Title/Abstract] OR Mao[Title/Abstract] OR Santiago[Title/Abstract] OR “San Juan”[Title/Abstract] OR Neiba[Title/Abstract] OR Jimani[Title/Abstract] OR Azua[Title/Abstract] OR Bani[Title/Abstract] OR Pedernales[Title/Abstract] OR La Romana[Title/Abstract] OR Ponce[Title/Abstract] OR Marrakech[Title/Abstract] OR Marrakech[Title/Abstract] OR Laayoune[Title/Abstract] OR Algeria[Title/Abstract] OR Tunisia[Title/Abstract] OR Libya[Title/Abstract] OR Egypt[Title/Abstract] OR Mauritania[Title/Abstract] OR Senegal[Title/Abstract] OR Gambia[Title/Abstract] OR Siguiri[Title/Abstract] OR Mali[Title/Abstract] OR Niger[Title/Abstract] OR Chad[Title/Abstract] OR Sudan[Title/Abstract] OR Eritrea[Title/Abstract] OR Ethiopia[Title/Abstract] OR Djibouti[Title/Abstract] OR Somalia[Title/Abstract] OR Kenya[Title/Abstract] OR Tanzania[Title/Abstract] OR Moroto[Title/Abstract] OR Mbarara[Title/Abstract] OR Ndele[Title/Abstract] OR “Kaga Bandoro”[Title/Abstract] OR Tingrela[Title/Abstract] OR Bouna[Title/Abstract] OR Katiola[Title/Abstract] OR Beoumi[Title/Abstract] OR Bouake[Title/Abstract] OR Tanda[Title/Abstract] OR Dimbokro[Title/Abstract] OR Toumodi[Title/Abstract] OR Yamoussoukro[Title/Abstract] OR Benin[Title/Abstract] OR Nigeria[Title/Abstract] OR Maroua[Title/Abstract] OR Garoua[Title/Abstract] OR Kigali[Title/Abstract] OR Kibungo[Title/Abstract] OR Bubanza[Title/Abstract] OR Bujumbura[Title/Abstract] OR Angola[Title/Abstract] OR Namibia[Title/Abstract] OR Botswana[Title/Abstract] OR Zambia[Title/Abstract] OR Botswana[Title/Abstract] OR Zimbabwe[Title/Abstract] OR Mozambique[Title/Abstract] OR Malawi[Title/Abstract] OR Eswatini[Title/Abstract] OR Pietersburg[Title/Abstract] OR Mmabatho[Title/Abstract] OR Toliara[Title/Abstract] OR Cyprus[Title/Abstract] OR Mersin[Title/Abstract] OR Seyhan[Title/Abstract] OR Israel[Title/Abstract] OR “Palestinian Territory”[Title/Abstract] OR Palestine[Title/Abstract] OR “Ar Raqqah”[Title/Abstract] OR “Al-Hasakah”[Title/Abstract] OR Iraq[Title/Abstract] OR Kuwait[Title/Abstract] OR “Saudi Arabia”[Title/Abstract] OR Bahrain[Title/Abstract] OR Ahvaz[Title/Abstract] OR Bushehr[Title/Abstract] OR “Bandar E Abbas”[Title/Abstract] OR Yazd[Title/Abstract] OR “Arab Emirates”[Title/Abstract] OR “United Emirates”[Title/Abstract] OR Oman[Title/Abstract] OR Yemen[Title/Abstract] OR Jordan[Title/Abstract] OR Farah[Title/Abstract] OR Zaranj[Title/Abstract] OR “Lashkar Gah”[Title/Abstract] OR Kandahar[Title/Abstract] OR Pakistan[Title/Abstract] OR India[Title/Abstract] OR Mandalay[Title/Abstract] OR Sagaing[Title/Abstract] OR Magway[Title/Abstract] OR “Mae Hong Son”[Title/Abstract] OR Lamphun[Title/Abstract] OR Lampang[Title/Abstract] OR Phrae[Title/Abstract] OR Tak[Title/Abstract] OR “Kamphaeng Phet”[Title/Abstract] OR Phetchabun[Title/Abstract] OR “Nakhon Ratchasima”[Title/Abstract] OR Perth[Title/Abstract] OR Fremantle[Title/Abstract] OR “Alice Springs”[Title/Abstract] OR Rockhampton[Title/Abstract]

**Scopus**

#1

TITLE-ABS-KEY("physical activity" OR exercis* OR sport* OR walking OR cycling OR biking OR bike OR "active transport*" OR "active commut*" OR "active travel*" OR "active living" OR "active lifestyle" OR "active play" OR "outdoor play")

#2

TITLE-ABS-KEY(“built environment” OR “healthy environment” OR “physical environment” OR “community characteristic*” OR “community feature*” OR neighborhood OR neighbourhood OR “living environment” OR “urban environment” OR “urban design” OR “urban feature*” OR “environment* feature*” OR “urban feature*” OR “walkability” OR “green space” OR “greenspace” OR “public space” OR “open space” OR park* OR greenery OR facility* OR safety OR traffic OR facilit* OR infrastructure OR aesthetic*)

#3

TITLE-ABS-KEY(Austin OR “San Antonio” OR Arizona OR “Las Vegas” OR Mexico OR “El Progreso” OR Zacapa OR Chiquimula OR Salama OR “La Paz” OR Comayagua OR Tegucigalpa OR Somoto OR Ocotal OR Esteli OR “Santa Marta” OR Barranquilla OR Cartagena OR Valledupar OR Maracaibo OR Riohacha OR Coro OR Trujillo OR Barquisimeto OR (Valencia AND Venezuela) OR Maracay OR Caracas OR Cucuta OR (Barcelona AND Venezuela) OR “La Asuncion” OR “Ciudad Bolivar” OR Portoviejo OR Guayaquil OR Machala OR Tumbes OR Piura OR Chiclayo OR Chimbote OR Huanuco OR Callao OR Lima OR Ica OR Moquegua OR “Mayo Pablo Lagarenza” OR “Fortin Coronel Eugenia Garay” OR Mariscal OR “Fuerte Otimpo” OR “Pozo Colorado” OR “San Miguel de Tucuman” OR “Santiago del Estero” OR Catamarca OR “La Rioja” OR Gonaives OR “Monte Cristi” OR “Fort Liberte” OR Fort-Liberte OR Mao OR Santiago OR “San Juan” OR Neiba OR Jimani OR Azua OR Bani OR Pedernales OR “La Romana” OR Ponce OR Marrakech OR Marrakech OR Laayoune OR Algeria OR Tunisia OR Libya OR Egypt OR Mauritania OR Senegal OR Gambia OR Siguiri OR Mali OR Niger OR Chad OR Sudan OR Eritrea OR Ethiopia OR Djibouti OR Somalia OR Kenya OR Tanzania OR Moroto OR Mbarara OR Birao OR Ndele OR “Kaga Bandoro” OR Tingrela OR Bouna OR Dabakala OR Katiola OR Beoumi OR Bouake OR Sakassou OR Tanda OR Mbahiakro OR Daoukro OR Dimbokro OR Toumodi OR Yamoussoukro OR Benin OR Nigeria OR Maroua OR Garoua OR Kigali OR Kibungo OR Bubanza OR Bujumbura OR Angola OR Namibia OR Botswana OR Zambia OR Botswana OR Zimbabwe OR Mozambique OR Malawi OR Eswatini OR Pietersburg OR Mmabatho OR Toliara OR Cyprus OR Mersin OR Seyhan OR Israel OR “Palestinian Territory” OR Palestine OR “Ar Raqqah” OR “Al-Hasakah” OR “Dayr az Zawr” OR Iraq OR Kuwait OR “Saudi Arabia” OR Bahrain OR Khorrambad OR Ahvaz OR Bushehr OR “Bandar E Abbas” OR Yazd OR “Arab Emirates” OR “United Emirates” OR “United Arab Emirates” OR Oman Or Yemen OR Jordan OR Farah OR Zaranj OR “Lashkar Gah” OR Kandahar OR “Mehtar Lam” OR Jalabad OR Pakistan OR India OR Mandalay OR Sagaing OR Magway OR “Mae Hong Son” OR Lamphun OR Lampang OR Phrae OR Tak OR “Kamphaeng Phet” OR Phetchabun OR “Nakhon Ratchasima” OR Perth OR Fremantle OR “Alice Springs” OR Birdum OR Rockhampton)

**SportDISCUS**

TITLE OR Abstract OR Keywords

#1

“physical activity” OR exercis* OR sport* OR walking OR cycling OR biking OR bike OR “active transport*” OR “active commut*” OR “active travel*” OR “active living” OR “active lifestyle” OR “active play” OR “outdoor play”

#2

“built environment” OR “healthy environment” OR “physical environment” OR “community characteristic*” OR “community feature*” OR neighborhood OR neighbourhood OR “living environment” OR “urban environment” OR “urban design” OR “urban feature*” OR “environment* feature*” OR “urban feature*” OR “walkability” OR “green space” OR “greenspace” OR “public space” OR “open space” OR park* OR greenery OR facility* OR safety OR traffic OR facilit* OR infrastructure OR aesthetic*

#3

Austin OR “San Antonio” OR Arizona OR “Las Vegas” OR Mexico OR “El Progreso” OR Zacapa OR Chiquimula OR Salama OR “La Paz” OR Comayagua OR Tegucigalpa OR Somoto OR Ocotal OR Esteli OR “Santa Marta” OR Barranquilla OR Cartagena OR Valledupar OR Maracaibo OR Riohacha OR Coro OR Trujillo OR Barquisimeto OR (Valencia AND Venezuela) OR Maracay OR Caracas OR Cucuta OR (Barcelona AND Venezuela) OR “La Asuncion” OR “Ciudad Bolivar” OR Portoviejo OR Guayaquil OR Machala OR Tumbes OR Piura OR Chiclayo OR Chimbote OR Huanuco OR Callao OR Lima OR Ica OR Moquegua OR “Mayo Pablo Lagarenza” OR “Fortin Coronel Eugenia Garay” OR Mariscal OR “Fuerte Otimpo” OR “Pozo Colorado” OR “San Miguel de Tucuman” OR “Santiago del Estero” OR Catamarca OR “La Rioja” OR Gonaives OR “Monte Cristi” OR “Fort Liberte” OR Mao OR Santiago OR “San Juan” OR Neiba OR Jimani OR Azua OR Bani OR Pedernales OR “La Romana” OR Ponce OR Marrakech OR Morocco OR Algeria OR Tunisia OR Libya OR Egypt OR Mauritania OR Senegal OR Gambia OR Siguiri OR Mali OR Niger OR Chad OR Sudan OR Eritrea OR Ethiopia OR Djibouti OR Somalia OR Kenya OR Tanzania OR Moroto OR Mbarara OR Birao OR Ndele OR “Kaga Bandoro” OR Tingrela OR Bouna OR Dabakala OR Katiola OR Beoumi OR Bouake OR Sakassou OR Tanda OR Mbahiakro OR Daoukro OR Dimbokro OR Toumodi OR Yamoussoukro OR Benin OR Nigeria OR Maroua OR Garoua OR Kigali OR Kibungo OR Bubanza OR Bujumbura OR Angola OR Namibia OR Botswana OR Zambia OR Botswana OR Zimbabwe OR Mozambique OR Malawi OR Eswatini OR Pietersburg OR Mmabatho OR Toliara OR Cyprus OR Mersin OR Seyhan OR Israel OR Palestinia OR “Ar Raqqah” OR “Al-Hasakah” OR “Dayr az Zwar” OR Iraq OR Kuwait OR “Saudi Arabia” OR Khorrambad OR Ahvaz OR Bushehr OR “Bandar E Abbas” OR Yazd OR “Arab Emirates” OR Oman Or Yemen OR Farah OR Zaranj OR “Lashkar Gah” OR Kandahar OR “Mehtar Lam” OR Jalabad OR Pakistan OR India OR Mandalay OR Sagaing OR Magway OR “Mae Hong Son” OR Lamphun OR Lampang OR Phrae OR Tak OR “Kamphaeng Phet” OR “Phetchabun” OR “Nakhon Ratchasima” OR Perth OR Fremantle OR “Alice Springs” OR Birdum OR Rockhampton

**Eligible countries and cities**

| Austin  “San Antonio”  Arizona  “Las Vegas”  Phoenix  Mexico  “El Progreso”  Zacapa  Chiquimula  Salama  “La Paz”  Comayagua  Tegucigalpa  Somoto  Ocotal  Esteli  “Santa Marta”  Barranquilla  Cartagena  Valledupar  Maracaibo  Riohacha  Coro  Trujillo  Barquisimeto  (Valencia AND Venezuela)  Maracay  Caracas  Cucuta  (Barcelona AND Venezuela)  “La Asuncion”  “Ciudad Bolivar”  Ptoviejo  Guayaquil  Machala  Tumbes  Piura  Chiclayo  Chimbote  Huanuco  Callao  Lima Ica  “Mayo Pablo Lagarenza”  “Ftin Conel Eugenia Garay” | Mariscal  “Fuerte Otimpo”  “Pozo Colado”  “San Miguel de Tucuman”  “Santiago del Estero”  Catamarca  “La Rioja”  Gonaives  “Monte Cristi”  “Ft Liberte”  Ft-Liberte  Mao  Santiago (only in the Dominican Republic)  “San Juan”  Neiba  Jimani  Azua  Bani  Pedernales  “La Romana”  Ponce  Marrakec  Laayoune  Algeria  Tunisia  Libya  Egypt  Mauritania  Senegal  Gambia  Siguiri  Mali  Niger  Chad  Sudan  Eritrea  Ethiopia  Djibouti  Somalia  Kenya  Tanzania  Moto | Mbarara  Birao  Ndele  “Kaga Bando”  Tingrela  Bouna  Dabakala  Katiola  Beoumi  Bouake  Sakassou  Tanda  Mbahiakro  Daoukro  Dimbokro  Toumodi  Yamoussoukro  Benin  Nigeria  Maroua  Garoua  Kigali  Kibungo  Bubanza  Bujumbura  Angola  Namibia  Botswana  Zambia  Zimbabwe  Mozambique  Malawi  Eswatini  Pietersburg  Mmabatho  Toliara  Cyprus  Mersin  Seyhan  Israel  “Palestinian Territy”  Palestine  “Ar Raqqah”  “Al-Hasakah” | “Dayr az Zawr”  Iraq  Kuwait  “Saudi Arabia”  Bahrain  Khrambad  Ahvaz  Bushehr  “Bandar E Abbas”  Yazd  “Arab Emirates”  “United Emirates”  Oman  Yemen  Jdan  Farah  Zaranj  “Lashkar Gah”  Kandahar  “Mehtar Lam”  Jalabad  Pakistan  India  Mandalay  Sagaing  Magway  “Mae Hong Son”  Lamphun  Lampang  Phrae  Tak  “Kamphaeng Phet”  Phetchabun  “Nakhon Ratchasima”  Perth  Fremantle  “Alice Springs”  Birdum  Rockhampton |
| --- | --- | --- | --- |
