# Supplementary material for: A systematic review on the associations between the built environment and adult’s physical activity in global tropical and subtropical climate regions
Source: Int J Behav Nutr Phys Act. 2024 May 21;21:59. doi: 10.1186/s12966-024-01582-x (PMC11107026; doi:10.1186/s12966-024-01582-x)
Supplement: Supplementary file 5 — Additional File 5: Associations by study region. [file 12966_2024_1582_MOESM5_ESM.docx]

**Additional File 3: Data extraction table**

| **#** | **Name & author, article reference in review**  **[Name of study, first author, publication year]** | **Participants**  **[Total sample size; urban, rural, or mixed sample; response rate or proof of representativeness of sample; study population; geographical location]** | **Study design**  **[Cross-sectional, longitudinal, or experimental; sampling method for clusters and individuals; stratification used by environment attributes; neighborhood definition]** | **Environmental exposure variables**  **[Environmental variables, their type (objective vs perceived); Validation or established measure (Yes/No)]** | **Outcome measures**  **[PA outcome measure; instrument; validity]** | **Moderators**  **[Moderators examined and breakdown of sample size by qualitative moderator (e.g., sex; educational attainment)]** | **Analytical approach  [Analytical approach; adjustment for clustering; appropriateness (distributional assumptions; moderation analyses) and presentation]** | **Covariates**  **[Covariates included in the analyses beyond exposure variable(s)]** | **Findings**  **Main effects or moderating effects (conclusion: bold and green = effect,** not bold and black = no effect**)** |
| --- | --- | --- | --- | --- | --- | --- | --- | --- | --- |
| 1 | NA  Acheampong & Siiba, 2018  [1] | N= 455 (urban)  Mean age: 30 ± 9 years  Women: 41%  Response rate: NR  Community dwellers  Tamale metropolis, Ghana | Cross-sectional  Cluster: purposive  Individuals: random  Stratification: none  Neighborhood definition: neighborhood boundaries | *Exposure variable*  1. Perceived neighborhood-scale challenges  2. Perceived metro-scale challenges  3. Perceived neighborhood-scale opportunities  4. Perceived metro-scale opportunities  *Measure*: Perceived  [15 items]  *Validation:* No | *Outcome*  Utility cycling frequently or occasionally [Yes/No]  *Measure:* Self-report  [1 item]  *Validation:* No | Metro-scale challenges * opportunities; neighborhood-scale challenges * opportunities | Logistic regression | Gender, education, marital status, bike ownership, location, personal transport ownership, commuting benefits, status symbol, influence of significant others | *Utility cycling*  1. **Perceived neighborhood-scale challenges**: OR=0.728, p<0.05  2. Perceived metro-scale challenges: OR=0.995, p>0.05  3. **Perceived neighborhood-scale opportunities**: OR=1.56, p<0.05  4. Perceived metro-scale opportunities: OR=1.10, p>0.05  5. **Perceived neighborhood-scale challenges * opportunities**: OR=0.55, p<0.05  6. Perceived metro-scale challenges * opportunities: OR=1.29, p>0.05 |
| 2 | NA  Adeniyi & Chedi, 2010  [2] | N= 532 (mixed)  Age range: 28-68 years  Women: NR  Response rate: NR  Pre-retirement and retired civil servants  Kano State, Nigeria | Cross-sectional  Cluster: none  Individuals: convenience  Stratification: none  Neighborhood definition: none | *Exposure variable*  1. Unavailability of recreational facilities  *Measure*: Perceived  [NR]  *Validation:* No | *Outcome*  Moderate physical activity  *Measure:* Self-report  []  *Validation:* | Retirement status | Logistic regression | Gender, age group, settlement type, marital status, education, income, duration of job/retirement, satisfaction with job/retirement | *Moderate physical activity – Pre-retirement*  1. Unavailability of recreational facilities: OR=0.79, p>0.05  *Moderate physical activity – Retirement*  1. **Unavailability of recreational facilities**: OR=0.42, p<0.05 |
| 3 | NA  Aliyas et al., 2018  [3] | N= 398 (urban)  Largest age group 18-30 years (45-60%)  Women: 51%  Response rate: 92%  Community dwellers  Bandar Abbas, Iran | Cross-sectional  Cluster: purposive  Individuals: random  Stratification: old, average, new neighborhood and by parks  Neighborhood definition: neighborhood boundaries (recruitment level) and 1500m network distance (participant level) | *Exposure variable - Perceived*  1. Neighborhood safety  2. Neighborhood aesthetics  3. Park facilities  4. Park attractiveness  5. Park proximity  *Measure*: Perceived  [used from previous study, but no specific information]  *Validation:* No  *Exposure variable - Objective*  6. Number of parks within 1500m street-network distance  7. Park area within 1500m street-network distance  *Measure*: Objective  [NR]  *Validation:* No | *Outcome*  Recreational walking [Yes/No]  Exercise walking [Yes/No]  *Measure:* Self-report  [Behavioral Risk Factor Surveillance System Questionnaire]  *Validation:* Yes | Gender | Binary logistic regression (separate model for each environmental variable and walking) | None | *Recreational walking - Men*  Perceived  1. Neighborhood safety: OR=0.86, p>0.05  2. Neighborhood aesthetics: OR=1.22, p>0.05  3. **Park facilities**: OR=1.44, p<0.05  4. Park attractiveness: OR=1.69, p>0.05  5. Park proximity: OR=0.87, p>0.05  Objective  6. Number of parks: OR=1.03, p>0.05  7. Park area: OR=0.83, p>0.05  *Recreational walking - Women*  Perceived  1. **Neighborhood safety**: OR=2.06, p<0.05  2. **Neighborhood aesthetics**: OR=1.46, p<0.05  3. **Park facilities**: OR=1.79, p<0.05  4. Park attractiveness: OR=1.72, p>0.05  5. **Park proximity**: OR=1.30, p<0.05  Objective  6. **Number of parks**: OR=1.30, p<0.05  7. **Park area**: OR=1.24, p<0.05  *Exercise walking - Men*  1. Neighborhood safety: OR=1.26, p>0.05  2. Neighborhood aesthetics: OR=0.93, p>0.05  3. **Park facilities**: OR=1.78, p<0.05  4. **Park attractiveness**: OR=1.67, p<0.05  5. **Park proximity**: OR=1.48, p<0.05  Objective  6. Number of parks: OR=1.02, p>0.05  7. Park area: OR=0.96, p>0.05  *Exercise walking - Women*  1. **Neighborhood safety**: OR=1.70, p<0.05  2. Neighborhood aesthetics: OR=1.27, p>0.05  3. Park facilities: OR=1.60, p>0.05  4. **Park attractiveness**: OR=1.77, p<0.05  5. **Park proximity**: OR=1.32, p<0.05  Objective  6. Number of parks: OR=1.02, p>0.05  7. Park area: OR=1.08, p>0.05 |
| 4 | NA  Aliyas et al., 2020  [4] | N= 1132 (urban)  Largest age group 18-30 years (49%)  Women: 51%  Response rate: 94%  Community dwellers  Bandar Abbas, Iran | Cross-sectional  Cluster: purposive  Individuals: random  Stratification: safety problems  Neighborhood definition: neighborhood boundaries | *Exposure variable*  1. Crime safety  2. Traffic safety  3. Physical safety  4. Social safety  5. General safety  *Measure*: Perceived  [Some scales from the Neighborhood Environment Walkability Scale (NEWS)]  *Validation:* Partially | *Outcome*  Transport walking ≥ 30 min/week [Yes/No]  Leisure walking ≥ 30 min/week [Yes/No]  *Measure:* Self-report  [International Physical Activity Questionnaire (IPAQ)]  *Validation:* Yes | Age-gender, Marital status, kids in the household, education, length of residency | Logistic regression for whole sample, binary logistic regression for stratified analysis | Gender, Age, kids in the household, residency length, education, marital status | *Recreational walking – total sample*  1. **Crime safety**: OR=2.50, p<0.05  2. **Traffic safety**: OR=1.86, p<0.05  3. **Physical safety**: OR=0.34, p<0.05  4. **Social safety**: OR=1.24, p<0.05  5. **General safety**: OR=3.91, p<0.05  *Transport walking – total sample*  1. **Crime safety**: OR=1.81, p<0.05  2. **Traffic safety**: OR=1.68, p<0.05  3. Physical safety: OR=1.09, p>0.05  4. Social safety: OR=1.12, p>0.05  5. **General safety**: OR=2.89, p<0.05  *Recreational walking – women 18-30 years*  1. **Crime safety**: OR=4.51, p<0.05  2. **Traffic safety**: OR=2.13, p<0.05  3. Physical safety: OR=0.71, p>0.05  4. **Social safety**: OR=1.74, p<0.05  5. **General safety**: OR=4.95, p<0.05  *Recreational walking – women 31-45 years*  1. **Crime safety**: OR=1.75, p<0.05  2. **Traffic safety**: OR=1.90, p<0.05  3. Physical safety: OR=1.14, p>0.05  4. **Social safety**: OR=3.63, p<0.05  5. **General safety**: OR=9.39, p<0.05  *Recreational walking – women 46-65 years*  1. **Crime safety**: OR=6.02, p<0.05  2. **Traffic safety**: OR=5.17, p<0.05  3. Physical safety: OR=5.30, p>0.05  4. **Social safety**: OR=6.00, p<0.05  5. **General safety**: OR=6.36, p<0.05  *Recreational walking – men 18-30 years*  1. **Crime safety**: OR=2.09, p<0.05  2. Traffic safety: OR=1.35, p>0.05  3. Physical safety: OR=1.31, p>0.05  4. Social safety: OR=1.18, p>0.05  5. **General safety**: OR=2.59, p<0.05  *Recreational walking –men 31-45 years*  1. **Crime safety**: OR=2.46, p<0.05  2. **Traffic safety**: OR=2.17, p<.0.05  3. Physical safety: OR=0.53, p>0.05  4. Social safety: OR=0.68, p>0.05  5. General safety: OR=1.98, p>0.05  *Recreational walking –men 46-65 years*  1. **Crime safety**: OR=5.04, p<0.05  2. Traffic safety: OR=0.46, p>0.05  3. Physical safety: OR=0.48, p>0.05  4. **Social safety**: OR=3.55, p<0.05  5. General safety: OR=2.07, p>0.05  *Recreational walking – single*  1. **Crime safety**: OR=3.40, p<0.05  2. **Traffic safety**: OR=1.72, p<0.05  3. Physical safety: OR=0.91, p>0.05  4. Social safety: OR=1.19, p>0.05  5. **General safety**: OR=3.74, p<0.05  *Recreational walking – married*  1. **Crime safety**: OR=2.26, p<0.05  2. **Traffic safety**: OR=1.88, p<0.05  3. Physical safety: OR=0.87, p>0.05  4. **Social safety**: OR=2.54, p<0.05  5. **General safety**: OR=4.41, p<0.05  *Recreational walking – no kids in the household*  1. **Crime safety**: OR=2.08, p<0.05  2. **Traffic safety**: OR=1.38, p<0.05  3. Physical safety: OR=1.13, p>0.05  4. **Social safety**: OR=2.57, p<0.05  5. **General safety**: OR=4.52, p<0.05  *Recreational walking – one or more kids in the household*  1. **Crime safety**: OR=3.20, p<0.05  2. **Traffic safety**: OR=2.11, p<0.05  3. Physical safety: OR=0.77, p>0.05  4. **Social safety**: OR=1.59, p<0.05  5. **General safety**: OR=3.64, p<0.05  *Recreational walking – lower educated*  1. **Crime safety**: OR=3.30, p<0.05  2. Traffic safety: OR=1.40, p>0.05  3. Physical safety: OR=0.91, p>0.05  4. **Social safety**: OR=1.72, p<0.05  5. **General safety**: OR=3.22, p<0.05  *Recreational walking – higher educated*  1. **Crime safety**: OR=3.11, p<0.05  2. **Traffic safety**: OR=2.10, p<0.05  3. Physical safety: OR=0.83, p>0.05  4. Social safety: OR=1.61, p<0.05  5. **General safety**: OR=4.41, p<0.05  *Recreational walking – residency length <8 years*  1. **Crime safety**: OR=2.35, p<0.05  2. **Traffic safety**: OR=1.56, p<0.05  3. Physical safety: OR=0.90, p>0.05  4. **Social safety**: OR=1.86, p<0.05  5. **General safety**: OR=3.97, p<0.05  *Recreational walking – residency length ≥8 years*  1. **Crime safety**: OR=6.11, p<0.05  2. **Traffic safety**: OR=2.24, p<0.05  3. Physical safety: OR=0.83, p>0.05  4. **Social safety**: OR=1.91, p<0.05  5. **General safety**: OR=4.68, p<0.05  *Transport walking – women 18-30 years*  1. **Crime safety**: OR=1.95, p<0.05  2. **Traffic safety**: OR=2.10, p<0.05  3. Physical safety: OR=0.73, p>0.05  4. Social safety: OR=1.18, p>0.05  5. **General safety**: OR=2.40, p<0.05  *Transport walking – women 31-45 years*  1. **Crime safety**: OR=2.56, p<0.05  2. **Traffic safety**: OR=2.31, p<0.05  3. Physical safety: OR=1.34, p>0.05  4. Social safety: OR=0.84, p>0.05  5. **General safety**: OR=2.40, p<0.05  *Transport walking – women 46-65 years*  1. **Crime safety**: OR=4.25, p<0.05  2. **Traffic safety**: OR=4.79, p<0.05  3. Physical safety: OR=1.71, p>0.05  4. **Social safety**: OR=2.12, p<0.05  5. General safety: OR=0.85, p>0.05  *Transport walking – men 18-30 years*  1. **Crime safety**: OR=2.64, p<0.05  2. Traffic safety: OR=1.62, p>0.05  3. Physical safety: OR=1.70, p>0.05  4. Social safety: OR=0.98, p>0.05  5. **General safety**: OR=3.63, p<0.05  *Transport walking –men 31-45 years*  1. **Crime safety**: OR=3.29, p<0.05  2. **Traffic safety**: OR=1.81, p<0.05  3. Physical safety: OR=0.69, p>0.05  4. Social safety: OR=1.29, p>0.05  5. **General safety**: OR=3.19, p<0.05  *Transport walking –men 46-65 years*  1. **Crime safety**: OR=3.47, p<0.05  2. Traffic safety: OR=1.03, p>0.05  3. Physical safety: OR=1.08, p>005  4. **Social safety**: OR=0.42, p<0.05  5. General safety: OR=1.13, p>0.05  *Transport walking – single*  1. **Crime safety**: OR=2.00, p<0.05  2. **Traffic safety**: OR=1.60, p<0.05  3. Physical safety: OR=0.93, p>0.05  4. Social safety: OR=0.83, p>0.05  5. **General safety**: OR=2.08, p<0.05  *Transport walking – married*  1. **Crime safety**: OR=2.88, p<0.05  2. **Traffic safety**: OR=2.03, p<0.05  3. Physical safety: OR=1.26, p>0.05  4. Social safety: OR=1.08, p>0.05  5. **General safety**: OR=3.72, p<0.05  *Transport walking – no kids in the household*  1. **Crime safety**: OR=1.81, p<0.05  2. **Traffic safety**: OR=1.88, p<0.05  3. Physical safety: OR=1.19, p>0.05  4. Social safety: OR=0.92, p>0.05  5. **General safety**: OR=2.53, p<0.05  *Transport walking – one or more kids in the household*  1. **Crime safety**: OR=3.24, p<0.05  2. **Traffic safety**: OR=1.84, p<0.05  3. Physical safety: OR=1.09, p>0.05  4. Social safety: OR=1.09, p>0.05  5. **General safety**: OR=3.33, p<0.05  *Transport walking – lower educated*  1. **Crime safety**: OR=2.99, p<0.05  2. **Traffic safety**: OR=2.07, p<0.05  3. Physical safety: OR=1.07, p>0.05  4. Social safety: OR=0.74, p>0.05  5. **General safety**: OR=2.52, p<0.05  *Transport walking – higher educated*  1. **Crime safety**: OR=2.40, p<0.05  2. **Traffic safety**: OR=1.68, p<0.05  3. Physical safety: OR=1.09, p>0.05  4. Social safety: OR=1.33, p>0.05  5. **General safety**: OR=3.33, p<0.05  *Transport walking – residency length <8 years*  1. **Crime safety**: OR=2.60, p<0.05  2. **Traffic safety**: OR=1.96, p<0.05  3. Physical safety: OR=1.25, p>0.05  4. Social safety: OR=1.12, p>0.05  5. **General safety**: OR=4.60, p<0.05  *Transport walking – residency length ≥8 years*  1. **Crime safety**: OR=2.24, p<0.05  2. **Traffic safety**: OR=1.79, p<0.05  3. Physical safety: OR=0.99, p>0.05  4. Social safety: OR=0.87, p>0.05  5. **General safety**: OR=2.07, p<0.05 |
| 5 | NA  AlKheder et al., 2022  [5] | N= 1075 (urban)  60% 18-44 years  Women: 90%  Response rate: NR  College students and staff  Kuwait City, Kuwait | Cross-sectional  Cluster: none  Individuals: convenience  Stratification: none  Neighborhood definition: none | *Exposure variable*  Traffic and crime hazard during night time  *Measure:* Perceived  [8 items]  *Validation:* No | *Outcome*  Walking frequency during night time  *Measure:* Self-report  [2 items asking about walking frequency during night- and daytime]  *Validation:* No | Season; owning a car | Group differences and Cohen’s d | None | *Mean worrying level amongst different walking levels per season*  *Winter*  1: Walking rarely: M=13.57; difference 1-3: d=0.16  2: Walking weekly: M=13.59; difference 1-2: d=00  3: Walking daily: M=12.85, difference 2-3: d=0.17  *Spring*  1: Walking rarely: M=13.44; difference 1-3: d=0.16  2: Walking weekly: M=13.78; difference 1-2: d=-0.07  3: **Walking daily**: M=12.73, difference 2-3: d=0.25  *Summer*  1: **Walking rarely:** M=13.85; difference 1-3: d=0.25  2: Walking weekly: M=13.32; difference 1-2: d=0.12  3: Walking daily: M=12.66, difference 2-3: d=0.16  *Fall*  1: **Walking rarely**: M=13.72; difference 1-3: d=0.20  2: Walking weekly: M=13.45; difference 1-2: d=0.06  3: Walking daily: M=12.76, difference 2-3: d=0.16  *Mean worrying level amongst different walking levels per season for non-car owners*  *Winter*  1: **Walking rarely**: M=15.59; difference 1-3: d=0.84  2: **Walking weekly**: M=14.04; difference 1-2: d=0.34  3: **Walking daily**: M=12.20, difference 2-3: d=0.42  *Spring*  1: **Walking rarely**: M=15.00; difference 1-3: d=0.0.61  2: Walking weekly: M=14.20; difference 1-2: d=-0.18  3: **Walking daily**: M=12.23, difference 2-3: d=0.46  *Summer*  1: **Walking rarely:** M=15.12; difference 1-3: d=0.58  2: **Walking weekly**: M=13.38; difference 1-2: d=0.30  3: **Walking daily**: M=12.42, difference 2-3: d=0.32  *Fall*  1: **Walking rarely**: M=15.33; difference 1-3: d=0.75  2: **Walking weekly**: M=13.97; difference 1-2: d=0.32  3: **Walking daily**: M=12.00, difference 2-3: d=0.45 |
| 6 | NA  Alqahtani et al., 2021  [6] | N= 1859 (mixed)  18-69 years  Women: 58%  Response rate: NR  Adults  Saudi Arabia | Cross-sectional  Cluster: none  Individuals: convenience  Stratification: none  Neighborhood definition: none | *Exposure variable*  Lack of exercise facilities  *Measure:* Perceived  [1 item]  *Validation:* No | *Outcome*  Regular exercise [Yes/No]  *Measure:* Self-report  [International Physical Activity Questionnaire (IPAQ-SF)]  *Validation:* Yes | None | Logistic regression | Age, sex, marital status, nationality, smoker, lifestyle diseases, level of education, employment status, resilience scores, total number of diseases, BMI, motivation, home space, time, family and work responsibility | *No regular exercise*  **Lack of exercise facilities**: OR=2.27, p<0.05 |
| 7 | Home-interview survey  Arasan, 1996  [7] | N= 15 148 (urban)  Age: NR  Women: NR  Response rate: NR  Community dwellers  Tiruchirapalli, India | Cross-sectional  Cluster: none  Individuals: NR  Stratification: none  Neighborhood definition: none | *Exposure variable*  Travel distance  *Measure:* Perceived  [NR]  *Validation:* NR | *Outcome*  Transport-related walking [Yes/No]  *Measure:* Self-report  [NR]  *Validation:* No | Urbanicity level (central, urban, suburban) | Logit model | Worker-household-size-ratio, age, gender, head of household, education level, bias constant for walking | *Transport-related walking*  Travel distance  **Central**: β = -4.41, p<0.05  **Urban**: β=-3.99, p<0.05  **Suburban**: β=-3.87, p<0.05 |
| 8 | NA  Awadalla et al., 2014  [8] | N= 1257 (mixed)  Mean age 20 ± 1 years  Women: 66%  Response rate: 86%  Health college students  Abha, Saudi Arabia | Cross-sectional  Cluster: purposive  Individuals: random  Stratification: college type, academic level  Neighborhood definition: none | *Exposure variable*  1. Lack of accessible and suitable sports places  2. Lack of safe sports places  *Measure:* Perceived  [2 items]  *Validation:* No | *Outcome*  Physically active (150+ min/week moderate-to-vigorous physical activity (Yes/No)  *Measure:* Self-report  [International Physical Activity Questionnaire (IPAQ-SF)]  *Validation:* Yes | None | T-test and Mann-Whitney-U test | None | *Physically active*  1. **Lack of accessible and suitable sports places**, p<0.05  Physically active: 23.5%  Physically inactive: 31.1%  2. **Lack of safe sports places**, p<0.05  Physically active: 16.5%  Physically inactive: 22.8% |
| 9 | RESIDE,  Badland et al., 2013  **[9]** | N=909 (urban)  Mean age 43 ± 12 years  Women: 63%  Response rate: 33%  Relocating adults, non-cycling at baseline (community dwellers)  Perth, Australia | Natural experiment  Cluster: Purposive  Individuals: Two-stage approach; 1) households (census); 2) individuals (random)  Stratification: Relocating development type (Livable Neighborhood, hybrid, conventional)  Neighborhood definition: housing development (recruitment level) ; 1600m street network buffer and 15 min walk from home (participant level) | *Exposure variables – Perceived (T2)*  1. Lots of neighborhood greenery  2. Interesting features in the neighborhood  3. Attractive buildings and homes in the neighborhood  4. Pleasant natural features in the neighborhood  5. Presence of neighborhood walking/cycling paths  6. Neighborhood near busy road  7. Traffic speeds slow on nearby streets  8. Many traffic-slowing devices in the neighborhood  9. Neighborhood streets not many cul-de-sacs  10 Neighborhood many four-way-intersections  11. Many alternative routes in the neighborhood  *Measure:* Perceived  [Neighborhood Environment Walkability Scale (NEWS)]  *Validation:* Yes  *Exposure variables – Objective (T2)*  12. Street connectivity  13. Residential density  14. Land use mix – recreation  15. Land use mix – transport  16. Walkability index – recreation  17. Walkability index – transport  18. Cyclable road ratio  19. Cyclable path length  *Measure:* Objective  [ArcGIS, various databases (see Foster 2014)]  *Validation:* Yes | *Outcome:*  Recreational cycling uptake at T3 (At least once in within a usual weak; Yes/No)  Transport-related cycling uptake at T3 (At least once in within a usual weak; Yes/No)  *Measure:* Self-report [Neighborhood Physical Activity Questionnaire (NPAQ)]  *Validity:* Yes | None | Logistic regression models accounting for housing estate clustering | Age, gender, education, income | *Recreational cycling uptake*  Perceived  1. Lots of neighborhood greenery: OR = 1.57, p>0.05  2. Interesting features in the neighborhood: OR=0.68, p>0.05  3. Attractive buildings and homes in the neighborhood: OR=0.76, p>0.05  4. Pleasant natural features in the neighborhood: OR=0.61, p>0.05  5. Presence of neighborhood walking/cycling paths: OR=1.15, p>0.05  6. **Neighborhood near busy road**: OR=0.49, p<0.05  7. Traffic speeds slow on nearby streets: OR=1.13, p>0.05  8. Many traffic-slowing devices in the neighborhood: OR=0.82, p>0.05  9. Neighborhood streets not many cul-de-sacs: OR=1.43, p>0.05  10 Neighborhood many four-way-intersections: OR=1.20, p>0.05  11. Many alternative routes in the neighborhood: OR=1.90, p>0.05  Objective  12. **Street connectivity**: OR=1.80, p<0.05  13. Residential density: OR=1.14, p>0.05  14. Land use mix – recreation: OR=1.16, p>0.05  15. Land use mix – transport: OR=1.37, p>0.05  16. **Walkability index – recreation**: OR=1.63, p<0.05  17. Walkability index – transport: OR=1.51, p>0.05  18. Cyclable road ratio: OR=0.82, p>0.05  19. Cyclable path length within 1600m service area: OR=1.31, p>0.05  *Transport-related cycling uptake*  Perceived  1. Lots of neighborhood greenery: OR = 2.40, p>0.05  2. Interesting features in the neighborhood: OR=1.47, p>0.05  3. Attractive buildings and homes in the neighborhood: OR=0.98, p>0.05  4. Pleasant natural features in the neighborhood: OR=0.69, p>0.05  5. Presence of neighborhood walking/cycling paths: OR=0.80, p>0.05  6. Neighborhood near busy road: OR=0.70, p>0.05  7. Traffic speeds slow on nearby streets: OR=1.40, p>0.05  8. Many traffic-slowing devices in the neighborhood: OR=1.23, p>0.05  9. Neighborhood streets not many cul-de-sacs: OR=0.88, p>0.05  10 Neighborhood many four-way-intersections: OR=0.93, p>0.05  11. Many alternative routes in the neighborhood: OR=1.54, p>0.05  Objective  12. Street connectivity: OR=1.58, p>0.05  13. Residential density: OR=1.46, p>0.05  14. Land use mix – recreation: OR=1.02, p>0.05  15. Land use mix – transport: OR=0.83, p>0.05  16. Walkability index – recreation: OR=1.44, p>0.05  17. Walkability index – transport: OR=1.45, p>0.05  18. Cyclable road ratio: OR=1.15, p>0.05  19. Cyclable path length within 1600m service area: OR=1.01, p>0.05 |
| 10 | UNVL Student Survey,  Bartshe et al, 2018  [10] | N=410 (mixed)  Mean age: 24 years  Women: 77%  Response rate: 2%  College students  Las Vegas, USA | Cross-sectional  Cluster: none  Individuals: convenience  Stratification: none  Neighborhood definition: none | *Exposure variables:*  1. Residential density  2. Land use mix (diversity)  3. Land use mix (access)  4. Street connectivity  5. Infrastructure and safety for walking and cycling  6. Aesthetics  7. Traffic safety  8. Crime safety  9. Lack of parking  10. Lack of cul-de-sacs  11. Physical barriers  *Measure:* Perceived  [Neighborhood Environment Walkability Scale (NEWS-A)]  *Validation:* Yes | *Outcome:*  Meeting the physical activity recommendations (150+ min/week; Yes/No)  *Measure:* Self-report [International Physical Activity Questionnaire (IPAQ)]  *Validity:* Yes | None | Logistic regression | Age, gender, ethnicity, vehicle owning, social capital | *Meeting PA recommendations*  1. Residential density: OR = 1.001, p>0.05  2. Land use mix (diversity): OR=1.125, p>0.05  3. Land use mix (access): OR = 1.187, p >0.05  4. Street connectivity: OR = 1.234, p >0.05  5. Infrastructure and safety for walking and cycling: OR = 0.870, p >0.05  6. Aesthetics: OR = 0.889, p >0.05  7. Traffic safety: OR = 0.923, p >0.05  8. Crime safety: OR = 1.005, p >0.05  9. Lack of parking: OR = 0.876, p >0.05  10. Lack of cul-de-sacs: OR = 1.112, p >0.05  11. Physical barriers: OR = 1.090, p >0.05 |
| 11 | UNVL Student Survey,  Bartshe et al., 2021  [11] | N=403 (mixed)  Mean age: 24 years  Women: 76%  Response rate: 2%  College students  Las Vegas, USA | Cross-sectional  Cluster: none  Individuals: convenience  Stratification: none  Neighborhood definition: 1km street network buffer (participant level) | *Exposure variables:*  1. Tree canopy  *Measure:* Objective  [ArcGIS, database: Nevada Division of Forestry data]  *Validation:* Yes | *Outcome:*  Meeting the physical activity recommendations (150+ min/week; Yes/No)  *Measure:* Self-report [International Physical Activity Questionnaire (IPAQ)]  *Validity:* Yes | None | Logistic regression | Age, gender, ethnicity, social capital | *Meeting PA recommendations*  1. Tree canopy: OR = 1.03, p>0.05 |
| 12 | RESIDE,  Beenackers et al., 2012  [12] | N=1289 (urban)  Mean age 41 ± 12 years  Women: 62%  Response rate: 33%  Relocating adults, non-cycling at baseline (community dwellers)  Perth, Australia | Natural experiment  Cluster: Purposive  Individuals: Two-stage approach; 1) households (census); 2) individuals (random)  Stratification: Relocating development type (Livable Neighborhood, hybrid, conventional)  Neighborhood definition: housing development (recruitment level) ; 1600m street network buffer and 15 min walk from home (participant level) | *Exposure variables - Perceived: Changes (T1-T2) in*  1. Access to mixed services  2. Neighborhood aesthetics  3. Traffic hazards  4. Major barriers present  5. Local parking is difficult  6. Access to park  7. Access to cycling paths  8. Pedestrian crossings present  9. Number of transport destinations  10. Number of recreation destinations  11. Crime hazards  12. Hilly streets  13. Many alternative routes  *Measure:* Perceived  [Neighborhood Environment Walkability Scale (NEWS)]  *Validation:* Yes  *Exposure variables - Objective: Changes (T1-T2) in*  11. Connectivity  12. Residential density  13. Land-use mix  14. Numbers of destinations for transport  15. Numbers of destinations for recreation  *Measure:* Objective  [ArcGIS, various databases (see Foster 2014)]  *Validation:* Yes | *Outcome:*  Cycling uptake at T2 (At least once in within a usual weak; Yes/No)  *Measure:* Self-report [Neighborhood Physical Activity Questionnaire (NPAQ)]  *Validity:* Yes | None | Logistic regression models with generalized estimating equations accounting for clustering within neighborhoods | Age, gender, education, marital status, children<18, car access, attitude, self-efficacy, social influence, intention | *Cycling uptake for transport*  Perceived  1. Access to mixed services: OR=1.42, p>0.05  2. Neighborhood aesthetics: OR=0.86, p>0.05  3. Traffic hazards: OR=0.98, p>0.05  4. Major barriers present: OR=0.98, p>0.05  5. Local parking is difficult: OR=0.96, p>0.05  **6. Access to park:** OR=2.60, p<0.05  7. Access to cycling paths: OR=1.07, p>0.05  8. Pedestrian crossings present: OR=1.33, p>0.05  9. Number of transport destinations: OR=0.96, p>0.05  10. **Number of recreation destinations**: OR=1.57, p<0.05  11. Crime hazards: OR=0.95, p>0.05  12. Hilly streets: OR=0.79, p>0.05  13. Many alternative routes: OR=1.08, p>0.05  Objective  14. Connectivity: OR=0.88, p>0.05  **15. Residential density:** OR=1.54, p<0.05  16. Land-use mix: OR=NR, p>0.05  17. Numbers of destinations for transport: OR=NR, p>0.05  18. Numbers of destinations for recreation: OR=NR, p>0.05  *Cycling uptake for recreation*  Perceived  1. Access to mixed services: OR=NR, p>0.05  2. Neighborhood aesthetics: OR=1.06, p>0.05  3. Traffic hazards: OR=NR, p>0.05  4. Major barriers present: OR=0.97, p>0.05  5. Local parking is difficult: OR=NR, p>0.05  6. Access to park: OR=1.14, p>0.05  7. Access to cycling paths: OR=1.03, p>0.05  8. Pedestrian crossings present: OR=NR, p>0.05  9. Number of transport destinations: OR=NR, p>0.05  10. Number of recreation destinations: OR=1.12, p>0.05  11. Crime hazards: OR=0.95, p>0.05  12. Hilly streets: OR=0.79, p>0.05  13. Many alternative routes: OR=1.08, p>0.05  Objective  14. **Connectivity**: OR=1.20, p<0.05  15. Residential density: OR=1.54, p<0.05  16. Land-use mix: OR=NR, p>0.05  17. Numbers of destinations for transport: OR=0.95, p>0.05  18. Numbers of destinations for recreation: OR=NR, p>0.05 |
| 13 | NA  Bungum et al, 2012  [13] | N=236 (urban)  Mean age 49 ± 18 years  Women: 65%  Response rate: NR  Asian and Asian-Pacific Islander Americans (community dwellers)  Las Vegas, USA | Cross-sectional  Cluster: none  Individuals: convenience  Stratification: none  Neighborhood definition: none | *Exposure variables:*  1. Environmental physical activity support (consisting of: sidewalk presence, nearness to recreation areas, nearby grocery stores)  2. Neighborhood safety (consisting of crime, neighborhood pleasantness as a place to walk, motorized traffic heaviness)  *Measure:* Perceived  [8 items]  *Validation:* No | *Outcome*  Being physically active (Yes/No)  *Measure*: Self-report  [8 single-response PA items with participants responding to the one that best fit their recent physical activity behavior]  *Validated*: Yes | None | Logistic regression | Age, gender, BMI, employment, marital status, educational attainment, neighborhood safety | *Main effects*  **1. Environmental physical activity support**  OR=1.52, p<0.05  2. Neighborhood safety: OR=1.15, p>0.05 |
| 14 | Healthy Austin Study,  Calise et al., 2012  [14] | N= 267 (urban)  Age: 54% 20-39 years  Women: 62%  Response rate: 63%  Relocating adults (community dwellers)  Mueller, Texas, USA | Natural experiment  Cluster: none  Individuals: census  Stratification: none  Neighborhood definition: New-urbanist development Mueller (recruitment level) and 10-15 minute walk from home (participant level) | *Exposure variable:*  Moving to new urbanist-designed development , Design, Distribution of intervention and resources  *Measure:* Objective (homes with activated electricity)  *Validation:* Yes | *Outcome: Pre-post duration changes in…*  1. Recreational physical activity  2. Walking for recreation  3. Biking for recreation  4. Total walking and biking for recreation  5. Total recreational physical activity  6. Walking for transport  7. Biking for transport  8. Total transport-related physical activity  *Measure*: Self-report  [Neighborhood Physical Activity Questionnaire [NPAQ]]  *Validated*: Yes | Inside / outside neighborhood | Paired sample t-test; Wilcoxon signed-rank test | None | *Main effects - Changes in…(min/week, mean (SD))*  1. **Recreational physical activity**  **Moderate**: 15.9, p<0.05  **Vigorous:** 9.9, p>0.05  **Moderate-vigorous**: 27.9, p<0.05  2. **Walking for recreation**  **Inside neighborhood**: 68.1, p<0.05  **Outside neighborhood**: -18.6, p<0.05  **Total**: 46.1, p<0.05  3. Biking for recreation  Inside neighborhood: 4.1, p>0.05  Outside neighborhood: -3.3, p>0.05  Total: 0.8, p>0.05  4. **Total walking and biking for recreation**  **Inside neighborhood**: 72.0, p<0.05  **Outside neighborhood**: -21.8, p<0.05  **Total**: 46.7, p<0.05  5. **Total recreational physical activity**  **Moderat**e: 63.8, p<0.05  Vigorous: 9.9, p>0.05  **Moderate-vigorous**: 73.9, p<0.05  6. **Walking for transport**  **Inside neighborhood**: -10.8, p<0.05  Outside neighborhood: -0.04, p>0.05  **Total**: -9.6, p<0.05  7. Biking for transport  Inside neighborhood: 4.4, p>0.05  Outside neighborhood: 2.3, p>0.05  Total: 6.6, p=0.05  8. Total transport-related physical activity  Inside neighborhood: -7.6, p>0.05  Outside neighborhood: 2.0, p>0.05  Total: -3.8, p>0.05 |
| 15 | Healthy Austin Study,  Calise et al., 2013  [15] | N= 267 (urban)  Age: 54% 20-39 years  Women: 62%  Response rate: 63%  Relocating adults (community dwellers)  Mueller, Texas, USA | Natural experiment  Cluster: none  Individuals: census  Stratification: none  Neighborhood definition: New-urbanist development Mueller (recruitment level) and 10-15 minute walk from home (participant level) | *Exposure variable:*  Moving to new urbanist-designed development  *Measure:* Objective (homes with activated electricity)  *Validation:* NA | *Outcome: Pre-post changes in…*  1. Transport-related physical activity  2. Recreational walking and biking  3. Total recreational physical activity  4. Total physical activity  *Measure*: Self-report  [Neighborhood Physical Activity Questionnaire [NPAQ]]  *Validated*: Yes | Pre-move physical activity levels (low, middle, high) | Paired sample t-test; Wilcoxon signed-rank test, one-way ANOVA | Importance of neighborhood characteristics, pre-move physical activity levels | *Main effects - Changes in…(min/week, mean (SD))*  1. Recreational / leisure PA – moderate (excl. walking/biking)  Low activity group: 24.1 (80.7)  Medium activity group:18.3 (95.4)  High activity group:3.7 (165.9)  p>0.05  **2. Recreational / leisure PA – vigorous** (excl. walking/biking)  Low activity group: 40.1 (109.3)  Medium activity group: 16.0 (75.0)  High activity group: -27.5 (142.3)  p<0.05  **3. Recreational / leisure PA – moderate-vigorous** (excl. walking/biking)  Low activity group: 64.6 (140.9)  Medium activity group: 34.3 (139.2)  High activity group: -17.9 (231.7)  p<0.05  4. Recreational walking – inside neighborhood  Low activity group: 100.7 (143.2)  Medium activity group: 47.3 (125.6)  High activity group: 56.5 (190.0)  p = 0.05  **5. Recreational walking – outside neighborhood**  Low activity group: -2.0 (27.6)  Medium activity group: -18.2 (60.2)  High activity group: -40.2 (79.1)  p<0.05  **6. Recreational walking – total**  Low activity group: 98.7 (146.8)  Medium activity group: 29.7 (133.7)  High activity group: 3.5 (180.5)  p<0.01  7. Recreational biking – inside neighborhood  Low activity group: 14.1 (33.4)  Medium activity group: 7.2 (53.9)  High activity group: -9.9 (110.9)  p>0.05  **8. Recreational biking – outside neighborhood**  Low activity group: 2.2 (12.3)  Medium activity group: 8.6 (38.6)  High activity group: -22.5 (83.0)  p<0.05  9. **Recreational biking – total**  Low activity group: 16.3 (38.8)  Medium activity group: 15.9 (68.0)  High activity group: -32.6 (168.2)  p<0.05  **10. Recreational biking and walking – in neighborhood**  Low activity group: 115.3 (145.5)  Medium activity group: 53.6 (144.7)  High activity group: 46.5 (216.5)  p<0.05  **11. Recreational biking and walking – outside neighborhood**  Low activity group: 0.2 (30.4)  Medium activity group: -9.1 (79.2)  High activity group: -63.3 (120.6)  p<0.05  **12. Recreational biking and walking – total**  Low activity group: 115.5 (152.6)  Medium activity group: 45.1 (171.0)  High activity group: -44.5 (331.1)  p<0.05  **13. Recreational physical activity – moderate** (incl. walking/biking)  Low activity group: 137.8 (174.6)  Medium activity group: 62.4 (194.5)  High activity group: -16.0 (288.0)  p<0.05  **14. Recreational physical activity – vigorous** (incl. walking/biking)  Low activity group: 40.1 (109.3)  Medium activity group: 16.0 (75.0)  High activity group: -27.5 (142.3)  p<0.05  **15. Recreational physical activity – total** (incl. walking/biking)  Low activity group: 178.8 (220.5)  Medium activity group: 77.4 (219.3)  High activity group: -44.5 (331.1)  p<0.05  16. Walking for transport – inside neighborhood  Low activity group: 0.8 (35.5)  Medium activity group: -7.9 (45.4)  High activity group: -20.3 (97.6)  p>0.05  17. Walking for transport – outside neighborhood  Low activity group: 4.1 (14.1)  Medium activity group: -1.6 (15.3)  High activity group: -4.2 (48.3)  p>0.05  **18. Walking for transport – total**  Low activity group: 3.8 (40.5)  Medium activity group: -10.0 (48.0)  High activity group: -24.6 (109.1)  p<0.05  19. Biking for transport – in neighborhood  Low activity group: 6.9 (44.8)  Medium activity group: 3.8 (22.3)  High activity group: 1.3 (57.8)  p>0.05  20. Biking for transport – outside neighborhood  Low activity group: 4.1 (26.1)  Medium activity group: 1.9 (17.3)  High activity group: 1.2 (42.9)  p>0.05  21. Biking for transport – total  Low activity group: 10.2 (38.3)  Medium activity group: 5.2 (36.4)  High activity group: 2.5 (80.9)  p>0.05  22. Transport physical activity – in neighborhood  Low activity group: 6.9 (44.8)  Medium activity group: -5.7 (50.1)  High activity group: -18.9 (112.3)  p>0.05  23. Transport physical activity – outside neighborhood  Low activity group: 8.3 (29.5)  Medium activity group: 0.3 (23.5)  High activity group: -3.1 (67.5)  p>0.05  **24. Transport physical activity – total**  Low activity group: 14.3 (60.8)  Medium activity group: -6.3 (55.8)  High activity group: -22.0 (133.4)  p<0.05  **25. Total physical activity – moderate**  Low activity group: 140.1 (159.7)  Medium activity group: 52.7 (202.7)  High activity group: -39.2 (289.4)  p<0.05  **26. Total physical activity – vigorous**  Low activity group: 40.1 (109.3)  Medium activity group: 16.0 (75.0)  High activity group: -27.5 (142.3)  p<0.05  **27. Total physical activity – moderate-to-vigorous**  Low activity group: 176.3 (188.1)  Medium activity group: 69.5 (227.4)  High activity group: -67.9 (321.7)  p<0.05 |
| 16 | NA  Cao et al., 2006  [16] | N= 1368 (urban)  Median age: 43-46 years  Women: 46-50%  Response rate: 23%  Community dwellers  Austin, Texas, USA | Cross-sectional  Cluster: purposive  Individuals: 1) households (random), 2) individuals (random)  Stratification: development era  Neighborhood definition: neighborhood boundaries (recruitment level) | *Exposure variable - Perceived*  1. Safety  2. Shade  3. Traffic comfortable  4. People  5. Walk advantage  6. Walk comfort  7. Store perception  *Measure:* Perceived  [NR]  *Validation:* No  *Exposure variable - Objective*  8. Store traffic volume  9. Pedestrians connections  10. Miles to the nearest store  *Measure:* Objective  [GIS, hardcopy maps, aerial photos, site visits]  *Validation:* Yes | *Outcome*  Frequency of strolling trips in the last 30 days  Frequency of trips walking to the store within the last 30 days  *Measure*: Self-report  [NR]  *Validated*: No | None | Negative binomial regression without accounting for clustering | *Strolling model:*  Age, residential preference, pets to walk, age, full-time worker, presence of kids<5 years in the household, importance of having stores (self-selection  *Walk-to-store model*:  Gender, residential preference, age, full-time worker, presence of kids<5 years, household income, importance of having stores (self-selection | *Main effects – Strolling frequency*  1. **Safety:** β=0.14, p<0.05  2**. Shade:** β=0.18; p<0.05  3. Traffic comfortable: β=0.07, p>0.05  4. **People:** β=0.11, p<0.05  *Main effects – Walk-to-store frequency*  3. **Traffic comfortable:** β=0.16, p>0.05  5. **Walk advantage:** β=0.28, p<0.05  6. **Walk comfort:** β=0.18, p<0.05  7. **Store perception:** β=0.21, p<0.05  8. Store traffic volume: β=-0.11; p>0.05  **9. Pedestrian connection:** β=0.39; p<0.05  10. **Miles to the nearest store:** β= -0.31; p<0.05 |
| 17 | RESIDE  Christian et al., 2011  [17] | N=1047 (urban)  Mean age 42 ± 12 years  Women: 61%  Response rate: 33%  Relocating adults  Perth, Australia | Cross-sectional  Cluster: Purposive  Individuals: Two-stage approach; 1) households (census); 2) individuals (random)  Stratification: Relocating development type (Livable Neighborhood, hybrid, conventional)  Neighborhood definition: housing development (recruitment level) ; 1600m street network buffer and 15 min walk from home (participant level) | *Exposure variable - Objective*  Walkability indices (WI) including street connectivity, net residential density, and different configurations of and land mix use (LUM) including:  1. WI with LUM1: Retail, office, health/welfare/community, and residential  2. WI with LUM2: Retail, office, health/welfare/community, residential, entertainment, culture, and recreation  3. WI with LUM3: Retail, office, health/welfare/community, residential, entertainment, culture, and recreation, and public open space, sporting infrastructure & primary and rural  4. WI with LUM4: Retail, office, health/welfare/community, residential, entertainment, culture, and recreation, and public open space, sporting infrastructure & primary and rural, and unclassified  5. WI with LUM5: Retail, health/welfare/community, residential, entertainment, culture, and recreation, and public open space, sporting infrastructure & primary and rural, and unclassified  6. Net residential density  7. Street connectivity  8. Land use mix  *Measure:* Objective  [GIS, GIS database: land tenure (taxation/rating) records and reserve vesting information; indices based upon Frank et al.]  *Validation:* Yes | *Outcome:*  Any recreational, transport-related or total neighborhood walking >0 minutes [Yes/No]  Recreational, transport-related or total neighborhood walking ≥ 60 min/week [Yes/No]  Recreational, transport-related or total neighborhood walking ≥ 150 min/week [Yes/No]  *Measure:* Self-report [Neighborhood Physical Activity Questionnaire (NPAQ)]  *Validity:* Yes | None | Logistic regression | Gender, age, education, marital status, presence of children at home | *Any transport walking*  1. **WI with LUM1** (z-score): OR=1.12, p<0.05  6. Residential density (z-score): OR=1.10, p>0.05  7. **Street connectivity** (z-score): OR=1.14, p<0.05  8. **Land use mix** (z-score): OR=1.13, p<0.05  2. **WI with LUM2** (z-score): OR=1.13, p<0.05  6. Residential density (z-score): OR=1.10, p>0.05  7. **Street connectivity** (z-score): OR=1.15, p<0.05  8. **Land use mix** (z-score): OR=1.15, p<0.05  3. **WI with LUM3** (z-score): OR=1.10, p<0.05  6. **Residential density** (z-score): OR=1.14, p<0.05  7. **Street connectivity** (z-score): OR=1.14, p<0.05  8. Land use mix (z-score): OR=1.03, p<0.05  4. **WI with LUM4** (z-score): OR=1.09, p<0.05  6. **Residential density** (z-score): OR=1.15, p<0.05  7. **Street connectivity** (z-score): OR=1.15, p<0.05  8. Land use mix (z-score): OR=0.97, p<0.05  5. **WI with LUM5** (z-score): OR=1.10, p<0.05  6. **Residential density** (z-score): OR=1.14, p<0.05  7. **Street connectivity** (z-score): OR=1.14, p<0.05  8. Land use mix (z-score): OR=1.02, p<0.05  *Any recreational walking*  1. WI with LUM1 (z-score): OR=1.02, p>0.05  6. Residential density (z-score): OR=0.99, p>0.05  7. Street connectivity (z-score): OR=1.06, p>0.05  8. Land use mix (z-score): OR=1.03, p>0.05  2. WI with LUM2 (z-score): OR=1.02, p>0.05  6. Residential density (z-score): OR=1.00, p>0.05  7. Street connectivity (z-score): OR=1.06, p>0.05  8. Land use mix (z-score): OR=1.02, p>0.05  3. WI with LUM3 (z-score): OR=1.03, p>0.05  6. Residential density (z-score): OR=0.99, p>0.05  7. Street connectivity (z-score): OR=1.05, p>0.05  8. Land use mix (z-score): OR=1.04, p>0.05  4. WI with LUM4 (z-score): OR=1.04, p>0.05  6. Residential density (z-score): OR=0.98, p>0.05  7. Street connectivity (z-score): OR=1.05, p>0.05  8. **Land use mix** (z-score): OR=1.12, p<0.05  5. WI with LUM5 (z-score): OR=1.03, p>0.05  6. Residential density (z-score): OR=0.99, p>0.05  7. Street connectivity (z-score): OR=1.05, p>0.05  8. Land use mix (z-score): OR=1.04, p>0.05  *Any total walking*  1. **WI with LUM1** (z-score): OR=1.06, p<0.05  6. Residential density (z-score): OR=1.03, p>0.05  7. Street connectivity (z-score): OR=1.06, p>0.05  8. Land use mix (z-score): OR=1.10, p>0.05  2. **WI with LUM2** (z-score): OR=1.06, p<0.05  6. Residential density (z-score): OR=1.04, p>0.05  7. Street connectivity (z-score): OR=1.06, p>0.05  8. Land use mix (z-score): OR=1.10, p>0.05  3. **WI with LUM3** (z-score): OR=1.06, p<0.05  6. Residential density (z-score): OR=1.05, p>0.05  7. Street connectivity (z-score): OR=1.06, p>0.05  8. Land use mix (z-score): OR=1.07, p>0.05  4. **WI with LUM4** (z-score): OR=1.07, p<0.05  6. Residential density (z-score): OR=1.05, p>0.05  7. Street connectivity (z-score): OR=1.06, p>0.05  8. Land use mix (z-score): OR=1.10, p>0.05  5. **WI with LUM5** (z-score): OR=1.06, p<0.05  6. Residential density (z-score): OR=1.05, p>0.05  7. Street connectivity (z-score): OR=1.05, p>0.05  8. Land use mix (z-score): OR=1.06, p>0.05  *Transport walking ≥ 60 min*  1. **WI with LUM1** (z-score): OR=1.11, p<0.05  6. Residential density (z-score): OR=1.03, p>0.05  7. **Street connectivity** (z-score): OR=1.22, p<0.01  8. Land use mix (z-score): OR=1.12, p>0.05  2. **WI with LUM2** (z-score): OR=1.12, p<0.05  6. Residential density (z-score): OR=1.03, p>0.05  7. **Street connectivity** (z-score): OR=1.22, p<0.05  8. **Land use mix** (z-score): OR=1.14, p<0.05  3. **WI with LUM3** (z-score): OR=1.11, p<0.05  6. Residential density (z-score): OR=1.05, p>0.05  7. **Street connectivity** (z-score): OR=1.21, p<0.05  8. Land use mix (z-score): OR=1.07, p>0.05  4. **WI with LUM4** (z-score): OR=1.11, p<0.05  6. Residential density (z-score): OR=1.05, p>0.05  7. **Street connectivity** (z-score): OR=1.21, p<0.05  8. Land use mix (z-score): OR=1.09, p>0.05  5. **WI with LUM5** (z-score): OR=1.10, p<0.05  6. Residential density (z-score): OR=1.06, p>0.05  7. **Street connectivity** (z-score): OR=1.21, p<0.05  8. Land use mix (z-score): OR=1.04, p>0.05  *Recreational walking ≥ 60 min*  1. WI with LUM1 (z-score): OR=1.02, p>0.05  6. Residential density (z-score): OR=1.03, p>0.05  7. Street connectivity (z-score): OR=1.03, p>0.05  8. Land use mix (z-score): OR=0.98, p>0.05  2. WI with LUM2 (z-score): OR=1.01, p>0.05  6. Residential density (z-score): OR=1.03, p>0.05  7. Street connectivity (z-score): OR=1.03, p>0.05  8. Land use mix (z-score): OR=0.98, p>0.05  3. WI with LUM3 (z-score): OR=1.02, p>0.05  6. Residential density (z-score): OR=1.02, p>0.05  7. Street connectivity (z-score): OR=1.03, p>0.05  8. Land use mix (z-score): OR=1.02, p>0.05  4. WI with LUM4 (z-score): OR=1.04, p>0.05  6. Residential density (z-score): OR=1.01, p>0.05  7. Street connectivity (z-score): OR=1.02, p>0.05  8. Land use mix (z-score): OR=1.10, p>0.05  5. WI with LUM5 (z-score): OR=1.03, p>0.05  6. Residential density (z-score): OR=1.02, p>0.05  7. Street connectivity (z-score): OR=1.02, p>0.05  8. Land use mix (z-score): OR=1.03, p>0.05  *Total walking ≥ 60 min*  1. **WI with LUM1 (z-score)**: OR=1.06, p<0.06  6. Residential density (z-score): OR=1.04, p>0.05  7. Street connectivity (z-score): OR=1.08, p>0.05  8. Land use mix (z-score): OR=1.07, p>0.05  2. **WI with LUM2 (z-score)**: OR=1.06, p<0.06  6. Residential density (z-score): OR=1.04, p>0.05  7. Street connectivity (z-score): OR=1.08, p>0.05  8. Land use mix (z-score): OR=1.07, p>0.05  3. **WI with LUM3 (z-score)**: OR=1.06, p<0.06  6. Residential density (z-score): OR=1.04, p>0.05  7. Street connectivity (z-score): OR=1.07, p>0.05  8. Land use mix (z-score): OR=1.07, p>0.05  4. **WI with LUM4 (z-score)**: OR=1.08, p<0.06  6. Residential density (z-score): OR=1.04, p>0.05  7. Street connectivity (z-score): OR=1.07, p>0.05  8. Land use mix (z-score): OR=1.13, p>0.05  5. **WI with LUM5 (z-score)**: OR=1.06, p<0.06  6. Residential density (z-score): OR=1.04, p>0.05  7. Street connectivity (z-score): OR=1.07, p>0.05  8. Land use mix (z-score): OR=1.06, p>0.05  *Transport walking ≥ 150 min*  1. **WI with LUM1 (z-score)**: OR=1.12, p<0.05  6. Residential density (z-score): OR=1.12, p>0.05  7. Street connectivity (z-score): OR=1.26, p>0.05  8. Land use mix (z-score): OR=0.99, p>0.05  2. **WI with LUM2** (z-score): OR=1.12, p<0.05  6. Residential density (z-score): OR=1.11, p>0.05  7. Street connectivity (z-score): OR=1.26, p>0.05  8. Land use mix (z-score): OR=1.01, p>0.05  3. WI with LUM3 (z-score): OR=1.09, p>0.05  6. Residential density (z-score): OR=1.16, p>0.05  7**. Street connectivity** (z-score): OR=1.28, p<0.05  8. Land use mix (z-score): OR=0.85, p>0.05  4. **WI with LUM4** (z-score): OR=1.10, p<0.05  6. Residential density (z-score): OR=1.14, p>0.05  7**. Street connectivity** (z-score): OR=1.29, p<0.05  8. Land use mix (z-score): OR=0.89, p>0.05  5. WI with LUM5 (z-score): OR=1.08, p>0.05  6. Residential density (z-score): OR=1.18, p>0.05  7**. Street connectivity** (z-score): OR=1.30, p<0.05  8. **Land use mix** (z-score): OR=0.78, p<0.05  *Recreation walking ≥ 150 min*  1. WI with LUM1 (z-score): OR=1.01, p>0.05  6. Residential density (z-score): OR=1.00, p>0.05  7. Street connectivity (z-score): OR=1.02, p>0.05  8. Land use mix (z-score): OR=1.02, p>0.05  2. WI with LUM2 (z-score): OR=1.01, p>0.05  6. Residential density (z-score): OR=1.00, p>0.05  7. Street connectivity (z-score): OR=1.02, p>0.05  8. Land use mix (z-score): OR=1.02, p>0.05  3. WI with LUM3 (z-score): OR=1.01, p>0.05  6. Residential density (z-score): OR=1.00, p>0.05  7. Street connectivity (z-score): OR=1.02, p>0.05  8. Land use mix (z-score): OR=1.03, p>0.05  4. WI with LUM4 (z-score): OR=1.02, p>0.05  6. Residential density (z-score): OR=1.00, p>0.05  7. Street connectivity (z-score): OR=1.02, p>0.05  8. Land use mix (z-score): OR=1.05, p>0.05  5. WI with LUM5 (z-score): OR=1.02, p>0.05  6. Residential density (z-score): OR=0.99, p>0.05  7. Street connectivity (z-score): OR=1.01, p>0.05  8. Land use mix (z-score): OR=1.06, p>0.05  *Total walking ≥ 150 min*  1. WI with LUM1 (z-score): OR=1.04, p>0.05  6. Residential density (z-score): OR=1.03, p>0.05  7. Street connectivity (z-score): OR=1.06, p>0.05  8. Land use mix (z-score): OR=1.01, p>0.05  2. WI with LUM2 (z-score): OR=1.04, p>0.05  6. Residential density (z-score): OR=1.03, p>0.05  7. Street connectivity (z-score): OR=1.06, p>0.05  8. Land use mix (z-score): OR=1.02, p>0.05  3. WI with LUM3 (z-score): OR=1.03, p>0.05  6. Residential density (z-score): OR=1.04, p>0.05  7. Street connectivity (z-score): OR=1.06, p>0.05  8. Land use mix (z-score): OR=1.01, p>0.05  4. WI with LUM4 (z-score): OR=1.05, p>0.05  6. Residential density (z-score): OR=1.03, p>0.05  7. Street connectivity (z-score): OR=1.06, p>0.05  8. Land use mix (z-score): OR=1.06, p>0.05  5. WI with LUM5 (z-score): OR=1.04, p>0.05  6. Residential density (z-score): OR=1.03, p>0.05  7. Street connectivity (z-score): OR=1.06, p>0.05  8. Land use mix (z-score): OR=1.02, p>0.05 |
| 18 | RESIDE  Christian et al., 2013  [18] | N=1047 (urban)  Mean age 42 ± 12 years  Women: 61%  Response rate: 33%  Relocating adults  Perth, Australia | Natural experiment  Cluster: Purposive  Individuals: Two-stage approach; 1) households (census); 2) individuals (random)  Stratification: Relocating development type (Livable Neighborhood, hybrid, conventional)  Neighborhood definition: housing development (recruitment level) ; 1600m street network buffer and 15 min walk from home (participant level) | *Exposure variables*  Livable, hybrid, or conventional neighborhood  Livable neighborhoods: Diversity, Design, Desirability, Destination accessibility, Destination proximity, Disaster mitigation  *Measure:* Objective  [Water state authority]  *Validation:* Yes | *Outcome:*  Changes in recreational, transport-related, and total neighborhood walking [min/week] across three timepoints (T1, T2, T3)  *Measure:* Self-report [Neighborhood Physical Activity Questionnaire (NPAQ)]  *Validity:* Yes | None | General linear models accounting for clustering within development | Age; gender; education level; marital status; children at home; baseline minutes of recreational, transportation, or total walking; self-selection factors for choice of new neighborhood | *Changes in transport-related neighborhood walking*  Livable neighborhood:  **T1-T2**: -10.8, SE=2.8, p<0.05  **T2-T3**: 9.1, SE=3.8, p<0.05  T1-T3: -0.4, SE=4.0, p>0.05  Hybrid development:  T1-T2: -8.4, SE=3.2, p>0.05  T2-T3: 3.6, SE=4.3, p>0.05  T1-T3: -4.3, SE=4.6, p>0.05  Conventional development:  **T1-T2**: -8.3, SE=1.5, p<0.05  **T2-T3**: 6.9, SE=2.0, p<0.05  T1-T3: -1.4, SE=2.1, p>0.05  Livable vs. conventional neighborhood: p>0.05  *Changes in recreational neighborhood walking*  Livable neighborhood:  **T1-T2**: 16.6, SE=5.7, p<0.05  T2-T3: 9.3, SE=8.8, p>0.05  **T1-T3**: 26.3, SE=8.8, p<0.05  Hybrid development:  **T1-T2**: 16.0, SE=6.6, p<0.05  T2-T3: 10.2, SE=10.1, p>0.05  **T1-T3**: 30.9, SE=10.2, p<0.05  Conventional development:  **T1-T2**: 17.3, SE=3.0, p<0.05  T2-T3: 2.9, SE=4.5, p>0.05  **T1-T3**: 19.6, SE=4.2, p<0.05  Livable vs. conventional neighborhood: p>0.05  *Changes in total neighborhood walking*  Livable neighborhood:  T1-T2: 5.7, SE=6.4, p>0.05  **T2-T3**: 19.3, SE=9.5, p<0.05  **T1-T3**: 27.4, SE=9.3, p<0.05  Hybrid development:  **T1-T2**: 7.6, SE=7.4, p>0.05  T2-T3: 13.2, SE=11.0, p<0.05  T1-T3: 26.2, SE=10.8, p>0.05  Conventional development:  **T1-T2**: 9.0, SE=3.4, p<0.05  T2-T3: 9.6, SE=5.0, p>0.05  **T1-T3**: 18.1, SE=4.9, p<0.05  Livable vs. conventional neighborhood: p>0.05 |
| 19 | RESIDE,  Christian et al., 2017  [19] | N=1771 (urban)  Mean age 40 ± 12 years  Women: 59%  Response rate: 33%  Relocating adults  Perth, Australia | Natural experiment  Cluster: Purposive  Individuals: Two-stage approach; 1) households (census); 2) individuals (random)  Stratification: Relocating development type (Livable Neighborhood, hybrid, conventional)  Neighborhood definition: housing development (recruitment level) ; 1600m street network buffer and 15 min walk from home (participant level) | *Exposure variables – Objective*  1. Street connectivity  2. Residential density  3. Presence of small park within 400m service area  4. Presence of large park within 400m service area  5. Presence of regional/district park within 1600m service area  6. Presence of a beach point access within 1600m service area  7. Objective neighborhood index (combining all of the above), Density, Disaster mitigation, Destination proximity  *Measure:* Objective  [ArcGIS, GIS data base not reported]  *Validation:* Yes  *Exposure variables - Perceived*  8. Street connectivity  9. Infrastructure and safety for walking  10. Traffic safety  11. Neighborhood aesthetics  12. Crime safety  13. Presence of park within 5-min walk  14. Presence of sports field within 15-min walk  15. Presence of a beach within 15-min walk  16. Perceived neighborhood environment index  *Measure:* Perceived  [Neighborhood Environment Walkability Scale (NEWS)]  *Validation:* Yes | *Outcome:*  Frequency and minutes of recreational neighborhood walking  *Measure:* Self-report [Neighborhood Physical Activity Questionnaire (NPAQ)]  *Validity:* Yes | None | Negative binomial log-linear repeated measures regression models with generalized estimating equations (GEE) and robust standard errors | Age, gender, marital status,  education level, occupation, weekly hours of work, daily minutes  of work travel, physical activity level at work, children at home,  and dog ownership | *Frequency of neighborhood walking for recreation per week (relative change in frequency)*  Objective  1. Street connectivity: 0.98, 95%CI: 0.95;1.01  2. Residential density: 1.02, 95%CI: 0.98;1.06  3. Presence of small park within 400m service area  0.97; 95%CI: 0.91;1.04  4. Presence of large park within 400m service area  1.03; 95%CI: 0.96;1.11  5. Presence of regional/district park within 1600m service area: 0.96; 95%CI: 0.90; 1.04  6. Presence of a beach point access within 1600m service area: 0.95; 95%CI: 0.84;1.09  7. Objective neighborhood index: 0.98; 95%CI: 0.95;1.01  Perceived  8. **Street connectivity**: 1.12; 95%CI: 1.06;1.19  9. Infrastructure and safety for walking  1.03; 95%CI: 0.96;1.10  10. Traffic safety: 1.03; 95%CI: 0.89;1.09  11. **Neighborhood aesthetics:** 1.20; 95%CI: 1.14;1.26  12. **Crime safety:** 1.08; 95%CI: 1.02; 1.14  13. **Presence of park within 5-min walk**  1.12, 95%CI: 1.05;1.20  14. Presence of sports field within 15-min walk  1.04, 95%CI: 0.97;1.11  15. **Presence of a beach within 15-min walk**  1.14, 95%CI: 1.01;1.28  16. **Perceived neighborhood environment index**  1.12; 95%CI: 1.10;1.14  *Minutes of neighborhood walking for recreation per week (absolute change in minutes)*  Objective  1. Street connectivity: 0.44, 95%CI: -2.16;3.04  2. Residential density: 1.59, 95%CI: -2.28;5.46  3. **Presence of small park within 400m service area**  -6.24, 95%CI: -12.11;-0.37  4. Presence of large park within 400m service area  2.90; 95%CI: -3.10;8.91  5. Presence of regional/district park within 1600m service area: -4.95; 95%CI: -11.31;1.42  6. Presence of a beach point access within 1600m service area: -4.43; 95%CI: -18.20;9.32  7. Objective neighborhood index: -0.40, 95%CI: -2.72;1.92  Perceived  8. **Street connectivity:** 8.65, 95%CI: 3.99;13.30  9. Infrastructure and safety for walking  4.70, 95%CI: -0.98;10.38  10. Traffic safety: -0.23, 95%CI: -4.64;4.17  11. **Neighborhood aesthetics:** 10.44, 95%CI: 6.00;14.87  12. **Crime safety:** 7.49, 95%CI: 2.91;12.08  13. **Presence of park within 5-min walk**  9.52, 95%CI: 3.37;15.67  14. Presence of sports field within 15-min walk  2.72; 95%CI: -3.09;8.53  15. **Presence of a beach within 15-min walk**  21.51; 95%CI: 8.59, 34.43  16. **Perceived neighborhood environment index**  8.81, 95%CI: 7.12;10.50 |
| 20 | NA  Coughenour et al., 2019  [20] | N= 144 (urban)  57% 20-49 years  Women: 69%  Response rate: 6.5%  Community dwellers  Las Vegas, Nevada, USA | Cross-sectional  Cluster: purposive  Individuals: census  Stratification: walkability  Neighborhood definition: census block (recruitment level) | *Exposure variable*  1. Residential density  2. High retail-floor-area ratio  3. Street connectivity  4. Land-use mix  5. Distance between crosswalks  6. Single-entry communities  7. Tree shade  8. High-speed streets  9. Access to transit  *Measure:* Perceived  [Single items based upon the Walkability Index and Sprawl Characteristics]  *Validation:* Yes [was validated in the survey with correlating it with GIS-measures] | *Outcome*  Minutes of active transport per week  *Measure:*  [International Physical Activity Questionnaire (IPAQ)]  *Validation:* Yes | None | Poisson regression model without clustering | Age, gender, education, ethnicity | *Active transport minutes*  1. **Residential density**: B=0.14, p<0.05  2. **Retail-floor-area ratio**: B= -0.09, p<0.05  3. **Street connectivity**: B= -0.25, p<0.05  4. **Land-use mix**: B=0.09, p<0.05  5. **Distance between crosswalks**: B= -0.02, p<0.05  6. **Single-entry communities**: B=-0.08, p<0.05.  7. **Tree shade**: B=0.24, p<0.05  8. **High-speed streets**: B=-0.03, p<0.05  9. **Access to transit**: B=0.17, p<0.05 |
| 21 | NA  Dellasserra et al., 2018  [21] | N= 75 (mixed)  Mean age: 38 ± 9 years  Women: 65%  Response rate: NR  Mexican-American adults  Maricopa County, Arizona, USA | Cross-sectional  Cluster: none  Individuals: convenience  Stratification: none  Neighborhood definition: none | *Exposure variable*  Environmental barriers  *Measure*: Perceived  [Neighborhood Environment Walkability Scale (NEWS)]  *Validation:* Yes | *Outcome*  Total MVPA ≥ 150 min/week [Yes/No]  Leisure-time MVPA ≥ 150 min/week [Yes/No]  *Measure:* Self-report  [Stanford Brief Activity Survey (SBAS)]  *Validation:* Yes | None | Logistic regression | Age, gender, acculturation level, social support, exercise intention | *Total MVPA*  **Environmental barriers**: OR=4.39, p<0.05  *Leisure-time MVPA*  **Environmental barriers**: OR=5.56, p<0.05 |
| 22 | NA  Duncan et al., 2004  [22] | N= 1215 (urban)  66% 18-29 years  Women: 52%  Response rate: 46.6%  Community dwellers  Rockhampton, Australia | Cross-sectional  Cluster: none  Individuals: 1) households (random) 2), individuals (random)  Stratification: none  Neighborhood definition: 300-1500m Euclidian and network distance (participant) | *Exposure variable - Perceived*  1. Safe to walk  2. Dogs frighten people  3. Friendly neighborhood  4. High crime  5. Pleasant walks available  6. Shops/services in walking distance  7. See people on walks  8. Clean and tidy  9. Busy streets to cross when walking  10. Footpaths in good conditions  11. Heavy traffic  12. Safe to cycle  13. Streets well lit  14. Steep hills  15. Connectivity of parks (parks intersect with shops/streets)  *Measure: Perceived*  [15 items]  *Validation:* No  *Exposure variable - Objective*  16. Euclidian and network distance to nearest parkland  17. Euclidian and network distance to nearest shopping center  18. Euclidian and network distance to nearest 300m pathway network  19. Euclidian and network distance to nearest busy street  20. Euclidian and network distance to nearest newsagent  21. Total amount of road within 20m of a streetlight within 500m, 800m, 1000m, 1500m radii  22. Registered dogs within 500m, 800m, 1000m, 1500m radii  *Measure: Objective*  [GIS, Euclidian and street network distances 300-1500m, databases: geocoded places based upon telephone directory, electrical supplier, and dog registry, amongst other – not all databases reported]  *Validation:* Partially | *Outcome*  Sufficient physical activity [150min/week (Yes/No)]  Participation in recreational walking in the previous week [any recreational walking (Yes/No)  *Measure:* Self-report  [Active Australia Physical Activity Questionnaire]  *Validation:* Yes | None | Logistic regression | Age, income, gender, BMI, social support for physical activity, self-efficacy, number of sufficiently active people 500m-1500m radii, number of dogs 500-1500m radii | *Main effects sufficient physical activity*  Perceived  1. Safe to walk: NR, p>0.05  2. Dogs frighten people: NR, p>0.05  3. Friendly neighborhood: NR, p>0.05  4. High crime: NR, p>0.05  5. Pleasant walks available: NR, p>0.05  6. Shops/services in walking distance: NR, p>0.05  7. See people on walks: NR, p>0.05  8. **Clean and tidy**: OR = 3.13, 95%CI: 1.48;6.64  9. Busy streets to cross when walking: NR, p>0.05  10. Footpaths in good conditions: NR, p>0.05  11. Heavy traffic: NR, p>0.05  12. Safe to cycle: NR, p>0.05  13. Streets well lit: NR, p>0.05  14. Steep hills: NR, p>0.05  15. **Connectivity of parks**: OR=1.41, 95%CI: 1.01;1.98  Objective  16. **Network distance to nearest parkland**: OR=1.41, 95%CI: 1.01;1.97, p>0.05  17. Distance to nearest shopping center: NR, p>0.05  18. Distance to nearest 300m pathway network: NR, p>0.05  19. Distance to nearest busy street: NR, p>0.05  20. Distance to to nearest newsagent: NR, p>0.05  21. Total amount of road within 20m of a streetlight within 500m, 800m, 1000m, 1500m radii: NR  22. Registered dogs within 500m, 800m, 1000m, 1500m radii: NR, p>0.05  *Main effects any recreational walking*  Perceived  1. Safe to walk: NR, p>0.05  2. Dogs frighten people: NR, p>0.05  3. Friendly neighborhood: NR, p>0.05  4. High crime: NR, p>0.05  5. Pleasant walks available: NR, p>0.05  6. Shops/services in walking distance: NR, p>0.05  7. See people on walks: NR, p>0.05  8. Clean and tidy: NR, p>0.05  9. Busy streets to cross when walking: NR, p>0.05  10. **Footpaths in good conditions**: OR = 1.43, 95%CI: 1.02;1.99  11. Heavy traffic: NR, p>0.05  12. Safe to cycle: NR, p>0.05  13. Streets well lit: NR, p>0.05  14. Steep hills: NR, p>0.05  15. Connectivity of parks: NR, p>0.05  Objective  16. Distance to nearest parkland: NR, p>0.05  17. Distance to nearest shopping center: NR, p>0.05  18. **Euclidian distance to nearest 300m pathway network**: OR=0.31, 95%CI: 0.18;0.55  19. Distance to nearest busy street: NR, p>0.05  20. Network distance to nearest newsagent: OR=1.65, 95%CI: 1.05;2.58  21. Total amount of road within 20m of a streetlight within 500m, 800m, 1000m, 1500m radii: NR, p>0.05  22. Registered dogs within 500m, 800m, 1000m, 1500m radii: NR, p>0.05 |
| 23 | RESIDE  Foster et al., 2014a  [23] | N=1044 (urban)  Mean age 44 ± 12 years  Women: 62%  Response rate: 33%  Relocating adults  Perth, Australia | Cross-sectional  Cluster: Purposive  Individuals: Two-stage approach; 1) households (census); 2) individuals (random)  Stratification: Relocating development type (Livable Neighborhood, hybrid, conventional)  Neighborhood definition: housing development (recruitment level) ; 1600m street network buffer and 15 min walk from home (participant level) | *Exposure variables - Perceived*  1. Neighborhood maintenance  2. Social incivilities  3. Graffiti and vandalism  4. Property crime  5. Violent crime  6. Inadequate police service  7. Traffic noise  8. Vacant houses / blocks  9. Loitering teenagers  10. Dangerous or drunk driving  11. Many pedestrians (vs. few)  *Measure:* Perceived  *Validation:* No  *Exposure variables – Objective*  12. Personal crime  13. Burglary  14. Transport walkability index  15. Greenness  16. Park disorder (graffiti, litter, vandalism)  17. Park attractive amenities (lake, fountain, birds)  18. Park lighting  *Measure:* Objective  [ArcGIS and environmental audit, walkability index based upon Frank et al., 2005, NDVI for greenness, Broomhall Public Open Space Tool (POST) for audit; Database for GIS: Landsat TM satellite imagery; Western Australian Land Information Authority and Western Australian Department of Planning for road centerline and property cadaster data; commercial database (SENSIS Yellow Pages for local destination); crime data from the Western Australia Police]  *Validation:* Yes | *Outcome:*  Any transport-related and recreational walking [Yes/No]  *Measure:* Self-report [Neighborhood Physical Activity Questionnaire (NPAQ)]  *Validity:* Yes | None | Logistic regression with generalized estimating equations accounting for neighborhood clustering | Age, gender, education, household income, car ownership, length of residence, victimization, | *Any transport-related walking*  Perceived  1. Neighborhood maintenance: OR=0.94, p>0.05  2. Social incivilities: OR=0.83, p>0.05  3. Graffiti and vandalism: OR=1.20, p>0.05  4. Property crime: OR=1.06, p>0.05  5. Violent crime: OR=0.72, p>0.05  6. Inadequate police service: OR=1.05, p>0.05  7. Traffic noise: OR=0.78, p>0.05  8. Vacant houses / blocks: OR=0.95, p>0.05  9. Loitering teenagers: OR=0.86, p>0.05  10. Dangerous or drink driving: OR=0.89, p>0.05  11. Many pedestrians (vs. few): OR=1.37, p>0.05  Objective  12. Personal crime: OR=1.01-1.16, p>0.05  13. Burglary: OR=1.19-1.28, p>0.05  14. **Transport walkability index**: OR=1.05-1.17, p<0.05  15. Greenness: OR=1.05-1.17, p>0.05  *Any recreational walking*  Perceived  1. Neighborhood maintenance: OR=0.75, p>0.05  2. **Social incivilities**: OR=0.52, p<0.05  3. Graffiti and vandalism: OR=1.13, p>0.05  4. Property crime: OR=0.78, p>0.05  5. Violent crime: OR=0.71, p>0.05  6. Inadequate police service: OR=0.92, p>0.05  7. **Traffic noise**: OR=0.62, p<0.05  8. Vacant houses / blocks: OR=0.91, p>0.05  9. Loitering teenagers: OR=0.88, p>0.05  10. **Dangerous or drink driving**: OR=0.62, p<0.05  11. **Many pedestrians (vs. few)**: OR=1.72, p<0.05  Objective  12. Personal crime: OR=1.01-1.03, p>0.05  13. Burglary: OR=0.95-1.05, p>0.05  15. Greenness: OR=0.85-1.04, p>0.05  16. **Park disorder**: OR=0.72, p>0.05 (1 disorder form), OR=0.53-0.6, p<0.05 (2 and 3 disorder forms)  17**. Park attractive amenities**: OR=0.89, p>0.05 (1 amenity), OR=1.61-2.10, p<0.05, (2 and 3 amenities)  18. **Park lighting present**: OR=1.43, p<0.05 |
| 24 | RESIDE  Foster et al., 2014b  [24] | N=485 (urban)  Mean age 42 ± 12 years  Women: 61%  Response rate: 33%  Relocating adults  Perth, Australia | Longitudinal  Cluster: Purposive  Individuals: Two-stage approach; 1) households (census); 2) individuals (random)  Stratification: Relocating development type (Livable Neighborhood, hybrid, conventional)  Neighborhood definition: housing development (recruitment level) ; 1600m street network buffer and 15 min walk from home (participant level) | *Exposure variables – Objective changes in*  1. Land use mix  2. Street connectivity  3. Residential density  4. Number of bus stops  *Measure:* Objective  [GIS-measurement within 1600m street network service area; databases: see Foster et al., 2014a]  *Validation:* Yes  *Exposure variables – Perceived changes in*  5. Traffic noise  6. Dangerous or drink driving  7. Social incivilities  8. Graffiti and vandalism  9. Neighborhood maintenance  10. Fear of crime  *Measure:* Perceived  *Validation:* No | *Outcome:*  Changes in recreational, transport-related, and total neighborhood walking [min/week] between T1 and T2  *Measure:* Self-report [Neighborhood Physical Activity Questionnaire (NPAQ)]  *Validity:* Yes | None | Mixed linear regression models accounting for neighborhood clustering | Age, gender, education, household income, car ownership, length of residence, baseline walking levels, clustering within development | *Changes in transport-related neighborhood walking*  Objective  1. Land use mix: -3.65, p>0.05  2. Street connectivity: 0.09, p>0.05  3. Residential density: 0.12, p>0.05  4. Number of bus stops: 0.10, p>0.05  Perceived  5. Traffic noise: -5.60, p>0.05  6. Dangerous or drink driving: -3.06, p>0.05  7. Social incivilities: -2.97, p>0.05  8. Graffiti and vandalism: -1.04, p>0.05  9. Neighborhood maintenance: 2.23, p>0.05  10. **Fear of crime**: -7.86, p<0.05  *Changes in recreational neighborhood walking*  Objective  1. Land use mix: 0.01, p>0.05  2. Street connectivity: 0.05, p>0.05  3. Residential density: 2.40, p>0.05  4. Number of bus stops: -0.02, p>0.05  Perceived  5. Traffic noise: 8.92, p>0.05  6. Dangerous or drink driving: 2.75, p>0.05  7. Social incivilities: 11.32, p>0.05  8. Graffiti and vandalism: 0.91, p>0.05  9. Neighborhood maintenance: 6.30, p>0.05  10. Fear of crime: -10.96, p>0.05  *Changes in total neighborhood walking*  Objective  1. Land use mix: -14.64, p>0.05  2. Street connectivity: 0.16, p>0.05  3. Residential density: 2.25, p>0.05  4. Number of bus stops: -0.13, p>0.05  Perceived  5. Traffic noise: 2.37, p>0.05  6. Dangerous or drink driving: 1.62, p>0.05  7. Social incivilities: 3.47, p>0.05  8. Graffiti and vandalism: -0.54, p>0.05  9. Neighborhood maintenance: 748, p>0.05  10. **Fear of crime**: -19.91, p<0.05 |
| 25 | RESIDE  Foster et al., 2016  [25] | N=1831 (urban)  Mean age 40 ± 12 years  Women: 60%  Response rate: 33%  Relocating adults  Perth, Australia | Longitudinal  Cluster: Purposive  Individuals: Two-stage approach; 1) households (census); 2) individuals (random)  Stratification: Relocating development type (Livable Neighborhood, hybrid, conventional)  Neighborhood definition: housing development (recruitment level) ; 1600m street network buffer and 15 min walk from home (participant level) | *Exposure variable - Perceived*  1. Perceived safety from crime  *Measure:* Perceived  [Modified version of the Neighborhood Environment Walkability Scale (NEWS)]  *Validation:* Yes  *Exposure variable - Objective*  2. Personal crimes reported to the police  *Measure:* Objective  [Crime data from the Western Australia Police]  *Validation:* Yes | *Outcome:*  Total, recreational, and transport-related neighborhood walking  [min/week]  *Measure:* Self-report [Neighborhood Physical Activity Questionnaire (NPAQ)]  *Validity:* Yes | Gender, age group, education | Mixed marginal model with unrestricted variance patterns across time points | Age, gender, education, household income, marital status, importance of safety from crime (self-selection), time, built environment, and neighborhood perceptions | *Total neighborhood walking*  1. **Perceived safety from crime**: 10.54, p<0.05  *Recreational neighborhood walking*  1. **Perceived safety from crime**: 7.01, p<0.05  *Transport-related neighborhood walking*  1. Perceived safety from crime: 0.68, p>0.05  *Total neighborhood walking*  2. Personal crimes reported to the police: -0.30, p>0.05  *Recreational neighborhood walking*  2. Personal crimes reported to the police: 0.00, p>0.05  *Transport-related walking inside the neighborhood*  2. Personal crimes reported to the police: 0.04, p>0.05  *No effect moderation observed (NR)* |
| 26 | NA  García-Pérez et al., 2021  [26] | N= 1285 (urban)  73% 25-44 years  Women: 100%  Response rate: 92%  Community dwellers  Hermosillo, Mexico | Cross-sectional  Cluster: random  Individuals: random  Stratification: none  Neighborhood definition: urban blocks (recruitment and participant level) | *Exposure variable*  1. Number of neighborhood parks  2. Park-to-people ratio  3. Distance to nearest park from residence  4. Combined park-service area  *Measure:* Objective  [GIS, Euclidian distance, Hermosillo’s Cadastral Office and Parks Department]  *Validation:* Yes | *Outcome*  Neighborhood-based leisure-time sport/exercise [walking/running (Yes/No)]  Total leisure-time sport/exercise [regular sport/exercise engagement (Yes/No)]  *Measure:* Self-report  [Kaiser physical activity survey]  *Validation:* Yes | None | Two-level logistic regression with random intercept for neighborhoods | Age, gender, individual and neighborhood socio-economic status, education | *Neighborhood-based leisure-time sport/exercise*  1. Number of neighborhood parks: OR=1.00, p>0.05  2. Park-to-people ratio: OR=0.93, p>0.05  3. Distance to nearest park from residence (>750m ref.):  <350m: OR=1.35, p>0.05  350-750m: OR=1.49, p>0.05  4. Combined park-service area: OR=1.00, 95 p>0.05  *Total leisure-time sport/exercise*  1. Number of neighborhood parks: p>0.05  2. Park-to-people ratio: p>0.05  3. Distance to nearest park from residence (>750m ref.):  <350m: OR=1.22, p>0.05  350-750m: OR=1.36, p>0.05  4. Combined park-service area: OR=0.99, p>0.05 |
| 27 | SEID (Study on Environmental and Individual Determinants of Physical Activity);  Giles-Corti et al., 2002  [27] | N= 1773 (urban)  69% 18-29 years  Women: 57%  Response rate: 53%  Community dwellers  Perth, Australia | Cross-sectional  Cluster: purposive  Individuals: random  Stratification: socio-economic status  Neighborhood definition: collector districts (recruitment) and shortest distance (individual) | *Exposure variable*  1. Functional environment (footpaths and shops)  2. Appeal of environment (street design and trees)  3. Access to built facilities  4. Access to natural facilities  *Measure:* Objective  [GIS, network distance, Perth Metropolitan Area street-network database ad MapInfo]  *Validation:* Yes | *Outcome*  Exercising as recommended [30min+ of the equivalent of moderate activity on most days of the week (Yes/No)]  *Measure:* Self-report  [modified physical activity items used in previous Australian studies]  *Validation:* No | None | Logistic regression analysis | Age, gender, number of children<18 years  in the household, work outside the home, household income, education | *Exercising as recommended*  1. Functional environment (ref.: no footpaths and shops): OR=0.82-3.50, p>0.05  2. Appeal of environment (ref: major traffic and no trees)  OR=0.96-1.18, p>0.05  3. Access to built facilities (ref.: top quartile of access):  OR=0.73-0.85, p>0.05  4. Access to natural facilities (ref.: top quartile of access):  OR=0.83-0.86, p>0.05 |
| 28 | SEID (Study on Environmental and Individual Determinants of Physical Activity);  Giles-Corti et al., 2005  [28] | N= 1773 (urban)  Equal age groups across 18-29, 30-39, and 40-49 years  Women: 68%  Response rate: 53%  Community dwellers  Perth, Australia | Cross-sectional  Cluster: purposive  Individuals: random  Stratification: socio-economic status  Neighborhood definition: collector districts (recruitment) | *Exposure variable*  1. Access to public open space (distance, attractiveness, and size)  *Measure:* Objective  [GIS, network distance, Perth Metropolitan Area street-network database and MapInfo]  *Validation:* Yes | *Outcome*  Sufficient physical activity [30min+ of the equivalent of moderate activity on most days of the week (Yes/No)]  Walking as recommended [>=5 walking sessions with a total of 150min/week+; (Yes/No)]  High levels of walking [>=6 walking sessions with a total of 180min/week+; (Yes/No)]  *Measure:* Self-report  [modified physical activity items used in previous Australian studies]  *Validation:* No | None | Logistic regression analysis | Age, gender, number of children<18 years  in the household, education, socioeconomic status of area of residence | *Sufficient physical activity*  1. Access to open space (ref. very poor access)  OR=0.73-0.91; 95%CI: 0.55;1.20  *Walking as recommended*  1. Access to open space (ref. very poor access)  OR=0.68-1.24; 95%CI: 0.48;1.70  *High levels of walking*  1. Access to open space (ref. very poor access)  **Very good access**: OR=1.50, 95%CI: 1.06;2.13  Poor-good access: OR=0.73-1.11; 95%CI: 0.50;1.59 |
| 29 | RESIDE,  Giles-Corti et al., 2013  [29] | N=1420 (urban)  Mean age 41 ± 12 years  Women: 62%  Response rate: 35%  Relocating adults  Perth, Australia | Natural experiment  Cluster: Purposive  Individuals: Two-stage approach; 1) households (census); 2) individuals (random)  Stratification: Relocating development type (Livable Neighborhood, hybrid, conventional)  Neighborhood definition: housing development (recruitment level) ; 1600m street network buffer and 15 min walk from home (participant level) | *Exposure variables – Decreases / increases / no change from T1 to T2 in perceptions regarding…*  1. Access to mixed services  2. Neighborhood aesthetics  3. Infrastructure and safety for walking  4. Traffic hazards  5. Crime safety  6. Hilly streets  7. Major barriers present  8. Lack of cul-de-sacs  9. Street connectivity  10. Many alternative routes  11. Slow traffic speed  12. Traffic-slowing devices present  13. Local park / nature reserve accessible  14. Footpaths on most streets  15. Number of transport-related neighborhood perceptions that changed favorably from T1 to T2  16. Number of recreation-related neighborhood  perceptions that changed favorably from T1 to T2  *Measure:* Perceived  [Neighborhood Environment Walkability Scale (NEWS)]  *Validation:* Yes  *Exposure variables – Decreases / increases / no change from T1 to T2 in objective measures regarding*  17. Number of objectively measured transport-related destination types that increased from T1 to T2  18. Number of objectively measured recreational destination types that increased from T1 to T2  *Measure:* Objective  [ArcGIS, 800m and 1600m street-network buffer, destinations sourced from SENSIS Pty. Ltd]  *Validation: Yes* | *Outcome:*  Changes in total weekly minutes of neighborhood recreational and transport-related walking  *Measure:* Self-report [Neighborhood Physical Activity Questionnaire (NPAQ)]  *Validity:* Yes | None | Generalized linear mixed models – univariate and multivariate analysis accounting for clustering of developments | Univariate analysis:  Age, gender, education, marital status, children<18, baseline recreational and transport-related walking, intrapersonal change (6 variables), social environment change (4 variables), and self-selection (21 items)  In addition for transport-related models: changes in work status, number of work hours/week, time to travel to work  In addition for recreational models: changes in education  Multivariate analysis:  Age, gender, education, marital status, children<18, baseline recreational and transport-related walking, intrapersonal change (6 variables), social environment change (4 variables), and self-selection (21 items) | *Univariate analysis environment and* *transport-related walking changes*  Perceived  1. **Access to mixed services: p<0.05**  Decrease: -11.9  No change: -4.8  Increase: -2.0  2. Neighborhood aesthetics: p>0.05  Decrease: -10.6  No change: -5.8  Increase: -7.9  3. **Infrastructure and safety for walking: p<0.05**  Decrease: -11.8  No change: -10.5  Increase: -5.1  4. Traffic safety, p>0.05  Decrease: -6.5  No change: -11.1  Increase: -7.9  5. Crime safety, p>0.05  Decrease: -8.2  No change: -8.0  Increase: -8.9  6. Hilly streets make it difficult to walk, p>0.05  Decrease: -10.4  No change: -7.8  Increase: -8.5  7. Major barriers present, p>0.05  Decrease: -9.2  No change: -7.7  Increase:-9.6  8. Lack of cul-de-sacs, p=0.05  Decrease: -8.7  No change: -12.5  Increase: -5.7  9. Street connectivity, p>0.05  Decrease: -9.4  No change: -8.8  Increase: -7.7  10. Many alternative routes, p>0.05  Decrease: -13.0  No change: -7.4  Increase: -7.9  11. Slow traffic speed, p>0.05  Decrease: -5.7  No change: -9.2  Increase: -9.2  12. Traffic-slowing devices present, p>0.05  Decrease: -10.2  No change: -6.5  Increase: NA  13. Local park / nature reserve accessible, p>0.05  Decrease: -13.0  No change: -7.9  Increase: -7.5  14. Footpaths on most streets, p>0.05  Decrease: -12.8  No change: -6.5  Increase: -9.6  15**. Number of transport-related neighborhood perceptions that changed favorably**: 3.5, p<0.05  16. Number of recreation-related neighbourhood  perceptions that changed favourably: 0.8, p>0.05  Objective  17. **Number of objectively measured transport-related destination types that increased**: 7.0, p<0.05  18. Number of objectively measured recreational destination types that increased: -3.0, p>0.05  *Multivariate model regarding* *transport-related walking changes*  15. **Number of objectively measured transport-related destination types that increased**: B = 5.8, p <0.05  17. **Number of transport-related neighbourhood**  **perceptions that changed favourably**: 3.0, p<0.05  *Univariate analysis environment and* *recreation-related walking changes*  Perceived  1. **Access to mixed services**, p<0.05  Decrease: 11.2  No change: 12.5  Increase: 28.5  2. **Neighborhood aesthetics**, p<0.05  Decrease: 2.8  No change: 21.6  Increase: 21.3  3. Infrastructure and safety for walking, p>0.05  Decrease: 11.7  No change: 10.2  Increase: 20.7  4. **Traffic safety**, p<0.05  Decrease: 10.7  No change: 7.9  Increase: 23.2  5. Crime safety, p>0.05  Decrease: 4.8  No change: 14.0  Increase: 19.5  6. Hilly streets make it difficult to walk, p>0.05  Decrease: 6.5  No change: 15.7  Increase: 20.1  7. **Major barriers present**, p<0.05  Decrease: 20.4  No change: 18.9  Increase: 2.8  8. **Lack of cul-de-sacs**, p<0.05  Decrease: 2.1  No change: 26.9  Increase: 12.9  9. Street connectivity, p>0.05  Decrease: 15.7  No change: 9.3  Increase: 23.1  10. **Many alternative routes**, p<0.05  Decrease: -3.4  No change: 15.1  Increase: 28.9  11. **Slow traffic speed**, p<0.05  Decrease: 9.6  No change: 10.0  Increase: 26.9  12. Traffic-slowing devices present, p>0.05  Decrease: 9.1  No change: 17.6  Increase: NA  13. **Local park / nature reserve accessible**, p<0.05  Decrease: -4.1  No change: 16.9  Increase: 22.6  14. **Footpaths on most streets**, p<0.05  Decrease: 3.8  No change: 13.4  Increase: 25.4  15. **Number of transport-related neighborhood perceptions that changed favorably**: 6.1, p<0.05  16. **Number of recreation-related neighborhood**  **perceptions that changed favorably**: 4.5, p<0.05  Objective  17. Number of objectively measured transport-related destination types that increased: B = -2.9, p>0.05  18. **Number of objectively measured recreational destination types that increased**: B=-22.1, p<0.05  *Multivariate model regarding recreational walking changes*  16. Number of objectively measured transport-related destination types that increased: 17.6, p>0.05  18. **Number of transport-related neighborhood**  **perceptions that changed favorably**: 2.2, p<0.05 |
| 30 | GCs-PAS (Gated communities physical activity study),  Gul et al., 2018a  [30] | N= 1042 (urban)  67% 18-40 years  Women: 33%  Response rate: 89%  Community dwellers  Karachi, Pakistan | Cross-sectional  Cluster: purposive  Individuals: random  Stratification: multi/single family and gated/non-gated community  Neighborhood definition: neighborhood boundaries | *Exposure variable*  Number of exercise facilities  *Measure:* Objective  [Aerial pictures and ground observation, exercise facilities within 500-1000m^2^ in community]  *Validation:* Yes | *Outcome*  Vigorous physical activity [0 min/week; <600 MET-min/week; >600 MET-min/week]  *Measure:* Self-report [International Physical Activity Questionnaire (IPAQ)]  *Validity:* Yes | Gated and non-gated communities | Cross-tabulation and Gamma-test | None | ***% of total sample engaging in >600 MET-min/week (gated + non-gated)***  1-3 exercise facilities: 13.4%  3-6 exercise facilities: 20.3%  6-10 exercise facilities: 42.3%  p<0.05  ***% of sample in gated communities engaging in >600 MET-min/week***  1-3 exercise facilities: 14.8%  3-6 exercise facilities: 27.0%  6-10 exercise facilities: 52.0%  p<0.05  ***% of sample in non-gated communities engaging in >600 MET-min/week***  1-3 exercise facilities: 10.7%  3-6 exercise facilities: 15.2%  6-10 exercise facilities: 27.7%  p<0.05 |
| 31 | GCs-PAS (Gated communities physical activity study),  Gul et al., 2018b  [31] | N= 1042 (urban)  67% 18-40 years  Women: 33%  Response rate: 89%  Community dwellers  Karachi, Pakistan | Cross-sectional  Cluster: purposive  Individuals: random  Stratification: multi/single family and gated/non-gated community  Neighborhood definition: neighborhood boundaries | *Exposure variable*  Perception of crime (POC)  *Measure:* Perceived  [Neighborhood Environment Walkabilty Scale (NEWS)]  *Validation:* Yes | *Outcome*  Walking [0 min/week; <600 MET-min/week; >600 MET-min/week]  *Measure:* Self-report [International Physical Activity Questionnaire (IPAQ)]  *Validity:* Yes | Gated and non-gated communities | Cross-tabulation, and gamma tests | None | ***% of total sample engaging in >600 walking MET-min/week (gated + non-gated)***  Very low crime perception: 18.1%  Low crime perception: 21.9%  High crime perception: 12.6%  Very high crime perception: 21.2%  p<0.05  ***% of sample in gated communities engaging in >600 MET-min/week***  Very low crime perception: 6.4%  Low crime perception: 23.0%  High crime perception: 5.8%  Very high crime perception: 18.2%  p<0.05  *% of sample in non-gated communities engaging in >600 MET-min/week*  Very low crime perception: 29.8%  Low crime perception: 19.6%  High crime perception: 17.9%  Very high crime perception: 23.1%  p>0.05 |
| 32 | GCs-PAS (Gated communities physical activity study),  Gul et al., 2020  [32] | N= 1042 (urban)  67% 18-40 years  Women: 33%  Response rate: 89%  Community dwellers  Karachi, Pakistan | Cross-sectional  Cluster: purposive  Individuals: random  Stratification: multi/single family and gated/non-gated community  Neighborhood definition: neighborhood boundaries | *Exposure variable*  Street connectivity  *Measure:* Objective  [GIS, Space Syntax method, database: street centerlines from ArcGIS]]  *Validation:* Yes | *Outcome*  Total walking [0 min/week; <600 MET-min/week; >600 MET-min/week]  *Measure:* Self-report [Neighborhood Environment Physical Activity Questionnaire (NPAQ)]  *Validity:* Yes | None | Cross-tabulation and Gamma-test | None | ***% of total sample engaging in >600 MET-min/week***:  Lowest connectivity: 12.0%  Low connectivity: 18.9%  High connectivity: 29.2%  Highest connectivity: 23.8%  p<0.05 |
| 33 | GCs-PAS (Gated communities physical activity study),  Gul et al., 2021  [33] | N= 1042 (urban)  67% 18-40 years  Women: 33%  Response rate: 89%  Community dwellers  Karachi, Pakistan | Cross-sectional  Cluster: purposive  Individuals: random  Stratification: multi/single family and gated/non-gated community  Neighborhood definition: neighborhood boundaries | *Exposure variable - Objective*  1. Land-use mix  2. Street connectivity  3. Housing density  4. Exercise facilities  *Measure:* Objective  [GIS and ground observation, database: street centerlines from ArcGIS, aerial maps of development authorities]  *Validation:* Yes  *Exposure variable - Perceived*  5. Crime perception  6. Traffic hazard perception  *Measure:* Perceived  [4 items]  *Validation:* No | *Outcome*  Moderate physical activity  Vigorous physical activity  Transport-related walking  Recreational walking  Aggregate physical activity [<600 MET-min/week; >600 MET-min/week; (Yes/No)]  *Measure:* Self-report [Neighborhood Environment Physical Activity Questionnaire (NPAQ) and International Physical Activity Questionnaire (IPAQ-SF)]  *Validity:* Yes | None | Partial correlation and logistic regression | Correlation analysis:  Age, gender, employment status  Logistic regression:  Age, gender, employment status, gated/non-gated community | *Correlation with vigorous physical activity*  Objective  1. Land-use mix: r = -0.01, p>0.05  2. Street connectivity: r = 0.06, p>0.05  3. Housing density: r = 0.06, p>0.05  4. **Exercise facilities**: r = 0.18, p<0.05  Perceived  5. **Crime perception**: r = -0.11, p<0.05  6. Traffic hazard perception: r = -0.05, p>0.05  *Correlation results moderate physical activity*  Objective  1. Land-use mix: r = -0.01, p>0.05  2. Street connectivity: r = 0.00, p>0.05  3. Housing density: r = 0.01, p>0.05  4. Exercise facilities: r = 0.04, p>0.05  Perceived  5. Crime perception: r = 0.01, p>0.05  6. Traffic hazard perception: r = -0.02, p>0.05  *Correlation with transport-related walking*  Objective  1. **Land-use mix**: r = 0.18, <0.05  2. **Street connectivity**: r = 0.14, p<0.05  3. Housing density: r = -0.08, p>0.05  4. **Exercise facilities**: r = 0.19, p<0.05  Perceived  5. Crime perception: r = -0.04, p>0.05  6. **Traffic hazard perception**: r = -0.08, p<0.05  *Correlation with recreational walking*  Objective  1. Land-use mix: r = 0.02, p>0.05  2. **Street connectivity**: r = 0.08, p<0.05  3. **Housing density**: r = -0.14, p<0.05  4. **Exercise facilities**: r = 0.09, p<0.05  Perceived  5. Crime perception: r = -0.03, p>0.05  6. Traffic hazard perception: r = 0.03, p>0.05  *Binary logistic regression for aggregate physical activity*  Objective  1. **Land-use mix**: OR = 1.32, p<0.05  2. Street connectivity: OR = 1.23, p>0.05  3. **Housing density**: OR = 0.99, p<0.05  4. **Exercise facilities**: OR = 1.96, p<0.05  Perceived  5. Crime perception: OR = 0.96, p>0.05  6. Traffic hazard perception: OR = 0.91, p>0.05 |
| 34 | NA  Hailemariam et al., 2020  [34] | N= 299 (mixed)  50% 16-24 years  Women: 100 %  Response rate: NR  Pregnant women  Mekelle, Ethiopia | Cross-sectional  Cluster: health facilities  Individuals: random  Stratification: none  Neighborhood definition: none | *Exposure variable*  Travel time to health facility  *Measure:* Self-report  [NA]  *Validation:* No | *Outcome*  Being active [NR]  *Measure:* Self-report  [Pregnancy Physical Activity Questionnaire (PPAQ)]  *Validation:* Yes | None | Logistic regression without clustering | None | *Being inactive*  **Travel time to health facility** (>60 min): OR = 0.31, 95%CI: 0.11;0.89 |
| 35 | Household travel survey  Handy, 1996  [35] | N= 1385 (urban)  Medium age: 44 years  Women: 45%  Response rate: 25%  Community dwellers  Austin, Texas, USA | Cross-sectional  Cluster: neighborhood  Individuals: two-stage: 1) households - random, 2) individuals - random  Stratification: traditional, early, modern neighborhoods  Neighborhood definition: neighborhood boundaries (recruitment) | *Exposure variable*  Neighborhood  1. Safety during walking at night  2. See other people  3 Trees provide shade  4. Comfortable walking when it’s hot  5. Interesting houses to look at  6. Like seeing other people when I walk  7. Comfortable walking on sidewalks  8. Like to look at interesting houses  9. Often see people I don’t know  10. Too much traffic  Shopping environment  11. Stores withing walking distance  12. Feel safe walking to shopping areas  13. High quality of local stores  14. Local stores meet needs  15. Hard to park at local stores  16. Have to cross busy street  17. Have to walk along busy street  *Measure:* Self-report  [16 items]  *Validation:* No | *Outcome*  Strolling frequency around the neighborhood  Frequency of walks to store  *Measure:* Self-report  [NA]  *Validation:* No | None | Correlation | None | *Strolling frequency in the neighborhood*   1. Safety during walking at night: r = 0.16, p >0.05 2. See other people: r = 0.16, p >0.05 3. Trees provide shade: r = 0.14, p >0.05 4. Comfortable walking when it’s hot: r = 0.14, p >0.05 5. Interesting houses to look at: r = 0.12, p >0.05 6. Like seeing other people when I walk: r = 0.12, p >0.05 7. Comfortable walking on sidewalks: 0.12, p >0.05 8. Like to look at interesting houses: 0.05, p >0.05 9. Often see people I don’t know: -0.01, p >0.05 10. Too much traffic: -0.01, p >0.05   *Walking to the store*   1. Stores withing walking distance: r = 0.32, p >0.05 2. Feel safe walking to shopping areas: r = 0.25, p >0.05 3. High quality of local stores: r = 0.22, p >0.05 4. Local stores meet needs: r = 0.21, p >0.05 5. Hard to park at local stores: r = -0.03, p >0.05 6. **Have to cross busy street**: r = -0.06, p <0.05 7. Have to walk along busy street: r = -0.10, p >0.05 |
| 36 | Household travel survey  Handy, 2001  [36] | N= 1385 (urban)  Median age: 44 years  Women: 45%  Response rate: 25%  Community dwellers  Austin, Texas, USA | Cross-sectional  Cluster: neighborhood  Individuals: two-stage: 1) households - random, 2) individuals - random  Stratification: traditional, early, modern neighborhoods  Neighborhood definition: neighborhood boundaries | *Exposure variable - Perceived*  1. Stores (high quality, meet needs)  2. Walking incentive (within walking distance, hard to park)  3. Walking comfort (safety and street traffic),  *Measure:* Perceived  [8 items]  *Validation:* No  *Exposure variable - Objective*  4. Miles to store  Measure: Objective  [TRANPLAN network skim function, database not reported]  *Validation*: Yes | *Outcome*  Frequency of walks to store  *Measure:* Self-report  [NA]  *Validation:* No | None | Linear regression models without accounting for clustering | Age, work status, kids<5 years, gender, income, strolling frequency, old West Austin, three urban form factors | *Walking to store frequency*  Perceived  1. **Stores**: B = 0.76, p<0.05  2. **Walking incentive**: B = 0.54, p<0.05  3. **Walking comfort**: B=0.91, p<0.05  Objective  4*.* **Miles to store**: -3.81, p<0.05 |
| 37 | Cameron County Latino Cohort;  Heredia et al., 2022  [37] | N= 495 (mixed)  50% 18-39 years  Women: 70%  Response rate:  Community dwellers  Cameron County, Texas, USA | Cross-sectional  Cluster: purposive  Individuals: random  Stratification: socio-economic status  Neighborhood definition: census tract | *Exposure variable*  1. Unfriendly pedestrians  2. Crime  3. Stray dogs  4. Lack of shops within walking distance  5. No bus stop within 15 min walking  6. No recreation facilities (free / low cost)  7. No streetlights  8. High traffic  9. No one out doing physical activity  *Measure:* Perceived  [Neighborhood Physical Activity Environment Scale (NEWS)]  *Validation:* Yes | *Outcome*  Meeting the physical activity guidelines [Yes/No]  *Measure:* Self-report  [International Physical Activity Questionnaire (IPAQ) and Godin Leisure Time Questionnaire]  *Validation:* Yes | None | Logistic regression without controlling for clustering (but tested for variance at census block level) | Gender, age, marital status, education, insurance | *Meeting physical activity guidelines*  1. Unfriendly pedestrians: OR=0.79, p>0.05  2. Crime: OR=0.76, p>0.05  3. Stray dogs: OR=1.09, p>0.05  4. **Lack of shops**: OR=0.57, p<0.05  5. No bus stop: OR=0.95, p>0.05  6. No recreation facilities: OR=0.83  7. No streetlights: OR=0.76, p>0.05  8. High traffic: OR=0.84, p>0.05  9. **No one out doing physical activity**: OR=0.53, p<0.05 |
| 38 | NA  Holt et al., 2016  [38] | N= 200 (urban)  Mean age 73 ± 5 years  Women: 74%  Response rate: 80%  Retirement village residents  Perth, Australia | Cross-sectional  Cluster: random  Individuals: random  Stratification: none  Neighborhood definition: retirement village | *Exposure variable - Perceived*  1. Lack of good places to walk to  2. Fear of crime or strangers  3. Traffic  4. Hills  5. Unsafe streets  *Measure:* Perceived  [5 items]  *Validation:* Yes  *Exposure variable - Objective*  6. Outside supportive physical activity features  7. Outside aesthetics  8. Outside hazards  9. Outside exercise facilities  10. Outside features  *Measure:* Objective  [Audit of Physical Activity Resources for Seniors]  *Validation:* Yes | *Outcome*  Weekly physical activity program attendance [Yes/No]  *Measure:* Self-report  [NR]  *Validation:* No | None | Logistic regression without accounting for clustering | Retirement village age, retirement village size, age, gender, education level, marital status | *Physical activity program attendance*  Perceived  1. Lack of good places to walk: OR = NR, p>0.05  2. Fear of crime or strangers: OR = NR, p>0.05  3. Traffic: OR = NR, p>0.05  4. Hills: OR = NR, p>0.05  5. Unsafe streets: OR = NR, p>0.05  Objective  6. Outside supportive physical activity features: OR = NR, p>0.05  7. Outside aesthetics: OR = NR, p>0.05  8. Outside hazards: OR = NR, p>0.05  9. Outside exercise facilities: OR = NR, p>0.05  10. Outside features: OR = NR, p>0.05 |
| 39 | RESIDE,  Hooper et al., 2014  [39] | N=594 (urban)  Mean age 43 ± 12 years  Women: 63%  Response rate: 33%  Relocating adults  Perth, Australia | Cross-sectional  Cluster: Purposive  Individuals: Two-stage approach; 1) households (census); 2) individuals (random)  Stratification: Relocating development type (Livable Neighborhood, hybrid, conventional)  Neighborhood definition: housing development (recruitment level) ; 1600m street network buffer and 15 min walk from home (participant level) | *Exposure variables*  Compliance with livable neighborhood design (LNG) policies  1. Community design  2. Movement networks  3. Lot layout  4. Public parkland  5. All policies  *Measure:* Objective  [GIS, various databases, see Foster et al., 2014a]  *Validation:* Yes | *Outcome:*  Any recreational neighborhood walking [Yes/No]  Recreational neighborhood walking ≥ 60 min [Yes/No]  Any transport-related neighborhood walking [Yes/No]  Transport-related neighborhood walking ≥ 60 min [Yes/No]  *Measure:* Self-report [Neighborhood Physical Activity Questionnaire (NPAQ)]  *Validity:* Yes | None | Logistic regression with generalized estimating equations accounting for clustering within developments | Age; gender; education level; children at home; stage of construction, development size, self-selection | *Association between 10% increase in percentage compliance score with LGN and any transport-related neighborhood walking*  1. **Community design**: OR=1.27, p<0.05  2. **Movement networks**: OR=2.49, p<0.05  3. **Lot layout**: OR=1.26, p<0.05  4. Public parkland: OR=0.78, p>0.05  5. **Total:** OR=1.53, p<0.05  *Association between 10% increase in percentage compliance score with LGN and any recreational neighborhood walking*  1. Community design: OR=1.03, p>0.05  2. Movement networks: OR=1.49, p>0.05  3. Lot layout: OR=1.06, p>0.05  4. Public parkland: OR=0.85, p>0.05  5. Total: OR=0.97, p>0.05  *Association between 10% increase in percentage compliance score with LGN and ≥60 min transport-related neighborhood walking*  1. **Community design**: OR=1.17, p<0.05  2. Movement networks: OR=2.01, p>0.05  3. Lot layout: OR=1.02 p>0.05  4. Public parkland: OR=0.76, p>0.05  5. Total: OR=1.14, p>0.05  *Association between 10% increase in percentage compliance score with LGN and ≥60 min recreational neighborhood walking*  1. Community design: OR=1.01, p>0.05  2. Movement networks: OR=1.35, p>0.05  3. Lot layout: OR=0.98, p>0.05  4. Public parkland: OR=0.75, p>0.05  5. Total: OR=0.97, p>0.05  *Association between cluster types and any transport-related walking with poor compliance for all parameters as reference category*  1. Higher movement network + lot layout compliance: OR=1.54, p>0.05  2. Higher public parkland compliance: OR=1.43, p>0.05  3. **Higher community design compliance**: OR = 2.64, p<0.05  *Association between cluster types and any recreational walking with poor compliance for all parameters as reference category*  1. **Higher movement network + lot layout compliance**: OR=1.74, p<0.05  2. **Higher public parkland compliance**: OR=3.53, p<0.05  3. Higher community design compliance: OR = 0.96, p<0.05  *Association between cluster types and ≥60 min transport-related walking with poor compliance for all parameters as reference category*  1. Higher movement network + lot layout compliance: OR=1.29, p>0.05  2. Higher public parkland compliance: OR=0.83, p>0.05  3. **Higher community design compliance**: OR = 1.98, p<0.05  *Association between cluster types and ≥60 min recreational walking with poor compliance for all parameters as reference category*  1. **Higher movement network + lot layout compliance**: OR=2.05, p<0.05  2. **Higher public parkland compliance**: OR=3.37, p<0.05  3. Higher community design compliance: OR = 0.96, p<0.05 |
| 40 | RESIDE,  Hooper et al., 2015a  [40] | N=664 (urban)  Mean age 43 ± 12 years  Women: 62%  Response rate: 33%  Relocating adults  Perth, Australia | Cross-sectional  Cluster: Purposive  Individuals: Two-stage approach; 1) households (census); 2) individuals (random)  Stratification: Relocating development type (Livable Neighborhood, hybrid, conventional)  Neighborhood definition: housing development (recruitment level); 1600m street network buffer and 15 min walk from home (participant level) | *Exposure variables*  Design clusters  1. Disconnected developments (poor community design and poor public parkland)  2. Connected and compact developments  3. Green Developments (best public parkland)  4. Livable Developments (best community design features)  *Measure:* Objective  [GIS, various databases]  *Validation:* Yes | *Outcome:*  Any recreational neighborhood walking [Yes/No]  Recreational neighborhood walking ≥ 60 min [Yes/No]  Any transport-related neighborhood walking [Yes/No]  Transport-related neighborhood walking ≥ 60 min [Yes/No]  Any total walking [Yes/No]  Total walking ≥ 60 min [Yes/No]  Total walking ≥ 150 min [Yes/No]  *Measure:* Self-report [Neighborhood Physical Activity Questionnaire (NPAQ)]  *Validity:* Yes | None | Cluster analysis and logistic regression with generalized estimating equations accounting for clustering | Age; gender; education level; children at home; stage of construction, development size, self-selection | *Association between cluster and any transport-related walking with disconnected developments as reference category*  2. Connected and compact developments: OR=NR, p>0.05  3. Green Developments: OR=NR, p>0.05  4. **Livable Developments**: OR=2.64; 95%CI: 1.38;5.06  *Association between cluster and transport-related walking ≥ 60 min with disconnected developments as reference category*  2. Connected and compact developments: OR=NR, p>0.05  3. Green Developments: OR=NR, p>0.05  4. **Livable Developments**: OR=1.98, 95%CI: 1.09;3.61  *Association between cluster and any recreational walking with disconnected developments as reference category*  2. **Connected and compact developments**: OR=1.74, 95%CI: 1.22;2.48  3. **Green Developments**: OR=3.53, 95%CI: 2.02;6.17  4. Livable Developments: OR=NR, p>0.05  *Association between cluster and recreational walking ≥ 60 min with disconnected developments as reference category*  2. **Connected and compact developments**:  OR=2.05, 95%CI: 1.46;2.88  3. **Green Developments**: OR=3.37, 95%CI: 1.98;5.74  4. Livable Developments: OR=NR, p>0.05  *Association between cluster and any total walking with disconnected developments as reference category*  2. Connected and compact developments: OR=NR, p>0.05  3. **Green Developments**: OR=2.35, 95%CI: 1.36;4.09  4. **Livable Developments**: OR=1.71, 95%CI: 1.44;2.03  *Association between cluster and total walking ≥ 60 min with disconnected developments as reference category*  2. Connected and compact developments: OR=NR, p>0.05  3. Green Developments: OR=NR, p>0.05  4. **Livable Developments**: OR=1.77, 95%CI: 1.14;2.76  *Association between cluster and total walking ≥ 150 min with disconnected developments as reference category*  2. Connected and compact developments: OR=NR, p>0.05  3. Green Developments: OR=NR, p>0.05  4. **Livable Developments**: OR=1.47, 95%CI: 1.15;1.86 |
| 41 | RESIDE,  Hooper et al., 2015b  [41] | N=664 (urban)  Mean age 43 ± 12 years  Women: 62%  Response rate: 33%  Relocating adults  Perth, Australia | Cross-sectional  Cluster: Purposive  Individuals: Two-stage approach; 1) households (census); 2) individuals (random)  Stratification: Relocating development type (Livable Neighborhood, hybrid, conventional)  Neighborhood definition: housing development (recruitment level) ; 1600m street network buffer and 15 min walk from home (participant level) | *Exposure variables*  Community design  1. Neighborhood center configuration  2. Center destination diversity  3. Primary school <400m  Movement network  4. Block density  5. Walkable block ratio  6. Cul-de-sacs link ratio  7. Number of external access points  8. Footpath length  9. Sidewalk road-ratio  10. Tree density along footpaths  Lot layout  11. %residential land area occupied by small lots  Public parkland:  12. Medium neighborhood park <400m  13. Number of parks  14. Regional park <2.5 km  15. Number of small neighborhood parks  16. Number of medium neighborhood parks  17. Number of parks with sports surfaces, marking or equipment  Variables considered but not presented since not significant for any physical activity outcome  Community design  *Access to neighborhood centers*   - Distance to the nearest neighborhood/town center - Centre accessible within 400m - Centre accessible within 800m - Centre accessible within 1600m   *Public transport access*   - Distance to nearest bus-stop - Bus stop accessible within 400m - Number of bus routes through development - Number of bus services to/from the development - Distance to nearest train station - Train station accessible within 800m - Distance to nearest primary school   *Primary school access*   - Primary school accessible within 1600m   Movement network  *Street network connectivity*   - Connected node ratio - Mean block parameter   *Cul-de-sac provision and design*   - Cul-de-sac length ratio - Cul-de-sac lot ratio - % of residential lots on cul de sacs - Cul-de-sac street %   *Total footpath provision*   - Footpath to road ratio - % of road length with sidewalks   *Streetscape*   - Tree canopy cover   Lot layout, Diversity  *Residential lot size*   - Mean residential lot size - Median residential lot size - Number of different lot sizes present - Residential area occupied by different lot sizes   *Housing diversity development*   - Number of dwelling types - Residential land area occupied by different dwelling types   Public parkland:  *Amount and type of parks*   - Area of all parks + publicly accessible school grounds - % provision of parks - Number of district parks - Number of regional parks   *Park access* and accessibility   - Any park accessible within 400m - Local park accessible within 200m - Small neighborhood park accessible within 400m - Medium neighborhood park accessible within 400m - Large neighborhood parks accessible within 400m - District park accessible within 600m-1km   *Park surveillance and safety*   - Park perimeter frontage ratio - Park perimeter roads ratio   *Measure:* Objective  [GIS, 1600m street-network buffers, various databases see Foster et al., 2014a]  *Validation:* Yes | *Outcome:*  Any recreational neighborhood walking [Yes/No]  Recreational neighborhood walking ≥ 60 min [Yes/No]  Any transport-related neighborhood walking [Yes/No]  Transport-related neighborhood walking ≥ 60 min [Yes/No]  Any total walking [Yes/No]  Total walking ≥ 60 min [Yes/No]  Total walking ≥ 150 min [Yes/No]  *Measure:* Self-report [Neighborhood Physical Activity Questionnaire (NPAQ)]  *Validity:* Yes | Interactions between neighborhood characteristics | Logistic regression with generalized estimating equations accounting for clustering | Age; gender; education level; children at home; stage of construction, development size, self-selection | *Association between characteristics and any transport-related walking*  Community design  1. **Neighborhood center configuration (ref. none)**:  Big box: OR=1.38, p>0.05  Main street: OR=2.10, p<0.05  2. **Center destination diversity**: OR=1.22, p<0.05  3. **Primary school <400m**: OR=0.55, p<0.05  Movement network  4. Block density: OR=NR, p>0.05  5. Walkable block ratio: OR=NR, p>0.05  6. Cul-de-sacs link ratio: OR=NR, p>0.05  7. **Number of external access points**: OR=1.35, p<0.05  8. **Footpath length**: OR=1.02, p<0.05  9. Sidewalk road ratio: OR=NR, p>0.05  10. **Tree density along footpaths**: OR=1.04, p<0.05  Lot layout  11. **%residential land area occupied by small lots**: OR=1.04, p<0.05  Public parkland:  12. Medium neighborhood park <400m: OR=NR, p>0.05  13. **Number of parks**: OR=1.08, p<0.05  14. **Regional park** <2.5 km: OR=3.97, p<0.05  15. **Number of small neighborhood parks**: OR=1.13, p<0.05  16. **Number of medium neighborhood parks**: OR=1.17, p<0.05  17. Number of parks with sports surfaces, marking or equipment: OR=NR, p>0.05  *Association between characteristics and ≥60 min transport-related walking*  Community design  1. **Neighborhood center configuration**:  Big box: OR=1.68, p<0.05  Main street: OR=1.70, p<0.05  2. **Center destination diversity**: OR=1.36, p<0.05  3. Primary school <400m: OR=NR, p>0.05  Movement network  4. Block density: OR=NR, p>0.05  5. Walkable block ratio: OR=NR, p>0.05  6. **Cul-de-sacs link ratio >50%:** OR=2.40, p<0.05  7. Number of external access points: OR=NR, p>0.05  8. **Footpath length**: OR=1.02, p<0.05  9. Sidewalk road ratio: OR=NR, p>0.05  10. Tree density along footpaths: OR=NR, p>0.05  Lot layout  11. %residential land area occupied by small lots: OR=NR, p>0.05  Public parkland:  12. **Medium neighborhood park <400m**: OR=1.09, p<0.05  13. Number of parks: OR=NR, p>0.05  14. **Regional park <2.5 km**: OR= 1.99, p<0.05  15. Number of small neighborhood parks: OR=NR, p>0.05  16. Number of medium neighborhood parks: OR=NR, p>0.05  17. **Number of parks with sports surfaces, marking or equipment**: OR = 1.26, p<0.05  *Association between characteristics and any recreational walking*  Community design  1. **Neighborhood center configuration**:  Big box: OR=1.07, p>0.05  Main street: OR=2.28, p<0.05  2. Center destination diversity: OR=NR, p>0.05  3. Primary school <400m: OR=NR, p>0.05  Movement network  4. **Block density**: OR=6.83, p<0.05  5. Walkable block ratio: OR=NR, p>0.05  6. Cul-de-sacs link ratio >50: OR=NR, p>0.05  7. Number of external access points: OR=NR, p>0.05  8. Footpath length: OR=NR, p>0.05  9. Sidewalk road ratio: OR=NR, p>0.05  10. Tree density along footpaths: OR=NR, p>0.05  Lot layout  11. %residential land area occupied by small lots: OR=NR, p>0.05  Public parkland:  12. Medium neighborhood park <400m: OR=NR, p>0.05  13. Number of parks: OR=NR, p>0.05  14. **Regional park** <2.5 km: OR=1.63, p<0.05  15. Number of small neighborhood parks: OR=NR, p>0.05  16. **Number of medium neighborhood parks**: OR=1.09, p<0.05  17. Number of parks with sports surfaces, marking or equipment:  OR=NR, p>0.05  *Association between characteristics and recreational walking ≥60 min*  Community design  1. **Neighborhood center configuration**:  Big box: OR=0.95, p>0.05  Main street: OR=2.37, p<0.05  2. Center destination diversity: OR=NR, p>0.05  3. Primary school <400m: OR=NR, p>0.05  Movement network  4. **Block density**: OR=5.14, p<0.05  5. Walkable block ratio: OR=NR, p>0.05  6. **Cul-de-sacs link ratio >50%:** OR=0.64, p<0.05  7. Number of external access points: OR=NR, p>0.05  8. **Footpath length**: OR=1.01, p<0.05  9. Sidewalk road ratio:  10. Tree density along footpaths: OR=NR, p>0.05  Lot layout  11. %residential land area occupied by small lots: OR=NR, p>0.05  Public parkland:  12. Medium neighborhood park <400m: OR=NR, p>0.05  13. Number of parks: OR=NR, p>0.05  14. **Regional park** <2.5 km: OR=1.82, p<0.05  15. Number of small neighborhood parks: OR=NR, p>0.05  16. **Number of medium neighborhood parks**: OR=1.08, p<0.05  17. Number of parks with sports surfaces, marking or equipment: OR=NR, p>0.05  *Association between characteristics and any total neighborhood walking*  Community design  1. **Neighborhood center configuration**:  Big box: OR=3.10, p<0.05  Main street: OR=5.51, p<0.05  2. Center destination diversity: OR=NR, p>0.05  3. Primary school <400m: OR=NR, p>0.05  Movement network  4. Block density: OR=NR, p>0.05  5. **Walkable block ratio**: OR=4.38, p<0.05  6. Cul-de-sacs link ratio: OR=NR, p>0.05  7. Number of external access points: OR=NR, p>0.05  8. Footpath length: OR=NR, p>0.05  9. Sidewalk road ratio: OR=NR, p>0.05  10. Tree density along footpaths: OR=NR, p>0.05  Lot layout  11. %residential land area occupied by small lots: OR=NR, p>0.05  Public parkland:  12. Medium neighborhood park <400m: OR=NR, p>0.05  13. Number of parks: OR=NR, p>0.05  14. **Regional park <2.5 km**: OR=1.58, p<0.05  15. Number of small neighborhood parks: OR=NR, p>0.05  16. **Number of medium neighborhood parks**: OR=1.06, p<0.05  17. Number of parks with sports surfaces, marking or equipment: OR=NR, p>0.05  *Association between characteristics and total neighborhood walking ≥60 min*  Community design  1. **Neighborhood center configuration**:  Big box: OR=1.44, p<0.05  Main street: OR=6.65, p<0.05  2. Center destination diversity: OR=NR, p>0.05  3. Primary school <400m: OR=NR, p>0.05  Movement network  4. **Block density:** OR=5.05, p<0.05  5. Walkable block ratio: OR=NR, p>0.05  6. Cul-de-sacs link ratio: OR=NR, p>0.05  7. Number of external access points: OR=NR, p>0.05  8. Footpath length: OR=NR, p>0.05  9. **Sidewalk road ratio**: OR=3.14, p<0.05  10. **Tree density along footpaths**: OR=1.02, p<0.05  Lot layout  11. %residential land area occupied by small lots: OR=NR, p>0.05  Public parkland:  12. Medium neighborhood park <400m: OR=NR, p>0.05  13. Number of parks: OR=NR, p>0.05  14. **Regional park <2.5 km**: OR=1.85, p<0.05  15. Number of small neighborhood parks: OR=NR, p>0.05  16. **Number of medium neighborhood parks**: OR=1.06, p<0.05  17. Number of parks with sports surfaces, marking or equipment: OR=NR, p>0.05  *Association between characteristics and total neighborhood walking ≥150 min*  Community design  1. Neighborhood center configuration: OR=NR, p>0.05  2. **Center destination diversity**: OR=1.16, p<0.05  3. **Primary school <400m:** OR=0.18, p<0.05  Movement network  4. Block density: OR=NR, p>0.05  5. Walkable block ratio: OR=2.27, p<0.05  6. **Cul-de-sacs link ratio >50%:** OR=3.24, p<0.05  7. Number of external access points: OR=NR, p>0.05  8. Footpath length: OR=NR, p>0.05  9. Sidewalk road ratio: OR=NR, p>0.05  10. Tree density along footpaths: OR=NR, p>0.05  Lot layout  11. %residential land area occupied by small lots: OR=NR, p>0.05  Public parkland:  12. Medium neighborhood park <400m: OR=NR, p>0.05  13. Number of parks: OR=NR, p>0.05  14. Regional park <2.5 km: OR=NR, p>0.05  15. Number of small neighborhood parks: OR=NR, p>0.05  16. Number of medium neighborhood parks: OR=NR, p>0.05  17. Number of parks with sports surfaces, marking or equipment: OR=NR, p>0.05  *Interaction – association with transport-related walking ≥60 min*  18. Configuration of center 1600m (main street/big box) * connected node ratio (high/low): OR=1.98-9.59, p>0.05  19. Configuration of center 1600m (no center/main street/big box) * cul-de-sac % (high/low): OR=2.14-10.25, p>0.05  20. Configuration of center 1600m (main street/big box) * Sidewalk: Road ratio (high/low): OR=1.25-16.23, p>0.05  21. Footpath: Road ratio (High/low) * number of parks: OR=0.97-2.27, p>0.05  22. **Access to a large/regional park * footpath to road ratio (high/low)**: OR=0.27-2.24, p<0.05  *Interaction – association with recreational walking ≥60 min*  18. **Configuration of center 1600m (main street/big box) * connected node ratio (high/low**): OR=9.12-20.42, p<0.05  19. **Configuration of center 1600m (no center/main street/big box) * cul-de-sac % (high/low)**: OR=6.53-18.56, p<0.05  20. **Configuration of center 1600m (main street/big box) * Sidewalk: Road ratio (high/low):** OR=2.94-5.87, p<0.05  21. **Footpath: Road ratio (High/low) * number of parks**: OR=2.47, p<0.05  22. **Access to a large/regional park * footpath to road ratio (high/low)**: OR=0.35-2.04, p<0.05  *Interaction – association with total walking ≥60 min*  18. **Configuration of center 1600m (main street/big box) * connected node ratio (high/low):** OR=29.83-69.40, p<0.05  19. **Configuration of center 1600m (no center/main street/big box) * cul-de-sac % (high/low)**: OR=24.21-67.12, p<0.05  20. **Configuration of center 1600m (main street/big box) * Sidewalk: Road ratio (high/low)**: OR=6.09-12.04  21. Footpath: Road ratio (High/low) * number of parks: OR=0.97-2.30, p<0.05  22. **Access to a large/regional park * footpath to road ratio (high/low)**: OR=0.19-1.80, p<0.05  *Interaction – association with total walking ≥150 min*  18. **Configuration of center 1600m (main street/big box) * connected node ratio (high/low):** OR=3.23-6.03, p<0.05  19. Configuration of center 1600m (no center/main street/big box) * cul-de-sac % (high/low): OR=3.37-4.79, p<0.05  20. Configuration of center 1600m (main street/big box) * Sidewalk: Road ratio (high/low): OR=1.20-2.54, p<0.01  21. Footpath: Road ratio (High/low) * number of parks: OR=1.22-1.50, p<0.05  22. Access to a large/regional park * footpath to road ratio (high/low): OR=0.44-1.25, p<0.05  Variables considered not significant for any physical activity outcome  Community design  *Access to neighborhood centers*   - Distance to the nearest neighborhood/town center - Centre accessible within 400m - Centre accessible within 800m - Centre accessible within 1600m   *Public transport access*   - Distance to nearest bus-stop - Bus stop accessible within 400m - Number of bus routes through development - Number of bus services to/from the development - Distance to nearest train station - Train station accessible within 800m - Distance to nearest primary school   *Primary school access*   - Primary school accessible within 1600m   Movement network  *Street network connectivity*   - Connected node ratio - Mean block parameter   *Cul-de-sac provision and design*   - Cul-de-sac length ratio - Cul-de-sac lot ratio - % of residential lots on cul de sacs - Cul-de-sac street %   *Total footpath provision*   - Footpath to road ratio - % of road length with sidewalks   *Streetscape*   - Tree canopy cover   Lot layout, Diversity  *Residential lot size*   - Mean residential lot size - Median residential lot size - Number of different lot sizes present - Residential area occupied by different lot sizes   *Housing diversity development*   - Number of dwelling types - Residential land area occupied by different dwelling types   Public parkland:  *Amount and type of parks*   - Area of all parks + publicly accessible school grounds - % provision of parks - Number of district parks - Number of regional parks   *Park access* and accessibility   - Any park accessible within 400m - Local park accessible within 200m - Small neighborhood park accessible within 400m - Medium neighborhood park accessible within 400m - Large neighborhood parks accessible within 400m - District park accessible within 600m-1km   *Park surveillance and safety*   - Park perimeter frontage ratio - Park perimeter roads ratio |
| 42 | IPEN (International Physical Activity and Environment) - Mexico,  Jáuregui et al., 2016  [42] | N= 659 (urban)  Mean age 42 (95%CI: 40.7-43.2) years  Women: 51%  Response rate: NR  Community dwellers  Cuernavaca, Mexico | Cross-sectional  Cluster: purposive  Individuals: 1) households (random) 2) individuals (random)  Stratification: socio-economic status and walkability  Neighborhood definition: census-tract (recruitment) | *Exposure variable*  1. Safety from crime  2. Easy access to small parks  3. Pedestrian infrastructure  4. Land use mix diversity  5. Land use mix access  6. Safety from traffic  7. Few cul-de-sacs  8. Proximity to transit stops  9. Proximity to large parks  10. Aesthetics  *Other variables not included in the final model due to non-significance in single-variable models:*  11. Residential density  12. Street connectivity  13. No major physical activity barriers for walking  14. Barriers for walking  15. Hilliness  17. Park safety  18. Easy access to large parks  19. Proximity to small parks  *Measure:*  [Latin-American Neighborhood Environment Walkability Scale short version (NEWS)]  *Validation:* Yes | *Outcome*  Total minutes of moderate-to-vigorous physical activity (MVPA) per week  Any MVPA within bouts (≥10 minutes)  Minutes per week in MVPA within bouts (≥10 minutes)  *Measure:* Device-based  [7-day Actigraph GT3X]  *Validation:* Yes | Interaction between environmental variables and gender as well as socio-economic status | Two-part model and logit model accounting for multistage clustering | Age, gender, marital status, socio-economic status, motor vehicle ownership, educational attainment, accelerometer wear time | *Associations with total minutes of MVPA*  1. **Safety from crime**:  Women: B=0.92, p<0.05  **Men**: B=1.14, p>0.05  2. **Easy access to small parks**: B=1.16, p<0.05  3. Pedestrian infrastructure: B=0.97, p>0.05  4. Land use mix diversity: B=1.00, p>0.05  5. Land use mix access: B=0.86, p>0.05  6. Safety from traffic: B=1.12, p>0.05  7. **Few cul-de-sacs**: B=0.91, p<0.05  8. Proximity to transit stops: B=1.08, p>0.05  9. **Proximity to large parks**: B=1.12, p<0.05  10. **Aesthetics**:  **Low SES**: B=1.47, p<0.05  Medium SES: B=1.23, p>0.05  Medium-high SES: B=0.90, p>0.05  High SES: B=0.83, p>0.05  *Associations with any MVPA in bouts*  1. Safety from crime: B=1.12, p>0.05  2. Easy access to small parks: B=1.49, p>0.05  3. Pedestrian infrastructure: B=0.95, p>0.05  4. Land use mix diversity: B=0.95, p>0.05  5. Land use mix access: B=0.76, p>0.05  6. Safety from traffic: B=1.38, p>0.05  7. Few cul-de-sacs: B=NR, p>0.05  8. Proximity to transit stops: B=1.04, p>0.05  9. **Proximity to large parks**:  Women: B=1.69, p<0.05  Men: B=0.94, p>0.05  10. Aesthetics: B=0.99, p>0.05  *Associations with minutes per week in MVPA bouts*  1. **Safety from crime**:  Women: B=0.90, p>0.05  **Men**: B=1.29, p<0.05  2. Easy access to small parks: B=1.02, p>0.05  3. Pedestrian infrastructure: B=0.97, p>0.05  4. Land use mix diversity: B=0.91, p>0.05  5. Land use mix access: B=0.95, p>0.05  6. Safety from traffic: B=1.06, p>0.05  7. Few cul-de-sacs: B=NR, p>0.05  8. Proximity to transit stops: B=  9. **Proximity to large parks**: B=0.81, p<0.05  10. **Aesthetics**:  Low SES: B=1.16, p>0.05  Medium SES: B=1.37, p>0.05  Medium-high SES: B=0.74, p>0.05  High SES: B=0.81, p>0.05  *Other variables not included in the final model due to non-significance in single-variable models:*  11. Residential density: B=NR, p>0.05  12. Street connectivity: B=NR, p>0.05  13. No major physical activity barriers for walking: B=NR, p>0.05  14. Barriers for walking: B=NR, p>0.05  15. Hilliness: B=NR, p>0.05  17. Park safety: B=NR, p>0.05  18. Easy access to large parks: B=NR, p>0.05  19. Proximity to small parks: B=NR, p>0.05 |
| 43 | IPEN (International Physical Activity and Environment) - Mexico,  Jáuregui et al., 2017  [43] | N= 668 (urban)  Mean age 42 (95%CI: 40.7-43.2) years  Women: 51%  Response rate: NR  Community dwellers  Cuernavaca, Mexico | Cross-sectional  Cluster: purposive  Individuals: 1) households (random) 2) individuals (random)  Stratification: socio-economic status and walkability  Neighborhood definition: census-tract (recruitment) | *Exposure variable*  1. Aesthetics  2. Land-use mix diversity  3. Pedestrian infrastructure  4. Traffic safety  5. Safety from crime  6. Proximity to small parks  7. Park access  8. Proximity to large parks  9. Access to large parks  10. Proximity to transit stops  *Other variables not included in the final model due to non-significance in single-variable models:*  11. Residential density  12. Land use mix access  13. Street connectivity  14. Few cul-de-sacs  15. No major physical activity barriers for walking  16. Hilliness  17. Park safety  *Measure:*  [Latin-American Neighborhood Environment Walkability Scale short version (NEWS)]  *Validation:* Yes | *Outcome*  Participation in and duration of:  Leisure time walking (min/week)  Leisure-time moderate-to-vigorous (min/week)  Transport physical activity (min/week)  *Measure:* Self-report  [International Physical Activity Questionnaire (IPAQ)]  *Validation:* Yes | Interaction between environmental variables and gender as well as socio-economic status | Hurdle models accounting for clustering | Age, gender, marital status, socio-economic status, motor vehicle ownership, educational attainment | *Associations with participation in leisure walking*  1. **Aesthetics: Interaction with socio-economic status**  **Low SES**: B=2.23, p<0.05  Medium SES: B=1.53, p>0.05  Medium-high SES: B=1.27, p>0.05  High SES: B=0.89, p>0.05  2. Land-use mix diversity: B=1.04, p>0.05  3. Pedestrian infrastructure: B=0.95, p>0.05  4. Traffic safety: B=1.16, p>0.05  5. Safety from crime: B=1.02, p>0.05  6. Proximity to small parks: B=1.01, p>0.05  7. Park access: B=1.19, p>0.05  8. Proximity to large parks: B=0.99, p>0.05  9. Access to large parks: B=0.97, p>0.05  10. Proximity to transit stops: B=0.88, p>0.05  *Associations with leisure walking duration*  1. **Aesthetics**: B=1.92, p<0.05  2. Land-use mix diversity: B=1.08, p>0.05  3. Pedestrian infrastructure: B=1.00, p>0.05  4. **Traffic safety**: B=1.33, p<0.05  5. Safety from crime: B=1.02, p>0.05  6. Proximity to small parks: B=1.03, p>0.05  7. Park access: B=1.23, p>0.05  8. Proximity to large parks: B=0.98, p>0.05  9. Access to large parks: B=0.85, p>0.05  10. Proximity to transit stops: B=0.77, p>0.05  *Associations with participation in transport-related physical activity*  1. Aesthetics: B=1.23, p>0.05  2. Land-use mix diversity: B=0.86, p>0.05  3. Pedestrian infrastructure: B=0.84, p>0.05  4. Traffic safety: B=0.91, p>0.05  5. Safety from crime: B=1.06, p>0.05  6. Proximity to small parks: B=1.02, p>0.05  7. Park access: B=0.75, p>0.05  8. Proximity to large parks: B=0.78, p>0.05  9. **Access to large parks**: B=1.69, p>0.05  10. Proximity to transit stops: B=1.04, p>0.05  *Associations with transport-related physical activity duration*  1. Aesthetics: B=1.27, p>0.05  2. Land-use mix diversity: B=0.82, p>0.05  3. Pedestrian infrastructure: B=0.97, p>0.05  4. Traffic safety: B=1.15, p>0.05  5. Safety from crime: B=1.11, p>0.05  6. Proximity to small parks: B=1.09, p>0.05  7. Park access: B=0.82, p>0.05  8. Proximity to large parks: B=0.69, p>0.05  9. Access to large parks: B=1.41, p>0.05  10. Proximity to transit stops: B=0.98, p>0.05  *Associations with participation in leisure-time moderate-to-vigorous physical activity*  1. **Aesthetics**: B=1.33, p<0.05  2. Land-use mix diversity: B=0.97, p>0.05  3. Pedestrian infrastructure: B=0.79, p>0.05  4. Traffic safety: B=1.09, p>0.05  5. Safety from crime: B=1.02, p>0.05  6. Proximity to small parks: B=1.07, p>0.05  7. Park access: B=0.95, p>0.05  8. Proximity to large parks: B=1.03, p>0.05  9. Access to large parks: B=1.05, p>0.05  10. Proximity to transit stops: B=0.86, p>0.05  *Associations with duration of leisure-time moderate-to-vigorous physical activity*  1. **Aesthetics**: B=1.67, p<0.05  2. Land-use mix diversity: B=0.95, p>0.05  3. Pedestrian infrastructure: B=0.60, p>0.05  4. Traffic safety: B=1.26, p>0.05  5. Safety from crime: B=1.06, p>0.05  6. Proximity to small parks: B=1.04, p>0.05  7. Park access: B=0.88, p>0.05  8. Proximity to large parks: B=1.08, p>0.05  9. Access to large parks: B=1.02, p>0.05  10. Proximity to transit stops: B=0.69, p>0.05  *Variables that were not included in the final model due to non-significance*  11. Residential density: B=NR, p>0.05  12. Land use mix access street: B=NR, p>0.05  13. Street connectivity: B=NR, p>0.05  14. Few cul-de-sacs: B=NR, p>0.05  15. No major physical activity barriers for walking: B=NR, p>0.05  *Other interactions were not significant.* |
| 44 | Mexican-Adult-Survey,  Joseph et al. 2020  [44] | N= 75 (urban)  Mean age: 38 ± 9 years  Women: 65%  Response rate: NR  Mexican community dwellers  Phoenix, Arizona, USA | Cross-sectional  Cluster: none  Individuals: convenience  Stratification: none  Neighborhood definition: none | *Exposure variable*  1. Walking environment  2. Safety  3. Aesthetic quality  4. Violence  5. Availability of healthy foods  *Measure:* Perceived  [Neighborhood Scales Questionnaire]  *Validation:* Yes | *Outcome*  Participants in 5 activity categories [ranging from sedentary to active (150min+ min of moderate activity or 60min+ of vigorous activity per week)  *Measure:* Self-report  [Rapid physical activity questionnaire (RAPA)]  *Validation:* Yes | None | Linear regression | Age, gender, social cohesion, activities with neighbors | *Association with physical activity*  1. **Walking environment**: β=0.39, p<0.05  2. **Safety**: β=0.26, p<0.05  3. Aesthetic quality: β=0.18, p>0.05  4. Violence: β=-0.19, p>0.05  5. Availability of healthy foods: β=0.20, p>0.05 |
| 45 | NA  Joseph et al., 2021  [45] | N= 39 (urban)  Mean age: 41 ± 4 years  Women: 100%  Response rate: NR  First-generation Latinas  Phoenix, Arizona, USA | Cross-sectional  Cluster: none  Individuals: convenience  Stratification: none  Neighborhood definition: none | *Exposure variable*  1. Walking environment  2. Aesthetics quality  3. Safety  4. Violence  *Measure:* Self-report  [Neighborhood Scales Questionnaire]  *Validation:* Yes | *Outcome*  Leisure-time physical activity  Transport-related physical activity  Overall moderate-to-vigorous physical activity  (all MET-min/week)  Leisure time PA >0 MET min/week  Transport-related PA >0 MET min/week  Meeting the physical activity guidelines [≥ 500 MET min/week of moderate-to-vigorous physical activity]  (all Yes/No)  *Measure:* Self-report  [International Physical Activity Questionnaire (IPAQ)]  *Validation:* Yes | None | Ordinal least square regression and logistic regression | Age, education | *Association with transport-related physical activity (MET-min/week)*  1. Walking environment: B= -31.45, p>0.05  2. Aesthetics quality: B= -5.99, p>0.05  3. Safety: B=47.58, p>0.05  4. Violence: B= 20.30, p>0.05  *Association with leisure-time physical activity (MET-min/week)*  1. Walking environment: B=734.54, p>0.05  2. Aesthetics quality: B=558.27, p>0.05  3. Safety: B=724.93, p>0.05  4. Violence: B= -1201.72, p>0.05  *Association with MVPA (MET-min/week)*  1. Walking environment: B=1934.56, p>0.05  2. Aesthetics quality: B=1162.82, p>0.05  3. Safety: B=593.57, p>0.05  4. Violence: B= -1679.53, p>0.05  *Association with engagement in transport-related physical activity (MET-min/week)*  1. Walking environment: OR= 1.60, p>0.05  2. Aesthetics quality: OR= 0.83, p>0.05  3. Safety: OR= 1.22, p>0.05  4. Violence: OR= 1.17, p>0.05  *Association with engagement in leisure-time physical activity (MET-min/week)*  1. **Walking environment**: OR= 5.95, p<0.05  2. **Aesthetics quality**: OR= 2.45, p<0.05  3. **Safety**: OR= 3.30, p<0.05  4. Violence: OR= 0.38, p>0.05  *Association with achieving the physical activity guidelines (MET-min/week)*  1. **Walking environment**: OR= 4.15, p<0.05  2. **Aesthetics quality**: OR= 6.43, p<0.05  3. **Safety**: OR= 2.53, p<0.05  4. Violence: OR= 0.43, p>0.05 |
| 46 | NA  Khalaf et al., 2013  [46] | N= 663 (urban)  Mean age: 20 ± 2 years  Women: 100%  Response rate: 95%  University students  Saudi Arabia | Cross-sectional  Cluster: none  Individuals: convenience  Stratification: none  Neighborhood definition: none | *Exposure variable*  1. Proximity to malls  2. Proximity to parks  3. Proximity to supermarkets  *Measure:* Perceived  [3 items]  *Validation:* No | *Outcome*  Low, moderate, and high physical activity [tertiles based upon MET-minutes]  *Measure:* Self-report  [Arab Teens Lifestyle Questionnaire]  *Validation:* Yes | None | Multivariate ordinal regression | BMI, marital status, mother’s education level | *Association with physical activity*  1. Proximity to malls: OR=NR, p>0.05  2. **Proximity to parks** (ref. very close)  Kind of close: OR=1.00, p>0.05  **Far from home**: OR=1.86, p<0.05  3. Proximity to supermarkets: OR=NR, p>0.05 |
| 47 | RESIDE,  Knuiman et al., 2014  [47] | N=1703 (urban)  Mean age 40 ± 12 years  Women: 60%  Response rate: 33%  Relocating adults  Perth, Australia | Longitudinal  Cluster: Purposive  Individuals: Two-stage approach; 1) households (census); 2) individuals (random)  Stratification: Relocating development type (Livable Neighborhood, hybrid, conventional)  Neighborhood definition: housing development (recruitment level); 1600m street network buffer and 15 min walk from home (participant level) | *Exposure variable – Objective*  1. Connectivity  2. Residential density  3. Land use mix  4. Number of bus stops within 1600m  5. Railway station present within 1600m  6. Total number of type of destinations within 1600m  *Measure:* Objective  [ArcGIS, 1600m street-network buffer, various databases see Foster et al. 2014a]  *Validation:* Yes  *Exposure variable - Perceived*  7. Connectivity  8. Residential density  9. Land use mix  10. Perceived access to bus stop  11. Perceived access to railway station  12. Perceived total number of type of destinations  *Measure:* Objective  [Neighborhood Environment Physical Activity Scale (NEWS)]  *Validation:* Yes | *Outcome:*  Any transport-related neighborhood walking [Yes/No]  *Measure:* Self-report [Neighborhood Physical Activity Questionnaire (NPAQ)]  *Validity:* Yes | None | Logistic mixed effect model accounting for neighborhood clustering (separate models for objective and perceived measures) | Age, gender, education, occupation, work hours per week, household income, number of adults in household, children at home, motor vehicle access, time | *Association with transport-related neighborhood walking*  Objective  1. **Connectivity**: OR=1.13, p<0.05  2. **Residential density**: OR=0.96, p>0.05  3. **Land use mix**: OR=1.33, p<0.05  4. **Number of bus stops within 1600m** (ref. 0-14):  15-29: OR=1.99, p<0.05  ≥30: OR=2.33, p<0.05  5. **Railway station present within 1600m**: OR=1.79, p<0.05  6. Total number of type of destinations within 1600m (ref. 0-3):  4-7: OR=1.08, p>0.05  8-15: OR=1.40, p>0.05  Perceived  7. Connectivity: OR=1.07, p>0.05  8. Residential density: OR=0.97, p>0.05  9. **Land use mix**: OR=1.27, p<0.05  10. Perceived access to bus stop: OR=1.31, p>0.05  11**. Perceived access to railway station**: OR=1.80, p<0.05  12. **Perceived total number of type of destinations (ref. 0-2)**:  3-6: OR=2.35, p<0.05  7-11: OR=3.11, p<0.05 |
| 48 | RESIDE,  Learnihan et al., 2011  [48] | N=1753 (urban)  Mean age 40 ± 12 years  Women: 60%  Response rate: 33%  Relocating adults  Perth, Australia | Cross-sectional  Cluster: Purposive  Individuals: Two-stage approach; 1) households (census); 2) individuals (random)  Stratification: Relocating development type (Livable Neighborhood, hybrid, conventional)  Neighborhood definition: housing development (recruitment level); 1600m street network buffer and 15 min walk from home (participant level) | *Exposure variable – Objective*  1. Walkability at suburb scale  2. Walkability at census collection district scale  3. Walkability within 15min from home  *Measure:* Objective  [ArcGIS, 1600m street-network buffer, various databases see Foster et al. 2014a] | *Outcome:*  Transport-related and recreational neighborhood walking; 150min+ walking [Yes/No]  Minutes of transport-related, recreational, and total neighborhood walking  *Measure:* Self-report [Neighborhood Physical Activity Questionnaire (NPAQ)]  *Validity:* Yes | None | Logistic regression and analysis of variance: | Age, gender, education, household income | *Association with transport-related neighborhood walking*  1. **Walkability at suburb scale** (ref. low):  Medium low: OR=1.11, p>0.05  Medium high: OR=1.01, p>0.05  **High**: OR=1.63, p<0.05  2. **Walkability at census district scale (ref. low)**:  Medium low: OR=1.11, p>0.05  Medium high: OR=1.12, p>0.05  **High**: OR=2.07, p<0.05  3. **Walkability at 15-min walk level (ref. low):**  Medium low: OR=1.92, p<0.05  Medium high: OR=1.85, p<0.05  High: OR=2.79, p<0.05  *Association with recreational neighborhood walking*  1. **Walkability at suburb scale (ref. low**):  Medium low: OR=0.80, p>0.05  **Medium high**: OR=0.67, p<0.05  High: OR=0.92, p>0.05  2. **Walkability at census district scale (ref. low):**  Medium low: OR=1.31, p>0.05  Medium high: OR=1.05, p>0.05  **High**: OR=1.53, p<0.05  3. **Walkability at 15-min walk level** (ref. low):  Medium low: OR=1.25, p>0.05  Medium high: OR=1.07, p>0.05  **High**: OR=1.40, p<0.05  *Association with recreational neighborhood walking 150min+*  1. Walkability at suburb scale (ref. low):  Medium low: OR=0.90, p>0.05  Medium high: OR=0.75, p>0.05  High: OR=1.21, p>0.05  2. Walkability at census district scale (ref. low):  Medium low: OR=1.03, p>0.05  Medium high: OR=1.03, p>0.05  High: OR=1.21, p>0.05  3. **Walkability at 15-min walk level** (ref. low):  Medium low: OR=1.19, p>0.05  Medium high: OR=1.06, p>0.05  **High**: OR=1.43, p<0.05  *Association with transport neighborhood walking 150+ min*  1. Walkability at suburb scale (ref. low):  Medium low: OR=1.14, p>0.05  Medium high: OR=0.70, p>0.05  High: OR=1.78, p>0.05  2. **Walkability at census district scale (ref. low):**  Medium low: OR=1.63, p>0.05  Medium high: OR=2.00, p>0.05  **High**: OR=2.85, p<0.05  3. **Walkability at 15-min walk level (ref. low**):  Medium low: OR=0.83, p>0.05  Medium high: OR=1.72, p>0.05  **High**: OR=2.41, p<0.05  *Mean minutes total neighborhood walking*  1. Walkability at suburb scale: p>0.05  Low: 97  Medium low: 92  Medium high: 83  High: 102  2. Walkability at census district scale: p>0.05  Low: 86  Medium low: 99  Medium high: 88  High: 105  3. **Walkability at 15-min walk level:** p<0.05  Low: 85  Medium low: 90  Medium high: 91  High: 108  *Mean minutes transport neighborhood walking*  1. **Walkability at suburb scale**: p<0.05  Low: 23  Medium low: 25  Medium high: 24  High: 36  2. **Walkability at census district scale**: p<0.05  Low: 21  Medium low: 26  Medium high: 24  High: 37  3. **Walkability at 15-min walk level**: p<0.05  Low: 18  Medium low: 23  Medium high: 28  High: 38  *Mean minutes recreational neighborhood walking*  1. Walkability at suburb scale: p>0.05  Low: 74  Medium low: 67  Medium high: 59  High: 66  2. Walkability at census district scale: p>0.05  Low: 65  Medium low: 73  Medium high: 65  High: 68  3. Walkability at 15-min walk level: p>0.05  Low: 67  Medium low: 67  Medium high: 63  High: 71 |
| 49 | India travel survey,  Manoj & Verma, 2015  [49] | N= 1679 (urban)  Mean age: 38 years  Male/female ratio 0.91  Response rate: NR  Non-workers  Bangalore City, India | Cross-sectional  Cluster: none  Individuals: 1) households (random, 2) individuals  Stratification: none  Neighborhood definition: none | *Exposure variable - Objective*  1. % of mixed residential area  *Measure:* Objective  [ArcGIS, Land use data from Bangalore Development Authority]  *Validation:* Yes  *Exposure variable - Perceived*  2. Trip distance  Measure: Perceived  *Validation:* No | *Outcome*  Transport walking as mode choice [Yes/No]  *Measure:* Self-report  [NR]  *Validation:* No | Household income group | Binary logit models | Age, gender, income, vehicle ownership, activity is medical | *Association with walking as mode choice*  1. **% of mixed residential area**  **Low income**: 0.01, p>0.05  **Middle income**: 0.05, p<0.05  High income: 0.06, p<0.05  2. **Trip distance**:  Low income: -0.09, p<0.05  Middle income: -0.11, p<0.05  High income: -0.13, p<0.05 |
| 50 | India travel survey,  Manoj & Verma, 2016  [50] | N= 1679 (urban)  Mean age: 38 years  Male/female ratio 0.91  Response rate: NR  Non-workers  Bangalore City, India | Cross-sectional  Cluster: none  Individuals: random households  Stratification: none  Neighborhood definition: none | *Exposure variable - Objective*  1. Land use mix diversity  2. Population density  3. School enrolment  4. Adequate footpath available  *Measure:* Objective  [ArcGIS, Land use data from Bangalore Development Authority]  *Validation:* Yes  *Exposure variable - Perceived*  5. Trip distance  Measure: Perceived  *Validation:* No | *Outcome*  Walking as mode choice [Yes/No]  *Measure:* Self-report  [NR]  *Validation:* No | Vehicle ownership, school-going children | Binary logit models | Age, gender, income, vehicle ownership, activity is medical, trip distance | *Association with walking for shopping as mode choice*  Objective  1. Land use mix diversity: 0.05, p>0.05  2. Population density: 0.04, p>0.05  3. School enrolment: NR, p>0.05  4. Adequate footpath availability: 0.17, p>0.05  Perceived  5. **Trip distance**: -0.07, p<0.05  *Association with walking for recreation as mode choice*  Objective  1. Land use mix diversity: 0.03, p>0.05  2. Population density: NR, p>0.05  3. School enrolment: NR, p>0.05  4. Adequate footpath availability: NR, p>0.05  Perceived  5. **Trip distance**: -0.07, p<0.05  *Association with walking for personal business as mode choice*  Objective  1. **Land use mix diversity**: 0.09, p<0.05  2. Population density: 0.02, p>0.05  3. School enrolment: 0.04, p>0.05  4. Adequate footpath availability: 0.20, p>0.05  Perceived  5. **Trip distance**: -0.09, p<0.05  *Association with walking for vehicle-owning group*  1. Land use mix diversity: 0.03, p>0.05  2. Population density: 0.01, p>0.05  3. School enrolment: NR, p>0.05  4. Adequate footpath availability: 0.21, p>0.05  Perceived  5. Trip distance: -0.09, p>0.05  *Association with walking for non-vehicle-owning group*  1. Land use mix diversity: 0.05, p>0.05  2. Population density: 0.03, p>0.05  3. School enrolment: NR, p>0.05  4. Adequate footpath availability: 0.43, p>0.05  Perceived  5. Trip distance: -0.05, p>0.05  *Association with walking for households with school-going children*  1. Land use mix diversity: 0.06, p>0.05  2. Population density: NR, p>0.05  3. **School enrolment**: 0.06, p<0.05  4. Adequate footpath availability: NR, p>0.05  Perceived  5. Trip distance: -0.04, p>0.05  *Association with walking for households without school-going children*  1. Land use mix diversity: 0.04, p>0.05  2. Population density: NR, p>0.05  3. School enrolment: NR, p>0.05  4. Adequate footpath availability: 0.11, p>0.05  Perceived  5. Trip distance: -0.03, p>0.05 |
| 51 | RESIDE,  McCormack et al., 2012  [51] | N=1681 (urban)  Mean age 40 ± 12 years  Women: 59%  Response rate: 33%  Relocating adults  Perth, Australia | Cross-sectional  Cluster: Purposive  Individuals: Two-stage approach; 1) households (census); 2) individuals (random)  Stratification: Relocating development type (Livable Neighborhood, hybrid, conventional)  Neighborhood definition: housing development (recruitment level); 1600m street network buffer and 15 min walk from home (participant level) | *Exposure variable – Objective*  1. Walkability (street connectivity, land-use mix, residential density, sidewalk availability)  2. Sidewalk length  *Measure:* Objective  [ArcGIS, 1600m street-network buffer, various databases see Forster et al. 2014a]  *Validation:* Yes | *Outcome:*  Transport-related, recreational, and total neighborhood walking [Yes/No] inside the neighborhood  *Measure:* Self-report [Neighborhood Physical Activity Questionnaire (NPAQ)]  *Validity:* Yes | None | Multivariate probit regression followed by sample selection-bias corrected ordinary least square regression | Age, gender, education, household income, self-selection | *Association with transport neighborhood walking – probit-model*  1. **Walkability**: 2.16, p<0.05  2. **Sidewalk length**: 2.97, p<0.05  *Association with transport neighborhood walking minutes*  1. Walkability: B=2.26, p>0.05  2. Sidewalk length: B=5.38, p>0.05  *Association with recreational neighborhood walking – probit-model*  1. Walkability: 0.48, p>0.05  2. Sidewalk length: -0.47, p>0.05  *Association with recreational neighborhood walking minutes*  1. Walkability: -1.04, p>0.05  2. Sidewalk length: 1.64, p>0.05  *Association with total neighborhood walking – probit-model*  1. Walkability: 1.11, p>0.05  2. Sidewalk length: 0.66, p>0.05  *Association with total neighborhood walking minutes*  1. Walkability: B=1.13, p>0.05  2. Sidewalk length: B=5.26, p>0.05 |
| 52 | NA  Mehriar et al., 2021  [52] | N= 861 (urban)  48% 18-30 years  Women: 32%  Response rate: NR  Community dwellers  Lahore, Pakistan | Cross-sectional  Cluster: purposive  Individuals: NR  Stratification: sprawling characteristics  Neighborhood definition: district | *Exposure variable*  1. Street length density [>137 m/m^2^]  *Measure:*  [ArcGIS, 600m street-network buffer]  *Validation:* Yes | *Outcome*  Active mobility [Yes/No]  *Measure:* Self-report  [NR]  *Validation:* No | None | Area-under-the-curve analysis (ROC) and Youden Index | None | *Association with active mobility*  1. **Street length density**: Higher count for people with higher street density (results only displayed in Figure 4) |
| 53 | NA  Mohamed et al., 2021  [53] | N= 372 (mixed)  64% 50-69 years  Women: 100%  Response rate: 98%  Women with Diabetes Type 2  Riyadh, Saudi Arabia | Cross-sectional  Cluster: none  Individuals: random  Stratification: none  Neighborhood definition: none | *Exposure variable*  1. Park proximity  2. Traffic severity  3. Presence of sidewalks  4. Street lighting at night  5. Intersections close to each other  *Measure:* Perceived  [5 items]  *Validation:* No | *Outcome*  Meeting the physical activity guidelines [150min+ moderate-to-vigorous physical activity/week; (Yes/No)]  Physical activity minutes per week (MVPA + walking)  *Measure:* Self-report  [International Physical Activity Questionnaire (IPAQ)]  *Validation:* Yes | None | Chi square tests and linear regression | Age, education, income, marital status, diabetes duration, BMI, number of children, sedentary behavior, social support | *% of participants meeting the physical activity guidelines within each physical environment response option*  1. **Park proximity**: p<0.05  Very close: 41.2%  Kind of close: 39.2%  Far: 32.4%  2. **Traffic severity:** p<0.05  Light: 47.8%  Moderate: 41.9%  Heavy: 23.3%  3. **Presence of sidewalks**: p<0.05  Yes: 40.8%  No: 13%  4. Street lighting at night: p>0.05  Yes: 30.1%%  No: 28.9%  5. **Intersections close to each other**: p<0.05  Yes: 30.1%  No: 13.7%  *Association with physical activity minutes*  1. **Proximity to parks**: B=2.68, p<0.05  2. **Traffic severity**: B=-3.88, p<0.05  3. Presence of sidewalks: p>0.05  4. Street lighting at night: p>0.05  5. Intersections close to each other: p>0.05 |
| 54 | Health and Wellbeing Survey,  Nathan et al., 2012  [54] | N= 2918 (mixed)  62% 65-74 years  Women: 56%  Response rate: 80-84%  Older adults  Perth, Australia | Cross-sectional  Cluster: none  Individuals: 1) households, 2) individuals (random)  Stratification: urbanicity level and health region  Neighborhood definition: none (recruitment), 400m and 800m street-network buffer (participant level) | *Exposure variable*  1. Food retail access within 400m  2. General retail access within 400m  3. Medical care service access within 400m  4. Financial services access within 400m  5. General services access within 400m  6. Social infrastructure access within 400m  7. Destination mix within 400m  8. Food retail access within 800m  9. General retail access within 800m  10. Medical care service access within 800m  11. Financial services access within 800m  12. General services access within 800m  13. Social infrastructure access within 800m  14. Destination mix within 800m  *Measure:* Objective  [ArcGIS, 400m-800m street network buffer, database Sensis Pty. Ltd. / Australian Yellow Pages]  *Validation:* Yes | *Outcome*  Weekly walking [None/some]  Sufficient walking [150min+/week (Yes/No)]  *Measure:* Self-report  [Active Australia Survey]  *Validation:* No | Gender | Logistic regression | Age, gender, highest level of education, marital status, self-rated health, use of assistive equipment, street connectivity | *Association with some walking*  1. Food retail access within 400m: OR=1.01, p>0.05  2. General retail access within 400m: OR=0.95, p>0.05  3. Medical care service access within 400m: OR=0.91, p>0.05  4. Financial services access within 400m: OR=1.03, p>0.05  5. **General services access within 400m**: OR=1.29, p<0.05  6. Social infrastructure access within 400m: OR=0.98, p>0.05  7. Destination mix within 400m: OR=1.01, p>0.05  8. Food retail access within 800m: OR=0.94, p>0.05  9. General retail access within 800m: OR=0.97, p>0.05  10. Medical care service access within 800m: OR=0.96, p>0.05  11. Financial services access within 800m: OR=0.90, p>0.05  12. General services access within 800m: OR=1.16, p>0.05  13. Social infrastructure access within 800m: OR=1.15, p>0.05  14. Destination mix within 800m: OR=1.01, p>0.05  *Association with sufficient walking*  1. Food retail access within 400m: OR=0.88, p>0.05  2. General retail access within 400m: OR=0.82, p>0.05  3. **Medical care service access within 400m**: OR=0.77, p<0.05  4. Financial services access within 400m: OR=0.89, p>0.05  5. General services access within 400m: OR=1.02, p>0.05  6. Social infrastructure access within 400m: OR=1.04, p>0.05  7. Destination mix within 400m: OR=0.96, p>0.05  8. Food retail access within 800m: OR=0.88, p>0.05  9. General retail access within 800m: OR=0.91, p>0.05  10. **Medical care service access within 800m**: OR=0.83, p<0.05  11. Financial services access within 800m: OR=0.97, p>0.05  12. General services access within 800m: OR=1.07, p>0.05  13. Social infrastructure access within 800m: OR=0.90, p>0.05  14. Destination mix within 800m: OR=0.97, p>0.05  *Associations were not moderated by gender* |
| 55 | Retirement village study [name invented],  Nathan et al., 2014a  [55] | N= 323 (mixed)  Mean age: 77 ± 7 years  Women: 68%  Response rate: 40% and 60%  Retirement village residents  Perth and Peel region, Australia | Cross-sectional  Cluster: purposive  Individuals: random and convenience  Stratification: walkability  Neighborhood definition: retirement village boundaries (recruitment), retirement village boundaries and 400m service area (participants) | *Exposure variable - Perceived*  1. Recreational facilities  2. Services and facilities  3. Clubhouse  *Measure:* Perceived  [Reported by village manager]  *Validation:* No  *Exposure variable - Objective*  4. Walkability, Density, Diversity  *Measure:* Objective  [ArcGIS, 400m street-network buffer, database not reported]  *Validation:* No | *Outcome*  Accelerometer-assessed high light physical activity and moderate-to-vigorous physical activity [150min+/week (Yes/No)]  Self-reported weekly participation in leisure walking, transport-related walking, and brisk walking [Yes/No]  *Measure:* Device-based and self-report  [Actigraph GT1M and Community Healthy Activities Model Program for Seniors (CHAMPS) instrument]  *Validation:* Yes | None | Logistic regression with generalized estimating equations accounting for village clustering | Age, gender, education, marital status, employment status, self-rated health, car access, use of assistive devices, physical functioning, village characteristics | *Association with accelerometer assessed high light physical activity*  Perceived  1. Recreational facilities: OR=NR, p>0.05  2. Services and facilities: OR=NR, p>0.05  3. Clubhouse: OR=NR, p>0.05  Objective  4. Walkability: OR=NR, p>0.05  *Association with accelerometer assessed moderate-to-vigorous physical activity*  Perceived  1. Recreational facilities: OR=NR, p>0.05  2. Services and facilities: OR=NR, p>0.05  3. Clubhouse: OR=NR, p>0.05  Objective  4. Walkability: OR=NR, p>0.05  *Association with self-reported leisure-time walking*  Perceived  1. Recreational facilities: OR=NR, p>0.05  2. Services and facilities: OR=NR, p>0.05  3. **Clubhouse**: OR=1.52, p<0.05  Objective  4. Walkability: OR=NR, p>0.05  *Association with self-reported transport-related walking*  Perceived  1. Recreational facilities: OR=NR, p>0.05  2. Services and facilities: OR=NR, p>0.05  3. Clubhouse: OR=NR, p>0.05  Objective  4. **Walkability**: OR=1.19, p<0.05  *Association with self-reported brisk walking*  Perceived  1. Recreational facilities: OR=NR, p>0.05  2. Services and facilities: OR=NR, p>0.05  3. Clubhouse: OR=NR, p>0.05  Objective  4. Walkability: OR=NR, p>0.05 |
| 56 | Retirement village study [name invented],  Nathan et al., 2014b  [56] | N= 323 (mixed)  Mean age: 77 ± 7 years  Women: 68%  Response rate: 40% and 60%  Retirement village residents  Perth and Peel region, Australia | Cross-sectional  Cluster: purposive  Individuals: random and convenience  Stratification: walkability  Neighborhood definition: retirement village boundaries and 400m service area | *Exposure variable - Objective*  1. Distance to local shop outside village  2. Distance to public transport outside village  3. Distance to supermarket outside village  4. Traffic volume exposure outside village  *Measure:* Objective  [ArcGIS, 400m street-network buffer, database not reported]  *Validation:* No  *Exposure variable - Perceived*  5. Personal safety – inside village  6. Aesthetics – outside village  7. Aesthetics – inside village  8. Proximate destinations – outside village  *Measure:* Perceived  [Neighborhood Environment Walkability Scale (NEWS-A)]  *Validation:* Yes  *Variables eliminated prior to model inclusion due to non-significance*  Objective  9. Distance to health service – outside village  10. Distance to entertainment facility – outside village  11. Distance to public recreation center – outside village  12. Slope  13. Walkability, Design, Diversity  Perceived  14. Access to services – outside village  15. Infrastructure for walking – outside village  16. Safety from crime – outside village  17. Safety from traffic – outside village  18. Fewer physical barriers – outside village  19. Clubhouse – inside village  20. Amenities – inside village  21. Recreational facilities– inside village  22. Access to activity center– inside village  23. Infrastructure for walking – inside village  24. Safety from traffic – inside village  25. Even gradient – inside village | *Outcome*  Accelerometer-assessed high moderate-to-vigorous physical activity [150min+/week (Yes/No)]  Self-reported weekly more/less brisk walking and leisure walking [150min+/week; (Yes/No), and transport-related walking [60min+/week; (Yes/No)]  *Measure:* Device-based and self-report  [Actigraph GT1M and Community Healthy Activities Model Program for Seniors (CHAMPS) instrument]  *Validation:* Yes | None | Logistic regression with generalized estimating equations with backwards elimination accounting for village clustering | Age, gender, education, physical functioning, sampling method, village characteristics, social environment in the village, residency factors, self-selection, mobility factors | *Association with accelerometer-assessed moderate-to-vigorous physical activity*  Objective  1. Distance to local shop – outside village: OR=NR, p>0.05  2. Distance to public transport – outside village: OR=NR, p>0.05  3. Distance to supermarket – outside village: OR=NR, p>0.05  4. Traffic volume exposure – outside village: OR=NR, p>0.05  Perceived  5. Personal safety – inside village: OR=NR, p>0.05  6. Aesthetics – outside village: OR=NR, p>0.05  7. Aesthetics – inside village: OR=NR, p>0.05  8. Proximate destinations – outside village: OR=NR, p>0.05  *Association with self-reported brisk walking*  Objective  1. Distance to local shop – outside village: OR=NR, p>0.05  2. Distance to public transport – outside village: OR=NR, p>0.05  3. Distance to supermarket – outside village: OR=NR, p>0.05  4. Traffic volume exposure – outside village: OR=NR, p>0.05  Perceived  5. Personal safety – inside village: OR=NR, p>0.05  6. Aesthetics – outside village: OR=NR, p>0.05  7. Aesthetics – inside village: OR=NR, p>0.05  8. Proximate destinations – outside village: OR=NR, p>0.05  *Association with self-reported leisure walking*  Objective  1. Distance to local shop – outside village: OR=NR, p>0.05  2. Distance to public transport – outside village: OR=NR, p>0.05  3. **Distance to supermarket – outside village**: OR=1.15, p<0.05  4. **Traffic volume exposure – outside village**: OR=1.35, p<0.05  Perceived  5. Personal safety – inside village: OR=NR, p>0.05  6. **Aesthetics – outside village**: OR=1.53, p<0.05  7. Aesthetics – inside village: OR=1.52, p>0.05  8. Proximate destinations – outside village: OR=NR, p>0.05  *Association with self-reported transport-related walking*  Objective  1. Distance to local shop – outside village: OR=NR, p>0.05  2. Distance to public transport – outside village: OR=NR, p>0.05  3. Distance to supermarket – outside village: OR=NR, p>0.05  4. Traffic volume exposure – outside village: OR=1.23, p>0.05  Perceived  5. Personal safety – inside village: OR=NR, p>0.05  6. Aesthetics – outside village: OR=NR, p>0.05  7. Aesthetics – inside village: OR=NR, p>0.05  8. **Proximate destinations– outside village**: OR=1.65, p<0.05  *Non-significant variables across all outcomes*  Objective  9. Distance to health service – outside village: OR=NR, p>0.05  10. Distance to entertainment facility – outside village: OR=NR, p>0.05  11. Distance to public recreation center – outside village: OR=NR, p>0.05  12. Slope: OR=NR, p>0.05  13. Walkability: OR=NR, p>0.05  Perceived  14. Access to services – outside village: OR=NR, p>0.05  15. Infrastructure for walking – outside village: OR=NR, p>0.05  16. Safety from crime – outside village: OR=NR, p>0.05  17. Safety from traffic – outside village: OR=NR, p>0.05  18. Fewer physical barriers – outside village: OR=NR, p>0.05  19. Clubhouse – inside village: OR=NR, p>0.05  20. Amenities – inside village: OR=NR, p>0.05  21. Recreational facilities– inside village: OR=NR, p>0.05  22. Access to activity center– inside village 🡪 Destination proximity  23. Infrastructure for walking – inside village: OR=NR, p>0.05  24. Safety from traffic – inside village: OR=NR, p>0.05  25. Even gradient – inside village: OR=NR, p>0.05 |
| 57 | Retirement village study [name invented],  Nathan et al., 2014c  [57] | N= 323 (mixed)  Mean age: 77 ± 7 years  Women: 68%  Response rate: 40% and 60%  Retirement village residents  Perth and Peel region, Australia | Cross-sectional  Cluster: purposive  Individuals: random and convenience  Stratification: walkability  Neighborhood definition: 10-15 minutes from home | *Exposure variable - Perceived*  1. Aesthetics – inside village  2. Personal safety– inside village  3. Traffic safety – inside village  4. Aesthetics – outside village  5. Traffic safety – outside village  6. Fewer physical barriers – outside village  7. Orderliness problem – outside village  8. Street connectivity– inside village  9. Services and facilities– inside village  10. Walking infrastructure – outside village  11. Age-appropriate walking infrastructure – outside village  12. Traffic signal transition – outside village  13. Even gradient – inside village  14. Access to services – outside village  15. Proximate destinations – outside village  *Variables not included in the final model due to non-significance*  16. Access to activity center – inside village  17. Walking infrastructure – inside village  18. Traffic safety – outside village  19. Traffic safety – outside village  *Measure:* Perceived  [Neighborhood Environment Walkability Scale (NEWS-A)]  *Validation:* Yes | *Outcome*  Brisk walking and leisure walking [150min+/week; (Yes/No), and transport-related walking [60min+/week; (Yes/No)]  *Measure:* Self-report  [Community Healthy Activities Model Program for Seniors (CHAMPS) instrument]  *Validation:* Yes | None | Logistic regression with generalized estimating equations accounting for village-level clustering | Age, gender, physical functioning, education, self-selection | *Association with leisure-time walking*  1. **Aesthetics – inside village**: OR=1.72, p<0.05  2. Personal safety– inside village: OR=1.24, p>0.05  3. Traffic safety – inside village: OR=0.78, p>0.05  4. Aesthetics – outside village: OR=1.20, p<0.05  5. Traffic safety – outside village: OR=0.76, p>0.05  6. **Fewer physical barriers – outside village**: OR=1.37, p>0.05  7. **Orderliness problem– outside village**: OR=0.67, p<0.05  8. Street connectivity– inside village: OR=NR, p>0.05  9. Services and facilities– inside village: OR=NR, p>0.05  10. Walking infrastructure – outside village: OR=NR, p>0.05  11. Age-appropriate walking infrastructure – outside village: OR=NR, p>0.05  12. Traffic signal transition – outside village: OR=: OR=NR, p>0.05  13. Even gradient – inside village: OR=: OR=NR, p>0.05  14. Access to services – outside village: OR=: OR=NR, p>0.05  15. Proximate destinations – outside village: OR=: OR=NR, p>0.05  *Association with brisk walking*  1. Aesthetics – inside village: OR=0.86, p>0.05  2. **Personal safety– inside village**: OR=0.43, p<0.05  3. Traffic safety – inside village: OR=NR, p>0.05  4. Aesthetics – outside village: OR= NR, p>0.05  5. Traffic safety – outside village: OR= NR, p>0.05  6. Fewer physical barriers – outside village: OR=NR, p>0.05  7. Orderliness problem– outside village: OR=NR, p>0.05  8. **Street connectivity– inside village**: OR=0.71, p<0.05  9. **Services and facilities– inside village**: OR=0.80, p<0.05  10. Walking infrastructure – outside village: OR=1.61, p>0.05  11. Age-appropriate walking infrastructure – outside village: OR=1.51, p>0.05  12. Traffic signal transition – outside village: OR=0.99, p>0.05  13. Even gradient – inside village: OR=NR, p>0.05  14. Access to services – outside village: OR=NR, p>0.05  15. Proximate destinations – outside village: OR=NR, p>0.05  *Association with weekly transport-related walking*  1. Aesthetics – inside village: OR=NR, p>0.05  2. Personal safety– inside village: OR=NR, p>0.05  3. Traffic safety – inside village: OR= NR, p>0.05  4. Aesthetics – outside village: OR= NR, p>0.05  5. Traffic safety – outside village: OR= NR, p>0.05  6. Fewer physical barriers – outside village: OR= NR, p>0.05  7. Orderliness – outside village: OR=0.79, p>0.05  8. Street connectivity– inside village: OR= NR, p>0.05  9. Services and facilities– inside village: OR= NR, p>0.05  10. Walking infrastructure – outside village: OR= NR, p>0.05  11. Age-appropriate walking infrastructure – outside village:  OR=1.14, p>0.05  12. Traffic signal transition – outside village: OR= NR, p>0.05  13. **Even gradient** **– inside village**: OR=0.60, p<0.05  14. Access to services – outside village: OR=0.82, p>0.05  15. **Proximate destinations – outside village**: OR=1.93, p<0.05  *Variables not included in the final model due to non-significance*  16. Access to activity center: OR=NR, p>0.05  17. Walking infrastructure – inside village: OR=NR, p>0.05  18. Traffic safety – outside village: OR=NR, p>0.05  19. Traffic safety – outside village: OR=NR, p>0.05 |
| 58 | NA  Obaid et al., 2020  [58] | N= 4300 (urban)  Age: NR  Women: 44%  Response rate: NR  University students and staff  City of Sharjah, Saudi Arabia | Cross-sectional  Cluster: none  Individuals: convenience  Stratification: none  Neighborhood definition: none | *Exposure variable*  1. Lack of adequate transport infrastructure  2. Inconvenient bus service  3. Trip distance to university  *Measure:* Perceived  [3 items]  *Validation:* No | *Outcome*  Mode choice active transportation (taxi as reference mode)  *Measure:* Self-report  [NR]  *Validation:* No | None | Multinomial logit model | Gender, citizenship, academic status, car sharing, number of cars in the household, car ownership, harsh weather conditions, in-vehicle-time, out-of-vehicle-time | *Association with active transportation as mode choice*  1. **Lack of adequate transport infrastructure**: 30.21, p<0.05  2. **Inconvenient bus service**: 1.65, p<0.05  3. **Trip distance to university**: -0.28, p<0.05 |
| 59 | Household survey,  Olaru & Curtis, 2015  [59] | N= 1533 (urban)  Age: NR  Women: NR  Response rate: NR  Community dwellers  Perth, Australia | Natural experiment  Cluster: purposive  Individuals: NR  Stratification: precinct  Neighborhood definition: none | *Exposure variable*  Railway opening  *Measure:* Objective  *Validation:* Yes | *Outcome*  % trips by active transport (cycling and walking) before and after the railway opening  *Measure:* Self-report  [1 item]  *Validation:* No | None | MANCOVA | Household size, car availability, income | *Association with active transport*  **Bull Creek**  Pre: 16.23%  Post: 17.89%  **Cockburn Central**  Pre: 11.22%  Post:16.65%  **Wellard**  Pre: 9.88%  Post:14.99%  **Total**  Pre: 12.60%  Post:16.42%  p-levels not reported |
| 60 | NA  Olojede et al., 2017  [60] | N= 524 (urban)  47% 21-40 years  Women: 48%  Response rate: 95%  Community dwellers  Ilesa, Nigeria | Cross-sectional  Cluster: purposive  Individuals:  Stratification: area density  Neighborhood definition: political wards | *Exposure variable*  1. Trip length  *Measure:* Perceived  [NR]  *Variables eliminated during stepwise regression*  2. Safety and security issues  3. Absence of pedestrian facilities  4. Relative slowness  5. Road business  6. Unfriendly topography  *Validation:* No | *Outcome*  Walking as mode choice [Yes/N]  *Measure:* Self-report  [NR]  *Validation:* No | None | Stepwise linear regression without accounting for political wards | Personal vehicle, income, travel costs, health benefits | *Association with walking*  1. **Trip length**: Beta=-0.12, p<0.05  *Variables eliminated during stepwise regression*  2. Safety and security issues: Beta=NR, p>0.05  3. Absence of pedestrian facilities: Beta=NR, p>0.05  4. Relative slowness: Beta=NR, p>0.05  5. Road business: Beta=NR, p>0.05  6. Unfriendly topography: Beta=NR, p>0.05 |
| 61 | NA  Oyeyemi et al., 2012  [61] | N= 219 (urban)  41% 20-29 years  Women: 39%  Response rate: 9%  Community dwellers  Maiduguri, Nigeria | Cross-sectional  Cluster: purposive  Individuals: random  Stratification: socio-economic status and walkability  Neighborhood definition: 10-15 minute walking distance from home or around 1km or half a mile (participant) | *Exposure variable*  1. Traffic safety for bicycling  2. Traffic safety for walking  3. Crime safety during the day  4. Crime safety at night  *Measure:* Perceived  [Physical Activity Neighborhood Environment Scale; (PANES)]  *Validation:* Yes | *Outcome*  Meeting the physical activity guidelines [accelerometer-assessed 150min+ moderate-to-vigorous physical activity/week; (Yes/No)]  Self-reported total walking minutes [150min+/week; (Yes/No)]  *Measure:* Device-based and self-reported  [Uniaxial accelerometer CSA Model 7614 and International Physical Activity Questionnaire (IPAQ-SF)]  *Validation:* Yes | Gender | Logistic regression without accounting for lustering | Age, gender, socio-economic neighborhood status, employment status, education | *Meeting the physical activity guidelines*  1. Traffic safety for bicycling: OR=0.87, p>0.05  2. **Traffic safety for walking**: OR=2.28, p<0.05  3. **Crime safety during the day**: OR=0.34, p<0.05  4. **Crime safety at night**: OR=1.68, p<0.05  *Self-reported walking*  1. Traffic safety for bicycling: OR=2.26, p>0.05  2. Traffic safety for walking: OR=1.19, p>0.05  3. **Crime safety during the day**: OR=5.92, p<0.05  4. **Crime safety at night**: OR=6.99, p<0.05  *Meeting the physical activity guidelines stratified by gender*  1. Traffic safety for bicycling:  Women: OR=0.77, p>0.05  Men: OR=0.54, p>0.05  2. Traffic safety for walking:  Women: OR=NR, p>0.05  Men: OR= NR, p>0.05  3. Crime safety during the day:  Women: OR= NR, p>0.05  Men: OR= NR, p>0.05  4. **Crime safety at night**:  Women: OR=0.63, p>0.05  **Men**: OR=3.82, p<0.05  *Self-reported walking stratified by gender*  1. Traffic safety for bicycling:  Women: OR= NR, p>0.05  Men: OR= NR, p>0.05  2. Traffic safety for walking:  Women: OR= NR, p>0.05  Men: OR= NR, p>0.05  3. **Crime safety during the day:**  **Women**: OR=10.27, p<0.05  Men: OR=3.34, p>0.05  4. **Crime safety at night**:  Women: OR=3.09, p>0.05  **Men**: OR=6.97, p<0.05 |
| 62 | NA  Oyeyemi et al., 2013  [62] | N= 1411 (urban)  Mean age: 36 ± 10 years Women: 43%  Response rate: 69%  Community dwellers  Maiduguri, Nigeria | Cross-sectional  Cluster: purposive  Individuals: random  Stratification: socio-economic status and walkability  Neighborhood definition: enumeration area | *Exposure variable*  1. Residential density  2. Commercial destination access  3. Street connectivity  4. Seeing people active  5. Aesthetics  6. Absence of garbage  7. Traffic safety for bicycling  8. Traffic safety for walking  9. Crime safety at night  *Measure:* Perceived  [Adapted Physical Activity Neighborhood Environment Scale; (PANES-N)]  *Validation:* Yes | *Outcome*  Total minutes of walking  Moderate-to-vigorous physical activity  Total physical activity  (MET-min/week)  *Measure:* Self-report  [International Physical Activity Questionnaire (IPAQ-SF)]  *Validation:* Yes | None | McKinnon product-of-coefficient test (including regression analysis) without accounting for clustering in enumeration areas | Gender, age, working status, education | *MVPA*  1. **Residential density**: β= -0.03, p<0.05  2. Commercial destination access: β=0.02, p>0.05  3. **Street connectivity**: β= 0.08, p<0.05  4. Seeing people active: β= -0.02, p>0.05  5. **Aesthetics**: β=0.04, p<0.05  6. **Absence of garbage**: β=0.03, p<0.05  7. Traffic safety for bicycling: β=0.01, p>0.05  8. Traffic safety for walking: β= -0.02, p>0.05  9. **Crime safety at night**: β=0.06, p<0.05  *Walking*  1. **Residential density**: β= 0.05, p<0.05  2. Commercial destination access: β= 0.10, p>0.05  3. **Street connectivity**: β= 0.10, p<0.05  4. Seeing people active: β= -0.02, p>0.05  5. **Aesthetics**: β=0.07, p<0.05  6. **Absence of garbage**: β=0.12, p<0.05  7. Traffic safety for bicycling: β= -0.03, p>0.05  8. **Traffic safety for walking**: β= -0.04, p<0.05  9. **Crime safety at night**: β=0.11, p<0.05  *Total physical activity*  1. **Residential density**: β= 0.04, p<0.05  2. **Commercial destination access**: β= 0.05, p<0.05  3. **Street connectivity**: β= 0.10, p<0.05  4. Seeing people active: β= -0.02, p>0.05  5. **Aesthetics**: β=0.08, p<0.05  6. **Absence of garbage**: β=0.10, p<0.05  7. Traffic safety for bicycling: β= 0.00, p>0.05  8. **Traffic safety for walking**: β= -0.03, p<0.05  9. **Crime safety at night**: β=0.10, p<0.05 |
| 63 | NA  Oyeyemi et al., 2015  [63] | N= 613 (urban)  Mean age: 28 ± 8 years Women: 32%  Response rate: 66%  Community dwellers in low-income neighborhoods  Maiduguri, Nigeria | Cross-sectional  Cluster: purposive  Individuals: random  Stratification: none  Neighborhood definition: enumeration area | *Exposure variable*  1. Absence of garbage  2. Presence of beautiful things  3. Absence of unattended animals  4. Inadequate crime security at night  5. Dangerous driving and traffic speed  6. Inadequate crime security during the day  *Measure:* Perceived  [Physical Activity Neighborhood Environment Scale; (PANES)]  *Validation:* Yes | *Outcome*  Sufficient health-related physical activity (150min+ moderate-to-vigorous physical activity; (Yes/No)]  *Measure:* Self-report  [International Physical Activity Questionnaire (IPAQ-SF)]  *Validation:* Yes | Gender | Logistic regression analysis without accounting for clustering in enumeration areas | Age, gender, marital status, ethnic group, religion, education, employment status | *Association with sufficient physical activity*  1. **Absence of garbage:** OR=1.56, p<0.05  2. Presence of beautiful things: OR=1.08, p>0.05  3. Absence of unattended animals: OR=1.59, p>0.05  4. Inadequate crime security at night: OR=0.81, p>0.05  5. **Dangerous driving and traffic speed**: OR=0.63, p<0.05  6. **Inadequate crime security during the day**: OR=0.62, p<0.05  *Association with sufficient physical activity - women*  1. **Absence of garbage**: OR=2.90, p<0.05  2. Presence of beautiful things: OR=1.37, p>0.05  3. Absence of unattended animals: OR=2.18, p>0.05  4. Inadequate crime security at night: OR=0.79, p>0.05  5. **Dangerous driving and traffic speed**: OR=0.50, p<0.05  6. **Inadequate crime security during the day**: OR=0.43, p<0.05  *Association with sufficient physical activity - men*  1. Absence of garbage: OR=1.23, p>0.05  2. Presence of beautiful things: OR=0.96, p>0.05  3. Absence of unattended animals: OR=1.30, p>0.05  4. Inadequate crime security at night: OR=0.81, p>0.05  5. Dangerous driving and traffic speed: OR=0.70, p>0.05  6. Inadequate crime security during the day: OR=0.76, p>0.05 |
| 64 | NA  Oyeyemi et al., 2018  [64] | N= 353 (urban)  Mean age: 69 ± 9 years Women: 40%  Response rate: 83%  Older adults  Maiduguri, Nigeria | Cross-sectional  Cluster: purposive  Individuals: random  Stratification: socio-economic status and walkability  Neighborhood definition: enumeration area | *Exposure variable*  1. Residential density  2. Destination proximity  3. Access to services and places  4. Street connectivity  5. Walking infrastructure and safety  6. Aesthetics  7. Traffic safety  8. Crime safety  *Measure:*  [Neighborhood Environment Walkability Scale (NEWS-A)]  *Validation:* Yes | *Outcome*  Total moderate-to-vigorous physical activity min/week  Transport-related walking min/week  Recreational walking min/week  *Measure:* Self-report  [International Physical Activity Questionnaire (IPAQ)]  *Validation:* Yes | Gender | Multilevel linear regression analysis | Age, gender, marital status, education, employment, neighborhood strata | *Association with total MVPA*  1. Residential density: β=0.01, p>0.05  2. **Destination proximity**: β=3.29, p<0.05  3. Access to services and places: β=2.04, p>0.05  4. Street connectivity: β= -0.46, p>0.05  5. Walking infrastructure and safety: β=2.15, p>0.05  6. Aesthetics: β=1.57, p>0.05  7. **Traffic safety**: β=5.69, p<0.05  8. **Crime safety**: β=1.72, p<0.05  *Association with transport-related walking*  1. Residential density: β=0.00, p>0.05  2. **Destination proximity**: β=1.66, p<0.05  3. Access to services and places: β=0.96, p>0.05  4. Street connectivity: β= -0.29, p>0.05  5. Walking infrastructure and safety: β= 1.64, p>0.05  6. Aesthetics: β=0.20, p>0.05  7. Traffic safety: β=0.07, p>0.05  8. **Crime safety**: β=2.21, p<0.05  *Association with recreational walking*  1. Residential density: β=0.00, p>0.05  2. Destination proximity: β=0.84, p>0.05  3. **Access to services and places**: β=2.09, p<0.05  4. Street connectivity: β=0.75, p>0.05  5. **Walking infrastructure and safety**: β=1.74, p<0.05  6. Aesthetics: β=0.23, p>0.05  7. Traffic safety: β=0.35, p>0.05  8. Crime safety: β=0.41, p>0.05  *Association with total MVPA stratified by gender*  1. Residential density:  Men: β=NR, p>0.05  Women: β=NR, p>0.05  2. **Destination proximity**:  Men: β=0.96, p>0.05  **Women**: β=4.73, p<0.05  3. **Access to services and places**:  Men: β= -0.65, p>0.05  **Women**: β=4.42, p<0.05  4. Street connectivity:  Men: β=NR, p>0.05  Women: β=NR, p>0.05  5. Walking infrastructure and safety:  Men: β=NR, p>0.05  Women: β=NR, p>0.05  6. **Aesthetics**:  Men: β= -1.19, p>0.05  **Women**: β=3.60, p<0.05  7. **Traffic safety**:  **Men**: β=9.81, p<0.05  Women: β=1.87, p>0.05  8. **Crime safety**:  **Men**: β=2.31, p<0.05  Women: β=0.35, p>0.05  *Association with transport-related walking stratified by gender*  1. Residential density:  Men: β=NR, p>0.05  Women: β=NR, p>0.05  2. Destination proximity:  Men: β=NR, p>0.05  Women: β=NR, p>0.05  3. Access to services and places:  Men: β=NR, p>0.05  Women: β=NR, p>0.05  4. Street connectivity:  Men: β=NR, p>0.05  Women: β=NR, p>0.05  5. **Walking infrastructure and safety**:  **Men**: β=3.52, p<0.05  Women: β= -0.69, p>0.05  6. Aesthetics:  Men: β=NR, p>0.05  Women: β=NR, p>0.05  7. Traffic safety:  Men: β=NR, p>0.05  Women: β=NR, p>0.05  8. Crime safety:  Men: β=NR, p>0.05  Women: β=NR, p>0.05  *Association with recreational walking stratified by gender*  1. Residential density:  Men: β=NR, p>0.05  Women: β=NR, p>0.05  2. Destination proximity:  Men: β=NR, p>0.05  Women: β=NR, p>0.05  3. **Access to services and places**:  Men: β=2.00, p>0.05  **Women**: β=1.79, p<0.05  4. Street connectivity:  Men: β=NR, p>0.05  Women: β=NR, p>0.05  5. **Walking infrastructure and safety**:  **Men**: β=2.85, p<0.05  Women: β=0.23, p>0.05  6. Aesthetics:  Men: β=NR, p>0.05  Women: β=NR, p>0.05  7. **Traffic safety**:  **Men**: β=1.84, p<0.05  **Women**: β= -1.29, p<0.05  8. Crime safety:  Men: β=NR, p>0.05  Women: β=NR, p>0.05 |
| 65 | NA  Pimenta et al., 2020  [65] | N= 438 (urban)  54% 25-32 years  Women: 44%  Response rate: NR  Travelers  Abu Dhabi, United Arab Emirates | Cross-sectional  Cluster: purposive  Individuals: convenience  Stratification: none  Neighborhood definition: 400m circular buffer or 5-min walk (recruitment) and 1 km (participant) | *Exposure variable - Perceived*  1. Other people walking/biking in the neighborhood  2. No. of service destinations accessible by walk/bike  3. No. of recreational destinations reached by walk/bike  4. Land-use accessibility index  5. High traffic in street of household  6. High traffic in nearby streets of household  *Measure:* Perceived  [Neighborhood Environment Walkability Scale (NEWS)]  *Validation:* Yes  *Exposure variable - Objective*  7. Service destination within 1km  8. Administrative destination within 1km  9. Recreational destination within 1km  10. Work destination within 1km  11. Religious destination within 1km  12. Bus stops within 1km  13. Walkability, Density, Diversity  *Measure: Objective*  [GIS, database not reported]  *Validation:* No | *Outcome*  Attainment of transport-related recommended physical activity [150min+/week; (Yes/No)]  Attainment of recreational recommended physical activity [150min+/week; (Yes/No)]  Attainment of total physical activity [150min+/week; (Yes/No)]  *Measure:* Self-report  [International Physical Activity Questionnaire (IPAQ)]  *Validation:* Yes | None | Binomial regression | Cars per household, household type, size, and income, mode of motorized travel used | *Association with recommended transport-related physical activity*  Perceived  1. **Other people walking/biking in the neighborhood**: B= -0.17, p<0.05  2. **No. of service destinations accessible by walk/bike**: B=0.10, p<0.05  3. No. of recreational destinations reached by walk/bike: B=NR, p>0.05  4. Land-use accessibility index: B=NR, p>0.05  5. High traffic in street of household: B=NR, p>0.05  6. High traffic in nearby streets of household: B=NR, p>0.05  Objective  7. Service destination within 1km: B=0.05, p>0.05  8. Administrative destination within 1km: B=-0.06, p>0.05  9. Recreational destination within 1km: B=0.00, p>0.05  10. Work destination within 1km: B=0.29, p>0.05  11. Religious destination within 1km: B=0.32, p>0.05  12. **Bus stops within 1km**: B= -0.90, p<0.05  13. Walkability: B=NR, p>0.05  *Association with attainment of recreational physical activity*  Perceived  1. Other people walking/biking in the neighborhood: B=0.11, p>0.05  2. No. of service destinations accessible by walk/bike: B=NR, p>0.05  3. No. of recreational destinations reached by walk/bike: B=0.03, p>0.05  4. **Land-use accessibility index**: B= -2.27, p<0.05  5. High traffic in street of household: B=0.18, p>0.05  6. **High traffic in nearby streets of household**: B= -0.28, p<0.05  Objective  7. Service destination within 1km: B=0.02, p>0.05  8. **Administrative destination within 1km**: B=0.23, p<0.05  9. Recreational destination within 1km: B= -0.12, p>0.05  10. **Work destination within 1km**: B=0.29, p<0.05  11. **Religious destination within 1km**: B=0.36, p<0.05  12. Bus stops within 1km: B= -0.24, p>0.05  13. Walkability: B=NR, p>0.05  *Association with attainment of total physical activity*  Perceived  1. **Other people walking/biking in the neighborhood**: B= -0.36, p<0.05  2. **No. of service destinations accessible by walk/bike**: B=0.24, p<0.05  3. **No. of recreational destinations reached by walk/bike**: B=0.55, p<0.05  4. Land-use accessibility index: B=NR, p>0.05  5. High traffic in street of household: B=NR, p>0.05  6. High traffic in nearby streets of household: B=NR, p>0.05  Objective  7. **Service destination within 1km**: B= -0.32, p<0.05  8. Administrative destination within 1km: B= -0.05, p>0.05  9. Recreational destination within 1km: B=0.23, p>0.05  10. Work destination within 1km: B=0.71, p>0.05  11. **Religious destination within 1km**: B=1.63, p<0.05  12. Bus stops within 1km: B=0.19, p>0.05  13. Walkability: B=NR, p>0.05  0 |
| 66 | Household travel survey  Rahul et al., 2017  [66] | N= 6722 (urban)  Age: NR  Women: NR%  Response rate: NR  Community dwellers  Bangalore, India | Cross-sectional  Cluster: purposive  Individuals: 1) households, 2) individuals - random  Stratification: none  Neighborhood definition: zones | *Exposure variable*  1. Total density in origin zone of trip  2. Total density at destination zone of trip  3. Employment accessibility  4. Land use diversity  5. Travel time  *Measure:* Objective and self-report  [Census data]  *Validation:* Yes | *Outcome*  Cycling and walking as mode choice  *Measure:* Self-report  [NR]  *Validation:* No | Vehicle owning | Multinomial logit model without accounting for clustering | Travel cost, household income, trip purpose, gender, age, vehicle-worker-ratio, out of vehicle travel time by public transit, head of household | *Association with mode choice for vehicle owning group (ref. car)*  Cycling  1. Total density in origin zone of trip: t-statistic=NR  2. **Total density at destination zone of trip**: t-statistic=0.01  3. Employment accessibility: t-statistic=NR  4. **Land use diversity**: t-statistic=1.27  5. **Travel time**: t-statistic= -0.08  Walking  1. Total density in origin zone of trip: t-statistic=NR  2. **Total density at destination zone of trip**: t-statistic=0.03  3. Employment accessibility: t-statistic==NR  4. **Land use diversity:** t-statistic=1.04  5. **Travel time**: t-statistic= -0.09  *Association with mode choice for non-vehicle owning group (ref. public transit)*  Cycling  1. **Total density in origin zone of trip**: t-statistic= -0.04  2. **Total density at destination zone of trip**: t-statistic= -0.09  3. Employment accessibility: t-statistic=NR  4. **Land use diversity**: t-statistic=1.95  5. **Travel time**: t-statistic= -0.15  Walking  1. **Total density in origin zone of trip: t-statistic**= -0.04  2. **Total density at destination zone of trip**: t-statistic= -0.07  3. Employment accessibility: t-statistic==NR  4. **Land use diversity:** t-statistic=0.17  5. **Travel time**: t-statistic= -0.15 |
| 67 | NA  Rai et al., 2018  [67] | N= 432 (urban)  Mean age: 70 ± 6 years  Women: 56%  Response rate: NR  Older adult community dwellers  Perth, Australia | Cross-sectional  Cluster: none  Individuals: Convenience  Stratification: none  Neighborhood definition: none | *Exposure variable*  1. Walkability  2. Public open spaces density  *Measure*: Objective  [WalkScore, POS Tool]  *Validation:* Yes | *Outcome*  Minutes of MVPA/week  *Measure:* Device-based  [Actigraph GT3X]  *Validation:* Yes | None | Correlation analysis | None | *MVPA*  1. **Walkabilit**y: r = 0.13, p<.05  2. Public Open Spaces Density: r = 0.07, p>0.05 |
| 68 | NA  Ross et al., 2019  [68] | N= 625 (urban)  29% 18-34 years  Women: 62%  Response rate: NR  Community dwellers  Phoenix, Arizona, USA | Cross-sectional  Cluster: none  Individuals: random  Stratification: age group  Neighborhood definition: 10-15 min walk from home | *Exposure variable*  1. Neighborhood environment, Diversity, Destination accessibility, Destination proximity, Desirability, Design  *Measure*: Perceived  [Physical Activity Neighborhood Environment Scale (PANES)]  *Validation:* Yes | *Outcome*  Leisure-time physical activity frequency (latent construct)  *Measure:* Self-report  [Neighborhood Physical Activity Questionnaire (NPAQ)]  *Validation:* Yes | None | Structural equation modeling | Age, gender, education, marital status, employment, income, no. of children at home, health, social support, attitudes, character, loyalty, home ownership, homeowner’s association, years in neighborhood | *Association with leisure-time physical activity*  1. **Neighborhood environment**: β=0.21, p<0.05 |
| 69 | NA  Ross et al., 2021  [69] | N= 796 (urban)  Mean age: 48 ± 17 years  Women: 51%  Response rate: NR  YouGov panel residents  Phoenix, Arizona, USA | Cross-sectional  Cluster: none  Individuals: random  Stratification: age, gender, race  Neighborhood definition: 10-15 min walk from home | *Exposure variable*  1. Neighborhood environment, Diversity, Destination accessibility, Destination proximity, Desirability, Design  *Measure*: Perceived  [Physical Activity Neighborhood Environment Scale (PANES)]  *Validation:* Yes | *Outcome*  Total walking [150min+/week; (Yes/No)]  Any recreational walking [Yes/No]  *Measure:* Self-report  [Neighborhood Physical Activity Questionnaire (NPAQ)]  *Validation:* Yes | None | Structural equation modeling | Age, gender, education, marital status, employment, income, no. of children at home, health, social support, attitudes, character, loyalty, home ownership, homeowner’s association, years in neighborhood | *Association with total walking*  1. **Neighborhood environment**: β=0.18, p<0.05  *Association with recreational walking*  1. **Neighborhood environment**: β=0.23, p<0.05 |
| 70 | Enlace,  Salinas et al., 2018  [70] | N= 614 (rural)  Mean age: 40 ± 10 years  Women: 100%  Response rate: NR  Hispanic women not meeting the physical activity guidelines  Rio Grande Valley, Texas, USA | Cross-sectional  Cluster: none  Individuals: NR  Stratification: none  Neighborhood definition: none | *Exposure variable*  1. Neighborhood environment  *Measure*: Perceived  [Physical Activity Neighborhood Environment Scale (PANES)]  *Validation:* Yes | *Outcome*  Device-based and self-reported moderate-vigorous physical activity (min/day)  Brisk, errand, and leisure-time walking (<2, 2-3, and >3 hours/week)  *Measure:* Device-based and self-report  [Actigraph GT3X and Community Healthy Activities Model Program for Seniors (CHAMPS) instrument]  *Validation:* Yes | Social support | Regression analysis | Age, education, household income, country of birth, health insurance, %body fat | *Association with accelerometer-assessed MVPA*  1. Neighborhood environment: B= -0.008, p>0.05  *Association with self-reported MVPA*  1. **Neighborhood environment**: B=0.30, p<0.05  *Association between PANES and brisk walking (ref. <2 hours/week)*  2-3 hours/week : OR= 1.10, p>0.05  >3 hours/week: OR=1.16, p>0.05  *Association between PANES and errand-related walking (ref. <2 hours/week)*  2-3 hours/week : OR= 1.06, p>0.05  >3 hours/week: OR=1.13, p>0.05  *Association between PANES and leisure-time walking (ref. <2 hours/week)*  2-3 hours/week : OR= 0.98, p>0.05  >3 hours/week: OR=1.09, p>0.05  *No interaction between social support and PANES for physical activity* |
| 71 | IPEN (International Physical Activity and Environment Network) study  Salvo et al., 2014  [71] | N= 662 (urban)  40% 35-50 years  Women: 52%  Response rate: 59%  Community dwellers  Cuernavaca, Mexico | Cross-sectional  Cluster: purposive and random  Individuals: 1) households – random, 2) individuals - random  Stratification: socio-economic status and walkability  Neighborhood definition: census-tract (recruitment) and 500-1000m street-network buffer (participant) | *Exposure variable - Perceived*  1. Neighborhood safety  2. Park safety  *Measure*: Perceived  [Neighborhood Environment Walkability Scale (NEWS-A)]  *Validation:* Yes  *Exposure variable - Objective*  3. Distance to park  4. No of public routes  5. Net residential density 500m buffer  6. Net residential density 1000m buffer  7. Proportion of commercial land use 500m buffer  8. Proportion of commercial land use 1000m buffer  9. Land-use mix 500m buffer  10. Land-use mix 1000m buffer  11. Connectivity 500m buffer  12. Connectivity 1000m buffer  13. Walkability 500m buffer  14. Walkability 1000m buffer  15. No of parks 500m buffer  16. No of parks 1000m buffer  *Measure*: Objective  [ArcGIS, 500m and 1000m street-network buffer, census tract data for categories]  *Validation:* Yes | *Outcome*  Moderate-to-vigorous physical activity (min/week)  Moderate-to-vigorous physical activity within 10-min bouts  *Measure:* Device-based  [ActiGraph GT3X]  *Validation:* Yes | Perceived safety and built environment | Regression analysis accounting for census tract clustering | Age, gender, education, marital status, socio-economic status, vehicle ownership, body-mass-index | *Association with total MVPA*  Perceived  1. Neighborhood safety: B= -1.33, p>0.05  2. Park safety: B= -23.17, p>0.05  Objective  3. Distance to park  Medium: B=12.93, p>0.05  Far: B=6.13, p>0.05  Very far: B=15.56, p>0.05  4. **No of public routes**  1: B= -7.76, p>0.05  2-7: B= -15.55, p>0.05  **≥8:** B= -23.78, p<0.05  5. Net residential density 500m buffer (ref. low):  Medium: B= -4.72, p>0.05  Medium-high: B= -39.10, p>0.05  High: B= -30.58, p>0.05  6. **Net residential density 1000m buffer** (ref. low):  **Medium**: B= -39.88, p<0.05  Medium-high: B= -40.42, p>0.05  High: B= -15.99, p>0.05  7. **Proportion of commercial land use 500m buffer** (ref. low):  Medium: B= -2.77, p>0.05  Medium-high: B= -9.48, p>0.05  **High**: B= -54.51, p<0.05  8. Proportion of commercial land use 1000m buffer (ref. low):  Medium: B= -0.37, p>0.05  Medium-high: B= -26.26, p>0.05  High: B= -20.27, p>0.05  9. Land-use mix 500m buffer (ref. low):  Medium: B=6.29, p>0.05  Medium-high: B= -25.61, p>0.05  High: B=-12.68, p>0.05  10. Land-use mix 1000m buffer (ref. low):  Medium: B=14.44, p>0.05  Medium-high: B= -11.20, p>0.05  High: B= -0.39, p>0.05  11. Connectivity 500m buffer (ref. low):  Medium: B= -36.14, p>0.05  Medium-high: B= -22.63, p>0.05  High: B= -18.68, p>0.05  12. **Connectivity 1000m buffer** (ref. low):  Medium: B= -21.55, p>0.05  **Medium-high**: B= -35.51, p<0.05  High: B= -32.07, p>0.05  13. Walkability index 500m buffer (ref. low):  Medium: B= -24.35, p>0.05  Medium-high: B= -27.11, p>0.05  High: B= -34.30, p>0.05  14. **Walkability 1000m buffer** (ref. low):  Medium: B= -20.42, p>0.05  Medium-high: B= -12.89, p>0.05  **High**: B= -46.91, p<0.05  15. No of parks 500m buffer (ref. 0):  1: B= -27.87, p=0.05  ≥ 2: B=31.61, p>0.05  16. No of parks 1000m buffer (ref. low):  1: B= -10.82, p>0.05  ≥ 2: B= -3.27, p>0.05  *Association with MVPA bouts*  Perceived  1. Neighborhood safety: B= -2.61, p>0.05  2. Park safety: B= -12.00, p=0.05  Objective  3. Distance to park  Medium: B=9.91, p>0.05  Far: B=8.16, p>0.05  Very far: B=8.24, p>0.05  4. No of public bus routes  1: B= -1.59, p>0.05  2-7: B= -4.51, p>0.05  ≥8: B= --6.95, p<0.05  5. **Net residential density 500m buffer** (ref. low):  Medium: B= -7.97, p>0.05  Medium-high: B= -22.87, p=0.05  **High**: B= -21.68, p<0.05  6. **Net residential density 1000m buffer** (ref. low):  **Medium**: B= -24.32, p<0.05  Medium-high: B= -23.34, p=0.05  High: B= -8.92, p>0.05  7. **Proportion of commercial land use 500m buffer** (ref. low):  Medium: B= -0.82, p>0.05  Medium-high: B= -8.59, p>0.05  **High:** B= -33.96, p<0.05  8. Proportion of commercial land use 1000m buffer (ref. low):  Medium: B=1.56, p>0.05  Medium-high: B= -5.70, p>0.05  High: B= -20.31, p>0.05  9. Land-use mix 500m buffer (ref. low):  Medium: B= -5.49, p>0.05  Medium-high: B= -20.65, p>0.05  High: B= -19.17, p>0.05  10. **Land-use mix 1000m buffer** (ref. low):  Medium: B= 1.03, p>0.05  **Medium-high**: B= -22.33, p<0.05  High: B= -17.34, p=0.05  11. Connectivity 500m buffer (ref. low):  Medium: B= -8.68, p>0.05  Medium-high: B= -11.54, p>0.05  High: B= -10.35, p>0.05  12. Connectivity 1000m buffer (ref. low):  Medium: B= -9.24, p>0.05  Medium-high: B= -18.33, p>0.05  High: B= -9.48, p>0.05  13. **Walkability index 500m buffer** (ref. low):  Medium: B= -10.22, p>0.05  Medium-high: B= -18.31, p>0.05  **High**: B= -31.49, p<0.05  14. **Walkability 1000m buffer** (ref. low):  **Medium**: B= -22.25, p<0.05  **Medium-high**: B= -34.21, p<0.05  High: B= -12.65, p>0.05  15. **No of parks 500m buffer** (ref. 0):  **1**: B= -16.84, p<0.05  ≥ 2: B= 20.51, p>0.05  16. No of parks 1000m buffer (ref. 0):  1: B= 0.24, p>0.05  ≥ 2: B= -2.17, p>0.05  *Interaction between unsafe park perception and number of parks within 500m buffer*  1 park: B= -30.8 total MVPA, p<0.05  1 park: B= -19.2 MVPA in bouts, p<0.05  No significant association if park was perceived as safe |
| 72 | IPEN (International Physical Activity and Environment Network) study  Salvo et al., 2017  [72] | N= 677 (urban)  39% 35-49 years  Women: 55%  Response rate: 59%  Community dwellers  Cuernavaca, Mexico | Cross-sectional  Cluster: purposive and random  Individuals: 1) households – random, 2) individuals - random  Stratification: socio-economic status and walkability  Neighborhood definition: census-tract | *Exposure variable*  1. Use of public access places for physical activity  2. Use of restricted access places for physical activity  *Measure*: Self-report  [Items asking about physical activity in specific places]  *Validation:* No | *Outcome*  Self-reported leisure walking [150min+/week, (Yes/No)]  Self-reported leisure-time MVPA [150min+/week, (Yes/No)]  Accelerometer-assessed MVPA [150min+/week, (Yes/No)]  Accelerometer-assessed MVPA within 10min bouts [150min+/week, (Yes/No)]  *Measure:* Self-report and device-based  [International Physical Activity Questionnaire (IPAQ-SF) and Actigraph GT3X]  *Validation:* Yes | None | Mixed-effect regression models with logit link function accounting for neighborhood clustering | Gender, age, education, motor vehicle ownership, use of home for physical activity, | *Self-reported leisure-time walking*  1. **Use of public access places for physical activity**: OR=4.9, p<0.05  2. **Use of restricted access places for physical activity**: OR=2.5, p<0.05  *Self-reported leisure-time MVPA*  1. **Use of public access places for physical activity**: OR=5.2, p<0.05  2. **Use of restricted access places for physical activity**: OR=8.1, p<0.05  *Device-based MVPA*  1. **Use of public access places for physical activity**: OR=1.3, p<0.05  2. Use of restricted access places for physical activity: OR=0.8, p>0.05  *Device-based MVPA within bouts*  1. **Use of public access places for physical activity**: OR=3.6, p<0.05  2. **Use of restricted access places for physical activity**: OR=1.9, p<0.05 |
| 73 | NA  Shuval et al., 2009  [73] | N= 198 (mixed)  Mean age: 23 years  Women: 59%  Response rate: 95%  Physical education college students  Qatsrin, Israel | Cross-sectional  Cluster: None  Individuals: Convenience  Stratification: None  Neighborhood definition: none | *Exposure variable*  1. Presence of sidewalks  2. Presence of bike lanes  3. High crime rate  4. Street lighting  5. Unattended dogs  6. Too many hills  7. Heavy traffic  8. Poor scenery  9. Verbal abuse  10. Speeding drivers  11. Neighborhood open space access  *Measure*: Perceived  [11 items]  *Validation:* No | *Outcome*  Meeting the physical activity guidelines [150min+/week of moderate-to-vigorous physical activity; (Yes/No)]  *Measure:* Self-report  [International Physical Activity Questionnaire (IPAQ-SF)]  *Validation:* Yes | None | Logistic regression analysis | Gender, ethnicity, lack of energy, self-efficacy | *Association with meeting physical activity guidelines*  1. Presence of sidewalks: OR=NR, p>0.05  2. Presence of bike lanes: OR=NR, p>0.05  3. High crime rate: OR=NR, p>0.05  4. Street lighting: OR=NR, p>0.05  5. Unattended dogs: OR=NR, p>0.05  6. Too many hills: OR=NR, p>0.05  7. Heavy traffic: OR=NR, p>0.05  8. Poor scenery: OR=NR, p>0.05  9. Verbal abuse: OR=NR, p>0.05  10. Speeding drivers: OR=NR, p>0.05  11. **Neighborhood open space access**: OR=2.67, p<0.05  Variables without reported OR were not included in final model to due non-significance in univariate analysis |
| 74 | RESIDE,  Sugiyama et al., 2010  [74] | N= 1366 (urban)  Mean age: 42 ± 12 years  Women: 60%  Response rate: 33%  Community dwellers  Perth, Australia | Cross-sectional  Cluster: Purposive  Individuals: Two-stage approach; 1) households (census); 2) individuals (random)  Stratification: Relocating development type (Livable Neighborhood, hybrid, conventional)  Neighborhood definition: 1600m street network buffer and 10-15 min walk from home | *Exposure variable*  1. Neighborhood open space attractiveness  2. Neighborhood open space size  3. Neighborhood open space network distance  *Measure*: Objective  [Public Open Space Tool (POST) and GIS street network-buffer 1600m]  *Validation:* Yes | *Outcome*  Any recreational neighborhood walking [Yes/No]  Sufficient recreational neighborhood walking [150min+/week of recreational walking (Yes/No)]  *Measure:* Self-report  [Neighborhood Physical Activity Questionnaire (NPAQ)]  *Validation:* Yes | Stratified by most attractive, largest, and nearest open space | Logistic regression | Age, gender, children in the household | *Association with recreational walking*  Most attractive neighborhood open space  1. Higher attractiveness: OR=1.19, p>0.05  2. Larger size: OR=1.13, p>0.05  3. **Nearer distance**: OR=1.33, p<0.05  Largest neighborhood open space  1. Higher attractiveness: OR=1.05, p>0.05  2. Larger size: OR=1.13, p>0.05  3. Nearer distance: OR=1.18, p>0.05  Nearest neighborhood open space  1. **Higher attractiveness**: OR=1.34, p<0.05  2. Larger size: OR=1.28, p>0.05  3. Nearer distance: OR=1.00, p>0.05  *Association with sufficient walking*  Most attractive neighborhood open space  1. Higher attractiveness: OR=0.93, p>0.05  2. **Larger size**: OR=1.38, p<0.05  3. Nearer distance: OR=1.14, p>0.05  Largest neighborhood open space  1. Higher attractiveness: OR=1.26, p>0.05  2. Larger size: OR=0.85, p>0.05  3. Nearer distance: OR=1.20, p>0.05  Nearest neighborhood open space  1. Higher attractiveness: OR=1.00, p>0.05  2. Larger size: OR=0.96, p>0.05  3. Nearer distance: OR=1.08, p>0.05 |
| 75 | IPEN,  Sugiyama et al., 2014  [75] | N= 677 (urban)  Mean age: 42 ± 13 years  Women: 55%  Response rate: 59%  Community dwellers  Cuernavaca, Mexico | Cross-sectional  Cluster: purposive and random  Individuals: 1) households – random, 2) individuals - random  Stratification: socio-economic status and walkability  Neighborhood definition: census-tract (recruitment) | *Exposure variable*  1. Aesthetics  *Measure*: Self-report  [Neighborhood Environment Walkability Scale (NEWS)]  *Validation:* Yes | *Outcome*  Duration of recreational walking (min/week)  *Measure:* Self-report  [International Physical Activity Questionnaire (IPAQ)]  *Validation:* Yes | None | Generalized additive mixed models (GAMMS) accounting for administrative unit clustering | Gender, age, education, work status, marital status, area-level socio-economic status | *Recreational walking*  1. Aesthetics: OR=1.06, p>0.05 |
| 76 | RESIDE,  Sugiyama et al., 2015  [76] | N= 1412 (urban)  Mean age: 42 ± 12 years  Women: 68%  Response rate: 33%  Relocating adults  Perth, Australia | Cross-sectional  Cluster: Purposive  Individuals: Two-stage approach; 1) households (census); 2) individuals (random)  Stratification: Relocating development type (Livable Neighborhood, hybrid, conventional)  Neighborhood definition: housing development (recruitment level); 1600m street network buffer and 15 min walk from home (participant level) | *Exposure variable*  1. Sports field  2. Recreational facility  3. Playground  4. Trees  5. Gardens  6. Grassed areas  7. Walking paths  8. Water features  9. Wildlife  10. Amenities  11. Other infrastructure  12. Lighting  13. Disorder  14. Parking  15. Dog-related facility  16.Off-leash area for dogs  17. Adjacent destinations  18. Located on beach or river  19. Next to major roads  *Measure*: Objective  [Public Open Space Tool (POST) and GIS street network-buffer 1600m, database see Foster 2014a]  *Validation:* Yes | *Outcome*  Self-reported walking to a neighborhood point of interest  *Measure:* Self-report  [1 Item]  *Validation:* No | Stratified by point of interest presence, count, and size-weighted presence | Logistic regression accounting for clustering within developments | Age, gender, children in the household, area-level socio-economic status | *Association with walking to point of interest*  Point of interest presence  1. Sports field: OR=1.09, p>0.05  2. Recreational facility: OR=1.05, p>0.05  3. Playground: OR=1.34, p>0.05  4. Trees: OR=1.00, p>0.05  5. Gardens: OR=1.44, p>0.05  6. **Grassed areas**: OR=1.85, p<0.05  7. Walking paths: OR=1.06, p>0.05  8. Water features: OR=1.24, p>0.05  9. Wildlife: OR=1.18, p>0.05  10. **Amenities**: OR=1.58, p<0.05  11. Other infrastructure: OR=1.19, p>0.05  12. Lighting: OR=1.12, p>0.05  13. Disorder: OR=1.18, p>0.05  14. Parking: OR=1.21, p>0.05  15. **Dog-related facility**: OR=1.35, p<0.05  16. **Off-leash area for dogs**: OR=1.58, p<0.05  17. Adjacent destinations: OR=1.07, p>0.05  18. Located on beach or river: OR=1.40, p>0.05  19. Next to major roads: OR=1.13, p>0.05  Point of interest count  1. Sports field: OR=1.07, p>0.05  2. Recreational facility: OR=0.99, p>0.05  3. Playground: OR=1.01, p>0.05  4. Trees: OR=1.01, p>0.05  5. Gardens: OR=1.03, p>0.05  6. Grassed areas: OR=1.02, p>0.05  7. Walking paths: OR=1.01, p>0.05  8. Water features: OR=0.99, p>0.05  9. Wildlife: OR=1.01, p>0.05  10. Amenities: OR=1.04, p>0.05  11. **Other infrastructure**: OR=1.27, p<0.05  12. Lighting: OR=1.03, p>0.05  13. Disorder: OR=1.00, p>0.05  14. Parking: OR=1.01, p>0.05  15. **Dog-related facility**: OR=1.39, p<0.05  16. Off-leash area for dogs: OR=1.04, p>0.05  17. Adjacent destinations: OR=1.11, p>0.05  18. Located on beach or river: OR=1.35, p>0.05  19. Next to major roads: OR=1.04, p>0.05  Point of interest size-weighted presence  1. Sports field: OR=1.04, p>0.05  2. Recreational facility: OR=1.04, p>0.05  3. Playground: OR=1.02, p>0.05  4. Trees: OR=1.02, p>0.05  5. **Gardens**: OR=1.09, p<0.05  6. Grassed areas: OR=1.04, p>0.05  7. **Walking paths**: OR=1.05, p<0.05  8. **Water features**: OR=1.07, p<0.05  9. **Wildlife**: OR=1.09, p<0.05  10. Amenities: OR=1.05, p>0.05  11. Other infrastructure: OR=1.05  12. Lighting: OR=1.04, p>0.05  13. Disorder: OR=1.03, p>0.05  14. Parking: OR=1.04, p>0.05  15. **Dog-related facility**: OR=1.18, p<0.05  16. Off-leash area for dogs: OR=1.02, p>0.05  17. Adjacent destinations: OR=1.03, p>0.05  18. Located on beach or river: OR=1.02, p>0.05  19. Next to major roads: OR=1.02, p>0.05 |
| 77 | RESIDE,  Titze et al., 2010  [77] | N= 1656 (urban)  Mean age: 42 ± 12 years  Women: 59%  Response rate: 33%  Relocating adults  Perth, Australia | Cross-sectional  Cluster: Purposive  Individuals: Two-stage approach; 1) households (census); 2) individuals (random)  Stratification: Relocating development type (Livable Neighborhood, hybrid, conventional)  Neighborhood definition: housing development (recruitment level); 1600m street network buffer and 15 min walk from home (participant level) | *Exposure variable*  1. Neighborhood surroundings leafy and attractive  2. Accessible walking/biking paths  3. Presence of traffic slowing devices  4. Presence of 4-way intersections  5. Presence of alternatives routes  *Not included in the final model due to non-significance*  6. Land-use mix  7. Aesthetics  8. Traffic volume  9. Neighborhood crime  10. Number of destinations for transport cycling  11. Number of destinations for recreational cycling  12. Presence of major barriers for walking  13. Car parking access in local shopping areas  14. Lack of cul-de-sacs  15. Hilly streets  16. Slow traffic speed on most nearby streets  17. Busy streets have pedestrian crossing and traffic lights  18. Streets well lit at night  19. Sidewalks connecting cul-de-sacs  20. Short distance between intersections  *Measure*: Perceived  [Neighborhood Environment Walkability Scale (NEWS)]  *Validation:* Yes | *Outcome*  Frequency of transport-related and recreational cycling  *Measure:* Self-report [Neighborhood Physical Activity Questionnaire (NPAQ)]  *Validation:* Yes | Gender and age | Logistic regression | Age, gender, education, car availability, attitude towards cycling, perceived behavioral control for cycling | *Association with transport-related cycling*  1. **Neighborhood surroundings leafy and attractive**: OR=1.97, p<0.05  2. **Accessible walking/biking paths**: OR=1.77, p<0.05  3. **Presence of traffic slowing devices**: OR=1.63, p<0.05  4. **Presence of 4-way intersections**: OR=1.76, p<0.05  5. Presence of alternatives routes: OR=NR, p>0.05  *Association with recreational cycling*  1. Neighborhood surroundings leafy and attractive: OR=NR, p>0.05  2. Accessible walking/biking paths: OR=NR, p>0.05  3. Presence of traffic slowing devices: OR=NR, p>0.05  4. Presence of 4-way intersections: OR=NR, p>0.05  5. **Presence of alternatives routes**: OR=1.72, p>0.05  *Not included in the final model due to non-significance*  6. Land-use mix  7. Aesthetics  8. Traffic volume  9. Neighborhood crime  10. Number of destinations for transport cycling  11. Number of destinations for recreational cycling  12. Presence of major barriers for walking  13. Car parking access in local shopping areas  14. Lack of cul-de-sacs  15. Hilly streets  16. Slow traffic speed on most nearby streets  17. Busy streets have pedestrian crossing and traffic lights  18. Streets well lit at night  19. Sidewalks connecting cul-de-sacs  20. Short distance between intersections  *Interactions with gender and age not significant* |
| 78 | Steps to Healthier US,  Vest et al., 2005  [78] | N= 1635 (mixed)  Mean age: NR  Women: NR%  Response rate: 52%  Community dwellers  Travis County, Texas, USA | Cross-sectional  Cluster: none  Individuals: random  Stratification: none  Neighborhood definition: half a mile or 10-min walk | *Exposure variable*  1. Neighborhood sidewalks  2. Neighborhood safety  3. Walkable neighborhood  4. Street lighting  5. Trustable neighborhood  *Measure*: Perceived  [Behavioral Risk Factor Surveillance System]  *Validation:* Yes | *Outcome*  Leisure time physical inactivity  *Measure:* Self-report  [Behavioral Risk Factor Surveillance System Questionnaire]  *Validation:* Yes | None | Logistic regression | Gender, age, ethnicity, education | *Association with leisure-time physical inactivity*  1. No neighborhood sidewalks: OR=1.33, p>0.05  2. **Neighborhood safety** (ref. extremely safe):  **Quite safe**: OR=2.02, p<0.05  **Slightly safe**: OR=2.42, p<0.05  **Not at all safe**: OR=2.87, p<0.05  3. **Walkable neighborhood** (ref. very pleasant)  Somewhat pleasant: OR=1.40, p>0.05  **Not very/not at all pleasant**: OR=1.94, p<0.05  4. Street lighting (ref. very good):  Fair: OR=1.21, p>0.05  Poor/very poor: OR=1.17, p>0.05  5. No trustable neighborhood: OR=1.32, p>0.05 |
| 79 | NA  Zahra et al., 2022  [79] | N= 234 (mixed)  60% 15-35 years  Women: 68%  Response rate: NR  Community dwellers  Saudi Arabia | Cross-sectional  Cluster: none  Individuals: NR  Stratification: none  Neighborhood definition: none | *Exposure variable*  1. Safety  2. Physical environment healthy  3. Opportunity for leisure activities  4. Access to health services  5. Transport services  *Measure*: Perceived  [World Health Organization Quality of Life Questionnaire (WHOQOL-BREF)]  *Validation:* Yes | *Outcome*  Meeting the physical activity guidelines (600 MET-minutes+/week; (Yes/No)]  *Measure:* Self-report  [International Physical Activity Questionnaire (IPAQ)]  *Validation:* Yes | None | Binary logistic regression | Age, gender | *Association with physical activity*  1. Safety: OR=1.19, p>0.05  2. Physical environment healthy: 0.96, p>0.05  3. **Opportunity for leisure activities**: 1.36, p<0.05  4. Access to health services: 1.10, p>0.05  5. Transport services: 1.22, p>0.05 |
| 80 | Active Living Austin Research Project,  Zhong et al., 2020  [80] | N= 455 (urban)  Mean age: 73 ± 6 years  Women: 72%  Response rate: NR  Older adults  Austin, Texas, USA | Cross-sectional  Cluster: none  Individuals: convenience  Stratification: none  Neighborhood definition: none (recruitment-level) and 0.5 mile street-network-buffer (participant-level) | *Exposure variable - Perceived*  1. Neighborhood walkability , Destination accessibility  2. Benches on most of the sidewalks  3. Neighborhood aesthetics  4. Traffic safety  *Measure*: Perceived  [Neighborhood Environment Walkability Scale (NEWS)]  *Validation:* Yes  *Exposure variable - Objective*  5. Street length  6. Sidewalk length  7. No. intersections ≥3 ways  8. Density of intersections ≥3 ways  9. Number of stop signs  10. Density of stop signs  11. Number of intersections with stop signs  12. Percentage of intersections with stop signs  13: Length of high-speed street  14. Percentage of high-speed street  15. Number of transit stops  16. Density of transit stops  17. Proximity to closest transit stop  18. Proximity to closest rail station  19. Number of total transit routes  20. Area of offices  21. Percentage of offices  22. Presence of food stores  23. Distance to closest food store  23. Percentage of offices 🡪 Distribution of employment  24. Presence of religious destinations  25. Presence of sport/fitness destinations  26. Presence of greenbelts  27. Number of parks  28. Presence of trails in park  29. Proximity to closest park with water body  30. Presence of undesirable destinations  31. Tree canopy area  32. Water body presence  33. Net population density  34. Gross population density  35. Number of all development permits  36. Commercial permits  37. Residential permits  38. Walk Score  39. Transit Score  40. Bike Score  *Measure*: Perceived  [GIS (half a mile network buffer, database: Austin Open Data Portal, ESRI business analyst, Capital Area Metropolitan Planning Organization, Austin Transportation Department, Texas NAIP Imagery data, ]  *Validation:* Yes | *Outcome*  Recreational and transport-related walking [1+ days/week; (Yes/No)]  *Measure:* Self-report  [International Physical Activity Questionnaire (IPAQ)]  *Validation:* Yes | None | Logistic regression | Age, gender, ethnicity, marital status, education, income, general health condition, housing typing, having a dog in the household, employment status, daily sleep time, mobility aids, residential self-selection | *Association with transport-related walking*  Perceived  1. **Neighborhood walkability**: OR=1.43, p<0.05  2. Benches on most of the sidewalks: OR=NR, p>0.05  3. Neighborhood aesthetics: OR=NR, p>0.05  4. Traffic safety: OR=NR, p>0.05  Objective  5. **Street length**: OR=1.11, p<0.05  6. **Sidewalk length**: OR=1.06, p<0.05  7. **No. intersections ≥3 ways**: OR=1.17, p<0.05  8. **Density of intersections ≥3 ways**: OR=1.24, p<0.05  9. Number of stop signs: OR=NR, p>0.05  10. Density of stop signs: OR=NR, p>0.05  11. **Number of intersections with stop signs**: OR=1.14, p>0.05  12. Percentage of intersections with stop signs: OR=NR, p>0.05  13: Length of high-speed street: OR=NR, p>0.05  14. Percentage of high-speed street: OR=NR, p>0.05  15. Number of transit stops: OR=NR, p>0.05  16. Density of transit stops: OR=NR, p>0.05  17. Proximity to closest transit stop: OR=NR, p>0.05  18. Proximity to closest rail station: OR=NR, p>0.05  19. Number of total transit routes: OR=NR, p>0.05  20. **Area of offices** (ref 0):  0-1.5: OR=1.39, p>0.05  **≥1.5**: OR=2.09, p<0.05  21. **Percentage of offices** (ref. 0):  0-2%: OR=1.54, p>0.05  **≥2**%: OR=2.11, p<0.05  22. Presence of food stores: OR=NR, p>0.05  23. **Distance to the closest food store**: OR=0.58, p<0.05  24. **Presence of religious destinations**: OR=1.59, p<0.05  25. Presence of sport/fitness destinations: OR=NR, p>0.05  26. **Presence of greenbelts**: OR=0.58, p<0.05  27. **Number of parks**: OR=1.12, p<0.05  28. **Presence of trails in park**: OR=1.89, p<0.05  29. Proximity to closest park with water body: OR=NR, p>0.05  30. Presence of undesirable destinations: OR=NR, p>0.05  31. Tree canopy area: OR=NR, p>0.05  32. Water body presence: OR=NR, p>0.05  33. **Net population density**: OR=1.03, p<0.05  34. Gross population density: OR=NR, p>0.05  35. Number of all development permits: OR=NR, p>0.05  36. Commercial permits: OR=NR, p>0.05  37. Residential permits: OR=NR, p>0.05  38. Walk Score: OR=NR, p>0.05  39. Transit Score: OR=NR, p>0.05  40. Bike Score: OR=NR, p>0.05  *Association with recreational walking*  Perceived  1. Neighborhood walkability: OR=NR, p>0.05  2. **Benches on most of the sidewalks**: OR=1.97, p<0.05  3. Neighborhood aesthetics: OR=NR, p>0.05  4. Traffic safety: OR=NR, p>0.05  Objective  5. Street length: OR=NR, p>0.05  6. Sidewalk length: OR=NR, p>0.05  7. No. intersections ≥3 ways: OR=NR, p>0.05  8. Density of intersections ≥3 ways: OR=NR, p>0.05  9. Number of stop signs: OR=NR, p>0.05  10. Density of stop signs: OR=NR, p>0.05  11. Number of intersections with stop signs: OR=NR, p>0.05  12. Percentage of intersections with stop signs: OR=NR, p>0.05  13: Length of high-speed street: OR=NR, p>0.05  14. Percentage of high-speed street: OR=NR, p>0.05  15. Number of transit stops: OR=NR, p>0.05  16. **Density of transit stops**: OR=2.17, p<0.05  17. Proximity to closest transit stop: OR=NR, p>0.05  18. Proximity to closest rail station: OR=NR, p>0.05  19. Number of total transit routes: OR=NR, p>0.05  20. Area of offices: OR=NR, p>0.05  21. Percentage of offices: OR=NR, p>0.05  22. Presence of food stores: OR=NR, p>0.05  23. Percentage of offices: OR=NR, p>0.05  24. Presence of religious destinations: OR=NR, p>0.05  25. Presence of sport/fitness destinations: OR=NR, p>0.05  26. Presence of greenbelts: OR=NR, p>0.05  27. Number of parks: OR=NR, p>0.05  28. Presence of trails in park: OR=NR, p>0.05  29. Proximity to closest park with water body: OR=NR, p>0.05  30. Presence of undesirable destinations: OR=NR, p>0.05  31. Tree canopy area: OR=NR, p>0.05  32. Water body presence: OR=NR, p>0.05  33. Net population density: OR=NR, p>0.05  34. **Gross population density**: OR=0.93, p<0.05  35. Number of all development permits: OR=NR, p>0.05  36. Commercial permits: OR=NR, p>0.05  37. Residential permits: OR=NR, p>0.05  38. Walk Score: OR=NR, p>0.05  39. Transit Score: OR=NR, p>0.05  40. Bike Score: OR=NR, p>0.05 |
| 81 | Mueller retrospective survey [name invented],  Zhu et al., 2014  [81] | N= 449 total sample / 284 Austin sample (urban)  Age: 48 years  Women: 66%  Response rate: 36%  Relocating adults  Mueller, Texas, USA | Retrospective  Cluster: none  Individuals: convenience  Stratification: none  Neighborhood definition: Mueller | *Exposure variable:*  Moving to new urbanist-designed development (retrospective assessment of physical activity)  *Measure:* NA  *Validation:* NA | *Outcome: Pre-post changes in…*  Number of days/week ≥30 min moderate physical activity  Bicycling (min/week)  Total walking (min/week)  Walking in community (min/week)  Walking in community (min/week)  *Measure*: Self-report  [International Physical Activity Questionnaire (IPAQ), Twin Cities Survey, Active Where Survey]]  *Validated*: Yes | Stratified analysis by pre-move neighborhood’s walkability (based upon objective Walk Score), pre-move physical activity, pre-move social interaction, pre-move social cohesion, and importance of walkability in relocating to Mueller | Paired sample t-test | None | *Changes for total sample (min)*  1. **Number of days/week ≥30 min moderate physical activity**: 0.6, p<0.05  2. **Bicycling**: 9.5, p<0.05  3. **Total walking**: 32.1, p<0.05  4. **Walking in community**: 37.8, p<0.05  *Changes for sample moving from Austin*  1. **Number of days/week ≥30 min moderate physical activity**: 0.6, p<0.05  2. **Bicycling**: 10.9, p<0.05  3. **Total walking**: 31.0, p<0.05  4. **Walking in community**: 41.4, p<0.05  *Changes stratified by pre-move neighborhood’s walkability*  High walkability  1. Number of days/week ≥30 min moderate physical activity: 0.1, p>0.05  2. Bicycling: 9.1, p>0.05  3. Total walking: 2.3, p>0.05  4. Walking in community: 10.0, p>0.05  Medium walkability  1. **Number of days/week ≥30 min moderate physical activity**: 0.8, p<0.05  2. Bicycling: 1.4, p>0.05  3. **Total walking**: 39.8, p<0.05  4. **Walking in community**: 49.8, p<0.05  Low walkability  1. **Number of days/week ≥30 min moderate physical activity**: 0.7, p<0.05  2. **Bicycling**: 12.2, p<0.05  3. **Total walking**: 37.8, p<0.05  4. **Walking in community**: 55.3, p<0.05  Very low walkability  1. **Number of days/week ≥30 min moderate physical activity**: 0.8, p<0.05  2. **Bicycling**: 40.5, p<0.05  3. **Total walking**: 53.5, p<0.05  4. **Walking in community**: 54.1, p<0.05  *Changes stratified by pre-move physical activity level*  Sufficiently active  1. **Number of days/week ≥30 min moderate physical activity**: -0.9, p<0.05  2. Bicycling: 7.1, p>0.05  3. Total walking: -0.4, p>0.05  4. Walking in community: 9.8, p>0.05  Insufficiently active  1. **Number of days/week ≥30 min moderate physical activity**: 1.3, p<0.05  2. Bicycling: 13.0, p<0.05  3. **Total walking**: 47.9, p<0.05  4. **Walking in community**: 58.2, p<0.05  *Changes stratified by pre-move social interactions*  High  1. Number of days/week ≥30 min moderate physical activity: 0.2, p>0.05  2. Bicycling: 11.7, p>0.05  3. Total walking: 25.1, p>0.05  4. **Walking in community**: 30.3, p<0.05  Medium  1. **Number of days/week ≥30 min moderate physical activity**: 0.6, p<0.05  2. **Bicycling**: 7.9, p<0.05  3. **Total walking**: 24.5, p<0.05  4. **Walking in community**: 55.4, p<0.05  Low  1. **Number of days/week ≥30 min moderate physical activity**: 0.9, p<0.05  2. Bicycling: 12.4, p>0.05  3. **Total walking**: 43.9, p<0.05  4. **Walking in community**: 38.9, p<0.05  *Changes stratified by pre-move neighborhood cohesion*  High  1. Number of days/week ≥30 min moderate physical activity: 0.3, p>0.05  2. Bicycling: 7.8, p>0.05  3. **Total walking**: 29.8, p<0.05  4. **Walking in community**: 27.3, p<0.05  Medium  1. **Number of days/week ≥30 min moderate physical activity**: 0.6, p<0.05  2. Bicycling: 6.7, p>0.05  3. **Total walking**: 27.3, p<0.05  4. **Walking in community**: 46.1, p<0.05  Low  1. **Number of days/week ≥30 min moderate physical activity**: 0.9, p<0.05  2. **Bicycling**: 19.2, p<0.05  3. **Total walking**: 37.3, p<0.05  4. **Walking in community**: 58.3, p<0.05  *Changes stratified by importance of walkability in relocating to Mueller*  Very important/important  1. **Number of days/week ≥30 min moderate physical activity**: 0.6, p<0.05  2. **Bicycling**: 16.4, p<0.05  3. **Total walking**: 39.0, p<0.05  4. **Walking in community**: 52.5, p<0.05  Moderate  1. Number of days/week ≥30 min moderate physical activity: 0.5, p>0.05  2. Bicycling: -4.8, p>0.05  3. Total walking: 11.3, p>0.05  4. **Walking in community**: 36.9, p<0.05  Little / no importance  1. Number of days/week ≥30 min moderate physical activity: 0.5, p<0.05  2. Bicycling: 10.1, p>0.05  3. Total walking: 27.7, p>0.05  4. Walking in community: 4.7, p>0.05 |
| 82 | Mueller retrospective survey [name invented],  Zhu et al., 2020  [82] | N= 446 (urban)  Age: 48 years  Women: 67%  Response rate: 36%  Relocating  Mueller, Texas, USA | Retrospective  Cluster: none  Individuals: convenience  Stratification: none  Neighborhood definition: Mueller | *Exposure variable - Perceived*  1. Number of post-move environmental barriers (unattended dogs, inadequate sidewalks, dangerous street crossings, too much traffic, lack of safe places for walking, lack of trees or shade, lack of lighting at night, lack of interesting/attractive things to see, crime concerns)  *Measure: Perceived*  [9 items)  *Validation:* No  *Exposure variable - Objective*  2. Post-move walkability  *Measure: Objective*  [Walk Score, 2014]  *Validation:* Yes | *Outcome:*  Post-move neighborhood walking  *Measure*: Self-report  [International Physical Activity Questionnaire (IPAQ), Twin Cities Survey, Active Where Survey]]  *Validated*: Yes | None | Structural equation modeling | Gender, age, ethnicity, education | *Association with post-move neighborhood walking*  Perceived  1. Number of post-move environmental barriers: β= -0.05, p=0.05  Objective  2. **Post-move walkability**: β = 0.05, p<0.05 |
| 83 | Arizona neighborhood study [name invented]  Zuniga-Teran et al., 2017a  [83] | N= 380 (urban)  46% 60+ years  Women: 88%  Response rate: NR / 30%  Community dwellers  Tucson, Arizona, USA | Cross-sectional  Cluster: purposive  Individuals: convenience and random  Stratification: neighborhood design  Neighborhood definition: living within neighborhood design type | *Exposure variable*  Neighborhood design type  1. Traditional (high connectivity, land use, traffic safety, surveillance, green space, and community), Disaster mitigation, Desirability  2. Cluster housing (high density and experience), Desirability  3. Suburbs/conventional (no land use combination, single family housing, focus on undeveloped land) , Density  4. Enclosed (gated communities)  5. Presence of trees  *Measure*: Perceived  [Aerial image shown to participants and Neighborhood Environment Walkability Scale (NEWS) stratified by neighborhood design type]  *Validation:* Yes | *Outcome*  Total physical activity  Recreational and transport-related walking  *Measure:* Self-report  [International Physical Activity Questionnaire (IPAQ)]  *Validation:* Yes | None | Mixed models account for recruitment method (not for neighborhood clustering) | None | *Association between neighborhood design type and physical activity: R=0.05, p<0.05*  1. **Traditional**: 0.52  2. Suburbs: 0.42  3. Enclosed: 0.46  4. Cluster: 0.43  *Association between neighborhood design type and recreational walking*: *R=0.02, p<0.05*  1. **Traditional**: 0.51  2. Suburbs: 0.40  3. Enclosed: 0.48  4. Cluster: 0.47  *Association between neighborhood design type and transport-related walking*: *R=0.05, p<0.05*  1. **Traditional**: 0.47  2. Suburbs: 0.39  3. Enclosed: 0.35  4. Cluster: 0.36  *Association between trees and physical activity*  5. **Trees**: R = 0.06, p<0.05 |
| 84 | Arizona neighborhood study [name invented]  Zuniga-Teran et al., 2017b  [84] | N= 486 (urban)  21% 60-69 years  Women: 88%  Response rate: NR / 30%  Community dwellers  Tucson, Arizona, USA | Cross-sectional  Cluster: purposive  Individuals: convenience and random  Stratification: neighborhood design  Neighborhood definition: neighborhood design type (recruitment) | *Exposure variable*  1. Connectivity  2. Density  3. Land use  4. Traffic safety  5. Surveillance  6. Experience, Design, Disaster mitigation  7. Green space, Destination accessibility  8. Community, Destination proximity  9. Walkability index  *Measure*: Perceived  [Neighborhood Environment Walkability Scale (NEWS) and elements from the Walkability Index]  *Validation:* Yes | *Outcome*  Physical activity index (walking to green space, for transportation, and days with walking)  Recreational walking  Transport-related walking  *Measure:* Self-report  [International Physical Activity Questionnaire (IPAQ) and items invented]  *Validation:* Yes | None | Pearson correlation analysis | None | *Physical activity index*  1. **Connectivity**: r=0.26, p<0.05  2. **Density:** r=0.47, p<0.05  3. **Land use**: r=0.51, p<0.05  4. **Traffic safety:** 0.64, p<0.05  5. **Surveillance**: r=0.31, p<0.05  6. **Experience**: r=0.61, p<0.05  7. **Green space**: r=0.65, p<0.05  8. **Community**: r=0.18, p<0.05  9. **Walkability index**: r=0.39, p<0.05  *Recreational walking*  1. **Connectivity**: r=0.17, p<0.05  2. **Density:** r=0.45, p<0.05  3. **Land use**: r=0.44, p<0.05  4. **Traffic safety:** 0.60, p<0.05  5. **Surveillance**: r=0.19, p<0.05  6. **Experience**: r=0.61, p<0.05  7. **Green space**: r=0.65, p<0.05  8. **Community**: r=0.12, p<0.05  9. **Walkability index**: r=0.27, p<0.05  *Transport-related walking*  1. **Connectivity**: r=0.28, p<0.05  2. Density: r= -0.04, p>0.05  3. **Land use**: r=0.24, p<0.05  4. **Traffic safety:** 0.21, p<0.05  5. **Surveillance**: r=0.26, p<0.05  6. Experience: r=0.04, p>0.05  7. **Green space**: r=0.11, p<0.05  8. **Community**: r=0.20, p<0.05  9. **Walkability index**: r=0.32, p<0.05 |

**References**

1. Acheampong RA, Siiba A. Examining the determinants of utility bicycling using a socio-ecological framework: An exploratory study of the Tamale Metropolis in Northern Ghana. J Transp Geogr. 2018;69(NA):1-10. doi:10.1016/j.jtrangeo.2018.04.004

2. Adeniyi AF, Chedi H. Levels and predictors of physical activity in a sample of pre-retirement and retired civil servants in Nigeria. East Afr J Public Health. 2010;7(2):140-3. doi:10.4314/eajph.v7i2.64713

3. Aliyas Z. Built environment correlates of walking for recreation or exercise. Journal of Public Health. 2018;27(3):349-56. doi:10.1007/s10389-018-0956-y

4. Aliyas Z. Why Some Walk and Others Don't: Neighborhood Safety and the Sociodemographic Variation Effect on Walking for Leisure and Transportation. Journal of public health management and practice : JPHMP. 2020;26(4):E24-E32. doi:10.1097/phh.0000000000000992

5. AlKheder S, Alkandriy F, Alkhames Z, Habeeb M, Alenezi R, Al Kader A. Walkability, risk perception and safety assessment among urban college pedestrians in Kuwait. Transportation Research Part F: Traffic Psychology and Behaviour. 2022;86:10-32. doi:10.1016/j.trf.2022.02.003

6. Alqahtani AS, Baattaiah BA, Alharbi MD, Khan F, Aldhahi MI. Barriers and facilitators affecting physical activity among adults in Saudi Arabia during COVID-19 quarantine. Health Promot Int. 2021. doi:10.1093/heapro/daab191

7. Arasan VT, Rengaraju VR, Rao KVK. Trip characteristics of travelers without vehicles. Journal of Transportation Engineering. 1996;122(1):76-81. doi:10.1061/(ASCE)0733-947X(1996)122:1(76)

8. Awadalla NJ, Aboelyazed AE, Hassanein MA, Khalil SN, Aftab R, Gaballa II, et al. Assessment of physical inactivity and perceived barriers to physical activity among health college students, south-western Saudi Arabia. Eastern Mediterranean Health Journal. 2014;20(10):596-604. doi:10.26719/2014.20.10.596

9. Badland H, Knuiman M, Hooper P, Giles-Corti B. Socio-ecological predictors of the uptake of cycling for recreation and transport in adults: results from the RESIDE study. Prev Med. 2013;57(4):396-9. doi:10.1016/j.ypmed.2013.06.015

10. Bartshe M, Coughenour C, Pharr J. Perceived walkability, social capital, and self-reported physical activity in Las Vegas college students. Sustainability (Switzerland). 2018;10(9). doi:10.3390/su10093023

11. Bartshe M, Coughenour C, Stephen H. The relationship between tree canopy and social capital on physical activity in college students. J Am Coll Health. 2021:1-10. doi:10.1080/07448481.2021.1947299

12. Beenackers MA, Foster S, Kamphuis CB, Titze S, Divitini M, Knuiman M, et al. Taking up cycling after residential relocation: built environment factors. Am J Prev Med. 2012;42(6):610-5. doi:10.1016/j.amepre.2012.02.021

13. Bungum TJ, Landers M, Azzarelli M, Moonie S. Perceived environmental physical activity correlates among Asian Pacific Islander Americans. J Phys Act Health. 2012;9(8):1098-104. doi:10.1123/jpah.9.8.1098

14. Calise TV, Dumith SC, Dejong W, Kohl HW, 3rd. The effect of a neighborhood built environment on physical activity behaviors. J Phys Act Health. 2012;9(8):1089-97. doi:10.1123/jpah.9.8.1089

15. Calise TV, Heeren T, DeJong W, Dumith SC, Kohl HW, 3rd. Do neighborhoods make people active, or do people make active neighborhoods? Evidence from a planned community in Austin, Texas. Prev Chronic Dis. 2013;10:E102. doi:10.5888/pcd10.120119

16. Cao X, Handy SL, Mokhtarian PL. The influences of the built environment and residential self-selection on pedestrian behavior: Evidence from Austin, TX. Transportation. 2006;33(1):1-20. doi:10.1007/s11116-005-7027-2

17. Christian H, Bull F, Middleton N, Knuiman M, Divitini ML, Hooper P, et al. How important is the land use mix measure in understanding walking behaviour? Results from the RESIDE study. Int J Behav Nutr Phys Act. 2011;8(1):55-. doi:10.1186/1479-5868-8-55

18. Christian H, Knuiman M, Bull F, Timperio A, Foster S, Divitini M, et al. A new urban planning code's impact on walking: the residential environments project. Am J Public Health. 2013;103(7):1219-28. doi:10.2105/ajph.2013.301230

19. Christian H, Knuiman M, Divitini M, Foster S, Hooper P, Boruff B, et al. A Longitudinal Analysis of the Influence of the Neighborhood Environment on Recreational Walking within the Neighborhood: Results from RESIDE. Environ Health Perspect. 2017;125(7):077009. doi:10.1289/ehp823

20. Coughenour C, de la Fuente-Mella H, Paz A. Analysis of self-reported walking for transit in a sprawling urban metropolitan area in the western U.S. Sustainability (Switzerland). 2019;11(3). doi:10.3390/su11030852

21. Dellaserra CL, Crespo NC, Todd MJ, Huberty J, Vega-López S. Perceived environmental barriers and behavioral factors as possible mediators between acculturation and leisure-Time physical activity among Mexican American Adults. Journal of physical activity & health. 2018;15(9):683-91. doi:10.1123/jpah.2016-0701

22. Duncan M, Mummery K. Psychosocial and environmental factors associated with physical activity among city dwellers in regional Queensland. Prev Med. 2005;40(4):363-72. doi:10.1016/j.ypmed.2004.06.017

23. Foster S, Giles-Corti B, Knuiman M. Does Fear of Crime Discourage Walkers? A Social-Ecological Exploration of Fear As a Deterrent to Walking. Environ Behav. 2014;46(6):698-717. doi:10.1177/0013916512465176

24. Foster S, Knuiman M, Hooper P, Christian H, Giles-Corti B. Do changes in residents' fear of crime impact their walking? Longitudinal results from RESIDE. Prev Med. 2014;62:161-6. doi:10.1016/j.ypmed.2014.02.011

25. Foster S, Hooper P, Knuiman M, Christian H, Bull F, Giles-Corti B. Safe RESIDential Environments? A longitudinal analysis of the influence of crime-related safety on walking. Int J Behav Nutr Phys Act. 2016;13:22. doi:10.1186/s12966-016-0343-4

26. García-Pérez H, Lara-Valencia F. Association between neighborhood parks and leisure-time physical activity among adult mexican women. Retos. 2021(41):544-54. doi:10.47197/retos.v0i41.83409

27. Giles-Corti B, Donovan RJ. The relative influence of individual, social and physical environment determinants of physical activity. Soc Sci Med. 2002;54(12):1793-812. doi:10.1016/s0277-9536(01)00150-2

28. Giles-Corti B, Broomhall MH, Knuiman M, Collins C, Douglas K, Ng K, et al. Increasing walking: how important is distance to, attractiveness, and size of public open space? Am J Prev Med. 2005;28(2 Suppl 2):169-76. doi:10.1016/j.amepre.2004.10.018

29. Giles-Corti B, Bull F, Knuiman M, McCormack G, Van Niel K, Timperio A, et al. The influence of urban design on neighbourhood walking following residential relocation: longitudinal results from the RESIDE study. Soc Sci Med. 2013;77:20-30. doi:10.1016/j.socscimed.2012.10.016

30. Gul Y, Sultan Z, Moeinaddini M, Jokhio GA. The effects of physical activity facilities on vigorous physical activity in gated and non-gated neighborhoods. Land Use Policy. 2018;77:155-62. doi:10.1016/j.landusepol.2018.05.040

31. Gul Y, Sultan Z, Jokhio GA. The association between the perception of crime and walking in gated and non-gated neighbourhoods of Asian developing countries. Heliyon. 2018;4(8):e00715. doi:10.1016/j.heliyon.2018.e00715

32. Gul Y, Jokhio GA, Bibi T, editors. Walk towards sustainability: Improved neighbourhood street connectivity helps2020: EDP Sciences.

33. Gul Y, Jokhio GA, Sultan Z. Steps towards sustainability: Relationships between neighborhood environment and physical activity. Environment and Ecology Research. 2021;9(6):315-29. doi:10.13189/eer.2021.090601

34. Hailemariam TT, Gebregiorgis YS, Gebremeskel BF, Haile TG, Spitznagle TM. Physical activity and associated factors among pregnant women in Ethiopia: facility-based cross-sectional study. BMC Pregnancy Childbirth. 2020;20(1):92. doi:10.1186/s12884-020-2777-6

35. Handy SL. Urban form and pedestrian choices: study of Austin neighborhoods. Transp Res Rec. 1996(1552):135-44. doi:10.3141/1552-19

36. Handy SL, Clifton KJ. Local shopping as a strategy for reducing automobile travel. TRANSPORTATION. 2001;28(4):317-46. doi:10.1023/A:1011850618753

37. Heredia NI, Xu T, Lee M, McNeill LH, Reininger BM. The Neighborhood Environment and Hispanic/Latino Health. Am J Health Promot. 2022;36(1):38-45. doi:10.1177/08901171211022677

38. Holt A, Lee AH, Jancey J, Kerr D, Howat P. Are Retirement Villages Promoting Active Aging? J Aging Phys Act. 2016;24(3):407-11. doi:10.1123/japa.2015-0194

39. Hooper P, Giles-Corti B, Knuiman M. Evaluating the implementation and active living impacts of a state government planning policy designed to create walkable neighborhoods in Perth, Western Australia. Am J Health Promot. 2014;28(3 Suppl):S5-18. doi:10.4278/ajhp.130503-QUAN-226

40. Hooper P, Knuiman M, Bull F, Jones E, Giles-Corti B. Are we developing walkable suburbs through urban planning policy? Identifying the mix of design requirements to optimise walking outcomes from the 'Liveable Neighbourhoods' planning policy in Perth, Western Australia. Int J Behav Nutr Phys Act. 2015;12:63. doi:10.1186/s12966-015-0225-1

41. Hooper P, Knuiman M, Foster S, Giles-Corti B. The building blocks of a 'Liveable Neighbourhood': Identifying the key performance indicators for walking of an operational planning policy in Perth, Western Australia. Health Place. 2015;36:173-83. doi:10.1016/j.healthplace.2015.10.005

42. Jáuregui A, Pratt M, Lamadrid-Figueroa H, Hernández B, Rivera JA, Salvo D. Perceived Neighborhood Environment and Physical Activity: The International Physical Activity and Environment Network Adult Study in Mexico. Am J Prev Med. 2016;51(2):271-9. doi:10.1016/j.amepre.2016.03.026

43. Jáuregui A, Salvo D, Lamadrid-Figueroa H, Hernández B, Rivera JA, Pratt M. Perceived neighborhood environmental attributes associated with leisure-time and transport physical activity in Mexican adults. Prev Med. 2017;103s:S21-s6. doi:10.1016/j.ypmed.2016.11.014

44. Joseph RP, Vega-López S. Associations of perceived neighborhood environment and physical activity with metabolic syndrome among Mexican-Americans adults: A cross sectional examination. BMC Res Notes. 2020;13(1). doi:10.1186/s13104-020-05143-w

45. Joseph RP, Vega-López S, Han S. Physical Activity Patterns and Neighborhood Characteristics of First-Generation Latina Immigrants Living in Arizona: Cross-sectional Study. JMIR Form Res. 2021;5(5):e25663. doi:10.2196/25663

46. Khalaf A, Ekblom Ö, Kowalski J, Berggren V, Westergren A, Al-Hazzaa H. Female university students' physical activity levels and associated factors--a cross-sectional study in southwestern Saudi Arabia. Int J Environ Res Public Health. 2013;10(8):3502-17. doi:10.3390/ijerph10083502

47. Knuiman MW, Christian HE, Divitini ML, Foster SA, Bull FC, Badland HM, et al. A longitudinal analysis of the influence of the neighborhood built environment on walking for transportation: the RESIDE study. Am J Epidemiol. 2014;180(5):453-61. doi:10.1093/aje/kwu171

48. Learnihan V, Van Niel KP, Giles-Corti B, Knuiman M. Effect of Scale on the Links between Walking and Urban Design. Geographical Research. 2011;49(2):183-91. doi:10.1111/j.1745-5871.2011.00689.x

49. Manoj M, Verma A. Activity-travel behaviour of non-workers belonging to different income group households in Bangalore, India. J Transp Geogr. 2015;49:99-109. doi:10.1016/j.jtrangeo.2015.10.017

50. Manoj M, Verma A. Effect of built environment measures on trip distance and mode choice decision of non-workers from a city of a developing country, India. Transportation Research Part D: Transport and Environment. 2016;46:351-64. doi:10.1016/j.trd.2016.04.013

51. McCormack GR, Shiell A, Giles-Corti B, Begg S, Veerman JL, Geelhoed E, et al. The association between sidewalk length and walking for different purposes in established neighborhoods. Int J Behav Nutr Phys Act. 2012;9:92. doi:10.1186/1479-5868-9-92

52. Mehriar M, Masoumi H, Aslam AB, Gillani SM, Suhail T, Zulfiqar A. The relations between street network configuration and travel behavior in pakistan; the optimal level of street connectivity for a more active mobility. Applied Sciences (Switzerland). 2021;11(22). doi:10.3390/app112211015

53. Mohamed BA, Mahfouz MS, Badr MF. Physical activity and its associated factors in females with type 2 diabetes in Riyadh, Saudi Arabia. PLoS One. 2020;15(10):e0239905. doi:10.1371/journal.pone.0239905

54. Nathan A, Pereira G, Foster S, Hooper P, Saarloos D, Giles-Corti B. Access to commercial destinations within the neighbourhood and walking among Australian older adults. Int J Behav Nutr Phys Act. 2012;9:133. doi:10.1186/1479-5868-9-133

55. Nathan A, Wood L, Giles-Corti B. Examining correlates of self-reported and objectively measured physical activity among retirement village residents. Australas J Ageing. 2014;33(4):250-6. doi:10.1111/ajag.12055

56. Nathan A, Wood L, Giles-Corti B. Exploring socioecological correlates of active living in retirement village residents. J Aging Phys Act. 2014;22(1):1-15. doi:10.1123/japa.2012-0189

57. Nathan A, Wood L, Giles-Corti B. Perceptions of the Built Environment and Associations With Walking Among Retirement Village Residents. Environ Behav. 2014;46(1):46-69. doi:10.1177/0013916512450173

58. Obaid L, Hamad K. Modelling Mode Choice at Sharjah University City, United Arab Emirates. 2019 8TH INTERNATIONAL CONFERENCE ON TRANSPORTATION AND TRAFFIC ENGINEERING (ICTTE 2019)2020.

59. Olaru D, Curtis C. Designing tod precincts- accessibility and travel patterns. European Journal of Transport and Infrastructure Research. 2015;15(1):6-26. doi:10.18757/ejtir.2015.15.1.3052

60. Olojede O, Yoade A, Olufemi B. Determinants of walking as an active travel mode in a Nigerian city. J Transp Health. 2017;6:327-34. doi:10.1016/j.jth.2017.06.008

61. Oyeyemi AL, Adegoke BO, Sallis JF, Oyeyemi AY, De Bourdeaudhuij I. Perceived crime and traffic safety is related to physical activity among adults in Nigeria. BMC Public Health. 2012;12:294. doi:10.1186/1471-2458-12-294

62. Oyeyemi AL, Deforche B, Sallis JF, De Bourdeaudhuij I, Van Dyck D. Behavioral mediators of the association between neighborhood environment and weight status in Nigerian adults. Am J Health Promot. 2013;28(1):23-31. doi:10.4278/ajhp.120509-QUAN-244

63. Oyeyemi AY, Akinrolie O, Oyeyemi AL. Health-related physical activity is associated with perception of environmental hygiene and safety among adults in low-income neighbourhoods in Nigeria. European Journal of Physiotherapy. 2015;17(1):45-53. doi:10.3109/21679169.2014.955526

64. Oyeyemi AL, Kolo SM, Oyeyemi AY, Omotara BA. Neighborhood environmental factors are related to health-enhancing physical activity and walking among community dwelling older adults in Nigeria. Physiother Theory Pract. 2018;35(3):288-97. doi:10.1080/09593985.2018.1443187

65. Pimenta AR, Maghelal PK, Alawadi K. Are transit-adjacent developments effective neighborhood design models to help meet the recommended weekly physical activity levels? The case of Abu Dhabi. International Journal of Sustainable Transportation. 2021;15(3):163-74. doi:10.1080/15568318.2020.1718253

66. Rahul TM, Verma A. The influence of stratification by motor-vehicle ownership on the impact of built environment factors in Indian cities. J Transp Geogr. 2017;58:40-51. doi:10.1016/j.jtrangeo.2016.11.008

67. Rai R, Jongenelis MI, Jackson B, Newton RU, Pettigrew S. Exploring Factors Associated With Physical Activity in Older Adults: An Ecological Approach. J Aging Phys Act. 2018;27(3):343-53. doi:10.1123/japa.2018-0148

68. Ross A, Searle M. A Conceptual Model of Leisure Time Physical Activity, Neighborhood Environment, and Sense of Community. Environ Behav. 2019;51(6):749-81. doi:10.1177/0013916517751582

69. Ross A, Godwyll J, Searle M. Walking mediates associations between the neighborhood environment and flourishing. Wellbeing, Space and Society. 2021;2. doi:10.1016/j.wss.2020.100014

70. Salinas JJ, McDaniel M, Parra-Medina D. The Role of Social Support and the Neighborhood Environment on Physical Activity in Low-income, Mexican-American Women in South Texas. J Prev Med Public Health. 2018;51(5):234-41. doi:10.3961/jpmph.18.052

71. Salvo D, Reis RS, Stein AD, Rivera J, Martorell R, Pratt M. Characteristics of the built environment in relation to objectively measured physical activity among Mexican adults, 2011. Prev Chronic Dis. 2014;11:E147. doi:10.5888/pcd11.140047

72. Salvo D, Sarmiento OL, Reis RS, Hino AAF, Bolivar MA, Lemoine PD, et al. Where Latin Americans are physically active, and why does it matter? Findings from the IPEN-adult study in Bogota, Colombia; Cuernavaca, Mexico; and Curitiba, Brazil. Prev Med. 2017;103:S27-S33. doi:10.1016/j.ypmed.2016.09.007

73. Shuval K, Weissblueth E, Brezis M, Araida A, Dipietro L. Individual and socio-ecological correlates of physical activity among Arab and Jewish college students in Israel. J Phys Act Health. 2009;6(3):306-14. doi:10.1123/jpah.6.3.306

74. Sugiyama T, Francis J, Middleton NJ, Owen N, Giles-Corti B. Associations between recreational walking and attractiveness, size, and proximity of neighborhood open spaces. Am J Public Health. 2010;100(9):1752-7. doi:10.2105/ajph.2009.182006

75. Sugiyama T, Cerin E, Owen N, Oyeyemi AL, Conway TL, Van Dyck D, et al. Perceived neighbourhood environmental attributes associated with adults recreational walking: IPEN Adult study in 12 countries. Health Place. 2014;28:22-30. doi:10.1016/j.healthplace.2014.03.003

76. Sugiyama T, Gunn LD, Christian H, Francis J, Foster S, Hooper P, et al. Quality of Public Open Spaces and Recreational Walking. Am J Public Health. 2015;105(12):2490-5. doi:10.2105/ajph.2015.302890

77. Titze S, Giles-Corti B, Knuiman MW, Pikora TJ, Timperio A, Bull FC, et al. Associations between intrapersonal and neighborhood environmental characteristics and cycling for transport and recreation in adults: baseline results from the RESIDE study. J Phys Act Health. 2010;7(4):423-31. doi:10.1123/jpah.7.4.423

78. Vest J, Valadez A. Perceptions of neighborhood characteristics and leisure-time physical inactivity -Austin/Travis County, Texas, 2004. MMWR Morb Mortal Wkly Rep. 2005;54(37):926-8.

79. Zahra A, Hassan MS, Park JH, Hassan SU, Parveen N. Role of Environmental Quality of Life in Physical Activity Status of Individuals with and without Physical Disabilities in Saudi Arabia. Int J Environ Res Public Health. 2022;19(7). doi:10.3390/ijerph19074228

80. Zhong S, Lee C, Lee H. Community Environments That Promote Intergenerational Interactions vs. Walking Among Older Adults. Front Public Health. 2020;8:587363. doi:10.3389/fpubh.2020.587363

81. Zhu X, Yu CY, Lee C, Lu Z, Mann G. A retrospective study on changes in residents' physical activities, social interactions, and neighborhood cohesion after moving to a walkable community. Prev Med. 2014;69 Suppl 1:S93-7. doi:10.1016/j.ypmed.2014.08.013

82. Zhu X, Yu CY, Lee C, Lu Z. From Walkable Communities to Active Lifestyles: Exploring Causal Pathways through a Case Study in Austin, Texas. Journal of Planning Education and Research. 2020. doi:10.1177/0739456X19900528

83. Zuniga-Teran AA, Orr BJ, Gimblett RH, Chalfoun NV, Guertin DP, Marsh SE. Neighborhood Design, Physical Activity, and Wellbeing: Applying the Walkability Model. Int J Environ Res Public Health. 2017;14(1). doi:10.3390/ijerph14010076

84. Zuniga-Teran AA, Orr BJ, Gimblett RH, Chalfoun NV, Marsh SE, Guertin DP, et al. Designing healthy communities: Testing the walkability model. Frontiers of Architectural Research. 2017;6(1):63-73. doi:10.1016/j.foar.2016.11.005
