# Supplementary material for: A systematic review on the associations between the built environment and adult’s physical activity in global tropical and subtropical climate regions
Source: Int J Behav Nutr Phys Act. 2024 May 21;21:59. doi: 10.1186/s12966-024-01582-x (PMC11107026; doi:10.1186/s12966-024-01582-x)
Supplement: Supplementary file 6 — Additional File 6: Data synthesis for studies with stratified and moderation analysis. [file 12966_2024_1582_MOESM6_ESM.docx]

**Additional File 5: Associations by study region**

*Table A5.1 Synthesis built environment and active transport by world region*

|  |  | ***Western countries*** | | | | | | | | |  | ***Non-western countries*** | | | | | | | | |
| --- | --- | --- | --- | --- | --- | --- | --- | --- | --- | --- | --- | --- | --- | --- | --- | --- | --- | --- | --- | --- |
| *11D-category* | *Sub-category* | ***Perceived*** | | | ***Objective*** | | | ***Total*** | | |  | ***Perceived*** | | | ***Objective*** | | | ***Total*** | | |
|  |  | *+* | *0* | *-* | *+* | *0* | *-* | *+* | *0* | *-* |  |  |  |  |  |  |  |  |  |  |
| Demand  management |  |  | 2 | 1 |  |  |  | ***0*** | ***2*** | ***1*** |  |  |  |  |  |  |  | ***0*** | ***0*** | ***0*** |
| Density |  | 1 | 2 |  | 4 | 13 | 1 | ***5*** | ***15*** | ***1*** |  |  | 3 |  |  | 5 |  | ***0*** | ***8*** | ***0*** |
| Design | Connectivity | 2 | 10 | 1 | 5 | 8 |  |  |  |  |  |  | 5 |  | 2 |  |  |  |  |  |
|  | Walking/cycling infrastructure | 2 | 15 |  | 4 | 6 |  |  |  |  |  |  | 6 |  |  | 2 |  |  |  |  |
|  | Lot layout |  |  |  | 2 | 2 |  |  |  |  |  |  |  |  |  |  |  |  |  |  |
|  | *Total favorable features* | *4* | *25* | *1* | *11* | *16* | *0* | *15* | *41* | *1* |  |  | *11* |  | *2* | *2* |  | *2* | *13* | *0* |
|  | Unfavorable connectivity features |  |  | 2 | 1 | 11 |  |  |  |  |  |  |  |  |  |  |  |  |  |  |
|  | Unfavorable walking/cycling infrastructure |  | 3 |  |  |  |  |  |  |  |  |  |  |  |  |  |  |  |  |  |
|  | *Total unfavorable features* | *0* | *3* | *2* | *1* | *11* | *0* | *1* | *14* | *2* |  |  |  |  |  |  |  | *0* | *0* | *0* |
|  | ***Design total*** |  |  |  |  |  |  | ***17*** | ***55*** | ***2*** |  |  |  |  |  |  |  | ***2*** | ***13*** | ***0*** |
| Desirability | General safety | 1 | 4 |  |  |  |  |  |  |  |  | 1 |  | 1 |  |  |  |  |  |  |
|  | Crime safety |  | 6 |  |  |  |  |  |  |  |  | 2 | 4 |  |  |  |  |  |  |  |
|  | Traffic safety | 4 | 14 |  |  | 3 |  |  |  |  |  | 2 | 4 |  |  |  |  |  |  |  |
|  | Aesthetics |  | 14 |  |  |  |  |  |  |  |  | 1 | 2 |  |  |  |  |  |  |  |
|  | *Total favorable features* | *5* | *38* | *0* | *0* | *3* | *0* | *5* | *41* | *0* |  | *6* | *10* | *1* |  |  |  | *6* | *10* | *1* |
|  | Compromised general safety |  | 1 |  |  |  |  |  |  |  |  |  | 1 |  |  |  |  |  |  |  |
|  | Criminality and crime concerns |  | 13 | 1 |  | 3 |  |  |  |  |  |  | 1 |  |  |  |  |  |  |  |
|  | Traffic hazards and concerns |  | 5 | 1 |  | 3 |  |  |  |  |  |  | 4 | 2 |  |  |  |  |  |  |
|  | Unfavorable aesthetics |  | 2 |  |  |  |  |  |  |  |  |  |  |  |  |  |  |  |  |  |
|  | *Total unfavorable features* |  | *21* | *2* |  | *6* |  | *0* | *27* | *2* |  | *0* | *6* | *2* |  |  |  | *0* | *6* | *2* |
|  | ***Desirability total*** |  |  |  |  |  |  | ***7*** | ***68*** | ***0*** |  |  |  |  |  |  |  | ***8*** | ***16*** | ***1*** |
| Destination accessibility | Destination mix | 2 | 4 |  | 1 | 4 |  |  |  |  |  | 1 | 1 |  |  |  |  |  |  |  |
|  | Parks, natural features, and public open space | 1 | 1 |  | 4 | 14 |  |  |  |  |  | 1 | 5 |  |  |  |  |  |  |  |
|  | Public transport | 3 | 1 |  | 3 | 7 |  |  |  |  |  |  |  |  |  |  |  |  |  |  |
|  | Recreational facilities |  | 2 |  |  |  |  |  |  |  |  |  |  |  | 1 |  |  |  |  |  |
|  | Shops and services for daily living | 3 | 3 |  | 2 | 2 |  |  |  |  |  | 1 | 4 |  |  |  |  |  |  |  |
|  | Walking/cycling infrastructure | 1 |  |  |  |  |  |  |  |  |  |  |  |  |  |  |  |  |  |  |
|  | Friendly topography |  | 1 | 1 |  |  |  |  |  |  |  |  |  |  |  |  |  |  |  |  |
|  | *Total favorable features* | *10* | *12* | *1* | *10* | *27* |  | *20* | *39* | *1* |  | *3* | *10* |  | *1* |  |  | *4* | *10* | *0* |
|  | Inaccessible public transport |  |  |  |  |  |  |  |  |  |  | 2 |  |  |  |  |  |  |  |  |
|  | Inaccessible walking/cycling infrastructure |  |  |  |  |  |  |  |  |  |  |  | 1 |  |  |  |  |  |  |  |
|  | Unfriendly topography |  | 3 |  |  | 1 |  |  |  |  |  |  | 3 |  |  |  |  |  |  |  |
|  | *Total* |  | *3* |  |  | *1* |  | *0* | *4* | *0* |  | *2* | *4* |  |  |  |  | *2* | *4* | *0* |
|  | ***Destination accessibility total*** |  |  |  |  |  |  | ***20*** | ***43*** | ***1*** |  |  |  |  |  |  |  | ***4*** | ***14*** | ***2*** |
| Destination proximity | Proximate destination mix | 2 |  |  | 1 |  |  |  |  |  |  |  |  |  |  |  |  |  |  |  |
|  | Proximate parks, natural features, and public open space |  |  |  | 3 | 14 |  |  |  |  |  |  | 4 |  |  |  |  |  |  |  |
|  | Proximate recreational facilities |  | 5 |  |  | 1 |  |  |  |  |  |  |  |  |  | 1 |  |  |  |  |
|  | Proximate shops and services for daily living |  | 2 |  |  | 8 |  |  |  |  |  |  |  |  |  | 3 |  |  |  |  |
|  | Proximate school/work |  |  |  |  | 3 | 1 |  |  |  |  |  |  |  |  | 1 |  |  |  |  |
|  | *Total favorable features* | *2* | *7* |  | *4* | *26* | *1* | *6* | *33* | *1* |  |  | *4* |  |  | *5* |  | *0* | *9* | *0* |
|  | Proximate unfavorable destinations |  |  |  |  | 1 |  |  |  |  |  |  |  |  |  |  |  |  |  |  |
|  | Distance to recreational facilities |  | 2 |  |  | 5 |  |  |  |  |  |  |  |  |  |  |  |  |  |  |
|  | Distance to parks, natural features, and public open space |  |  |  |  | 1 |  |  |  |  |  |  |  |  |  |  |  |  |  |  |
|  | Distance to shops and services for daily living |  |  |  |  | 3 | 2 |  |  |  |  |  |  |  |  |  |  |  |  |  |
|  | Distance to school/work |  |  |  |  | 2 |  |  |  |  |  |  |  | 1 |  |  |  |  |  |  |
|  | General travel distance or time |  |  |  |  |  |  |  |  |  |  |  |  | 3 |  |  |  |  |  |  |
|  | *Total* |  | *2* |  |  | *12* | *2* | *0* | *14* | *2* |  |  |  | *4* |  |  |  | *0* | *0* | *4* |
|  | ***Destination proximity total*** |  |  |  |  |  |  | ***8*** | ***47*** | ***1*** |  |  |  |  |  |  |  | ***4*** | ***9*** | ***0*** |
| Disaster mitigation | General greenery | 1 | 1 |  |  | 2 |  |  |  |  |  |  |  |  |  |  |  |  |  |  |
|  | Trees and shade | 1 |  |  | 1 | 4 |  |  |  |  |  |  |  |  |  |  |  |  |  |  |
|  | Parks and park area |  |  |  |  | 4 |  |  |  |  |  |  |  |  |  |  |  |  |  |  |
|  | ***Disaster mitigation total*** | *2* | *1* |  | *1* | *10* |  | ***3*** | ***11*** | ***0*** |  |  |  |  |  |  |  | ***0*** | ***0*** | ***0*** |
| Distance to public transport |  |  |  |  |  | 12 |  | ***0*** | ***12*** | ***0*** |  |  | 2 |  |  | 1 |  | ***0*** | ***3*** | ***0*** |
| Diverse housing and land use |  | *2* | *1* |  | *1* | *17* |  | ***3*** | ***18*** | ***0*** |  |  | *2* |  | *1* | *2* |  | ***1*** | ***4*** | ***0*** |
| Multi-component category | General environment supportive of physical activity | 3 | 4 |  |  | 2 |  |  |  |  |  |  |  |  |  |  |  |  |  |  |
|  | Walkability and walking-friendly environment | 4 | 2 |  | 14 | 6 |  |  |  |  |  |  |  |  |  | 1 |  |  |  |  |
|  | New urbanist designed development |  |  |  | 10 | 16 | 1 |  |  |  |  |  |  |  |  |  |  |  |  |  |
|  | *Total favorable features* | *7* | *6* | *0* | *24* | *24* | *1* | *31* | *30* | *1* |  |  |  |  |  | *1* |  | *0* | *1* | *0* |
|  | General environment compromising physical activity |  | 1 |  |  |  |  | *0* | *1* | *0* |  |  |  |  |  |  |  |  |  |  |
|  |  |  |  |  |  |  |  | ***31*** | ***31*** | ***1*** |  |  |  |  |  |  |  | ***0*** | ***1*** | ***0*** |

*Please note: “+” = positive relationship / supports of physical activity, “0” = null relationship, “-“ = negative relationship / compromises physical activity*

*Table A5.2. Synthesis built environment and recreational physical activity by world region*

|  |  | ***Western countries*** | | | | | | | | |  | ***Non-western countries*** | | | | | | | | |
| --- | --- | --- | --- | --- | --- | --- | --- | --- | --- | --- | --- | --- | --- | --- | --- | --- | --- | --- | --- | --- |
| *11D-category* | *Sub-category* | ***Perceived*** | | | ***Objective*** | | | ***Total*** | | |  | ***Perceived*** | | | ***Objective*** | | | ***Total*** | | |
|  |  | *+* | *0* | *-* | *+* | *0* | *-* | *+* | *0* | *-* |  | *+* | *0* | *-* | *+* | *0* | *-* | *+* | *0* | *-* |
| Demand  management |  |  | 3 |  |  |  |  | ***0*** | ***3*** | ***0*** |  |  |  |  |  |  |  | ***0*** | ***0*** | ***0*** |
| Density |  | *1* |  |  | *2* | *17* |  | ***3*** | ***17*** | ***0*** |  |  | *5* |  |  | *2* | *1* | ***0*** | ***7*** | ***1*** |
| Design | Connectivity | 3 | 7 | 2 | 5 | 10 |  |  |  |  |  |  | 9 |  | 1 |  |  |  |  |  |
|  | Walking/cycling infrastructure | 4 | 19 |  | 2 | 7 |  |  |  |  |  | 1 | 8 |  |  | 1 |  |  |  |  |
|  | Lot layout |  |  |  | 2 | 2 |  |  |  |  |  |  |  |  |  |  |  |  |  |  |
|  | *Total favorable features* | *7* | *26* | *2* | *9* | *19* |  | *16* | *45* | *2* |  | *1* | *17* |  | *1* | *1* |  | *2* | *18* | *0* |
|  | Unfavorable connectivity features |  |  |  |  | 11 | 1 |  |  |  |  |  |  |  |  |  |  |  |  |  |
|  | Unfavorable walking/cycling infrastructure |  | 3 | 1 |  |  |  |  |  |  |  |  |  |  |  |  |  |  |  |  |
|  | *Total unfavorable features* | *0* | *3* | *1* | *0* | *11* | *1* | *0* | *14* | *2* |  |  |  |  |  |  |  | *0* | *0* | *0* |
|  | ***Design total*** |  |  |  |  |  |  | ***18*** | ***59*** | ***2*** |  |  |  |  |  |  |  | ***2*** | ***18*** | ***0*** |
| Desirability | General safety | 7 | 11 |  |  | 2 |  |  |  |  |  | 1 | 1 |  |  |  |  |  |  |  |
|  | Crime safety | 3 | 4 | 1 |  |  |  |  |  |  |  | 2 | 5 | 1 |  |  |  |  |  |  |
|  | Traffic safety | 3 | 18 |  |  | 3 |  |  |  |  |  | 2 | 4 |  |  |  |  |  |  |  |
|  | Aesthetics | 5 | 14 |  |  |  |  |  |  |  |  | 3 | 2 |  |  |  |  |  |  |  |
|  | *Total favorable features* | *18* | *47* | *1* | *0* | *5* | *0* | *18* | *52* | *1* |  | *8* | *12* | *1* |  |  |  | *8* | *12* | *1* |
|  | Compromised general safety |  | 3 |  |  |  |  |  |  |  |  |  |  |  |  |  |  |  |  |  |
|  | Criminality and crime concerns |  | 14 | 1 |  | 3 |  |  |  |  |  |  | 1 |  |  |  |  |  |  |  |
|  | Traffic hazards and concerns |  | 6 | 3 | 1 | 1 |  |  |  |  |  |  | 2 | 1 |  |  |  |  |  |  |
|  | Unfavorable aesthetics |  | 1 | 5 |  |  |  |  |  |  |  |  |  |  |  |  |  |  |  |  |
|  | *Total* | *0* | *24* | *6* | *1* | *4* | *0* | *1* | *28* | *5* |  |  | *3* | *1* |  |  |  | *0* | *3* | *1* |
|  | ***Desirability total*** |  |  |  |  |  |  | ***23*** | ***80*** | ***2*** |  |  |  |  |  |  |  | ***9*** | ***15*** | ***1*** |
| Destination accessibility | Destination mix | 2 | 3 |  | 1 | 3 |  |  |  |  |  | 5 | 3 | 1 |  |  |  |  |  |  |
|  | Parks, natural features, and public open space | 1 | 2 |  | 5 | 14 | 1 |  |  |  |  |  | 12 |  |  |  |  |  |  |  |
|  | Public transport |  | 1 |  |  | 8 |  |  |  |  |  |  |  |  |  |  |  |  |  |  |
|  | Recreational facilities |  | 2 |  |  |  |  |  |  |  |  |  |  |  | 1 |  |  |  |  |  |
|  | Shops and services for daily living | 1 | 5 |  | 2 | 2 |  |  |  |  |  | 1 | 5 |  |  |  |  |  |  |  |
|  | Walking/cycling infrastructure |  | 2 |  |  |  |  |  |  |  |  |  |  |  |  |  |  |  |  |  |
|  | Friendly topography |  | 2 |  |  |  |  |  |  |  |  |  |  |  |  |  |  |  |  |  |
|  | *Total favorable features* | *4* | *18* |  | *8* | *27* | *1* | *12* | *45* | *1* |  | *6* | *20* | *1* | *1* |  |  | *7* | *20* | *1* |
|  | Inaccessible parks, natural features, and public open space |  |  |  |  |  | 1 |  |  |  |  |  |  |  |  |  |  |  |  |  |
|  | Unfriendly topography |  | 4 |  |  | 1 |  |  |  |  |  |  | 4 |  |  |  |  |  |  |  |
|  | *Total unfavorable features* |  | *4* |  |  | *1* | *1* | *0* | *5* | *1* |  |  | *4* |  |  |  |  | *0* | *4* | *0* |
|  | ***Destination accessibility*** |  |  |  |  |  |  | ***13*** | ***50*** | ***1*** |  |  |  |  |  |  |  | ***7*** | ***24*** | ***1*** |
| Destination proximity | Proximate destination mix | 1 | 2 |  | 1 | 1 |  |  |  |  |  |  |  |  |  |  |  |  |  |  |
|  | Proximate parks, natural features, and public open space | 4 |  |  | 2 | 22 | 1 |  |  |  |  |  | 8 |  |  |  |  |  |  |  |
|  | Proximate school/work |  |  |  |  | 4 |  |  |  |  |  |  |  |  | 1 |  |  |  |  |  |
|  | Proximate recreational facilities | 1 | 5 |  | 1 |  |  |  |  |  |  |  |  |  |  | 1 |  |  |  |  |
|  | Proximate shops and services for daily living |  | 2 | 1 |  | 8 |  |  |  |  |  |  |  |  | 2 | 1 |  |  |  |  |
|  | *Total favorable features* | *6* | *9* | *1* | *4* | *35* | *1* | *10* | *44* | *2* |  |  | *8* |  | *3* | *2* |  | *3* | *10* | *0* |
|  | Travel time or distance |  |  |  |  |  |  |  |  |  |  |  |  | 1 |  |  |  |  |  |  |
|  | Proximate unfavorable destination mix |  |  |  |  | 1 |  |  |  |  |  |  |  |  |  |  |  |  |  |  |
|  | Distance to destination mix |  |  |  |  | 1 |  |  |  |  |  |  |  |  |  |  |  |  |  |  |
|  | Distance to parks, natural features, and public open space |  |  |  |  | 2 |  |  |  |  |  |  |  |  |  |  |  |  |  |  |
|  | Distance to recreational facilities |  | 1 |  | 1 | 4 |  |  |  |  |  |  |  |  |  |  |  |  |  |  |
|  | Distance to school/work |  |  |  |  | 2 |  |  |  |  |  |  |  |  |  |  |  |  |  |  |
|  | Distance to shops and services for daily living |  |  |  |  | 4 | 1 |  |  |  |  |  |  |  |  |  |  |  |  |  |
|  | Distance to walking/cycling infrastructure |  |  |  |  |  | 1 |  |  |  |  |  |  |  |  |  |  |  |  |  |
|  | *Total unfavorable features* |  | *1* |  | *1* | *14* | *2* | *1* | *15* | *2* |  |  |  | *1* |  |  |  | *0* | *0* | *1* |
|  | ***Destination proximity total*** |  |  |  |  |  |  | ***12*** | ***59*** | ***3*** |  |  |  |  |  |  |  | ***4*** | ***10*** | ***0*** |
| Disaster mitigation | General greenery |  | 3 |  |  | 2 |  |  |  |  |  |  |  |  |  |  |  |  |  |  |
|  | Trees and shade | 1 | 1 |  |  | 5 |  |  |  |  |  |  |  |  |  |  |  |  |  |  |
|  | Parks and park area |  |  |  |  | 4 |  |  |  |  |  |  |  |  |  |  |  |  |  |  |
|  | Unspecified |  | 1 |  |  |  |  |  |  |  |  |  |  |  |  |  |  |  |  |  |
|  | ***Disaster mitigation total*** | *1* | *5* |  |  | *11* |  | ***1*** | ***16*** | ***0*** |  |  |  |  |  |  |  | ***0*** | ***0*** | ***0*** |
| Distance to public transport |  |  |  |  |  | 11 |  | ***0*** | ***11*** | ***0*** |  |  | 4 |  |  | 1 |  | ***0*** | ***5*** | ***0*** |
| Diverse housing and land use |  |  | *1* |  |  | *18* |  | ***0*** | ***19*** | ***0*** |  |  | *4* |  |  | *2* |  | ***0*** | ***6*** | ***0*** |
| Multi-component category | General environment supportive of physical activity | 9 | 2 |  |  | 4 |  |  |  |  |  |  |  |  |  |  |  |  |  |  |
|  | Walkability and walking-friendly environment | 2 | 1 |  | 4 | 14 | 1 |  |  |  |  |  |  |  |  | 1 |  |  |  |  |
|  | New urbanist designed development |  |  |  | 10 | 16 |  |  |  |  |  |  |  |  |  |  |  |  |  |  |
|  | *Total favorable features* | *11* | *3* |  | *14* | *34* | *1* | *25* | *37* | *1* |  |  |  |  |  | 1 |  | *0* | *1* | *0* |
|  | General environment compromising physical activity | 1 | 1 |  |  |  |  | *1* | *1* | *0* |  |  |  |  |  |  |  | *0* | *0* | *0* |
|  | **Multicomponent category total** |  |  |  |  |  |  | ***25*** | ***38*** | ***2*** |  |  |  |  |  |  |  | ***0*** | ***1*** | ***0*** |

*Please note: “+” = positive relationship / increased physical activity, “0” = null relationship, “-“ = negative relationship / compromised physical activity*

*Table A5.3. Synthesis built environment and total walking and cycling / general physical activity by world region*

|  |  | ***Western countries*** | | | | | | | | |  | ***Non-western countries***  ***Asia, Africa, Latin America and Caribbean*** | | | | | | | | |
| --- | --- | --- | --- | --- | --- | --- | --- | --- | --- | --- | --- | --- | --- | --- | --- | --- | --- | --- | --- | --- |
| *11D-category* | *Sub-category* | ***Perceived*** | | | ***Objective*** | | | ***Total*** | | |  | ***Perceived*** | | | ***Objective*** | | | ***Total*** | | |
|  |  | *+* | *0* | *-* | *+* | *0* | *-* | *+* | *0* | *-* |  | *+* | *0* | *-* | *+* | *0* | *-* | *+* | *0* | *-* |
| Density |  | *1* |  |  | *1* | *15* | *0* | ***2*** | ***15*** | ***0*** |  | *2* |  |  |  |  |  | ***2*** | ***0*** | ***0*** |
| Design | Connectivity | 1 | 1 |  |  | 11 |  |  |  |  |  | 2 | 1 |  |  |  |  |  |  |  |
|  | Walking/cycling infrastructure | 1 | 6 |  | 1 | 7 |  |  |  |  |  |  | 1 |  |  |  |  |  |  |  |
|  | Lot layout |  |  |  | 3 | 3 |  |  |  |  |  |  |  |  |  |  |  |  |  |  |
|  | *Total favorable features* | *2* | *7* | *0* | *4* | *21* | *0* | *6* | *28* | *0* |  | *2* | *2* |  |  |  |  | *2* | *2* | *0* |
|  | Unfavorable connectivity features |  |  |  | 1 | 17 |  |  |  |  |  |  |  |  |  |  |  |  |  |  |
|  | Unfavorable walking/cycling infrastructure |  | 1 |  |  |  |  |  |  |  |  |  |  |  |  |  |  |  |  |  |
|  | *Total unfavorable features* | *0* | *1* | *0* | *1* | *17* | *0* | *1* | *18* | *0* |  |  |  |  |  |  |  | *0* | *0* | *0* |
|  | ***Design total*** |  |  |  |  |  |  | ***6*** | ***46*** | ***1*** |  |  |  |  |  |  |  | ***2*** | ***2*** | ***0*** |
| Desirability | General safety | 2 | 1 |  |  |  |  |  |  |  |  | 1 | 3 |  |  |  |  |  |  |  |
|  | Crime safety | 1 | 4 |  |  |  |  |  |  |  |  | 4 |  |  |  |  |  |  |  |  |
|  | Traffic safety | 1 | 8 |  |  |  |  |  |  |  |  |  | 4 | 2 |  |  |  |  |  |  |
|  | Aesthetics | 1 | 5 |  |  | 1 |  |  |  |  |  | 4 |  |  |  |  |  |  |  |  |
|  | *Total favorable features* | *5* | *18* | *0* | *0* | *1* | *0* | *5* | *19* | *0* |  | *9* | *7* | *2* |  |  |  | *9* | *7* | *2* |
|  | Compromised general safety |  | 1 |  |  |  |  |  |  |  |  |  |  |  |  |  |  |  |  |  |
|  | Criminality and crime concerns |  | 5 | 1 |  | 1 |  |  |  |  |  |  |  |  |  |  |  |  |  |  |
|  | Traffic hazards and concerns |  | 1 |  |  | 2 |  |  |  |  |  |  | 2 | 1 |  |  |  |  |  |  |
|  | Unfavorable aesthetics |  | 1 |  |  |  |  |  |  |  |  |  |  |  |  |  |  |  |  |  |
|  | *Total unfavorable features* |  | *8* | *1* |  | *3* |  | *0* | *11* | *1* |  |  | *2* | *1* |  |  |  | *0* | *2* | *1* |
|  | ***Desirability total*** |  |  |  |  |  |  | ***6*** | ***30*** | ***0*** |  |  |  |  |  |  |  | ***10*** | ***9*** | ***2*** |
| Destination accessibility | Destination mix |  |  |  |  |  |  |  |  |  |  | 2 |  |  |  |  |  |  |  |  |
|  | Parks, natural features, and public open space |  |  |  | 3 | 23 |  |  |  |  |  |  |  |  |  | 4 |  |  |  |  |
|  | Public transport |  |  |  |  | 7 |  |  |  |  |  |  |  |  |  |  |  |  |  |  |
|  | Recreational facilities |  | 2 |  |  | 1 |  |  |  |  |  | 1 |  |  |  |  |  |  |  |  |
|  | Shops and services for daily living |  | 3 |  | 1 | 2 |  |  |  |  |  | 1 | 2 |  |  |  |  |  |  |  |
|  | Friendly topography |  | 2 |  |  |  |  |  |  |  |  |  |  |  |  |  |  |  |  |  |
|  | *Total favorable features* |  | *7* |  | *4* | *33* |  | *4* | *40* | *0* |  | *4* | *2* |  |  | *4* |  | *4* | *6* | *0* |
|  | Inaccessible shops and services for daily living |  | 1 |  |  |  |  |  |  |  |  |  |  |  |  |  |  |  |  |  |
|  | Unfriendly topography |  | 1 |  |  | 1 |  |  |  |  |  |  |  |  |  |  |  |  |  |  |
|  | *Total* |  | *2* |  |  | *1* |  | *0* | *3* | *0* |  |  |  |  |  |  |  | *0* | *0* | *0* |
|  | ***Destination accessibility total*** |  |  |  |  |  |  | ***4*** | ***43*** | ***0*** |  |  |  |  |  |  |  | ***4*** | ***6*** | ***0*** |
| Destination proximity | Proximate destination mix | 1 | 1 |  |  | 9 |  |  |  |  |  |  |  |  |  |  |  |  |  |  |
|  | Proximate parks, natural features, and public open space |  |  |  | 2 | 22 |  |  |  |  |  | 1 |  | 1 |  |  |  |  |  |  |
|  | Proximate school/work |  |  |  |  | 5 | 1 |  |  |  |  |  |  |  |  | 1 |  |  |  |  |
|  | Proximate recreational facilities |  | 7 |  |  |  |  |  |  |  |  |  |  |  |  | 1 |  |  |  |  |
|  | Proximate shops and services for daily living |  | 3 |  | 1 | 26 | 2 |  |  |  |  |  | 2 |  | 1 | 1 | 1 |  |  |  |
|  | *Total favorable features* | *1* | *11* |  | *3* | *62* | *3* | *4* | *73* | *3* |  | *1* | *2* | *1* | *1* | *3* | *1* | *2* | *5* | *2* |
|  | Distance to recreational facilities |  |  |  |  | 6 |  |  |  |  |  |  |  |  |  |  |  |  |  |  |
|  | Distance to parks, natural features, and public open space |  |  |  |  |  |  |  |  |  |  |  |  |  |  | 2 |  |  |  |  |
|  | Distance to shops and services for daily living |  |  |  |  | 2 |  |  |  |  |  |  |  | 1 |  |  |  |  |  |  |
|  | Distance to school/work |  |  |  |  | 3 |  |  |  |  |  |  |  |  |  |  |  |  |  |  |
|  | *Total unfavorable features* |  |  |  |  | *11* |  | *0* | *11* | *0* |  |  |  | *1* |  | *2* |  | *0* | *2* | *1* |
|  | ***Destination proximity total*** |  |  |  |  |  |  | ***4*** | ***84*** | ***3*** |  |  |  |  |  |  |  | ***3*** | ***7*** | ***2*** |
| Disaster mitigation | Trees and shade | *1* |  |  | *1* | *5* |  |  |  |  |  |  |  |  |  |  |  |  |  |  |
|  | Parks and park area |  |  |  |  | *6* |  |  |  |  |  |  |  |  |  | *2* |  |  |  |  |
|  | ***Disaster mitigation total*** | *1* |  |  | *1* | *11* |  | ***2*** | ***11*** | ***0*** |  |  |  |  |  | *2* |  | ***0*** | ***2*** | ***0*** |
| Distance to public transport |  |  |  |  |  | 13 |  | ***0*** | ***13*** | ***0*** |  |  |  |  |  | 1 |  | ***0*** | ***1*** | ***0*** |
| Diverse housing and land use |  |  |  |  |  | *15* |  | ***0*** | ***15*** | ***0*** |  |  |  |  |  |  |  | ***0*** | ***0*** | ***0*** |
| Multi-component category | General environment supportive of physical activity | 6 | 2 |  |  | 1 |  |  |  |  |  |  |  |  |  |  |  |  |  |  |
|  | Walkability and walking-friendly environment | 2 |  |  | 3 | 8 |  |  |  |  |  |  |  |  |  | 1 |  |  |  |  |
|  | New urbanist designed development |  |  |  | 6 | 6 |  |  |  |  |  |  |  |  |  |  |  |  |  |  |
|  | *Total favorable features* | *8* | *2* | *0* | *9* | *15* | *0* | *17* | *17* | *0* |  |  |  |  |  | 1 |  | *0* | *1* | *0* |
|  | General environment compromising physical activity |  | 1 |  |  |  |  | *0* | *1* | *0* |  |  |  |  |  |  |  | *0* | *0* | *0* |
|  | ***Multicomponent category total*** |  |  |  |  |  |  | ***17*** | ***18*** | ***0*** |  |  |  |  |  |  |  | ***0*** | ***1*** | ***0*** |

*Please note: “+” = positive relationship / increased physical activity, “0” = null relationship, “-“ = negative relationship / compromised physical activity*

*Table A5.4. Synthesis built environment and MVPA stratified by western and non-western study regions*

|  |  | ***Western countries*** | | | | | | | | |  | ***Asia, Africa, Latin America and Caribbean*** | | | | | | | | |
| --- | --- | --- | --- | --- | --- | --- | --- | --- | --- | --- | --- | --- | --- | --- | --- | --- | --- | --- | --- | --- |
| *11D-category* | *Sub-category* | ***Perceived*** | | | ***Objective*** | | | ***Total*** | | |  | ***Perceived*** | | | ***Objective*** | | | ***Total*** | | |
|  |  | *+* | *0* | *-* | *+* | *0* | *-* | *+* | *0* | *-* |  | *+* | *0* | *-* | *+* | *0* | *-* | *+* | *0* | *-* |
| Demand management |  |  | *1* |  |  |  |  | ***0*** | ***1*** | ***0*** |  |  |  |  |  |  |  | ***0*** | ***0*** | ***0*** |
| Density |  |  | *1* |  |  |  |  | ***0*** | ***1*** | ***0*** |  |  | *4* | *1* |  | *3* | *4* | ***0*** | ***7*** | ***5*** |
| Design | Connectivity |  | 2 |  |  |  |  |  |  |  |  | 2 | 6 | 1 | 1 | 6 | 1 |  |  |  |
|  | Walking/cycling infrastructure |  | 5 |  |  |  |  |  |  |  |  | 1 | 9 |  |  |  |  |  |  |  |
|  | *Total favorable features* | *0* | *9* | *0* | *0* | *0* | *0* | *0* | *9* | *0* |  | *3* | *15* | *1* | *1* | *6* | *1* | *4* | *21* | *2* |
|  | Unfavorable walking/cycling infrastructure |  | 2 |  |  |  |  | *0* | *2* | *0* |  |  | 3 |  |  |  |  | *0* | *3* | *0* |
|  | ***Design total*** |  |  |  |  |  |  | ***0*** | ***11*** | ***0*** |  |  |  |  |  |  |  | ***4*** | ***24*** | ***2*** |
| Desirability | General safety | 1 | 4 |  |  | 2 |  |  |  |  |  |  | 7 |  |  |  |  |  |  |  |
|  | Crime safety |  | 3 |  |  |  |  |  |  |  |  | 3 | 1 | 1 |  |  |  |  |  |  |
|  | Traffic safety |  | 5 |  |  |  |  |  |  |  |  | 2 | 6 |  |  |  |  |  |  |  |
|  | Aesthetics | 2 | 4 |  |  |  |  |  |  |  |  | 3 | 4 |  |  |  |  |  |  |  |
|  | *Total favorable features* | *3* | *16* | *0* | *0* | *2* | *0* | *3* | *18* | *0* |  | *8* | *18* | *1* | *0* | *0* | *0* | *8* | *18* | *1* |
|  | Compromised general safety |  | 4 | 1 |  |  |  |  |  |  |  |  | 1 |  |  |  |  |  |  |  |
|  | Criminality and crime concerns |  | 3 | 1 |  |  |  |  |  |  |  |  | 5 | 3 |  |  |  |  |  |  |
|  | Traffic hazards and concerns |  | 2 |  |  | 1 |  |  |  |  |  |  | 5 | 2 |  |  |  |  |  |  |
|  | Unfavorable aesthetics |  |  |  |  |  |  |  |  |  |  |  | 1 |  |  |  |  |  |  |  |
|  | *Total unfavorable features* | *0* | *9* | *2* | *0* | *1* | *0* | *0* | *10* | *2* |  | *0* | *12* | *5* | *0* | *0* | *0* | *0* | *12* | *5* |
|  | ***Desirability total*** |  |  |  |  |  |  | ***5*** | ***28*** | ***0*** |  |  |  |  |  |  |  | ***13*** | ***30*** | ***1*** |
| Destination accessibility | Destination mix |  |  |  |  |  |  |  |  |  |  | 2 |  |  |  |  |  |  |  |  |
|  | Parks, natural features, and public open space | 1 |  |  |  | 1 |  |  |  |  |  | 2 | 10 |  |  | 3 | 1 |  |  |  |
|  | Public transport |  |  |  |  |  |  |  |  |  |  |  | 1 |  |  | 1 | 1 |  |  |  |
|  | Recreational facilities |  | 1 |  |  |  |  |  |  |  |  | 3 |  |  | 3 | 1 |  |  |  |  |
|  | Shops and services for daily living |  | 2 |  |  |  |  |  |  |  |  | 1 | 6 |  |  | 2 | 2 |  |  |  |
|  | Walking/cycling infrastructure |  | 1 |  |  |  |  |  |  |  |  |  |  |  |  |  |  |  |  |  |
|  | Friendly topography |  | 1 |  |  |  |  |  |  |  |  |  | 1 |  |  |  |  |  |  |  |
|  | *Total favorable features* | *1* | *5* | *0* | *0* | *1* | *0* | *1* | *6* | *0* |  | *8* | *18* | *0* | *3* | *7* | *4* | *11* | *25* | *4* |
|  | Inaccessible recreational facilities |  | 1 |  |  |  |  |  |  |  |  |  |  |  |  |  |  |  |  |  |
|  | Inaccessible shops and services for daily living |  |  | 1 |  |  |  |  |  |  |  |  |  |  |  |  |  |  |  |  |
|  | Inaccessible parks, natural features, and public open space |  |  |  |  | 1 |  |  |  |  |  |  |  |  |  |  |  |  |  |  |
|  | Unfriendly topography |  | 1 |  |  | 1 |  |  |  |  |  |  | 3 |  |  |  |  |  |  |  |
|  | *Total unfavorable features* | *0* | *2* | *1* | *0* | *2* | *0* | *0* | *4* | *1* |  | *0* | *3* | *0* | *0* | *0* | *0* | *0* | *3* | *0* |
|  | ***Destination accessibility total*** |  |  |  |  |  |  | ***2*** | ***10*** | ***0*** |  |  |  |  |  |  |  | ***11*** | ***28*** | ***4*** |
| Destination proximity | Proximate destination mix |  |  |  | 1 | 2 |  |  |  |  |  |  |  |  |  |  |  |  |  |  |
|  | Proximate parks, natural features, and public open space |  |  |  |  |  |  |  |  |  |  | 2 | 3 | 1 |  |  |  |  |  |  |
|  | Proximate recreational facilities |  | 5 |  |  |  |  |  |  |  |  |  |  |  |  |  |  |  |  |  |
|  | Proximate shops and services for daily living |  | 2 |  |  |  |  |  |  |  |  |  |  |  |  |  |  |  |  |  |
|  | *Total favorable features* |  | *7* |  | *1* | *2* |  | *1* | *9* | *0* |  | *2* | *3* | *1* |  |  |  | *2* | *3* | *1* |
|  | Distance to destination mix |  |  |  |  | 1 |  |  |  |  |  |  |  |  |  |  |  |  |  |  |
|  | Distance to parks, natural features, and public open space |  |  |  | 1 |  |  |  |  |  |  |  |  |  |  | 2 |  |  |  |  |
|  | Distance to recreational facilities |  |  |  |  | 3 |  |  |  |  |  |  |  |  |  |  |  |  |  |  |
|  | Distance to shops and services for daily living |  |  |  |  | 4 |  |  |  |  |  |  |  |  |  |  |  |  |  |  |
|  | Distance to walking/cycling infrastructure |  |  |  |  | 1 |  |  |  |  |  |  |  |  |  |  |  |  |  |  |
|  | *Total unfavorable features* |  |  |  | *1* | *9* |  | *1* | *9* | *0* |  |  |  |  |  | *2* |  | *0* | *2* | *0* |
|  | ***Destination proximity total*** |  |  |  |  |  |  | ***1*** | ***18*** | ***1*** |  |  |  |  |  |  |  | ***2*** | ***5*** | ***1*** |
| Disaster mitigation | General greenery |  | 1 |  |  |  |  |  |  |  |  |  |  |  |  |  |  |  |  |  |
|  | Trees and shade |  | 1 |  |  |  |  |  |  |  |  |  |  |  |  |  |  |  |  |  |
|  | ***Disaster mitigation total*** | *0* | *2* | *0* |  |  |  | ***0*** | ***2*** | ***0*** |  |  |  |  |  |  |  | ***0*** | ***0*** | ***0*** |
| Distance to public transport |  |  | 1 |  |  | 1 |  | ***0*** | ***2*** | ***0*** |  |  | 3 |  |  |  |  | ***0*** | ***3*** | ***0*** |
| Diverse housing and land use |  |  | *1* |  |  |  |  | ***0*** | ***1*** | ***0*** |  |  | *3* |  | *1* | *5* | *1* | ***1*** | ***8*** | ***1*** |
| Multi-component category | General environment supportive of physical activity | 1 | 1 |  |  | 2 |  |  |  |  |  |  |  |  |  |  |  |  |  |  |
|  | Walkability and walking-friendly environment | 1 | 1 |  |  | 2 |  |  |  |  |  |  |  |  |  | 1 | 3 |  |  |  |
|  | New urbanist designed development |  |  |  | 5 | 1 |  |  |  |  |  |  |  |  |  |  |  |  |  |  |
|  | *Total favorable features* | *2* | *2* |  | *5* | *5* |  | *7* | *8* | *0* |  |  |  |  |  | *1* | *3* | *0* | *1* | *3* |
|  | General environment compromising physical activity | 1 |  |  |  |  |  | *1* | *0* | *0* |  |  |  |  |  |  |  |  |  |  |
|  |  |  |  |  |  |  |  | ***7*** | ***8*** | ***1*** |  |  |  |  |  |  |  | ***0*** | ***1*** | ***3*** |

*Please note: “+” = positive relationship / increased physical activity, “0” = null relationship, “-“ = negative relationship / compromised physical activity*
