# Supplementary material for: A systematic review on the associations between the built environment and adult’s physical activity in global tropical and subtropical climate regions
Source: Int J Behav Nutr Phys Act. 2024 May 21;21:59. doi: 10.1186/s12966-024-01582-x (PMC11107026; doi:10.1186/s12966-024-01582-x)
Supplement: Supplementary file 7 — Additional File 7: Associations for main effects only including studies rated as high quality. [file 12966_2024_1582_MOESM7_ESM.docx]

**Additional File 6: Data synthesis for studies with stratified and moderation analysis**

*Table A6.1 Synthesis built environment and active transport by moderation variable*

| *11D-category* | *Sub-category* | *Moderator / stratum* | ***Perceived*** |
| --- | --- | --- | --- |
| Demand management | Parking support | Age | 0 [1] |
|  |  | Gender | 0 [1] |
| Density |  | Gender | Men (0 [1]), Women (0 [1]); |
| Design | Connectivity | Age | 0 [4] |
|  |  | Gender | 0 [4], women (0 [1]), men (0 [1]), |
|  |  | Neighborhood center configuration | 0 [1] |
|  | Walking/cycling infrastructure | Age | 0 [3] |
|  |  | Gender | 0 [5], men (+ [1]), women (0 [1]) |
|  |  | Socioeconomic status | 0 [2] |
|  |  | Children | No school going children (0 [1]), school-going children (0 [1]) |
|  |  | Vehicle ownership | No vehicle (0 [1]), vehicle owner (0 [1]) |
|  |  | Park access | # [1] greatest benefit: access to large park and high foot path provision |
|  |  | Neighborhood center configuration | 0 [1] |
|  | Unfavorable connectivity features | Neighborhood center configuration | 0 [1] |
|  | Unfavorable walking/cycling infrastructure | Age | 0 [1] |
|  |  | Gender | 0 [1] |
| Desirability | Crime safety | Gender | 0 [2], Men (0 [1]), Women (0 [2]) |
|  |  | Gender and age | Men 18-30 years (0 [2], + [1]); men 31-45 years (0 [2], + [1]); men 46-65 years (0 [1], + [1], - [1])  women 18-30 years (0 [2], + [1]); women 31-45 years (0 [2], + [1]); women 46-65 years (+ [2]) |
|  |  | Socio-economic status | Higher education (0 [2], + [1]); lower education (0 [2], + [1]), 0 [2]) |
|  |  | Marital status | Married (0 [2], + [1]); single (0 [2], + [1]) |
|  |  | Children | Kids in house (0 [2], + [1]); no kids in household (0 [2], + [1]) |
|  |  | Residency length | <8 years (0 [2], + [1]); ≥ 8 years <8 years (0 [2], + [1]) |
|  | Traffic safety | Age | 0 [2] |
|  |  | Gender | 0 [4]; men (0 [1]), women (0 [1]) |
|  |  | Gender and age | Men 18-30 years (0 [1]); men 31-45 years (+ [1]); men 46-65 years (0 [1])  women 18-30 years (+ [1]); women 31-45 years (+ [1]); women 46-65 years (+ [1]) |
|  |  | Socio-economic status | 0 [2] |
|  |  | Education | Lower education (+ [1]), higher education (+ [1]) |
|  |  | Marital status | Married (+ [1]); single (+ [1]) |
|  |  | Children | Kids in household (0 [1]); no kids in the house (0 [1]) |
|  |  | Residency length | <8 years (+ [1]); ≥ 8 years (+ [1]) |
|  | Aesthetics | Age | 0 [1] |
|  |  | Gender | 0 [3]; men (0 [1]), women (0 [1]) |
|  |  | Socio-economic status | 0 [2] |
|  | General safety | Age | 0 [1] |
|  |  | Gender | 0 [1] |
|  |  | Gender and age | Men 18-30 years (0 [1], + [2]); men 31-45 years (0 [1], + [2]); men 46-65 years (0 [1], + [2]); women 18-30 years (0 [1], + [2]); women 31-45 years (0 [1], + [2]); women 46-65 years (0 [1], + [2]) |
|  |  | Education | Lower education (+ [1]), higher education (+ [1]) |
|  |  | Marital status | Married (+ [1]); single (+ [1]) |
|  |  | Children | Kids in house (+ [1]); no kids in house (+[1]) |
|  |  | Residency length | <8 years (+ [1]); ≥ 8 years (+ [1]) |
|  | Criminality and crime concerns | Age | 0 [1] |
|  |  | Gender | 0 [1] |
|  | Traffic hazards and concerns | Age | 0 [1] |
|  |  | Gender | 0 [1] |
|  |  | Metro-scale traffic safety | 0 [1] |
|  |  | Neighborhood-scale traffic safety | # [1] opportunities without challenges (traffic hazards) increase utility cycling; challenges decrease cycling also when there are opportunities |
| Destination accessibility | Accessible parks, natural features, and public open space | Gender | 0 [4] |
|  |  | Socio-economic status | 0 [4] |
|  |  | Walkability | 0 [1] |
|  | Accessible shops and services | Gender | Men (0 [2]), Women (0 [2]) |
|  |  | Vehicle ownership | Vehicle owners (0 [2]), no vehicle (0 [2]) |
|  | Accessible destination mix | Age | 0 [2] |
|  |  | Gender | 0 [2] |
|  | Unfriendly topography | Age | 0 [1] |
|  |  | Gender | 0 [1] |
| Destination proximity | Proximate parks, natural facilities, and public open space | Gender | 0 [4] |
|  |  | Socioeconomic status | 0 [4] |
|  | Travel distance or time | Income | High (- [1]), Medium (- [1]), Low (- [1]) |
|  |  | Children | No school going children (0 [1]), school-going children (0 [1]) |
|  |  | Vehicle ownership | No vehicle (- [2], 0 [1]), vehicle owner (- [2], 0 [1]) |
|  |  | Urbanicity | Central (- [1]), Sub-urban (- [1]), Urban (- [1]) |
| Disaster mitigation | Greenery | Age | 0 [1] |
|  |  | Gender | 0 [1] |
| Distance to public transport | Proximity to public transport | Gender | 0 [2] |
|  |  | Socioeconomic status | 0 [2] |
| Diverse housing and land use |  | Age | 0 [1] |
|  |  | Gender | 0 [3] |
|  |  | Socio-economic status | 0 [2] |
|  |  | Income | High (0 [1]), Medium (+ [1]), Low (+ [1]) |
|  |  | Children | No school going children (0 [3]), school-going children (0 [2], + [1]) |
|  |  | Vehicle ownership | No vehicle (- [4], 0 [3], + [2]), vehicle owner (0 [5], + [4]) |
| Multi component | General physical activity friendly environment | Social support | 0 [1] |
|  | New urbanist-designed development | Importance of walkability for move | Little / no (0 [1]), moderate (+ [1]), very important / important (+ [1]) |
|  |  | Pre-move walkability | High (+ [1]), medium (+ [1]), low (+ [1]), very low (+ [1]); |
|  |  | Physical activity location | Inside neighborhood (- [1], 0 [2]), outside neighborhood (0 [3]) |
|  |  | Pre-move physical activity | # [1] largest decrease for people with high pre-move physical activity levels vs. slight increases / no change for people with low physical activity  Insufficiently active (+ [1]), sufficiently active (0 [1]) |
|  |  | Pre-move physical activity and physical activity location (inside / outside neighborhood) | 0 [6] |
|  |  | Pre-move social support | High (+ [2]), medium (+ [2]), low (+ [2]) |

*Please note: + = positive effect, 0 = null, - = negative effect in the stratum. The number in [] indicates that number of associations for the stratum. Numbers and numbers in brackets without any stratum (e.g., 0 [1]) refer to moderation analysis in this stratum: # = moderation effect; 0 = no moderation effect.*

*Table A6.2 Synthesis built environment and recreational physical activity moderation variables*

| *11D-category* | *Sub-category* | *Moderator* | ***Perceived*** |
| --- | --- | --- | --- |
| Demand management | Parking support | Age | 0 [1] |
|  |  | Gender | 0 [1] |
| Density |  | Gender | Men (0 [1]), Women (0 [1]); |
| Design | Connectivity | Age | 0 [4] |
|  |  | Gender | 0 [4], men [0], women [0] |
|  |  | Neighborhood center configuration | # [1] greatest benefit: access to main street center and high connected node ratio |
|  | Walking/cycling infrastructure | Age | 0 [3] |
|  |  | Gender | 0 [7], men (+ [1]), women (0 [1]) |
|  |  | Socioeconomic status | 0 [4] |
|  |  | Park access | # [1] greatest benefit: access to large park and high foot path provision |
|  |  | Neighborhood center configuration | # [1]: People without access to neighborhood center benefit most from infrastructure |
|  | Unfavorable connectivity features | Neighborhood center configuration | # [1] People without access to neighborhood center benefit from cul-de-sacs |
|  | Unfavorable walking/cycling infrastructure | Age | 0 [1] |
|  |  | Gender | 0 [1] |
| Desirability | Crime safety | Gender | 0 [4], men (0 [1]), women (0 [1]) |
|  |  | Gender and age | Men 18-30 years (0 [2], + [1]); men 31-45 years (0 [2], + [1]); men 46-65 years (0 [1], + [2])  Women 18-30 years (0 [1], + [2]); women 31-45 years (0 [1], + [2]); women 46-65 years (0 [1], + [2]) |
|  |  | Socio-economic status | 0 [4]  Higher education (0 [2], + [1]); lower education (0 [1], + [2]) |
|  |  | Children | Kids in house (0 [1], + [2]); no kids in household (0 [1], + [2]) |
|  |  | Marital status | Married (0 [1], + [2]); single (0 [2], + [1]) |
|  |  | Residency length | <8 years (0 [1], + [2]); ≥ 8 years (0 [1], + [2]) |
|  | Traffic safety | Age | 0 [2] |
|  |  | Gender | 0 [6]; men (0 [1]), women (- [2]) |
|  |  | Gender and age | Men 18-30 years (0 [1]); men 31-45 years (+ [1]); men 46-65 years (0 [1])  women 18-30 years (+ [1]); women 31-45 years (+ [1]); women 46-65 years (+ [1]) |
|  |  | Socio-economic status | 0 [4] |
|  |  | Education | Lower education (0 [1]), higher education (+ [1]) |
|  |  | Children | Kids in household (+ [1]); no kids in the house (+ [1]) |
|  |  | Marital status | Married (+ [1]); single (+ [1]) |
|  |  | Residency length | <8 years (+ [1]); ≥ 8 years (+ [1]) |
|  | Aesthetics | Age | 0 [1] |
|  |  | Gender | 0 [5], men (0 [2]), women (+ [1], 0 [1]) |
|  |  | Socio-economic status | 0 [3], high (0 [1]), medium-high (0 [1]), medium-low (0 [1]), low (+ [1]) |
|  | General safety | Age | 0 [1] |
|  |  | Gender | 0 [1]; men (0 [1]), women (+ [1]) |
|  |  | Gender and age | Men 18-30 years (+ [1]); men 31-45 years (0 [1]); men 46-65 years (0 [1]); women 18-30 years (+ [1]); women 31-45 years (+ [1]); women 46-65 years (+ [1]) |
|  |  | Education | Lower education (+ [1]), higher education (+ [1]) |
|  |  | Marital status | Married (+ [1]); single (+ [1]) |
|  |  | Children | Kids in house (+ [1]); no kids in house (+ [1]) |
|  |  | Residency length | <8 years (+ [1]); ≥ 8 years (+ [1]) |
|  | Criminality and crime concerns | Age | 0 [1] |
|  |  | Gender | 0 [1] |
|  | Traffic hazards and concerns | Age | 0 [1] |
|  |  | Gender | 0 [1] |
| Destination accessibility | Accessible parks, natural features, and public open space | Socio-economic status | 0 [8] |
|  |  | Nearest neighborhood open space characteristics | Largest neighborhood open space (0 [4]); most attractive neighborhood open space (+ [1], 0 [3]), nearest neighborhood open space (+ [1], 0 [3]) |
|  |  | Footpath to road ratio | # [1] most beneficial: High number of parks and high footpath provision |
|  | Accessible shops and services | Gender | Men (0 [2]), women (+ [1], 0 [1]) |
|  | Accessible destination mix | Age | 0 [2] |
|  |  | Gender | 0 [2] |
|  | Unfriendly topography | Age | 0 [1] |
|  |  | Gender | 0 [1] |
| Destination proximity | Distance to parks, natural features, and public open space | Neighborhood open space characteristics | Largest neighborhood open space (0 [2]); most attractive neighborhood open space (+ [1], 0 [1]), nearest neighborhood open space (0 [2]) |
|  | Proximate parks, natural facilities, and public open space | Gender | 0 [8] |
| Disaster mitigation | Greenery | Age | 0 [1] |
|  |  | Gender | 0 [1] |
|  | Park and park area | Gender | Men (0 [1]), women (+ [1]) |
| Distance to public transport | Proximity to public transport | Gender | 0 [4] |
|  |  | Socioeconomic status | 0 [4] |
| Diverse housing and land use |  | Age | 0 [1] |
|  |  | Gender | 0 [5] |
|  |  | Socio-economic status | 0 [4] |
| Multi component | General physical activity friendly environment | Social support | 0 [1] |
|  | New urbanist-designed development | Physical activity location | Inside neighborhood (+ [2], 0 [1]), outside neighborhood (- [2], 0 [1]) |
|  |  | Pre-move physical activity | # [2] Largest increase for people with low physical activity vs. slight increases / no changes in group with high physical activity  # [1] Largest decrease for people with high physical activity vs. slight decreases / no changes in group with low physical activity |
|  |  | Pre-move physical activity and physical activity location (inside / outside neighborhood) | 0 [2], # [1] largest increase for people with low physical activity inside neighborhood  # [3] Largest decrease for people with high physical activity vs. slight decreases / no changes in group with low physical activity |

*Please note: + = positive effect, 0 = null, - = negative effect in the stratum. The number in [] indicates that number of associations for the stratum. Numbers and numbers in brackets without any stratum (e.g., 0 [1]) refer to moderation analysis in this stratum: # = moderation effect; 0 = no moderation effect.*

*Table A6.3 Synthesis built environment and general physical activity / total walking and cycling*

| *11D-category* | *Sub-category* | *Moderator* | ***Perceived*** |
| --- | --- | --- | --- |
| Demand management | Parking support | Point of interest characteristics | Count (0 [1]), presence (0 [1]), size-weighted presence (0 [1]) |
| Design | Connectivity | Neighborhood center configuration | # [2] greatest benefit: access to main street center and high connected node ratio |
|  | Walking/cycling infrastructure | Park access | 0 [1], # [1] access to large park and high foot path provision |
|  |  | Neighborhood center configuration | 0 [1], # [1] people without access to neighborhood center benefit most from sidewalk-to-road-ratio |
|  |  | Point of interest characteristics | Count (0 [1]), presence (0 [1]), size-weighted presence (+ [1]) |
|  | Unfavorable connectivity features | Neighborhood center configuration | 0 [1], # [1] people without access to neighborhood center benefit most from cul-de-sacs |
| Desirability | Crime safety | Age | 0 [1] |
|  |  | Gender | 0 [1], men (0 [1]. + [1]), women (0 [1], + [1]) |
|  |  | Education | 0 [1] |
|  | Traffic safety | Gender | Men (0 [2]), women (0 [2]) |
|  | Aesthetics | Gender | Men (0 [1]), women (0 [1]) |
|  | General safety | Gender | Men (0 [1]), women (0 [1]) |
|  |  | Point of interest characteristics | Count (0 [1]), presence (0 [1]), size-weighted presence (0 [1]) |
|  | Criminality and crime concerns | Point of interest characteristics | Count (0 [1]), presence (0 [1]), size-weighted presence (0 [1]) |
|  | Traffic hazards and concerns | Point of interest characteristics | Count (0 [1]), presence (0 [1]), size-weighted presence (0 [1]) |
|  | Compromised general safety | Season | Fall (- [2]), spring (- [2]), summer (- [2]), winter(- [0]) |
|  |  | Season and no car | Fall (- [2]), spring (- [2]), summer (- [2]), winter(- [2]) |
| Destination accessibility | Accessible parks, natural features, and public open space | Gender | Men (0 [4]), women (0 [4]) |
|  |  | Point of interest characteristics | Count (0 [4]), presence (0 [4]), size-weighted presence (+ [3], 0 [1]) |
|  |  | Footpath to road ratio | 0 [1], # [1] greatest benefit: High number of parks and high footpath provision |
|  | Accessible recreational facilities | Point of interest characteristics | Count (0 [5], + [1]), presence (0 [3], + [3]), size-weighted presence (+ [1], 0 [5]) |
| Destination proximity | Proximate destination mix | Gender | 0 [4] |
|  | Proximate shops and services for daily living | Gender | 0 [2] |
|  | Travel distance or time | Point of interest characteristics | Count (0 [1]), presence (0 [1]), size-weighted presence (0 [1]) |
| Disaster mitigation | Greenery | Point of interest characteristics | Count (0 [1]), presence (+ [1]), size-weighted presence (0 [1]) |
|  | Trees and shade | Point of interest characteristics | Count (0 [1]), presence (+ [1]), size-weighted presence (0 [1]) |
|  | Park and park area | Gender | Men (0 [1]), women (0 [1]) |
| Multi component | General physical activity friendly environment | Social support | 0 [1] |
|  |  | Point of interest characteristics | Count (0 [1]), presence (0 [1]), size-weighted presence (+ [1]) |
|  | New urbanist-designed development | Importance of walkability for move | Little / none (0 [2]), moderate (0 [2]), very important / important (+ [2]) |
|  |  | Pre-move walkability | High (0 [2]), medium (+ [1], 0 [1]), low (+ [2]), very low (+ [2]) |
|  |  | Physical activity location | Inside neighborhood (- [1], 0 [2]), outside neighborhood (0 [3]) |
|  |  | Pre-move physical activity | Insufficiently active (+ [1], 0 [1]), sufficiently active (0 [1])  # [1] increase for people with low physical activity, decrease for people with high physical activity |
|  |  | Pre-move social support | High (0 [3], + [1]), medium (+ [3], 0 [1]), low (+ [3], 0 [1]) |

*Please note: + = positive effect, 0 = null, - = negative effect in the stratum. The number in [] indicates that number of associations for the stratum. Numbers and numbers in brackets without any stratum (e.g., 0 [1]) refer to moderation analysis in this stratum: # = moderation effect; 0 = no moderation effect.*

*Table A6.4. Synthesis built environment and meeting the WHO guidelines / MVPA*

| *11D-category* | *Sub-category* | *Moderator* | ***Perceived*** |
| --- | --- | --- | --- |
| Density |  | Gender | Men (0 [1]), women (0 [1]) |
|  |  | Neighborhood safety | 0 [4] |
|  |  | Park safety | 0 [4] |
| Design | Connectivity | Gender | 0 [3], men (0 [1]), women (0 [1]) |
|  |  | Socio-economic status | 0 [3] |
|  |  | Neighborhood safety | 0 [4] |
|  |  | Park safety | 0 [4] |
|  | Walking/cycling infrastructure | Gender | 0 [3], men (0 [1]), women (0 [1]) |
|  |  | Socioeconomic status | 0 [3] |
| Desirability | Crime safety | Gender | 0 [1], men (+ [4], 0 [1]), women (0 [4]) |
|  |  | Socio-economic status | 0 [3] |
|  | Traffic safety | Gender | 0 [3], men (+ [1], 0 [3]), women (0 [3]) |
|  |  | Socio-economic status | 0 [3] |
|  | Aesthetics | Gender | 0 [3], men (0 [3]), women (+ [2], 0 [1]) |
|  |  | Socio-economic status | 0 [1], # [1] tendency low socio-economic status for increased physical activity, in contrast to tendency for decreasing physical activity for high socio-economic status  High (0 [1]), medium-high (0 [1]), medium-low (0 [1]), low (1 [1]) |
|  | General safety | Gender | Men (0 [1]), women (0 [1]) |
|  | Criminality and crime concerns | Community gatedness | Gated (0 [1]), ungated (+ [1]) |
|  |  | Gender | Men (0 [2]), women (0 [1], + [1]) |
|  | Traffic hazards and concerns | Gender | Men (0 [1]), women (- [1]) |
| Destination accessibility | Accessible parks, natural features, and public open space | Socio-economic status | 0 [3] |
|  |  | Neighborhood safety | 0 [4] |
|  |  | Park safety | 0 [2], # [2] less physical activity if park was perceived as unsafe, but no association when perceived as safe |
|  | Accessible public transport | Neighborhood safety | 0 [2] |
|  |  | Park safety | 0 [2] |
|  | Accessible recreational facilities | Community gatedness | Gated [1], Ungated [1] |
|  | Accessible shops and services | Gender | 0 [3], men (0 [2]), women (+ [2]) |
|  |  | Socioeconomic status | 0 [3] |
|  |  | Neighborhood safety | 0 [4] |
|  |  | Park safety | 0 [4] |
|  | Inaccessible recreational facilities | Retirement status | Pre-retirement (0 [1]), retired (- [1]) |
| Destination proximity | Distance to parks, natural features, and public open space | Neighborhood safety | 0 [2] |
|  |  | Park safety | 0 [2] |
|  | Proximate parks, natural facilities, and public open space | Gender | 0 [2], men (0 [1]), women (+ [1]) |
|  |  | Socioeconomic status | 0 [3] |
| Distance to public transport | Proximity to public transport | Gender | 0 [3] |
|  |  | Socioeconomic status | 0 [3] |
| Diverse housing and land use |  | Gender | 0 [3] |
|  |  | Socioeconomic status | 0 [3] |
|  |  | Neighborhood safety | 0 [4] |
|  |  | Park safety | 0 [4] |
| Multi component | General physical activity friendly environment | Social support | 0 [2] |
|  | New urbanist-designed development | Importance of walkability for move | Little / no (0 [1]), moderate (0 [1]), very important / important (+ [1]) |
|  |  | Pre-move walkability | High (+ [1]), medium (+ [1]), low (+ [1]), very low (+ [1]); |
|  |  | Physical activity location | Inside neighborhood (- [1], 0 [2]), outside neighborhood (0 [3]) |
|  |  | Pre-move physical activity | 0 [1]  # [2] Largest increase for people with low physical activity  # [6] Largest increase for people with low physical activity; decrease high activity group  Insufficiently active (+ [1]), sufficiently active (- [1]) |
|  |  | Pre-move social support | High (0 [2]), medium (+ [2]), low (+ [2]) |
|  | Walkability and walking-friendly environment | Neighborhood safety | 0 [4] |
|  |  | Park safety | 0 [4] |

*Please note: + = positive effect, 0 = null, - = negative effect in the stratum. The number in [] indicates that number of associations for the stratum. Numbers and numbers in brackets without any stratum (e.g., 0 [1]) refer to moderation analysis in this stratum: # = moderation effect; 0 = no moderation effect.*
