# Supplementary material for: A systematic review on the associations between the built environment and adult’s physical activity in global tropical and subtropical climate regions
Source: Int J Behav Nutr Phys Act. 2024 May 21;21:59. doi: 10.1186/s12966-024-01582-x (PMC11107026; doi:10.1186/s12966-024-01582-x)
Supplement: Supplementary file 8 — Additional File 8: [file 12966_2024_1582_MOESM8_ESM.docx]

**Additional File 7**

**Association between built environment and physical activity**

**considering only high-quality studies**

*Table A7.1 Synthesis of built environment associations with active transport*

| *11D-category* | *Sub-category* | ***Perceived*** | | | ***Objective*** | | | ***Total*** | | |
| --- | --- | --- | --- | --- | --- | --- | --- | --- | --- | --- |
|  |  | *+* | *0* | *-* | *+* | *0* | *-* | *+* | *0* | *-* |
| Demand management | Parking restrictions |  | 1 |  |  |  |  | ***0*** | ***1*** | ***0*** |
| Density |  |  | *2* |  | *3* | *12* |  | ***0*** | ***15*** | ***0*** |
| Design | Connectivity |  | 7 |  | 3 | 5 |  |  |  |  |
|  | Walking/cycling infrastructure | 1 | 15 |  | 3 | 7 |  |  |  |  |
|  | Lot layout |  |  |  | 2 | 2 |  |  |  |  |
|  | *Total favorable features* | *1* | *22* | *0* | *8* | *14* | *0* | *9* | *36* | *0* |
|  | Unfavorable connectivity features |  | 2 |  | 1 | 11 |  |  |  |  |
|  | *Total unfavorable features* | *0* | *2* | *0* | *1* | *11* | *0* | *1* | *13* | *0* |
|  | ***Design total*** |  |  |  |  |  |  | ***9*** | ***49*** | ***1*** |
| Desirability | Crime safety | 1 | 5 |  |  |  |  |  |  |  |
|  | Traffic safety |  | 13 |  |  |  |  |  |  |  |
|  | Aesthetics |  | 9 |  |  |  |  |  |  |  |
|  | *Total favorable features* | *1* | *27* | *0* | *0* | *0* | *0* | *1* | *27* | *0* |
|  | Criminality and crime concerns |  | 2 |  |  | 1 |  |  |  |  |
|  | Traffic hazards and concerns |  | 2 |  |  | 1 |  |  |  |  |
|  | *Total unfavorable features* | *0* | *4* | *0* | *0* | *2* | *0* | *0* | *6* | *0* |
|  | ***Desirability total*** |  |  |  |  |  |  | ***1*** | ***33*** | ***0*** |
| Destination accessibility | Destination mix | 2 | 2 |  | 1 | 4 |  |  |  |  |
|  | Parks, natural features, and public open space | 1 | 1 |  | 4 | 12 |  |  |  |  |
|  | Public transport | 2 | 1 |  | 1 | 4 |  |  |  |  |
|  | Recreational facilities |  | 2 |  |  |  |  |  |  |  |
|  | Shops and services for daily living | 2 | 4 |  | 2 |  |  |  |  |  |
|  | Friendly topography |  | 1 | 1 |  |  |  |  |  |  |
|  | *Total favorable features* | *7* | *11* | *1* | *8* | *20* | *0* | *15* | *31* | *1* |
|  | Unfriendly topography |  | 2 |  |  | 1 |  |  |  |  |
|  | *Total unfavorable features* | *0* | *2* | *0* | *0* | *1* | *0* | *0* | *3* | *0* |
|  | ***Destination accessibility total*** |  |  |  |  |  |  | ***15*** | ***34*** | ***1*** |
| Destination proximity | Proximate destination mix | 1 |  |  | 1 |  |  |  |  |  |
|  | Proximate parks, natural features, and public open space |  |  |  | 3 | 13 |  |  |  |  |
|  | Proximate recreational facilities |  | 3 |  |  |  |  |  |  |  |
|  | Proximate shops and services for daily living |  | 1 |  |  | 6 |  |  |  |  |
|  | Proximate school/work |  |  |  |  | 3 | 1 |  |  |  |
|  | *Total favorable features* | 1 | 4 | 0 | 4 | 22 | 1 | *5* | *26* | *1* |
|  | Distance to recreational facilities |  |  |  |  | 5 |  |  |  |  |
|  | Distance to shops and services for daily living |  |  |  |  | 2 |  |  |  |  |
|  | Distance to school/work |  |  |  |  | 2 |  |  |  |  |
|  | General travel distance or time |  |  | 2 |  |  |  |  |  |  |
|  | *Total unfavorable features* | *0* | *0* | *2* | *0* | *9* | *0* | *0* | *9* | *2* |
|  | ***Destination proximity total*** |  |  |  |  |  |  | ***7*** | ***35*** | ***1*** |
| Disaster mitigation | General greenery |  | 1 |  |  |  |  |  |  |  |
|  | Trees and shade |  |  |  | 1 | 3 |  |  |  |  |
|  | Parks and park area |  |  |  |  | 4 |  |  |  |  |
|  | ***Disaster mitigation total*** | *0* | *1* | *0* | *1* | *7* | *0* | *1* | *8* | *0* |
| Distance to public transport |  |  |  |  |  | 10 |  | **0** | **10** | **0** |
| Diverse housing and land use |  | *1* |  |  | *1* |  |  | **2** | **0** | **0** |
| Multi-component category | Walkability and walking-friendly environment |  |  |  | 9 | 5 |  |  |  |  |
|  | New urbanist designed development |  |  |  | 9 | 14 |  |  |  |  |
|  | **Multicomponent category total** | 0 | 0 | 0 | 18 | 19 | 0 | ***18*** | ***19*** | ***0*** |

*Note: “+” = positive relationship / supports of physical activity, “0” = null relationship, “-“ = negative relationship / compromises physical activity*

*Table A7.2. Synthesis of built environment associations with recreational physical activity*

| *11D-category* | *Sub-category* | ***Perceived*** | | | ***Objective*** | | | ***Total*** | | |
| --- | --- | --- | --- | --- | --- | --- | --- | --- | --- | --- |
|  |  | *+* | *0* | *-* | *+* | *0* | *-* | *+* | *0* | *-* |
| Demand  management | Parking restrictions |  | 1 |  |  |  |  | ***0*** | ***1*** | ***0*** |
| Density |  |  | *1* |  | *1* | *13* |  | ***1*** | ***14*** | ***0*** |
| Design | Connectivity | 2 | 4 | 2 | 2 | 6 |  |  |  |  |
|  | Walking/cycling infrastructure | 3 | 15 |  | 1 | 8 |  |  |  |  |
|  | Lot layout |  |  |  | 2 | 2 |  |  |  |  |
|  | *Total favorable features* | *5* | *19* | *2* | *5* | *16* | *0* | *10* | *35* | *2* |
|  | Unfavorable connectivity features |  |  |  |  | 11 | 1 |  |  |  |
|  | Unfavorable walking/cycling infrastructure |  | 1 | 1 |  |  |  |  |  |  |
|  | *Total unfavorable features* | *0* | *1* | *1* | *0* | *11* | *1* | *0* | *12* | *2* |
|  | ***Design total*** |  |  |  |  |  |  | ***12*** | ***47*** | ***2*** |
| Desirability | Crime safety | 3 | 4 | 1 |  |  |  |  |  |  |
|  | Traffic safety | 2 | 13 |  |  |  |  |  |  |  |
|  | Aesthetics | 4 | 7 |  |  |  |  |  |  |  |
|  | *Total favorable features* | *9* | *24* | *1* | *0* | *0* | *0* | *9* | *24* | *1* |
|  | Compromised general safety |  |  |  |  |  |  |  |  |  |
|  | Criminality and crime concerns |  | 2 |  |  | 1 |  |  |  |  |
|  | Traffic hazards and concerns |  | 1 | 1 | 1 |  |  |  |  |  |
|  | *Total unfavorable features* | *0* | *3* | *1* | *1* | *1* | *0* | *1* | *4* | *1* |
|  | ***Desirability total*** |  |  |  |  |  |  | ***10*** | ***28*** | ***2*** |
| Destination accessibility | Destination mix | 2 | 1 |  | 1 | 3 |  |  |  |  |
|  | Parks, natural features, and public open space | 1 | 1 |  | 2 | 14 |  |  |  |  |
|  | Public transport |  | 1 |  |  | 4 |  |  |  |  |
|  | Recreational facilities |  | 2 |  |  |  |  |  |  |  |
|  | Shops and services for daily living | 2 | 4 |  |  | 2 |  |  |  |  |
|  | Friendly topography |  | 2 |  |  |  |  |  |  |  |
|  | *Total favorable features* | *5* | *11* | *0* | *3* | *23* | *0* | *8* | *34* | *0* |
|  | Unfriendly topography |  | 2 |  |  | 1 |  |  |  |  |
|  | *Total unfavorable features* | *0* | *2* | *0* | *0* | *1* | *0* | *0* | *3* | *0* |
|  | ***Destination accessibility total*** |  |  |  |  |  |  | ***8*** | ***37*** | ***0*** |
| Destination proximity | Proximate destination mix |  | 1 |  |  | 1 |  |  |  |  |
|  | Proximate parks, natural features, and public open space | 4 |  |  | 2 | 21 |  |  |  |  |
|  | Proximate school/work |  |  | 1 |  | 4 |  |  |  |  |
|  | Proximate recreational facilities |  | 4 |  |  |  |  |  |  |  |
|  | Proximate shops and services for daily living |  |  |  |  | 6 |  |  |  |  |
|  | *Total favorable features* | *4* | *5* | *1* | *2* | *32* | *0* | *6* | *37* | *1* |
|  | Travel time or distance |  |  | 1 |  |  |  |  |  |  |
|  | Distance to recreational facilities |  | 1 |  | 1 | 4 |  |  |  |  |
|  | Distance to shops and services for daily living |  |  |  |  | 2 |  |  |  |  |
|  | Distance to school/work |  |  |  |  | 2 |  |  |  |  |
|  | *Total unfavorable features* | *0* | *1* | *1* | *1* | *8* | *0* | *1* | *9* | *1* |
|  | ***Destination proximity total*** |  |  |  |  |  |  | ***7*** | ***46*** | ***2*** |
| Disaster mitigation | General greenery |  | 2 |  |  |  |  |  |  |  |
|  | Trees and shade |  |  |  |  | 4 |  |  |  |  |
|  | Parks and park area |  |  |  |  | 4 |  |  |  |  |
|  | ***Disaster mitigation total*** | *0* | *2* | *0* | *0* | *8* | *0* | ***0*** | ***10*** | ***0*** |
| Distance to public transport |  |  |  |  |  | 9 |  | ***0*** | ***9*** | ***0*** |
| Diverse housing and land use |  |  |  |  |  | *12* |  | ***0*** | ***12*** | ***0*** |
| Multi-component category | General environment supportive of physical activity | 2 |  |  |  | 2 |  |  |  |  |
|  | Walkability and walking-friendly environment |  |  |  | 4 | 9 | 1 |  |  |  |
|  | New urbanist designed development |  |  |  | 8 | 15 |  |  |  |  |
|  | **Multicomponent category total** | *2* | *0* | *0* | *12* | *26* | *1* | ***14*** | ***26*** | ***1*** |

*Note: “+” = positive relationship / increased physical activity, “0” = null relationship, “-“ = negative relationship / compromised physical activity*

*Table A7.3. Synthesis of built environment associations with total walking and cycling*

| *11D-category* | *Sub-category* | ***Perceived*** | | | ***Objective*** | | | ***Total*** | | |
| --- | --- | --- | --- | --- | --- | --- | --- | --- | --- | --- |
|  |  | *+* | *0* | *-* | *+* | *0* | *-* | *+* | *0* | *-* |
| Density |  |  |  |  | *1* | *11* |  | ***1*** | ***11*** | ***0*** |
| Design | Connectivity |  | 2 |  |  | 6 |  |  |  |  |
|  | Walking/cycling infrastructure | 1 | 7 |  | 1 | 7 |  |  |  |  |
|  | Lot layout |  |  |  | 3 | 3 |  |  |  |  |
|  | *Total favorable features* | *1* | *9* | *0* | *4* | *16* | *0* | *5* | *25* | *0* |
|  | Unfavorable connectivity features |  |  |  | 1 | 17 |  |  |  |  |
|  | *Total unfavorable features* | *0* | *0* | *0* | *1* | *17* | *0* | *1* | *17* | *0* |
|  | ***Design total*** |  |  |  |  |  |  | ***5*** | ***42*** | ***1*** |
| Desirability | General safety |  | 2 |  |  |  |  |  |  |  |
|  | Crime safety | 1 | 3 |  |  |  |  |  |  |  |
|  | Traffic safety |  | 7 |  |  |  |  |  |  |  |
|  | Aesthetics | 1 | 3 |  |  |  |  |  |  |  |
|  | *Total favorable features* | *2* | *15* | *0* | *0* | *0* | *0* | *2* | *15* | *0* |
|  | Criminality and crime concerns |  | 1 |  |  | 1 |  |  |  |  |
|  | Traffic hazards and concerns |  |  | 1 |  | 1 |  |  |  |  |
|  | *Total unfavorable features* | *0* | *1* | *1* | *0* | *2* | *0* | *0* | *3* | *1* |
|  | ***Desirability total*** |  |  |  |  |  |  | ***3*** | ***18*** | ***0*** |
| Destination accessibility | Parks, natural features, and public open space |  |  |  | 2 | 22 |  |  |  |  |
|  | Public transport |  |  |  |  | 6 |  |  |  |  |
|  | Shops and services for daily living |  | 2 |  | 1 | 2 |  |  |  |  |
|  | Friendly topography |  | 2 |  |  |  |  |  |  |  |
|  | *Total favorable features* | *0* | *4* | *0* | *3* | *30* | *0* | *3* | *34* | *0* |
|  | Unfriendly topography |  |  |  |  | 1 |  |  |  |  |
|  | *Total unfavorable features* | *0* | *0* | *0* | *0* | *1* | *0* | *0* | *1* | *0* |
|  | ***Destination accessibility total*** |  |  |  |  |  |  | ***3*** | ***35*** | ***0*** |
| Destination proximity | Proximate destination mix |  | 1 |  |  | 9 |  |  |  |  |
|  | Proximate parks, natural features, and public open space | 1 |  |  | 2 | 22 |  |  |  |  |
|  | Proximate school/work |  |  |  |  | 5 | 1 |  |  |  |
|  | Proximate recreational facilities |  | 3 |  |  |  |  |  |  |  |
|  | Proximate shops and services for daily living |  | 1 |  | 1 | 26 | 2 |  |  |  |
|  | *Total favorable features* | *1* | *5* | *0* | *3* | *62* | *3* | *4* | *67* | *3* |
|  | Distance to recreational facilities |  |  |  |  | 6 |  |  |  |  |
|  | Distance to shops and services for daily living |  |  |  |  | 2 |  |  |  |  |
|  | Distance to school/work |  |  |  |  | 3 |  |  |  |  |
|  | *Total unfavorable features* | *0* | *0* | *0* | *0* | *11* | *0* | *0* | *11* | *0* |
|  | ***Destination proximity total*** |  |  |  |  |  |  | ***4*** | ***78*** | ***3*** |
| Disaster mitigation | Trees and shade |  |  |  | *1* | *5* |  |  |  |  |
|  | Parks and park area |  |  |  |  | *6* |  |  |  |  |
|  | ***Disaster mitigation total*** | *0* | *0* | *0* | *1* | *11* | *0* | ***1*** | ***11*** | ***0*** |
| Distance to public transport |  |  |  |  |  | 13 |  | ***0*** | ***13*** | ***0*** |
| Diverse housing and land use |  |  |  |  |  | *12* |  | ***0*** | ***12*** | ***0*** |
| Multi-component category | General environment supportive of physical activity | 1 |  |  |  |  |  |  |  |  |
|  | Walkability and walking-friendly environment |  |  |  | 1 | 5 |  |  |  |  |
|  | New urbanist designed development |  |  |  | 4 | 6 |  |  |  |  |
|  | **Multicomponent category** | *1* | *0* | *0* | *5* | *11* | *0* | ***6*** | ***11*** | ***0*** |

*Note: “+” = positive relationship / increased physical activity, “0” = null relationship, “-“ = negative relationship / compromised physical activity*

*Table A7.4. Synthesis of built environment associations with MVPA*

| *11D-category* | *Sub-category* | ***Perceived*** | | | ***Objective*** | | | ***Total*** | | |
| --- | --- | --- | --- | --- | --- | --- | --- | --- | --- | --- |
|  |  | *+* | *0* | *-* | *+* | *0* | *-* | *+* | *0* | *-* |
| Density |  |  | *1* |  |  |  |  | ***0*** | ***1*** | ***0*** |
| Design | Connectivity | 1 | 1 |  |  |  |  |  |  |  |
|  | Walking/cycling infrastructure | 1 | 4 |  |  |  |  |  |  |  |
|  | ***Design total*** | *2* | 5 | *0* | *0* | *0* | *0* | ***2*** | ***5*** | ***0*** |
| Desirability | General safety |  | 1 |  |  |  |  |  |  |  |
|  | Crime safety | 1 | 2 |  |  |  |  |  |  |  |
|  | Traffic safety | 1 | 2 |  |  |  |  |  |  |  |
|  | Aesthetics |  | 3 |  |  |  |  |  |  |  |
|  | *Total favorable features* | *2* | *8* | *0* | *0* | *0* | *0* | *2* | *8* | *0* |
|  | Traffic hazards and concerns |  |  | 1 |  | 1 |  |  |  |  |
|  | *Total unfavorable features* | *0* | *0* | *1* | *0* | *1* | *0* | *0* | *1* | *1* |
|  | ***Desirability total*** |  |  |  |  |  |  | ***3*** | ***9*** | ***0*** |
| Destination accessibility | Recreational facilities |  | 1 |  |  |  |  |  |  |  |
|  | Shops and services for daily living | 1 | 2 |  |  |  |  |  |  |  |
|  | Friendly topography |  | 1 |  |  |  |  |  |  |  |
|  | *Total favorable features* | *1* | *4* | *0* | *0* | *0* | *0* | *1* | *4* | *0* |
|  | Unfriendly topography |  |  |  |  | 1 |  |  |  |  |
|  | *Total unfavorable features* | *0* | *0* | *0* | *0* | *1* | *0* | *0* | *1* | *0* |
|  | ***Destination accessibility total*** |  |  |  |  |  |  | ***1*** | ***5*** | ***0*** |
| Destination proximity | Proximate destination mix |  |  |  |  | 1 |  |  |  |  |
|  | Proximate parks, natural features, and public open space | 1 |  |  |  |  |  |  |  |  |
|  | Proximate recreational facilities |  | 3 |  |  |  |  |  |  |  |
|  | *Total favorable features* | *1* | *3* | *0* | *0* | *1* | *0* | *1* | *4* | *0* |
|  | Distance to recreational facilities |  |  |  |  | 3 |  |  |  |  |
|  | Distance to shops and services for daily living |  |  |  |  | 2 |  |  |  |  |
|  | *Total unfavorable features* | *0* | *0* | *0* | *0* | *5* | *0* | *0* | *5* | *0* |
|  | ***Destination proximity total*** |  |  |  |  |  |  | ***1*** | ***9*** | ***0*** |
| Distance to public transport |  |  |  |  |  | 1 |  | ***0*** | ***1*** | ***0*** |
| Multi-component category | Walkability and walking-friendly environment |  |  |  |  | 1 |  | *0* | *1* | *0* |
|  | ***Multi-component category total*** |  |  |  |  |  |  | ***0*** | ***1*** | ***0*** |

*Note: “+” = positive relationship / increased physical activity, “0” = null relationship, “-“ = negative relationship / compromised physical activity*
